# Patient-reported outcomes from a randomized trial of neoadjuvant atezolizumab-chemotherapy in early triple-negative breast cancer

**DOI:** 10.1038/s41523-022-00457-3

**Published:** 2022-09-19

**Authors:** Carlos H. Barrios, Shigehira Saji, Nadia Harbeck, Hong Zhang, Kyung H. Jung, Sheetal Patel, Shilpen Patel, Anh Nguyen Duc, Mario Liste-Hermoso, Stephen Y. Chui, Elizabeth A. Mittendorf

**Affiliations:** 1 Centro de Pesquisa em Oncologia, Hospital São Lucas da PUCRS, Porto Alegre, RS Brazil; 2 Fukushima Medical University, Fukushima, Japan; 3 Breast Center, Department of Obstetrics and Gynecology and CCCLMU, LMU University Hospital, Munich, Germany; 4 Memorial Sloan Kettering Cancer Center, New York, NY USA; 5 Asan Medical Center, University of Ulsan College of Medicine, Seoul, Korea; 6 Genentech, Inc., South San Francisco, CA USA; 7 F. Hoffmann-La Roche Ltd, Basel, Switzerland; 8 Dana-Farber/Brigham and Women’s Cancer Center, Boston, MA USA

**Keywords:** Breast cancer, Outcomes research

## Abstract

Patient-reported outcomes data assessing patients’ experience of immunotherapy treatment burden in potentially curable early-stage triple-negative breast cancer (TNBC) are lacking. These patient-reported data inform clinical benefit and decision-making for adding atezolizumab to neoadjuvant chemotherapy in early-stage TNBC. IMpassion031 (NCT03197935) randomly assigned patients with stage II/III TNBC (T2–T4d primary tumors) to 5 cycles (4 weeks/cycle) of every 2-week neoadjuvant atezolizumab 840 mg or placebo with weekly *nab*-paclitaxel (3 cycles) followed by every 2-week dose-dense doxorubicin+cyclophosphamide (2 cycles). After surgery, the atezolizumab-chemotherapy arm received atezolizumab 1200 mg every 3 weeks (11 cycles). The placebo-chemotherapy arm was observed under standard of care. To assess treatment burden from the patients’ perspective, which comprised measures of the treatment-related impact on patients’ functioning and health-related quality of life (HRQoL), as well as patients’ experience of treatment-related symptoms plus their associated bother, patients completed the EORTC QLQ-C30 and FACT-G single-item GP5. Predefined secondary endpoints included mean and mean change from baseline values in the QLQ-C30 function (role and physical) and global health status/quality of life scales. Exploratory endpoints included mean and mean change from baseline in treatment-related symptoms, and treatment side effect bother. Mean physical, role function, and HRQoL were similar between arms at baseline and throughout treatment. In the neoadjuvant period, both arms exhibited clinically meaningful declines of similar magnitude from baseline in physical, role function, and HRQoL, and reported similar treatment side effect to bother at each visit. Improved pathologic complete response from adding atezolizumab to neoadjuvant chemotherapy for early-stage TNBC occurred without imposing additional treatment burden on patients.

## Introduction

In early-stage breast cancer (stage I–III), 10% to 20% of patients present with triple-negative breast cancer (TNBC)^1^. Combination chemotherapy regimens including anthracyclines and taxanes are given with curative intent in early TNBC, with up to 60% of patients experiencing long-term recurrence-free outcomes^2^. However, 30% to 40% of patients with early-stage TNBC treated with a standard neoadjuvant anthracycline-taxane regimen develop metastatic breast cancer within 5 years and eventually die, even when adjuvant capecitabine is added for those with residual disease after surgery^3–5^.

The randomized, double-blind, phase III IMpassion031 study aimed to improve outcomes by adding the programmed death-ligand 1 (PD-L1)–targeted checkpoint inhibitor atezolizumab to a neoadjuvant regimen of *nab*-paclitaxel followed by dose-dense doxorubicin plus cyclophosphamide^6^. The rate of pathologic complete response (pCR) in the breast and lymph nodes was significantly improved in patients treated with atezolizumab plus chemotherapy vs placebo plus chemotherapy, regardless of baseline tumor PD-L1 status. During the neoadjuvant period, any grade and grade 3 or 4 treatment-related adverse events were similar between the atezolizumab-chemotherapy and placebo-chemotherapy arms (99% vs 99% and 57% vs 53%, respectively), while the incidence of treatment-related serious adverse events was higher in the atezolizumab-chemotherapy arm (23% vs 16%). Grade 3 or 4 treatment-related adverse events occurring in ≥10% of patients in either the atezolizumab-chemotherapy or placebo-chemotherapy arm involved reduced neutrophil counts (neutropenia, 23% vs 22%; decreased neutrophil count, 12% vs 11%; and febrile neutropenia, 11% vs 9%, respectively). The addition of atezolizumab did not increase treatment-related discontinuation rates^6^.

Early breast cancer is localized and largely asymptomatic. Therefore, treatment-related toxicities and symptoms are the primary effectors of how patients feel and function, often with significant impact^7^. Importantly, chemotherapy toxicities that physicians may regard as clinically manageable can significantly increase patients’ distress and interfere with daily life. This can lead to reduced treatment adherence or early treatment discontinuation, with subsequent decreased efficacy outcomes^8^.

The evaluation of treatment burden using patient-reported outcomes (PROs) therefore supports the overall risk-benefit assessment of a therapeutic regimen and informs physician and patient decision-making^9–11^. There are currently no published PRO data for checkpoint inhibitors in early breast cancer, particularly in the neoadjuvant setting, leaving patients with inadequate information regarding experience and duration of treatment-related symptoms. Here, we report results for prespecified PRO endpoints in the IMpassion031 study, investigating the impact of the addition of atezolizumab on patient-reported treatment burden. This treatment burden comprised measures of the treatment-related impact on patients’ functioning and overall health-related quality of life (HRQoL), as well as patients’ experience of treatment-related symptoms plus their associated bother.

## Results

### Patients

Overall, 333 patients were randomized (165 to atezolizumab-chemotherapy, 168 to placebo-chemotherapy)^6^. The population that was PRO evaluable for the European Organisation for the Research and Treatment of Cancer (EORTC) quality of life questionnaire (QLQ-C30) analyses included 161 patients in the atezolizumab-chemotherapy arm and 167 in the placebo-chemotherapy arm (Supplementary Fig. 1). The Functional Assessment of Cancer Therapy-General (FACT-G) GP5 PRO-evaluable population included 152 patients in the atezolizumab-chemotherapy arm and 161 in the placebo-chemotherapy arm. At the clinical cutoff date of 3 April 2020, the median duration of follow-up was 20.6 months (interquartile range, 8.7–24.9 months) in the atezolizumab-chemotherapy arm and 19.8 months (interquartile range, 8.1–24.5 months) in the placebo-chemotherapy arm. During the adjuvant period, patients in the placebo-chemotherapy arm received standard-of-care treatment, which potentially included capecitabine in cases where pathologic complete response was not found; however, the majority of patients were no longer receiving any further systemic treatment.

### Compliance

Very high rates of compliance with completion of the PRO instruments were seen at baseline (C1D1) among all randomized patients in both study arms (EORTC QLQ-30, 100%; FACT-G GP5, 98%) and throughout the neoadjuvant (>90% and >88%, respectively) and adjuvant (>89% and >88%) periods (Supplementary Fig. 2). At this data cutoff, with a median follow-up of <21 months, compliance rates post treatment discontinuation were similar in both arms during follow-up (Supplementary Fig. 2). The rates stayed above 85% until the month 9 follow-up visit after treatment discontinuation (i.e., ~21 months of follow-up from study start).

### Physical and role function

**Fig. 1:**
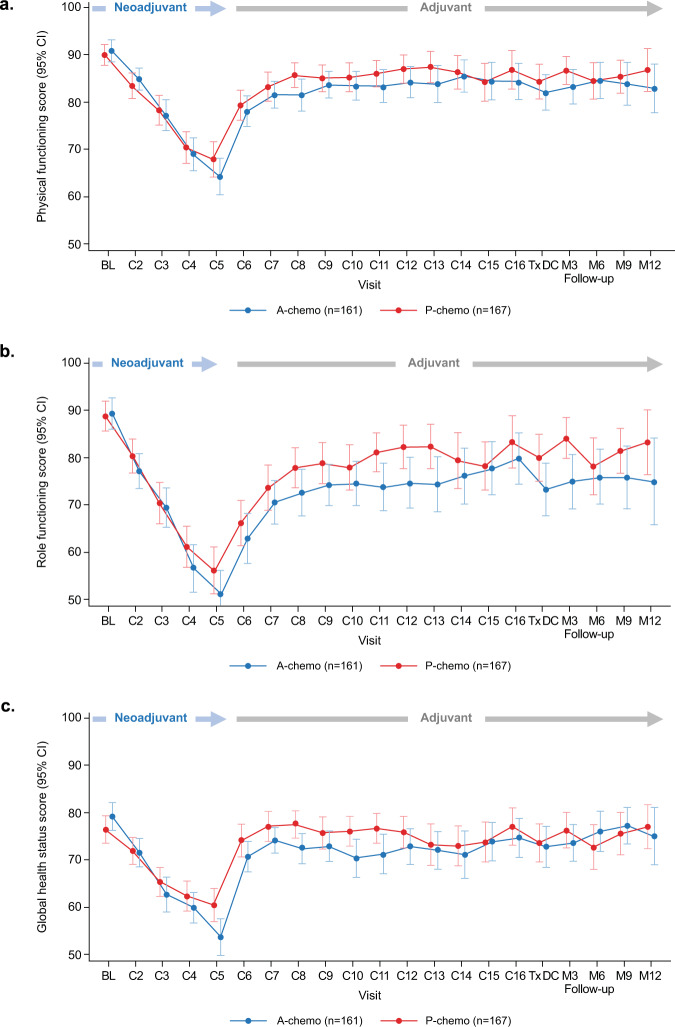
Mean values at each time point for physical function, role function, and HRQoL. Error bars indicate 95% confidence intervals. Mean values at each time point for **a** physical function, **b** role function, and **c** HRQoL was measured using the EORTC QLQ-C30. A atezolizumab, C cycle, M month, P placebo, Tx DC treatment discontinuation.

**Fig. 2:**
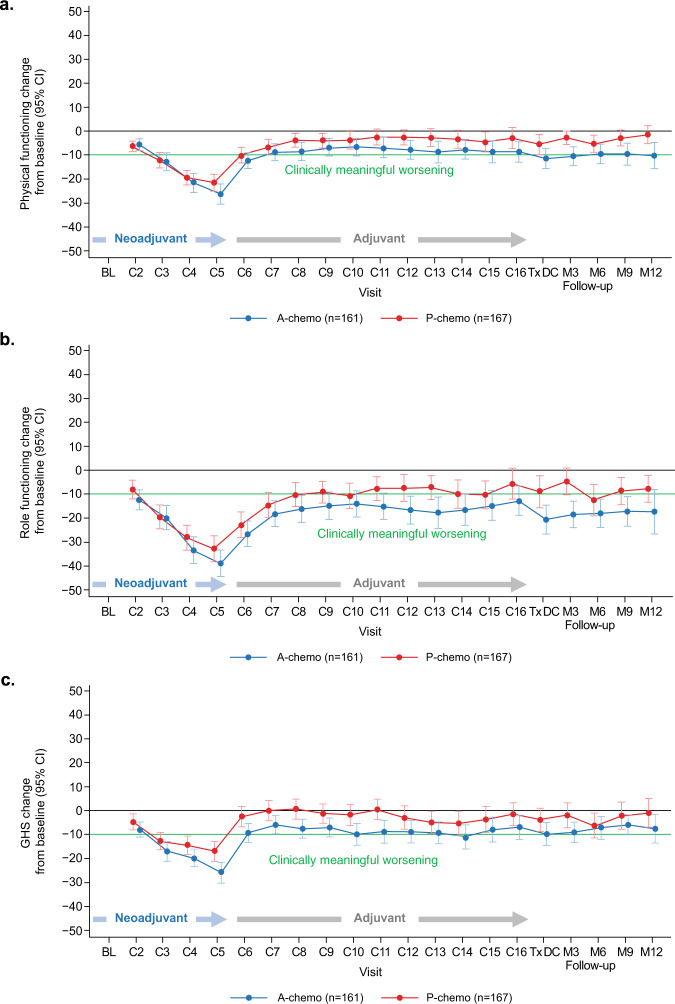
Mean change from baseline values at each time point in physical function, role function, and HRQoL. Error bars indicate 95% confidence intervals. Mean change from baseline values at each time point in **a** physical function, **b** role function, and **c** HRQoL was measured using the EORTC QLQ-C30. A atezolizumab, C cycle, GHS global health status, ﻿M month, P placebo, Tx DC treatment discontinuation.

Baseline mean values for the physical function scale of the EORTC QLQ-30 were high and similar based on overlapping 95% CIs in both arms: 90.9 (95% CI, 88.5–93.2) in the atezolizumab-chemotherapy arm and 90.0 (95% CI, 87.8–92.2) in the placebo-chemotherapy arm (Supplementary Table 1). The 95% CIs continued to overlap between arms across time points throughout the on-treatment and follow-up periods (Fig. 1a). Mean change from baseline values in physical function showed a similar overlap of 95% CIs between the treatment arms (Fig. 2a), with no differential treatment effect observed over time between arms (*P* = 0.36; Supplementary Table 2). In the neoadjuvant period, patients in both arms experienced clinically meaningful deterioration (a ≥10-point decrease from baseline) in physical functioning beginning at cycle 3 and sustained through cycle 5. During the adjuvant period (cycles 6–16), a rebound in physical function was observed, with patients in both arms beginning to experience a gradual stability (defined as mean change in values within a 10-point range) in physical function from cycle 7.

Results for role function were similar: baseline mean values for the role function scale of the EORTC QLQ-C30 were high with overlapping CIs observed between treatment arms, 89.4 (95% CI, 86.1–92.8) with atezolizumab-chemotherapy and 88.9 (95% CI, 85.7–92.0) with placebo-chemotherapy (Supplementary Table 1). The 95% CIs for mean role function values overlapped between the two arms across time points during treatment and follow-up periods (Fig. 1b). Similarly, 95% CIs for mean changes from baseline values in role function overlapped between the treatment arms at each time point (Fig. 2b), with no differential treatment effect observed over time between arms (*P* = 0.77; Supplementary Table 2). During the neoadjuvant period, clinically meaningful deterioration in role function started at cycle 2 in the atezolizumab-chemotherapy group and at cycle 3 in the placebo-chemotherapy group, continuing until cycle 5 in both groups. Impact on role function was sustained during the adjuvant period, but values stabilized from cycle 9 in the placebo arm.

### HRQoL

Baseline mean values for the global health status (GHS)/QoL scale of the EORTC QLQ-30 were high and similar with overlapping CIs between treatment groups: 79.2 (95% CI, 76.3–82.1) with atezolizumab-chemotherapy and 76.5 (95% CI, 73.5–79.4) with placebo-chemotherapy (Supplementary Table 1). The 95% CIs for mean values overlapped between the 2 arms across time points during treatment and follow-up (Fig. 1c). There was no differential treatment effect observed over time between arms (*P* = 0.50; Supplementary Table 2). In both arms, mean changes in HRQoL from baseline showed a clinically meaningful decline in the neoadjuvant period from cycle 3 to cycle 5, with a rebound in the adjuvant period and stabilization from cycle 6 (Fig. 2c).

### Exploratory PRO endpoints

**Fig. 3:**
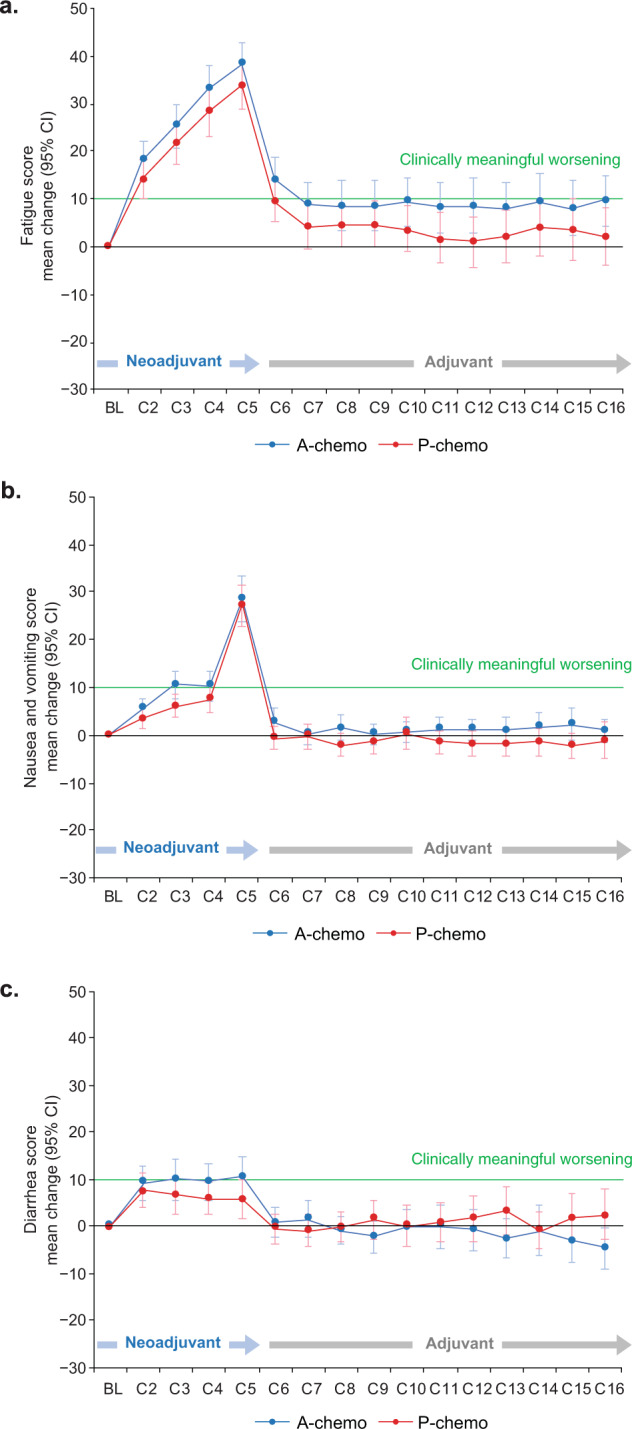
Mean change from baseline values at each time point in fatigue, nausea and vomiting, and diarrhea. Error bars indicate 95% confidence intervals. Mean change from baseline values at each time point in **a** fatigue, **b** nausea and vomiting, and **c** diarrhea was measured using the EORTC QLQ-C30. A atezolizumab, BL baseline, C cycle, P placebo.

Based on the EORTC QLQ-C30 treatment-related symptom items, mean values for most symptoms, including fatigue, diarrhea, nausea, and vomiting, increased in both treatment arms from baseline to cycle 5 as expected (Supplementary Fig. 3). In the neoadjuvant period, mean change from baseline values for symptoms in both treatment arms demonstrated clinically meaningful worsening of treatment symptoms, peaking at cycle 5 (except for pain, which peaked at cycle 4) (Fig. 3 and Supplementary Fig. 4). During the adjuvant period, mean treatment-related symptom values in both arms were similar to those at baseline for most symptoms, except fatigue, which remained above baseline levels and was more pronounced in the atezolizumab-chemotherapy arm. EORTC QLQ-C30 emotional and social functioning data showed no difference between arms across both the neoadjuvant and adjuvant periods (Supplementary Fig. 4).

**Fig. 4:**
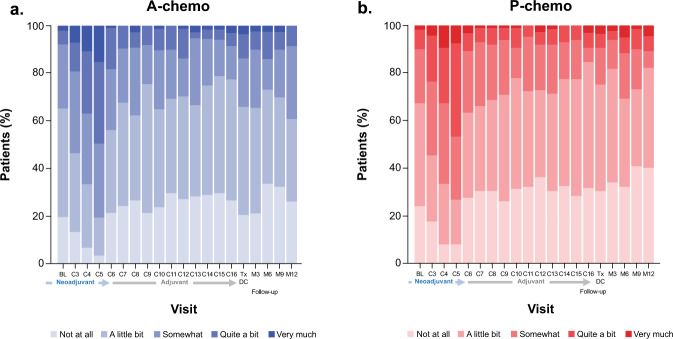
Proportion of patients selecting each response option by visit in the FACT-G GP5 item in the atezolizumab-chemotherapy and placebo-chemotherapy arms. Proportion of patients selecting each response option by a visit in the FACT-G GP5 item (“I am bothered by side effects of treatment”) in **a** the atezolizumab-chemotherapy arm and **b** the placebo-chemotherapy arm. BL baseline, C cycle, M month, Tx DC treatment discontinuation.

A similar proportion of patients in each arm reported each response option of the FACT-G GP5 treatment side-effect bother item at baseline (cycle 2 day 1), with the majority of patients (65–67%) reporting their level of bother to be “not at all” or “a little bit” (Supplementary Table 3). During the neoadjuvant period, a similar proportion of patients in both arms reported increased bother from side effects at subsequent visits, with 66.0% of patients in the atezolizumab-chemotherapy arm and 65.4% of patients in the placebo-chemotherapy arm reporting their level of bother to be “somewhat” or “quite a bit” by cycle 5 (Fig. 4). During the adjuvant period and follow-up, no additional side-effect bother was experienced by patients receiving atezolizumab compared with patients in the placebo-chemotherapy arm who were not receiving any study treatment, as reflected in the proportion of patients reporting “somewhat,” “quite a bit,” or “very much” bother at each visit (Fig. 4).

## Discussion

There are few published studies of PROs for taxane and anthracycline chemotherapy regimens in early breast cancer and none, to our knowledge, for those receiving immunotherapy^8^. Although adverse events associated with immunotherapy have been reported in this setting by treating physicians^6,12^, patient-reported data on the effect of these adverse events on their treatment experience have been lacking to date. As treatment effects, rather than disease symptoms, largely define the patient experience in the curative setting of early breast cancer, the lack of PRO data for checkpoint inhibitor therapy constitutes an important evidence gap. Therefore, the current study provides important data in this treatment setting with results from a comprehensive assessment of treatment burden from the patients’ perspective including the experience of treatment-related symptoms and associated bother, as well as the impact of treatment on functioning, and patients’ HRQoL. The PRO data in IMpassion031 were collected frequently and systematically, and the completion rates of the PRO assessments were high. Overall, PRO data from this study showed very similar trends over the course of the study in the atezolizumab-chemotherapy and placebo-chemotherapy arms for mean values for physical and role function, as well as HRQoL. Each arm demonstrated a similar degree of clinically meaningful deterioration in each of these constructs during the neoadjuvant period, with observed trends in values stabilizing over the course of the adjuvant period.

As the clinically meaningful worsening in functioning and HRQoL in the current study was largely due to chemotherapy, which patients in both arms received, no differences were expected between the groups. Role function was a notable exception, remaining below the negative 10-point threshold of clinically meaningful decline during the adjuvant period in the atezolizumab arm, likely due in part to these patients continuing to receive treatment infusions in the clinic, which could impact their ability to work or conduct other daily activities. In contrast, due to the absence of study treatment infusions after surgery, patients in the placebo-chemotherapy arm likely did not experience the same reduction in role function. Additionally, fatigue is an adverse event known to be related to atezolizumab treatment. It occurs in an estimated 24.5% of patients, with 2.2% having Grade 3 or higher^13^. In our study, mean values for fatigue approached the threshold for clinically meaningful worsening in the adjuvant period for the atezolizumab arm and likely could also have contributed to reductions in role function among these patients.

The baseline functioning and HRQoL values recorded for the IMpassion031 population were higher than published reference values for a general population of patients with early breast cancer^6,14^, possibly reflecting the selection of more fit patients in this trial. Accordingly, the degree of deterioration from baseline over the course of neoadjuvant therapy seen here, namely a change from baseline values that far exceeded the 10-point threshold of clinically meaningful change, in some cases reaching as high as a 40-point decline, may differ from that seen in a general early TNBC population. Although reference values specific to an early-stage TNBC population are not available, a lower baseline value in those patients would not eliminate the expectation of a clinically meaningful decline in functioning and HRQoL that was observed in this trial. However, either a larger or smaller decline might be observed in the general population, and the speed and pattern of recovery may differ from this trial.

Mean absolute values and mean change from baseline values for treatment symptoms were comparable between arms during the neoadjuvant period, with no differences in clinically meaningful worsening observed between arms through cycle 5, and a similar proportion of patients in each arm reported the same level of overall bother with treatment side effects at each visit. Additionally, raw patient-reported symptom scores in each arm generally remained very low, reflecting a change from baseline of <1 category (i.e., from “not at all” to “a little”). Notably, during the adjuvant period, all patients in the atezolizumab-chemotherapy arm were due to receive atezolizumab maintenance therapy through cycle 16, whereas patients in the placebo-chemotherapy arm could receive adjuvant systemic treatment (according to the standard of care, potentially including capecitabine) only in the absence of a pCR. Therefore, patients in the atezolizumab arm would more likely experience treatment-related symptoms during the adjuvant phase because many placebo-arm patients were expected to have physician visits but no treatment. However, patient-reported treatment-related symptom values were generally stable and close to baseline values in both arms throughout the adjuvant period, and patients in the atezolizumab arm did not report more side effect bother through cycle 16. Furthermore, longitudinal analysis showed no differential treatment effect over time for role, physical function, or HRQoL. Therefore, as treatment burden may be most relevant during prolonged periods of therapy in otherwise asymptomatic patients, the data to date from the adjuvant period assessing functioning, HRQoL, treatment-related symptoms, and side effect bother to provide important support for the tolerability of this regimen.

In this population of patients with early-stage TNBC, atezolizumab was given as add-on treatment to chemotherapy in a largely asymptomatic, treatment-naive patient population. As expected, both the atezolizumab-chemotherapy and the placebo-chemotherapy groups experienced similar treatment-related decline due to the impact of chemotherapy. PRO measures stabilized after the chemotherapy was completed. The IMpassion031 PRO analysis indicated that several measures of treatment burden were comparable between atezolizumab chemotherapy and placebo chemotherapy over the course of patients’ treatment journeys. These findings demonstrate that the pCR improvement from adding atezolizumab to neoadjuvant chemotherapy with *nab*-paclitaxel followed by doxorubicin-cyclophosphamide did not impose additional treatment burden on patients during the neoadjuvant treatment period.

## Methods

### Study design

IMpassion031 (NCT03197935) is an ongoing global, randomized, double-blind, placebo-controlled trial. The study design and patient eligibility criteria, as well as the full protocol, have been published previously^6^; in brief, patients with cT2-T4d, cN0-cN3, M0 TNBC (with histology confirmed by central assessment), no prior systemic therapy for breast cancer, and an Eastern Cooperative Oncology Group performance status of 0 or 1 were randomized 1:1 to receive double-blind neoadjuvant treatment with atezolizumab or placebo combined with sequential chemotherapy with *nab*-paclitaxel followed by dose-dense doxorubicin plus cyclophosphamide. Any level of PD-L1 expression was permitted, provided expression status on tumor-infiltrating immune cells by central assessment was known; stratification was performed according to both PD-L1 status (<1% vs ≥1% of tumor area containing PD-L1–expressing immune cells as assessed by the VENTANA SP142 immunohistochemical assay) and American Joint Committee on Cancer breast cancer stage (II vs III).

Patients received *nab*-paclitaxel 125 mg/m^2^ intravenously (IV) once weekly for 12 weeks (cycles 1–3, 4 weeks/cycle) followed by doxorubicin 60 mg/m^2^ and cyclophosphamide 600 mg/m^2^ IV every 2 weeks for 8 weeks (cycles 4-5); atezolizumab 840 mg or placebo was given IV every 2 weeks throughout. Following surgery and pathologic evaluation, treatment assignment was unblinded to patients and study investigators, and patients in the atezolizumab arm received atezolizumab (1200 mg every 3 weeks) for cycles 6–16 (an additional 11 cycles of atezolizumab, 3 weeks/cycle) for a total of approximately 1 year of therapy; patients in the placebo arm had doctor visits on the same schedule without receiving study treatment infusions. In both study arms, patients could receive postoperative radiotherapy per local practice; patients not achieving pCR were managed according to standard guidelines, including adjuvant capecitabine or other systemic chemotherapies per treating investigator decision. This report focuses on the PRO results from the neoadjuvant period, while providing data from the adjuvant period to further contextualize the trends and findings of the neoadjuvant period.

The study was conducted in accordance with the guidelines for Good Clinical Practice and the Declaration of Helsinki. The study protocol was approved by institutional review boards of participating institutions, including the Taipei General Hospital Institutional Review Board and the Western Institutional Review Board. All patients provided written informed consent to participate.

### PRO assessments

Assessment of treatment burden included measures of treatment-related symptoms, side effect bother, and the impact of treatment on functioning and HRQoL (Supplementary Fig. 5). Prespecified secondary endpoints included mean and mean changes from baseline in physical function (items 1–5), role function (items 6–7), and GHS/HRQoL (items 29 and 30) as measured using the EORTC QLQ-C30^15,16^. Exploratory PRO endpoints included disease- and treatment-related symptoms measured using symptom scales of the EORTC QLQ-C30 (items 8–19), emotional and social functioning (items 21–27), and the proportion of patients reporting each response option at each assessment time point by treatment arm for a single item (GP5: “I am bothered by side effects of treatment”) from the FACT-G instrument^17^.

PRO data were collected frequently and systematically throughout the blinded neoadjuvant and unblinded adjuvant portions of this study. Patients completed the PRO instruments on paper at clinic visits before any other study assessments on day 1 (±3 days) of each cycle (starting at cycle 2 for FACT-G GP5), at the treatment discontinuation visit (≤30 days after the last dose of study treatments or after the last monitoring visit in the control arm), and during survival follow-up (every 3 months for the first year, every 6 months for years 2 and 3, and annually thereafter); there was a ±28-day completion window during follow-up. The PRO-evaluable population included all patients who completed a baseline assessment and at least 1 postbaseline assessment for the instrument or scale being analyzed.

The 30 items of the EORTC QLQ-C30 instrument comprise 5 aspects of patient functioning (physical, emotional, role, cognitive, and social); 8 symptom scales (fatigue, nausea and vomiting, pain, dyspnea, insomnia, appetite loss, constipation, and diarrhea); GHS/QoL, and financial difficulties, with a recall period of “the past week.” Each item in the function and symptom scales was scored on a 4-point Likert scale, with responses ranging from 1 (not at all) to 4 (very much); the GHS/QoL scale included 2 items, each measured on a 7-point scale from 1 (very poor) to 7 (excellent). Scoring of the EORTC QLQ-C30 items was conducted according to the EORTC QLQ-C30 Scoring Manual^18^; a raw score was calculated by estimating the average of items contributing to a scale, and was then standardized to a 0- to 100-point range using a linear transformation. Higher values on the function and GHS scales represent better functioning/HRQoL. Higher values on the symptom scales indicate worsening symptoms. The minimal clinically meaningful within-group change in EORTC QLQ-C30 values has been established as 10 points^19^.

The single-item GP5 from the physical well-being subscale of the FACT-G was used to document patients’ level of bother from side effects. Patients indicated how true the statement “I am bothered by side effects of treatment” had been for them during the previous 7 days on a 5-point scale (0, not at all; 1, a little bit; 2, somewhat; 3, quite a bit; and 4, very much). Item GP5 from version 4 of the FACT-G questionnaire was scored according to the Functional Assessment of Chronic Illness Therapy scoring manual^20^. For the FACT-G GP5 and each item/subscale of the EORTC QLQ-C30, summary statistics (mean with 95% CIs and SD, median, and range) were calculated for absolute values and mean changes from baseline at each time point for each arm. The EORTC QLQ-C30 physical and role function and GHS scales were also analyzed longitudinally using a mixed model for repeated measures by treatment arm across visits through survival follow-up, adjusted for covariates. The proportion of patients in each arm responding to each response option of item GP5 by time point was calculated and presented graphically in bar charts.

### Missing data

Missing data were assessed and reported by cycle. In the event of incomplete data, for all questionnaire subscales, if >50% of the constituent items were completed, a prorated score was computed consistent with the scoring manuals and validation papers. For subscales with <50% of the items completed, the subscale was considered missing. Patient-reported outcome completion, compliance rates, and reasons for missing data (i.e., measure not administered; respondent declined; other, please specify) were summarized at each time point by the treatment arm.

## Supplementary information

IMpassion031 Approved Protocol

Supplemental material

## Data Availability

For eligible studies, qualified researchers may request access to individual patient level clinical data through a data request platform. At the time of this writing this request platform is Vivli. https://vivli.org/ourmember/roche/. Further up-to-date details on Roche’s Global Policy on the Sharing of Clinical Information and how to request access to related clinical study documents see here: https://go.roche.com/data_sharing/). Anonymized records for individual patients across more than one data source external to Roche cannot, and should not, be linked due to a potential increase in risk of patient re-identification.
