# Supplementary material for: Patient-reported outcomes from a randomized trial of neoadjuvant atezolizumab-chemotherapy in early triple-negative breast cancer
Source: NPJ Breast Cancer. 2022 Sep 19;8:108. doi: 10.1038/s41523-022-00457-3 (PMC9485121; doi:10.1038/s41523-022-00457-3)
Supplement: Supplementary file 1 — IMpassion031 Approved Protocol [file 41523_2022_457_MOESM1_ESM.pdf]

PLEASE READ CAREFULLY:

By opening the attached document you agree to the following terms of use:

- You may not use this document or the information contained herein to a regulatory authority in connection with an application for a marketing authorization or any other regulatory submission without the express written consent of Roche. Please contact global.data_sharing@roche.com

- You may not use this document or the information contained herein to identify clinical trial patients.

- You may not copy, reproduce, or make this document available in any manner that would permit a third-party to review or use the document without first agreeing to these terms of use.

PROTOCOL

**TITLE:**	**A PHASE III RANDOMIZED STUDY TO INVESTIGATE THE EFFICACY AND SAFETY OF ATEZOLIZUMAB (ANTI–PD-L1 ANTIBODY) IN COMBINATION WITH NEOADJUVANT ANTHRACYCLINE/NAB-PACLITAXEL-BASED CHEMOTHERAPY COMPARED WITH PLACEBO AND CHEMOTHERAPY IN PATIENTS WITH PRIMARY INVASIVE TRIPLE-NEGATIVE BREAST CANCER**
**PROTOCOL NUMBER:**	WO39392
**VERSION NUMBER:**	7
**EUDRACT NUMBER:**	2016-004734-22
**IND NUMBER:**	123277
**NCT NUMBER:**	NCT03197935
**TEST PRODUCT:**	Atezolizumab (RO5541267)
**MEDICAL MONITOR:**	[redacted], *M.D.*
**SPONSOR:**	F. Hoffmann-La Roche Ltd
**APPROVAL DATE:**	See electronic date stamp below.

PROTOCOL AMENDMENT APPROVAL

**Date and Time (UTC)**	**Title**	**Approver's Name**
11-Feb-2020 14:53:54	Company Signatory	[redacted]

CONFIDENTIAL

This clinical study is being sponsored globally by F. Hoffmann-La Roche Ltd of Basel, Switzerland. However, it may be implemented in individual countries by Roche's local affiliates, including Genentech, Inc. in the United States. The information contained in this document, especially any unpublished data, is the property of F. Hoffmann-La Roche Ltd (or under its control) and therefore is provided to you in confidence as an investigator, potential investigator, or consultant, for review by you, your staff, and an applicable Ethics Committee or Institutional Review Board. It is understood that this information will not be disclosed to others without written authorization from Roche except to the extent necessary to obtain informed consent from persons to whom the drug may be administered.

"Use of this document is governed by the terms of use on the first page of this document."

PROTOCOL HISTORY

Protocol		Associated Country-Specific Protocols		
**Version**	**Date Final**	**Country**	**Version**	**Date Final**
6	7 June 2019	VHP adopted Version 6.		
5	10 October 2018	VHP	5	10 December 2018
4	11 May 2018	VHP adopted Version 4.		
3	4 May 2017	VHP adopted Version 3.		
		VHP (only)	2	17 April 2017
1	14 December 2016	VHP adopted Version 1.		

"Use of this document is governed by the terms of use on the first page of this document."

PROTOCOL AMENDMENT, VERSION 7:
RATIONALE

Protocol WO39392 has primarily been amended to update risks and management guidelines for atezolizumab to align with the latest Atezolizumab Investigator's Brochure (Version 15). Changes to the protocol, along with a rationale for each change, are summarized below:

- To align with the Atezolizumab Investigator's Brochure, Version 15, "immune-related" has been changed to "immune-mediated" when describing events associated with atezolizumab (Sections 1.4, 4.4.2, 5.1.1, and Appendix 13).
- To address a request by the [redacted], systemic immune activation has been replaced by hemophagocytic lymphohistiocytosis and macrophage activation syndrome in the list of potential risks for atezolizumab (Section 5.1.1) and the management guidelines for systemic immune activation have been replaced with management guidelines for hemophagocytic lymphohistiocytosis and macrophage activation syndrome (Appendix 13). In addition, systemic immune activation has been removed from the list of adverse events of special interest (Section 5.2.3).
- To align with the nab-paclitaxel (Abraxane®) prescribing information (Summary of Product Characteristics), the risk of tumor lysis syndrome has been included (Section 5.1.2).
- The Medical Monitor name and associated contact information has been updated (Section 5.4.1).
- A new section has been added to describe the implementation of a system to manage the quality of the study (Section 9.3).
- The Appendix 9 (Anaphylaxis Precautions) has been modified to remove the requirement for use of a tourniquet. The application of a tourniquet is no longer recommended due to the limited therapeutic benefit and risk of losing time for more important measures (Ring J, Beyer K, Biedermann T, et al. Allergo J Int. 2014;23:96–112).
- To address a request by the [redacted], the atezolizumab adverse event management guidelines have been revised to add laboratory (e.g., B-type natriuretic peptide) and cardiac imaging abnormalities as signs or symptoms that are suggestive of myocarditis (Appendix 13).
- The management guidelines for infusion-related reactions associated with atezolizumab have been updated to include guidelines for cytokine-release syndrome (CRS) to align with the definition, grading, and management of CRS reflected in a recent publication (Lee et al. 2019) (Appendix 13).

Additional minor changes have been made to improve clarity and consistency. Substantive new information appears in italics. This amendment represents cumulative changes to the original protocol.

"Use of this document is governed by the terms of use on the first page of this document."

TABLE OF CONTENTS

PROTOCOL AMENDMENT ACCEPTANCE FORM 11

PROTOCOL SYNOPSIS 12

1. BACKGROUND 26

1.1 Background on Breast Cancer 26

1.2 Treatment for Early Triple-Negative Breast Cancer 26

1.3 Background on Atezolizumab 27

1.4 Study Rationale and Benefit-Risk Assessment 28

2. OBJECTIVES AND ENDPOINTS 30

3. STUDY DESIGN 33

3.1 Description of the Study 33

3.1.1 Overview of Study Design 33

3.1.2 Independent Data Monitoring Committee 38

3.2 End of Study and Length of Study 39

3.3 Rationale for Study Design 39

3.3.1 Rationale for Atezolizumab Dose and Schedule 39

3.3.2 Rationale for Patient Population and Analysis Groups 40

3.3.3 Rationale for Comparator of Nab-Paclitaxel Followed by Doxorubicin and Cyclophosphamide 40

3.3.4 Rationale for Pathologic Complete Response as Primary Endpoint 43

3.3.5 Rationale for Biomarker Assessments 43

3.3.6 Rationale for Patient-Reported Outcome Assessments 45

4. MATERIALS AND METHODS 46

4.1 Patients 46

4.1.1 Inclusion Criteria 46

4.1.2 Exclusion Criteria 49

4.2 Method of Treatment Assignment and Blinding 53

4.3 Study Treatment 55

"Use of this document is governed by the terms of use on the first page of this document."

4.3.1 Formulation, Packaging, and Handling 55
4.3.1.1 Atezolizumab and Placebo 55
4.3.1.2 Background Treatment 55
4.3.2 Dosage, Administration, and Compliance 55
4.3.2.1 Atezolizumab or Placebo 55
4.3.2.2 Background Treatment 57
4.3.3 Investigational Medicinal Product Accountability 58
4.3.4 Post-Trial Access to Atezolizumab 59
4.4 Concomitant Therapy and Additional Restrictions 59
4.4.1 Permitted Therapy 59
4.4.2 Cautionary Therapy 60
4.4.3 Potential Drug Interactions with Chemotherapy 60
4.4.4 Prohibited Therapy 61
4.5 Study Assessments 62
4.5.1 Informed Consent Forms and Screening Log 62
4.5.2 Medical History, Concomitant Medication, and Demographic Data 63
4.5.3 Physical Examinations 63
4.5.4 Vital Signs 63
4.5.5 Locoregional Tumor Status 64
4.5.6 Distant Sites Tumor Assessment 65
4.5.7 Disease Follow-Up and Confirmation of Disease Progression or Recurrence 65
4.5.8 Surgical Specimen Pathology 69
4.5.9 Laboratory, Biomarker, and Other Biological Samples 69
4.5.10 Electrocardiograms 72
4.5.11 Echocardiograms or Multiple-Gated Acquisition Scans 72
4.5.12 Pulmonary Function Tests (Spirometry) 73
4.5.13 Patient-Reported Outcomes 73
4.5.13.1 EORTC QLQ-C30 73
4.5.13.2 FACT-G Single Item GP5 74
4.5.13.3 EQ-5D-5L 74

"Use of this document is governed by the terms of use on the first page of this document."

4.5.14 Mandatory Samples for Whole Genome Sequencing 74
4.5.15 Optional Samples for Research Biosample Repository 75
4.5.15.1 Overview of the Research Biosample Repository 75
4.5.15.2 Approval by the Institutional Review Board or Ethics Committee 76
4.5.15.3 Sample Collection 76
4.5.15.4 Confidentiality 77
4.5.15.5 Consent to Participate in the Research Biosample Repository 77
4.5.15.6 Withdrawal from the Research Biosample Repository 78
4.5.15.7 Monitoring and Oversight 78
4.6 Treatment, Patient, Study, and Site Discontinuation 78
4.6.1 Study Treatment Discontinuation 78
4.6.2 Patient Discontinuation from Study 79
4.6.3 Study Discontinuation 79
4.6.4 Site Discontinuation 80

5. ASSESSMENT OF SAFETY 80
5.1 Safety Plan 80
5.1.1 Risks Associated with Atezolizumab 80
5.1.2 Risks Associated with Nab-Paclitaxel 81
5.1.3 Risks Associated with Doxorubicin 81
5.1.3.1 Cardiomyopathy 81
5.1.4 Risks Associated with Cyclophosphamide 82
5.1.5 Management of Patients Who Experience Specific Adverse Events 82
5.2 Safety Parameters and Definitions 84
5.2.1 Adverse Events 85
5.2.2 Serious Adverse Events (Immediately Reportable to the Sponsor) 85
5.2.3 Adverse Events of Special Interest (Immediately Reportable to the Sponsor) 86

"Use of this document is governed by the terms of use on the first page of this document."

5.2.4 Selected Adverse Events 87
5.2.4.1 Cardiac, General 87
5.2.4.2 Asymptomatic Left Ventricular Systolic Dysfunction 87
5.3 Methods and Timing for Capturing and Assessing Safety Parameters 88
5.3.1 Adverse Event Reporting Period 88
5.3.2 Eliciting Adverse Event Information 88
5.3.3 Assessment of Severity of Adverse Events 88
5.3.4 Assessment of Causality of Adverse Events 89
5.3.5 Procedures for Recording Adverse Events 90
5.3.5.1 Infusion-Related Reactions 90
5.3.5.2 Diagnosis Versus Signs and Symptoms 90
5.3.5.3 Adverse Events That Are Secondary to Other Events 91
5.3.5.4 Persistent or Recurrent Adverse Events 91
5.3.5.5 Abnormal Laboratory Values 92
5.3.5.6 Abnormal Vital Sign Values 92
5.3.5.7 Abnormal Liver Function Tests 93
5.3.5.8 Deaths 93
5.3.5.9 Preexisting Medical Conditions 94
5.3.5.10 Lack of Efficacy or Worsening of TNBC 94
5.3.5.11 Hospitalization or Prolonged Hospitalization 94
5.3.5.12 Cases of Accidental Overdose or Medication Error 95
5.3.5.13 Patient-Reported Outcome Data 96
5.4 Immediate Reporting Requirements from Investigator to Sponsor 96
5.4.1 Emergency Medical Contacts 97
5.4.2 Reporting Requirements for Serious Adverse Events and Adverse Events of Special Interest 97
5.4.2.1 Events That Occur Prior to Study Treatment Initiation 97
5.4.2.2 Events That Occur After Study Treatment Initiation 97

"Use of this document is governed by the terms of use on the first page of this document."

5.4.3 Reporting Requirements for Pregnancies 98
5.4.3.1 Pregnancies in Female Patients 98
5.4.3.2 Pregnancies in Female Partners of Male Patients 98
5.4.3.3 Abortions 99
5.4.3.4 Congenital Anomalies/Birth Defects 99
5.5 Follow-Up of Patients After Adverse Events 99
5.5.1 Investigator Follow-Up 99
5.5.2 Sponsor Follow-Up 100
5.6 Adverse Events That Occur After the Adverse Event Reporting Period 100
5.7 Expedited Reporting to Health Authorities, Investigators, Institutional Review Boards, and Ethics Committees 100

6. STATISTICAL CONSIDERATIONS AND ANALYSIS PLAN 101
6.1 Determination of Sample Size 101
6.1.1 Adaptive Design 101
6.1.2 Type I Error Control 102
6.2 Summaries of Conduct of Study 102
6.3 Summaries of Treatment Group Comparability 103
6.4 Efficacy Analyses 103
6.4.1 Primary Efficacy Endpoint: Pathologic Complete Response 103
6.4.2 Secondary Efficacy Endpoints 103
6.4.2.1 Event-Free Survival 104
6.4.2.2 Disease-Free Survival 104
6.4.2.3 Overall Survival 104
6.4.2.4 Patient-Reported Outcomes of Role and Physical Function and GHS/HRQoL—EORTC Data.......................... 105
6.4.3 Exploratory Efficacy Endpoints 105
6.4.3.1 Patient-Reported Outcomes of Disease/Treatment-Related Symptoms, Emotional and Social Function—EORTC Data.................... 105
6.4.3.2 Patient-Reported Outcome of Treatment Bother—FACT-G, GP5 Single Item Data............................ 105
6.4.3.3 Health Economic EQ-5D-5L Data 106

"Use of this document is governed by the terms of use on the first page of this document."

6.5 Safety Analyses 106
6.6 Pharmacokinetic Analyses 106
6.7 Immunogenicity Analyses 106
6.8 Biomarker Analyses 107
6.9 Interim Analyses 107
6.9.1 Planned Interim Analysis 107
6.9.2 Optional Interim Analysis 108

7. DATA COLLECTION AND MANAGEMENT 108
7.1 Data Quality Assurance 108
7.2 Electronic Case Report Forms 108
7.3 Source Data Documentation 109
7.4 Use of Computerized Systems 109
7.5 Retention of Records 110

8. ETHICAL CONSIDERATIONS 110
8.1 Compliance with Laws and Regulations 110
8.2 Informed Consent 110
8.3 Institutional Review Board or Ethics Committee 111
8.4 Confidentiality 112
8.5 Financial Disclosure 112

9. STUDY DOCUMENTATION, MONITORING, AND ADMINISTRATION 113
9.1 Study Documentation 113
9.2 Protocol Deviations 113
9.3 Management of Study Control 113
9.4 Site Inspections 113
9.5 Administrative Structure 114
9.6 Publication of Data and Protection of Trade Secrets 114
9.7 Protocol Amendments 115

10. REFERENCES 116

"Use of this document is governed by the terms of use on the first page of this document."

LIST OF TABLES

Table 1 Objectives and Corresponding Endpoints 30
Table 2 Definitions of and Procedures for Confirming Disease Recurrence, Death, and Other Noteworthy Events on Follow-Up 67
Table 3 Dose Levels for Nab-Paclitaxel (Weekly Dose)........................... 83
Table 4 Guidelines for Management of Patients Who Experience Nab-Paclitaxel–Specific Adverse Events 83
Table 5 Adverse Event Severity Grading Scale for Events Not Specifically Listed in NCI CTCAE 89
Table 6 Causal Attribution Guidance 90

LIST OF FIGURES

Figure 1 Study Schema.. 35
Figure 2 Documenting Treatment Burden in Early Breast Cancer Patients 46

LIST OF APPENDICES

Appendix 1 Schedule of Activities (for Both Stage 1 and Stage 2) 123
Appendix 2 Schedule of Pharmacokinetic, Immunogenicity, and Fluid Biomarker Samples.. 131
Appendix 3 Tumor Tissue Requirements... 133
Appendix 4 Cardiac Safety Cohort Cardiac Assessments 134
Appendix 5 European Organisation for Research and Treatment of Cancer Quality of Life Questionnaire Core 30........................... 136
Appendix 6 FACT-G Single Item GP5.. 138
Appendix 7 EuroQol 5-Dimension, 5-Level Questionnaire: EQ-5D-5L........ 139
Appendix 8 Preexisting Autoimmune Diseases 142
Appendix 9 Anaphylaxis Precautions... 143
Appendix 10 Eastern Cooperative Oncology Group Scale of Performance Status.. 144
Appendix 11 Guideline on the Management of Axillary Lymph Nodes for Study WO39392 (Impassion031) 145
Appendix 12 Criteria for Baseline Nodal Staging and Implications on Post-Neoadjuvant Axillary Lymph Node Management for Study WO39392 (IMpassion031) 146
Appendix 13 Risks Associated with Atezolizumab and Guidelines for Management of Adverse Events Associated with Atezolizumab 147
Appendix 14 Statistical Detail of the Adaptive Design for Study WO39392 (IMpassion031) 174
Appendix 15 Statistical Performance of the Adaptive Design 177

"Use of this document is governed by the terms of use on the first page of this document."

PROTOCOL AMENDMENT ACCEPTANCE FORM

**TITLE:**	**A PHASE III RANDOMIZED STUDY TO INVESTIGATE THE EFFICACY AND SAFETY OF ATEZOLIZUMAB (ANTI–PD-L1 ANTIBODY) IN COMBINATION WITH NEOADJUVANT ANTHRACYCLINE/NAB-PACLITAXEL-BASED CHEMOTHERAPY COMPARED WITH PLACEBO AND CHEMOTHERAPY IN PATIENTS WITH PRIMARY INVASIVE TRIPLE-NEGATIVE BREAST CANCER**
**PROTOCOL NUMBER:**	WO39392
**VERSION NUMBER:**	7
**EUDRACT NUMBER:**	2016-004734-22
**IND NUMBER:**	123277
**NCT NUMBER:**	NCT03197935
**TEST PRODUCT:**	Atezolizumab (RO5541267)
**MEDICAL MONITOR:**	[redacted], *M.D.*
**SPONSOR:**	F. Hoffmann-La Roche Ltd

I agree to conduct the study in accordance with the current protocol.

____

Principal Investigator's Name (print)

____ __________________

Principal Investigator's Signature Date

Please retain the signed original of this form for your study files. Please return a copy of this form to the Sponsors or their designee. Contact details will be provided to the investigator prior to study start.

"Use of this document is governed by the terms of use on the first page of this document."

PROTOCOL SYNOPSIS

**TITLE:**	**A PHASE III RANDOMIZED STUDY TO INVESTIGATE THE EFFICACY AND SAFETY OF ATEZOLIZUMAB (ANTI–PD-L1 ANTIBODY) IN COMBINATION WITH NEOADJUVANT ANTHRACYCLINE/NAB-PACLITAXEL-BASED CHEMOTHERAPY COMPARED WITH PLACEBO AND CHEMOTHERAPY IN PATIENTS WITH PRIMARY INVASIVE TRIPLE-NEGATIVE BREAST CANCER**
**PROTOCOL NUMBER:**	WO39392
**VERSION NUMBER:**	7
**EUDRACT NUMBER:**	2016-004734-22
**IND NUMBER:**	123277
**NCT NUMBER:**	NCT03197935
**TEST PRODUCT:**	Atezolizumab (RO5541267)
**PHASE:**	III
**INDICATION:**	Triple-negative breast cancer
**SPONSOR:**	F. Hoffmann-La Roche Ltd

Objectives and Endpoints

Study WO39392 (also known as IMpassion031) will evaluate the efficacy, safety, and pharmacokinetics of neoadjuvant nab-paclitaxel and atezolizumab followed by doxorubicin and cyclophosphamide with atezolizumab (referred to as atezolizumab + nab-pac–AC) or neoadjuvant nab-paclitaxel and placebo followed by doxorubicin and cyclophosphamide with placebo (referred to as placebo + nab-pac–AC) in patients with T2–4d triple-negative breast cancer (TNBC). Specific objectives and corresponding endpoints for the study are as follows:

Objectives	Corresponding Endpoints
**Primary Efficacy Objective:**	
• To evaluate the efficacy of atezolizumab + nab-pac–AC compared with placebo + nab-pac–AC in the neoadjuvant setting	• pCR defined as eradication of tumor from both breast and lymph nodes (ypT0/is ypN0) in the following:<br>– All patients (ITT population)<br>– Subpopulation of patients with PD-L1–positive tumor status (IC1/2/3)

"Use of this document is governed by the terms of use on the first page of this document."

Objectives	Corresponding Endpoints
**Secondary Efficacy Objectives:**	
• To evaluate the efficacy of atezolizumab + nab-pac–AC compared with placebo + nab-pac–AC in the neoadjuvant setting	• EFS defined as the time from randomization until documented disease recurrence, progression, or death from any cause in all patients (ITT population) and in the subpopulation with PD-L1–positive tumor status<br>• DFS defined as the time from surgery until documented disease recurrence or death from any cause in all patients (ITT population) who undergo surgery and in the subpopulation of patients with PD-1–positive tumor status who undergo surgery<br>• OS defined as the time from randomization to the date of death from any cause in all patients (ITT population) and in the subpopulation with PD-L1–positive tumor status
• To evaluate PROs of function and HRQoL associated with atezolizumab + nab-pac–AC compared with placebo + nab-pac–AC, measured by the functional and HRQoL scales of the EORTC QLQ-C30	• Mean and mean changes from baseline score in function (role, physical) and GHS/HRQoL by cycle and between treatment arms as assessed by the functional and HRQoL scales of the EORTC QLQ-C30
**Exploratory Efficacy Objectives:**	
• To evaluate the efficacy of atezolizumab + nab-pac–AC compared with placebo + nab-pac–AC in the neoadjuvant setting	• Proportion of patients undergoing breast-conserving surgery<br>• RCB index<br>• Correlation of RCB with other clinical endpoints (if deemed appropriate)
• To evaluate PROs of disease/treatment-related symptoms associated with atezolizumab + nab-pac–AC compared with placebo + nab-pac–AC, as measured by the EORTC QLQ-C30	• Mean and mean changes from baseline score in disease/treatment-related symptoms by cycle and between treatment arms as assessed by all symptom items/scales of the EORTC QLQ-C30
• To evaluate any treatment burden patients may experience associated with the addition of atezolizumab to nab-pac–AC compared with placebo + nab-pac–AC, as measured by a single item (GP5: "I am bothered by side effects of treatment") from the physical well-being subscale of the FACT-G Quality of Life instrument	• Proportion of patients reporting each response option at each assessment timepoint by treatment arm for item GP5 from the FACT-G
• To evaluate and compare between treatment arms patient's health utility as measured by the EQ-5D-5L questionnaire to generate utility scores for use in economic models	• Utility scores of the EQ-5D-5L questionnaire
**Safety Objective:**	
• To evaluate the safety and tolerability of atezolizumab + nab-pac–AC compared with placebo + nab-pac–AC	• Occurrence and severity of adverse events as defined by NCI CTCAE v4.0

"Use of this document is governed by the terms of use on the first page of this document."

Objectives	Corresponding Endpoints
**Pharmacokinetic Objectives:**	
• To characterize the pharmacokinetics of atezolizumab when administered in combination with nab-pac–AC chemotherapy	• Serum concentration of atezolizumab at specified timepoints
**Immunogenicity Objective:**	
• To evaluate the immune response to atezolizumab	• Incidence of ADAs during the study and the prevalence of ADAs at baseline
**Exploratory Immunogenicity Objective:**	
• To evaluate potential effects of ADAs	• Relationship between ADA status and efficacy, safety, and PK endpoints
**Exploratory Biomarker Objective:**	
• To assess predictive, prognostic, and pharmacodynamic exploratory biomarkers in archival and/or fresh tumor tissue and blood and their association with efficacy endpoints including but not limited to pCR	• Relationship between PD-L1 IHC and efficacy endpoints other than pCR<br>• Relationship between tumor derived RNA-based immune gene signatures and efficacy endpoints, including but not limited to pCR<br>• Relationship between tumor-based tumor infiltrating lymphocytes and/or CD8 IHC and efficacy endpoints, including but not limited to pCR<br>• Pharmacodynamic changes in cancer-related immune, stroma and tumor immune biology parameters on-treatment by, but not limited to, gene expression and IHC in baseline, on-treatment, and residual disease tumor tissues<br>• Relationship of baseline and on-treatment plasma biomarkers and efficacy endpoints, including but not limited to pCR

ADA = anti-drug antibody; atezolizumab + nab-pac–AC = nab-paclitaxel and atezolizumab followed by doxorubicin and cyclophosphamide with atezolizumab; DFS = disease-free survival; EFS = event-free survival; EORTC = European Organisation for Research and Treatment of Cancer; EQ-5D-5L = EuroQoL 5-Dimension, 5-Level; FACT–G = Functional Assessment of Cancer Therapy–General; GHS = global health status; HRQoL = health-related quality of life; IC = tumor-infiltrating immune cell; IHC = immunohistochemistry; ITT = intent to treat; NCI CTCAE = National Cancer Institute Common Terminology Criteria for Adverse Events; OS = overall survival; pCR = pathologic complete response; PD–L1 = programmed death–ligand 1; PK = pharmacokinetics; placebo + nab-pac–AC = neoadjuvant nab-paclitaxel and placebo followed by doxorubicin and cyclophosphamide with placebo; PRO = patient-reported outcome; QLQ-C30 = Quality of Life Questionnaire Core 30; RCB = residual cancer burden.

<u>Study Design</u>

Description of Study

This is a global Phase III, double-blind, randomized, placebo-controlled study designed to evaluate the efficacy and safety of neoadjuvant treatment with atezolizumab + nab-pac-AC, or placebo + nab-pac–AC in patients eligible for surgery with initial clinically assessed T2-4d TNBC.

Female and male patients aged ≥ 18 years with an Eastern Cooperative Oncology Group (ECOG) performance status of 0 or 1 who have histologically confirmed invasive TNBC with a primary tumor size of > 2 cm are eligible.

"Use of this document is governed by the terms of use on the first page of this document."

Human epidermal growth factor receptor 2 (HER2) and estrogen/progesterone receptor (ER/PR) status will be used to define TNBC. HER2 negativity will be defined by central laboratory assessment using in situ hybridization (ISH) or immunohistochemistry (IHC) assays per American Society of Clinical Oncology/College of American Pathologists (ASCO/CAP) criteria, and ER/PgR negativity will be defined by using IHC per ASCO/CAP criteria. Central laboratory assessment will occur prior to randomization. Patients whose tumors are not confirmed to be triple negative will not be eligible. Patients whose tumor tissue is not evaluable for PD-L1 will not be eligible.

Patients who do not initially meet all eligibility criteria, other than TNBC status, may be rescreened once.

This study has an adaptive design consisting of two stages. Stage 1 is the randomization and treatment of approximately 204 patients. At the end of Stage 1, an interim analysis for efficacy will be done by an iDMC. Then, a recommendation will be made to either continue the study unchanged or to expand the patient population (Stage 2; approximately 120 additional patients). If the recommendation is to expand the patient population into Stage 2, the Sponsor will remain blinded to the results of the interim analysis performed at the end of Stage 1.

Patients who have consented and are eligible will be randomized in a 1:1 ratio to either of the following treatment groups:

- **Arm A**: atezolizumab (840 mg) administered via intravenous (IV) infusion Q2W in combination with nab-paclitaxel (125 mg/m^2) administered via IV infusion QW for 12 weeks followed by atezolizumab (840 mg) administered Q2W in combination with doxorubicin (60 mg/m^2) + cyclophosphamide (600 mg/m^2) administered Q2W via IV infusions with filgrastim/pegfilgrastim support for 4 cycles. Patients randomized to the atezolizumab arm will continue to receive unblinded atezolizumab post-surgery at a fixed dose of 1200 mg by IV infusion every 3 weeks (Q3W) for 11 cycles, for a total of approximately 12 months of atezolizumab therapy.
- **Arm B**: placebo administered Q2W via IV infusion in combination with nab-paclitaxel (125 mg/m^2) administered QW via IV infusion for 12 weeks followed by placebo administered Q2W in combination with doxorubicin (60 mg/m^2) + cyclophosphamide (600 mg/m^2) administered Q2W via IV infusions with filgrastim/pegfilgrastim support for 4 cycles. Patients randomized to the placebo arm will be unblinded post-surgery and will continue to be followed.

For patients in Stage 1 of the study, randomization will be stratified by the following factors:

- American Joint Committee on Cancer (AJCC) stage at diagnosis (II vs. III; see below for evaluation and classification of lymph nodes)
- Tumor PD-L1 status (tumor-infiltrating immune cell [IC] IC0 [<1% PD-L1 expressing IC per tumor area] vs. IC1/2/3 [≥1% PD-L1 expressing IC per tumor area])

Depending on the iDMC recommendation, Stage 2 could follow either an all-comer or PD-L1 enrichment design. If an all-comer design is recommended, the patients enrolled in Stage 2 will be stratified the same as patients enrolled in Stage 1. If an enrichment design is recommended, only PD-L1–positive patients will be enrolled, and the AJCC stage at diagnosis will be used as the sole randomization stratification factor.

Patients who discontinue neoadjuvant therapy early as a result of disease progression must be discontinued from all study treatment, will be managed as per local practice, and will be followed for survival only. Patients who discontinue prematurely from the study will not be replaced.

Any patient who receives non-protocol therapy prior to surgery will be discontinued from study treatment and will be managed as per local practice; these patients will remain on study for survival follow-up.

The primary efficacy endpoint (pathologic complete response [pCR]; ypT0/is ypN0) will be established via local review following completion of neoadjuvant therapy and surgery. Pathologists who review study specimens must utilize the evaluations and assessments outlined in the Pathology Manual. Investigator/individual patient unblinding will occur after pCR assessment. Surgery should be performed at least 14 days after the last dose of neoadjuvant

"Use of this document is governed by the terms of use on the first page of this document."

therapy but no later than 6 weeks after the last infusion. Platelet counts should be checked prior to surgery and should be $\geq$75,000 cells/μL.

Patients with clinically positive axillary nodes by physical examination or by any radiographic imaging at baseline should undergo fine-needle aspiration or core-needle biopsy prior to randomization followed by axillary lymph node dissection (ALND) at time of definitive surgery. The results of the baseline fine-needle aspiration or core-needle biopsy will determine the nodal staging, so that patients with a positive biopsy result should be staged as lymph-node-positive (N1-N3c) whereas patients with a negative or equivocal biopsy result should be staged as lymph-node-negative (N0) regardless of any other clinical measurements.

Baseline fine-needle aspiration (FNA) or core-needle biopsy is not mandatory for clinically/radiologically enlarged/suspicious internal mammary, subpectoral, infraclavicular, or supraclavicular lymph nodes. Investigators are advised to follow local practice guidelines regarding assessment of internal mammary, subpectoral, infraclavicular, or supraclavicular lymph nodes. Management of internal mammary, subpectoral, infraclavicular, or supraclavicular lymph nodes at the time of definitive surgery will be based on local practice guidelines. Patients with baseline axillary lymph nodes involvement who may also have clinically/radiologically enlarged internal mammary, subpectoral, infraclavicular, or supraclavicular lymph nodes must still undergo ALND as described earlier.

For patients with baseline clinically/radiologically enlarged/suspicious internal mammary, subpectoral, infraclavicular, or supraclavicular lymph nodes, pCR may be reported only if all the following conditions are met:

1. Presurgical imaging assessment of internal mammary, subpectoral, infraclavicular, or supraclavicular lymph nodes does not show any enlarged/suspicious lymph node(s) after neoadjuvant study treatment.

AND

2. Pathological evaluation of the breast and all resected lymph node(s) shows no residual invasive disease (i.e., pCR; ypT0/is ypN0).

Postoperative radiotherapy may be administered based on local practice guidelines and after approval from the Medical Monitor has been obtained.

In patients with clinically or fine-needle biopsy (FNA)/core needle biopsy-proven negative axillary nodes at baseline, axillary surgical management after completion of neoadjuvant therapy should include sentinel lymph node biopsy (SLNB) or ALND. If SLNB is conducted, it is strongly recommended that more than one lymph node (two to three minimum) be removed and all patients with positive macrometastases in sentinel nodes should undergo ALND regardless of the number of positive nodes. All patients with T4 tumors should undergo ALND or current standard of care as described in international or national guidelines.

Postoperative patient management for those in either treatment arm should include radiotherapy as clinically indicated, and management of patients who do not achieve a pCR should follow current standard-of-care guidelines. For those randomized to receive atezolizumab, patients may receive this therapy simultaneously with atezolizumab.

For those randomized to receive atezolizumab, the first dose of postoperative atezolizumab should be administered within 45 days of surgery.

Efficacy, safety, laboratory measurements, patient-reported outcomes (PROs), and pharmacokinetics will be assessed throughout the study. The first 26 patients enrolled (approximately 13 patients in the control arm and approximately 13 patients in the atezolizumab containing arm) will undergo additional cardiac safety monitoring as part of a cardiac safety cohort. Following completion of study treatment and surgery, all patients will continue to be followed for efficacy, safety, and PRO objectives until the end of the study. No interim efficacy analyses for early stopping are planned.

Safety assessments will include the occurrence and severity of adverse events and laboratory abnormalities graded per National Cancer Institute Common Terminology Criteria for Adverse Events (NCI CTCAE) Version 4.0. Laboratory safety assessments will include the regular monitoring of hematology and blood chemistry. Serum samples will be collected to monitor chemotherapy and atezolizumab pharmacokinetics and to detect the presence of antibodies to

"Use of this document is governed by the terms of use on the first page of this document."

atezolizumab. Patient samples, including tumor tissues, as well as plasma and blood, will be collected for exploratory biomarker assessments.

An iDMC will evaluate the primary efficacy endpoint of pCR in the intent-to-treat (ITT) population (defined as all randomized patients) and in the PD-L1–positive subpopulation based on an interim analysis of efficacy data from the Stage 1 patients (approximately 204 enrolled patients). The iDMC will make a recommendation either to continue the study unchanged or to enroll an additional 120 patients (Stage 2). The decision rules to be applied at the interim analysis will be clearly expressed to the iDMC and documented in the iDMC charter so that the study can be conducted with the Sponsor remaining completely blinded to all results at this stage.

The iDMC will evaluate safety data and study conduct on a regular basis during the study until the primary analysis of pCR, which is performed at Stage 1 (approximately 204 patients) or Stage 2 (approximately 324 patients), if the iDMC recommends extending the target population. After which, iDMC review of the study data will be discontinued.

Sponsor affiliates will be excluded from iDMC membership. The iDMC will follow a charter that outlines the iDMC roles and responsibilities.

Number of Patients

Approximately 204 patients will be enrolled in this study during Stage 1 and approximately 120 additional patients will be enrolled if the iDMC recommends to expand to Stage 2. Enrollment will take place at approximately 75 global sites.

Target Population

Inclusion Criteria

Patients must meet the following criteria for study entry:

- Signed Informed Consent Form
- Ability to comply with protocol, in the investigator's judgment
- Women or men aged ≥ 18 years
- ECOG performance status of 0 or 1
- Histologically documented TNBC (negative HER2, ER, and PgR status); HER2 negativity will be defined by central laboratory assessment using ISH or IHC assays per ASCO/CAP criteria and ER/PgR negativity will be defined by central laboratory assessment using IHC per ASCO/CAP criteria. Central laboratory assessment will occur prior to randomization.

  Patients with multifocal tumors (more than one tumor confined to the same quadrant as the primary tumor) are eligible provided all discrete lesions are sampled and centrally confirmed as TNBC.
- Confirmed tumor PD-L1 evaluation as documented through central testing of a representative tumor tissue specimen

  In Stage 2, if the iDMC recommendation is to expand to a PD-L1–positive population, only patients with confirmed tumor PD-L1 positive (IC1/2/3) will be considered eligible.
- Primary breast tumor size of > 2 cm by at least one radiographic or clinical measurement
- Stage at presentation: cT2–cT4, cN0–cN3, cM0
- Patient agreement to undergo appropriate surgical management including axillary lymph node surgery and partial or total mastectomy after completion of neoadjuvant treatment
- Baseline LVEF ≥ 53% measured by echocardiogram (ECHO) or multiple-gated acquisition (MUGA) scans
- Adequate hematologic and end-organ function, as defined by the following laboratory results obtained within 14 days prior to the first study treatment:
  - ANC ≥ 1500 cells/μL (without granulocyte colony–stimulating factor [G-CSF] support within 2 weeks prior to Cycle 1, Day 1)
  - Lymphocyte count ≥ 500 cells/μL
  - Platelet count ≥ 100,000 cells/μL (without transfusion within 2 weeks prior to Cycle 1, Day 1)
  - Hemoglobin ≥ 9.0 g/dL

"Use of this document is governed by the terms of use on the first page of this document."

- AST, ALT, and alkaline phosphatase ≤ 2.5 × the upper limit of normal (ULN)
- Serum bilirubin ≤ 1.0 × ULN

  Patients with known Gilbert syndrome who have serum bilirubin level ≤ 3 × ULN may be enrolled.
- For patients not receiving therapeutic anticoagulation: INR or aPTT ≤ 1.5 × ULN within 14 days prior to initiation of study treatment
- For patients receiving therapeutic anticoagulation: stable anticoagulant regimen and stable INR during the 14 days immediately preceding initiation of study treatment
- Creatinine clearance ≥ 30 mL/min (calculated using the Cockcroft-Gault formula)
- Serum albumin ≥ 25 g/L (≥ 2.5 g/dL)

• Representative formalin-fixed, paraffin-embedded (FFPE) tumor specimen in paraffin blocks (preferred) or at least 20 unstained slides, with an associated pathology report documenting ER, PgR, and HER2 negativity

  Tumor tissue should be of good quality based on total and viable tumor content and must be evaluated for PD-L1 expression prior to enrollment. Patients whose tumor tissue is not evaluable for PD-L1 expression are not eligible.

  If multiple tumor specimens are submitted, patients may be eligible if at least one specimen is evaluable for PD-L1. For the purpose of stratification, the PD-L1 score of the patient will be the maximum PD-L1 score among the samples.

  In Stage 2, if the recommendation from the iDMC is to expand to a PD-L1–positive population, no further stratification based on PD-L1 status will be conducted.

  Acceptable samples include core-needle biopsies for deep tumor tissue (minimum three cores) or excisional, incisional, punch, or forceps biopsies for cutaneous, subcutaneous, or mucosal lesions.

  Fine-needle aspiration, brushing, and cell pellet from cytology specimens are not acceptable.

• For women of childbearing potential: agreement to remain abstinent (refrain from heterosexual intercourse) or use contraceptive methods, and agreement to refrain from donating eggs, as defined below:

  Women must remain abstinent or use contraceptive methods that result in a failure rate of < 1% per year during the treatment period and for at least 5 months after the last dose of atezolizumab, or 1 month after the last dose of nab-paclitaxel, or 6 months after the last dose of doxorubicin, or 12 months after the last dose of cyclophosphamide, whichever is later. Women must refrain from donating eggs during this same period.

  A woman is considered to be of childbearing potential if she is postmenarcheal, has not reached a postmenopausal state (≥ 12 continuous months of amenorrhea with no identified cause other than menopause), and has not undergone surgical sterilization (removal of ovaries and/or uterus).

  Examples of contraceptive methods with a failure rate of < 1% per year when used consistently and correctly, include combined (estrogen and progestogen containing) hormonal contraception associated with inhibition of ovulation, progestogen-only hormonal contraception associated with inhibition of ovulation, bilateral tubal occlusion; male sterilization; intrauterine devices; intrauterine hormone-releasing system; and sexual abstinence.

  The reliability of sexual abstinence should be evaluated in relation to the duration of the clinical study and the preferred and usual lifestyle of the patient. Periodic abstinence (e.g., calendar, ovulation, symptothermal, or postovulation methods) and withdrawal are not acceptable methods of contraception.

"Use of this document is governed by the terms of use on the first page of this document."

- For men: agreement to remain abstinent (refrain from heterosexual intercourse) or use contraceptive measures and agreement to refrain from donating sperm, as defined below:

  With female partners of childbearing potential, men must remain abstinent or use a condom plus an additional contraceptive method that together result in a failure rate of < 1% per year during the treatment period and for 6 months after the last dose of nab-paclitaxel, cyclophosphamide, or doxorubicin. Men must refrain from donating sperm during this same period.

  With pregnant female partners, men must remain abstinent or use a condom during the treatment period and for 6 months after the last dose of nab-paclitaxel, cyclophosphamide, or doxorubicin to avoid exposing the embryo.

  The reliability of sexual abstinence should be evaluated in relation to the duration of the clinical trial and the preferred and usual lifestyle of the patient. Periodic abstinence (e.g., calendar, ovulation, symptothermal, or postovulation methods) and withdrawal are not acceptable methods of contraception.
- Women who are not postmenopausal (≥ 12 months of non–therapy-induced amenorrhea) or have undergone a sterilization procedure must have a negative serum pregnancy test result within 14 days prior to initiation of study drug
- Willingness and ability to comply with scheduled visits, treatment plans, laboratory tests, and other study procedures, including the completion of PRO questionnaires

<u>Exclusion Criteria</u>

Patients who meet any of the following criteria will be excluded from study entry:

- Prior history of invasive breast cancer
- Stage IV (metastatic) breast cancer
- Prior systemic therapy for treatment and prevention of breast cancer
- Previous therapy with anthracyclines or taxanes for any malignancy
- History of ductal carcinoma in situ (DCIS), except for patients treated exclusively with mastectomy > 5 years prior to diagnosis of current breast cancer
- History of pleomorphic lobular carcinoma in situ (LCIS), except for patients surgically managed > 5 years prior to diagnosis of current breast cancer (note that patients with non-pleomorphic LCIS [either untreated or treated with surgery] are allowed)
- Bilateral breast cancer
- Undergone incisional and/or excisional biopsy of primary tumor and/or axillary lymph nodes. Patients who have undergone SLNB at the baseline may be eligible only if the SLNB was free of invasive carcinoma. Any patient with a positive SLN (involved with invasive carcinoma) is ineligible to participate in this study.
- Axillary lymph node dissection prior to initiation of neoadjuvant therapy
- History of other malignancy within 5 years prior to screening, with the exception of those with a negligible risk of metastasis or death (e.g., 5-year OS of > 90%), such as adequately treated carcinoma in situ of the cervix, non-melanoma skin carcinoma, localized prostate cancer, or Stage I uterine cancer
- History of cerebrovascular accident within 12 months prior to randomization
- Cardiopulmonary dysfunction as defined by any of the following prior to randomization:
  - History of NCI CTCAE v4.0 Grade ≥ 3 symptomatic congestive heart failure or New York Heart Association (NYHA) criteria Class ≥ II
  - Angina pectoris requiring anti-anginal medication, serious cardiac arrhythmia not controlled by adequate medication, severe conduction abnormality, or clinically significant valvular disease
  - High-risk uncontrolled arrhythmias (i.e., atrial tachycardia with a heart rate > 100/min at rest, significant ventricular arrhythmia [ventricular tachycardia], or higher-grade atrioventricular [AV]-block [second-degree AV-block Type 2 [Mobitz 2] or third degree AV-block])

"Use of this document is governed by the terms of use on the first page of this document."

- Significant symptoms (Grade ≥ 2) relating to left ventricular dysfunction, cardiac arrhythmia, or cardiac ischemia
- Myocardial infarction within 12 months prior to randomization
- Uncontrolled hypertension (systolic blood pressure $>$ 180 mmHg and/or diastolic blood pressure $>$ 100 mmHg)
- Evidence of transmural infarction on ECG
- Requirement for oxygen therapy

- History of severe allergic, anaphylactic, or other hypersensitivity reactions to chimeric or humanized antibodies or fusion proteins
- Known hypersensitivity to biopharmaceuticals produced in Chinese hamster ovary cells
- Known allergy or hypersensitivity to the components of the atezolizumab formulation
- Known allergy or hypersensitivity to the components of the nab-paclitaxel, cyclophosphamide, or doxorubicin formulations
- Known allergy or hypersensitivity to filgrastim or pegfilgrastim formulations
- Active or history of autoimmune disease or immune deficiency, including but not limited to myasthenia gravis, myositis, autoimmune hepatitis, systemic lupus erythematosus, rheumatoid arthritis, inflammatory bowel disease, antiphospholipid syndrome, Wegener granulomatosis, Sjögren syndrome, Guillain-Barré syndrome, or multiple sclerosis with the following exceptions:

  Patients with a history of autoimmune-related hypothyroidism on a stable dose of thyroid replacement hormone may be eligible for this study.

  Patients with controlled Type I diabetes mellitus on a stable dose of insulin regimen may be eligible for this study.

  Patients with eczema, psoriasis, lichen simplex chronicus, or vitiligo with dermatologic manifestations only (e.g., patients with psoriatic arthritis are excluded) are permitted provided all of following conditions are met:

  - Rash must cover $<$ 10% of body surface area
  - Disease is well controlled at baseline and requires only low-potency topical corticosteroids
  - No occurrence of acute exacerbations of the underlying condition requiring psoralen plus ultraviolet A radiation, methotrexate, retinoids, biologic agents, oral calcineurin inhibitors, or high-potency or oral corticosteroids within the previous 12 months

- History of idiopathic pulmonary fibrosis, organizing pneumonia (e.g., bronchiolitis obliterans), drug-induced pneumonitis, idiopathic pneumonitis, or evidence of active pneumonitis on screening chest computed tomography (CT) scan

  History of radiation pneumonitis in the radiation field (fibrosis) is permitted.

- Positive HIV test at screening
- Active hepatitis B virus (HBV) infection, defined as having a positive hepatitis B surface antigen (HBsAg) test at screening

  Patients with a past or resolved HBV infection, defined as having a negative HBsAg test and a positive total hepatitis B core antibody (HBcAb) test at screening, are eligible for the study if active HBV infection is ruled out on the basis of HBV DNA viral load per local guidelines.

- Active hepatitis C virus (HCV) infection, defined as having a positive HCV antibody test at screening

  Patients who have a positive HCV antibody test are eligible for the study if a polymerase chain reaction (PCR) assay is negative for HCV RNA.

- Active tuberculosis
- Severe infections within 4 weeks prior to initiation of study treatment, including but not limited to hospitalization for complications of infection, bacteremia, or severe pneumonia

"Use of this document is governed by the terms of use on the first page of this document."

- Treatment with therapeutic oral or IV antibiotics within 2 weeks prior to initiation of study treatment

    Patients receiving prophylactic antibiotics (e.g., for prevention of a urinary tract infection or to prevent chronic obstructive pulmonary disease exacerbation) are eligible for the study.

- Major surgical procedure within 4 weeks prior to initiation of study treatment or anticipation of need for a major surgical procedure (other than anticipated breast surgery) during the course of the study
- Prior allogeneic stem cell or solid organ transplantation
- Administration of a live attenuated vaccine within 4 weeks prior to initiation of study treatment or anticipation of need for such a vaccine during the atezolizumab/placebo treatment or within 5 months after the last dose of atezolizumab/placebo

    Patients must agree not to receive live, attenuated influenza vaccine (e.g., FluMist®) within 4 weeks prior to randomization, during treatment or within 5 months following the last dose of atezolizumab (for patients randomized to atezolizumab).

- Any other disease, metabolic dysfunction, physical examination finding, or clinical laboratory finding giving reasonable suspicion of a disease or condition that contraindicates the use of an investigational drug or that may affect the interpretation of the results or render the patient at high risk from treatment complications
- Prior treatment with CD137 agonists or immune checkpoint–blockade therapies, including anti-CD40, anti–CTLA-4, anti–PD-1, and anti–PD-L1 therapeutic antibodies
- Treatment with systemic immunostimulatory agents (including but not limited to interferons, IL-2) within 4 weeks or 5 half-lives of the drug, whichever is longer, prior to initiation of study treatment
- Treatment with systemic immunosuppressive medications (including but not limited to prednisone, cyclophosphamide, azathioprine, methotrexate, thalidomide, and anti–tumor necrosis [anti-TNF] factor agents) within 2 weeks prior to initiation of study treatment or anticipation of need for systemic immunosuppressive medications during the study

    Patients who have received acute, low-dose, systemic immunosuppressant medications (e.g., a one-time dose of dexamethasone for nausea) may be enrolled in the study after discussion with and approval by the Medical Monitor.

    The use of inhaled corticosteroids and mineralocorticoids (e.g., fludrocortisone for adrenal insufficiency) is allowed.

- Pregnant or lactating, or intending to become pregnant during the study

    Women of childbearing potential must have a negative serum pregnancy test result within 14 days prior to initiation of study treatment.

End of Study

The end of the study is defined as the date when the last patient, last visit (LPLV) occurs for evaluation of secondary endpoints, or the date of Sponsor decision to end the study, whichever is earlier.

Length of Study

The total duration of the study for Stage 1 is expected to be approximately 51 months. If the iDMC recommends to expand enrollment (Stage 2), the duration of the study may increase to approximately 74 months.

<u>Investigational Medicinal Products</u>

The investigational medicinal products (IMPs) for this study are atezolizumab, its placebo, and nab-paclitaxel.

Atezolizumab and Placebo

Patients will receive 840 mg atezolizumab or placebo administered by IV infusion Q2W (14 [±3] days) for 20 weeks (i.e., 10 doses) in combination with nab-paclitaxel, doxorubicin, and cyclophosphamide chemotherapy (see below). Postoperatively, patients randomized to the

"Use of this document is governed by the terms of use on the first page of this document."

atezolizumab arm will continue to receive unblinded atezolizumab post-surgery at a fixed dose of 1200 mg by IV infusion every 3 weeks (Q3W) for 11 cycles, for a total of approximately 12 months of atezolizumab therapy; patients randomized to the placebo arm will stop receiving placebo.

Nab-Paclitaxel

Nab-paclitaxel will be administered as background treatment along with the non-IMPs doxorubicin, cyclophosphamide, and filgrastim/pegfilgrastim as specified below.

Non-Investigational Medicinal Products

Patients will receive nab-paclitaxel (125 mg/m^2) administered via IV infusion given over 30 minutes weekly for 12 weeks followed by doxorubicin (60 mg/m^2) + cyclophosphamide (600 mg/m^2) administered via IV infusion Q2W with filgrastim/pegfilgrastim support for 4 cycles (i.e., a total of 4 doses of doxorubicin and cyclophosphamide). The dose of cyclophosphamide should be capped at 1200 mg.

Statistical Methods

Primary Analysis

The primary efficacy objective for this study is to evaluate the efficacy of neoadjuvant atezolizumab + nab-pac–AC compared with placebo + nab-pac–AC in patients with T2-4d TNBC, as measured by pCR defined as eradication of tumor from both breast and lymph nodes (ypT0/is ypN0). The primary efficacy endpoint will be established following completion of neoadjuvant therapy and surgery.

The ITT as well as PD-L1–positive populations will be used for the primary analysis of pCR. In the primary analysis, patients whose pCR assessment was missing will be counted as not achieving a pCR. An estimate of the pCR rate and its 95% CI (Clopper and Pearson 1934) will be calculated for each treatment arm. The difference in pCR rates will be provided with 95% CI, using the normal approximation to the binomial distribution. For the ITT population, the Cochran-Mantel-Haenszel χ^2 test stratified according to tumor PD-L1 status (IC0 vs. IC1/2/3) and clinical stage at presentation (Stage II vs. III) will be used to test pCR rates between treatment groups at a two-sided significance level of 5%. For the PD-L1–positive population, similar test will be used with stratification only for clinical stage at presentation. An unstratified χ^2 versions of these tests will also be provided as a sensitivity analysis.

Determination of Sample Size

The study will first randomize approximately N1 = 204 patients in Stage 1 (1:1 randomization ratio). Based on information from these patients and a pre-specified adaptive rule, the decision will be made regarding whether or not to randomize approximately N2 = 120 patients in Stage 2 (1:1 randomization ratio).

The pre-defined one-sided type I error for the interim analysis at Stage 1 is $\alpha_1 = 0.0125$ (i.e., 50% of the total type I error). The co-primary endpoints in the ITT and PD-L1-positive populations at Stage 1 will be tested using a closed testing procedure using Simes' test for the intersection hypothesis. Test statistics in ITT and PD-L1-positive populations are always positively correlated, hence type I error rate control of the Simes test can be guaranteed in general.

Importantly, the p-value p_1 based on Stage 1 data only and the combination p-value p_{comb} have the same joint distribution under the null hypothesis as the p-values from a group-sequential test with two stages at information times $t_1 = w_1$ and $t_2 = 1$. Thus standard statistical software for group sequential designs can be used for the determination of critical values for the adaptive design. As the study design uses a critical value of $\alpha_1 = 0.0125$ (i.e., 50% of the total type I error) for Stage 1, this implies that a critical value of $\alpha_{comb} = 0.0184$ can be applied to the combination p-values.

"Use of this document is governed by the terms of use on the first page of this document."

LIST OF ABBREVIATIONS AND DEFINITIONS OF TERMS

Abbreviation	Definition
AC	doxorubicin + cyclophosphamide
ADA	anti-drug antibody, also known as an anti-therapeutic antibody (ATA)
AE	adverse event
AJCC	American Joint Committee on Cancer
ALND	axillary lymph node dissection
ASCO	American Society of Clinical Oncology
AV	atrioventricular
CAP	College of American Pathologists
CT	computed tomography
CV	coefficient of variation
CYP	cytochrome P450
DCIS	ductal carcinoma in situ
DFS	disease-free survival
EBC	early breast cancer
EC	Ethics Committee
ECHO	echocardiogram
ECOG	Eastern Cooperative Oncology Group
eCRF	electronic Case Report Form
EDC	electronic data capture
EFS	event-free survival
EORTC	European Organisation for Research and Treatment of Cancer
EQ-5D-5L	EuroQoL 5-Dimension, 5-Level
ER	estrogen receptor
ESMO	European Society for Medical Oncology
FACT-G	Functional Assessment of Cancer Therapy-General
FDA	Food and Drug Administration
FFPE	formalin-fixed paraffin embedded
G-CSF	granulocyte colony–stimulating factor
GHS	global health status
HBcAb	hepatitis B core antibody
HBsAg	hepatitis B surface antigen
HBV	hepatitis B virus
HCV	hepatitis C virus
HER2	human epidermal growth factor receptor 2

"Use of this document is governed by the terms of use on the first page of this document."

Abbreviation	Definition
HIPAA	Health Insurance Portability and Accountability Act
HR	hazard ratio
HRQoL	health-related quality of life
IC	tumor-infiltrating immune cell
ICH	International Council for Harmonisation
iDMC	independent Data Monitoring Committee
Ig	immunoglobulin
IHC	immunohistochemistry
IMP	investigational medicinal product
IND	Investigational New Drug
IRB	Institutional Review Board
IRR	infusion-related reaction
ISH	in situ hybridization
ITT	intent to treat
IV	intravenous
IxRS	interactive voice or web-based response system
LCIS	lobular carcinoma in situ
LPLV	last patient, last visit
LVEF	left ventricular ejection fraction
MRI	magnetic resonance imaging
MUGA	multiple-gated acquisition
nab-pac–AC	nab-paclitaxel followed by doxorubicin and cyclophosphamide
NAST	neoadjuvant systemic therapy
NCCN	National Comprehensive Cancer Network
NCI CTCAE	National Cancer Institute Common Terminology Criteria for Adverse Events
NGS	next-generation sequencing
NYHA	New York Heart Association
OS	overall survival
pCR	pathologic complete response
PCR	polymerase chain reaction
PD	pharmacodynamic
PD-1	programmed death–1
PD-L1	programmed death–ligand 1
PET	positron emission tomography
P-gp	P-glycoprotein

"Use of this document is governed by the terms of use on the first page of this document."

Abbreviation	Definition
PgR	progesterone receptor
PK	pharmacokinetic
PRO	patient-reported outcome
PVC	polyvinyl chloride
Q	question
QW	weekly
Q2W	every 2 weeks
Q3W	every 3 weeks
QLQ-C30	Quality of Life Questionnaire Core 30
RBR	Research Biosample Repository
SAE	serious adverse event
SAP	Statistical Analysis Plan
SLNB	sentinel lymph node biopsy
T3	triiodothyronine
TNBC	triple-negative breast cancer
TNF	tumor necrosis factor
ULN	upper limit of normal
WGS	whole genome sequencing

"Use of this document is governed by the terms of use on the first page of this document."

1. BACKGROUND

1.1 BACKGROUND ON BREAST CANCER

Globally, breast cancer is the second most common invasive malignancy and the most common cause of cancer-related mortality in women. The majority of breast cancers in the Western world are diagnosed when the cancer is still confined to the breast, with or without locoregional lymph node spread (Sant et al. 2003; Jemal et al. 2011; Ferlay et al. 2013; Howlader et al. 2016). At these early stages (I–III, early breast cancer [EBC]), the largely asymptomatic disease is usually operable and can be treated with curative intent.

Triple-negative breast cancer (TNBC) is defined by the absence of immunostaining for estrogen receptor (ER), progesterone receptor (PgR), and human epidermal growth factor receptor 2 (HER2). Overall, approximately 15%–20% of all breast cancers are classified as TNBC. Large-scale comprehensive genomic analyses have characterized the heterogeneous nature of TNBCs and their diverse gene expression patterns and underlying genomic changes, but these insights have not yet provided clear guidance for the identification of clinically effective targeted therapies currently under laboratory and clinical investigation. Unfortunately, TNBCs are more likely to have aggressive features, such as a high proliferative rate, and exhibit an invasive phenotype. Patients with TNBCs exhibit a poor clinical outcome, generally with rapid progression and a shorter time to local and distant relapse (Dent et al. 2007).

Early-stage TNBC comprises 10%–20% of all new diagnoses of EBC defined as Stages I–III (Lehmann et al. 2011; Howlader et al. 2016). Three-year event-free survival (EFS) rates between 74% and 76% have been reported for patients with TNBC who have received neoadjuvant anthracycline/taxane therapy (Sikov et al. 2015). Upon relapse, patients with metastatic TNBC have poor outcomes, with rapid progression and decreased overall survival (OS) (Kassam et al. 2009).

1.2 TREATMENT FOR EARLY TRIPLE-NEGATIVE BREAST CANCER

Multi-agent chemotherapy regimens have proven benefit as neoadjuvant/adjuvant therapy for early-stage TNBC, improving both disease-specific and OS outcomes (Berry et al. 2006; Senkus et al. 2015; NCCN 2016). Chemotherapy intended to reduce the risk of relapse may be given preoperatively (neoadjuvant) or postoperatively (adjuvant) to patients with EBC and is currently recommended for TNBC patients with Stage I–III disease. Globally, chemotherapy is most often administered as adjuvant therapy; however, rates of neoadjuvant treatment use are increasing.

The most effective chemotherapy combinations used for early-stage TNBC include anthracyclines, topoisomerase II inhibitors, platinum agents, cyclophosphamide, and/or taxanes (Early Breast Cancer Trialists' Collaborative Group 2005; Peto et al. 2012). Studies looking at optimizing the dose and schedule of EBC chemotherapy regimens (Citron et al. 2003; Sparano et al. 2008; Budd et al. 2015) have established one of the

"Use of this document is governed by the terms of use on the first page of this document."

optimal regimens with respect to maximizing efficacy as doxorubicin 60 mg/m^2 and cyclophosphamide 600 mg/m^2 administered every 2 weeks (Q2W) for 4 cycles, followed by weekly (QW) paclitaxel 80 mg/m^2 for 12 weeks; the regimen is included as a preferred option in international guidelines (Senkus et al. 2015; NCCN 2016).

However, despite having received standard anthracycline-taxane–based therapy, approximately 30%–40% of patients with clinically localized disease at diagnosis develop metastatic disease and die of the cancer (Haffty et al. 2006; Tan et al. 2008; Budd et al. 2015). Thus, there is a substantial need to improve long-term treatment outcomes for patients with early-stage TNBC.

1.3 BACKGROUND ON ATEZOLIZUMAB

Atezolizumab is a humanized immunoglobulin (Ig) G1 monoclonal antibody that targets programmed death–ligand 1 (PD-L1) and inhibits the interaction between PD-L1 and its receptors, programmed death–1(PD-1) and B7.1 (also known as CD80), both of which function as inhibitory receptors expressed on T cells. Therapeutic blockade of PD-L1 binding by atezolizumab has been shown to enhance the magnitude and quality of tumor-specific T-cell responses, resulting in improved anti-tumor activity (Fehrenbacher et al. 2016; Rosenberg et al. 2016). Atezolizumab has minimal binding to Fc receptors, thus eliminating detectable Fc-effector function and associated antibody-mediated clearance of activated effector T cells.

Atezolizumab shows anti-tumor activity in both nonclinical models and cancer patients and is being investigated as a potential therapy in a wide variety of malignancies. Atezolizumab is being studied as a single agent as well as in combination with chemotherapy, targeted therapy, and cancer immunotherapy.

Refer to the Atezolizumab Investigator's Brochure for additional details on nonclinical and clinical studies.

Clinical Experience with Atezolizumab in Triple-Negative Breast Cancer

Atezolizumab is currently being tested in multiple Phase I, II, and III studies, both as monotherapy and in combination with several anti-cancer therapies in multiple indications (see the Atezolizumab Investigator's Brochure for full study descriptions and available data).

Much of the available safety and efficacy data in TNBC are from two studies:

- Study PCD4989g, a Phase Ia, multicenter, first-in-human, open-label, dose-escalation trial evaluating the safety, tolerability, immunogenicity, pharmacokinetics, exploratory pharmacodynamics, and preliminary evidence of biologic activity of atezolizumab monotherapy in patients with locally advanced or metastatic solid malignancies or hematologic malignancies. Data from the TNBC cohort have been reported and demonstrate promising safety and efficacy results mainly in 1L and PD-L1–positive patients (Emens et al. 2018).

"Use of this document is governed by the terms of use on the first page of this document."

- Study GP28328, a Phase Ib multi-cohort trial of the safety and pharmacology of atezolizumab administered with bevacizumab and/or chemotherapy in patients with advanced solid tumors. Arm F of the trial is evaluating atezolizumab administered in combination with weekly nab-paclitaxel in patients with TNBC. Data from Arm F have been reported and support the use of atezolizumab in combination with chemotherapy in TNBC patients. Confirmed responses were seen in 46% (95% CI: 19%, 75%) of patients with first-line metastatic TNBC and 38% (95% CI: 21%, 56%) of patients regardless of line of therapy and PD-L1 expression. No new safety signals were seen (Adams et al. 2016). Results from Study GP28328 support the hypothesis that tumor-cell killing by cytotoxic chemotherapy may expose the immune system to high levels of tumor antigens. Boosting tumor-specific T-cell immunity in this setting by blocking the PD-L1 pathway may result in deeper and more durable responses than those observed with standard chemotherapy alone (Merritt et al. 2003; Apetoh et al. 2007), and these responses may reasonably occur in tumors regardless of PD-L1 expression. The inclusion of patients with all levels of PD-L1 expression in this study will enable a robust assessment of this hypothesis.

A third study (WO29522, IMpassion130) has read out for progression-free survival (Schmid et al. 2018). This is a global, multicenter, double-blind, two-arm, randomized, placebo-controlled study designed to evaluate the efficacy and safety of atezolizumab administered with nab-paclitaxel in patients with metastatic or unresectable locally advanced TNBC who have not received prior systemic therapy for metastatic breast cancer.

Refer to the Atezolizumab Investigator's Brochure for details on the clinical studies involving atezolizumab as well as updated detailed safety and efficacy results.

1.4 STUDY RATIONALE AND BENEFIT-RISK ASSESSMENT

Encouraging clinical data emerging in the field of tumor immunotherapy have demonstrated that therapies focused on enhancing T-cell responses against cancer can result in a significant survival benefit in patients with Stage IV cancer (Hodi et al. 2010; Kantoff et al. 2010; Chen et al. 2012).

The PD-L1 pathway serves as an immune checkpoint to temporarily dampen immune responses in states of chronic antigen stimulation, such as chronic infection or cancer. PD-L1 is an extracellular protein that downregulates immune responses through binding to its two receptors, PD-1 and B7.1. PD-1 is an inhibitory receptor expressed on T cells following T-cell activation, and expression is sustained in states of chronic stimulation (Blank et al. 2005; Keir et al. 2008). B7.1 is a molecule expressed on antigen-presenting cells and activated T cells. Binding of PD-L1 to PD-1 and B7.1 inhibits T-cell proliferation and activation, cytokine production, and cytolytic activity, leading to the functional inactivation or exhaustion of T cells (Butte et al. 2007; Yang et al. 2011). Overexpression of PD-L1 on tumor and tumor-infiltrating immune cells has been reported to impede anti-tumor immunity, resulting in immune evasion (Blank and

"Use of this document is governed by the terms of use on the first page of this document."

Mackensen 2007). Therefore, interruption of the PD-L1 pathway represents an attractive strategy for restoring tumor-specific T-cell immunity.

Targeting the PD-L1 pathway with atezolizumab has demonstrated activity in patients with advanced malignancies. Objective responses have been observed across a broad range of malignancies, including non–small-cell lung cancer, urothelial carcinoma, renal cell carcinoma, melanoma, colorectal cancer, head and neck cancer, gastric cancer, breast cancer, and sarcoma. In particular, available data from the TNBC cohorts in Studies PCD4989g and GP28328 have demonstrated activity and durable responses in patients receiving atezolizumab as monotherapy or in combination with nab-paclitaxel (see the Atezolizumab Investigator's Brochure for detailed efficacy results).

Atezolizumab has been generally well tolerated. *Immune-mediated* adverse events are consistent with the role of the PD-L1/PD-1 pathway in regulating peripheral tolerance. Given the mechanism of action of atezolizumab, events associated with inflammation and/or *immune-mediated* adverse events are closely monitored in the atezolizumab clinical program. *Immune-mediated* adverse events associated with atezolizumab include dermatologic, hepatic, endocrine, gastrointestinal, respiratory, and neurological events. To date, the majority of these events have been manageable without requiring treatment discontinuation. Refer to the Atezolizumab Investigator's Brochure for additional details regarding *immune-mediated* adverse events observed in patients treated with atezolizumab.

Atezolizumab in combination with taxanes (including paclitaxel and nab-paclitaxel) has also been investigated or is currently being investigated as part of several studies including Studies GP28328, GO29436 (IMpower150), GO29537 (IMpower130), GO30140, and WO29522 (IMpassion130). Atezolizumab in combination with chemotherapeutic regimens including anthracyclines and cyclophosphamide is currently being investigated as part of Studies BO29563 and GO29831. Thus far, reported adverse events observed were similar to those experienced with the individual components of the study treatment and have generally been manageable. According to data available from the TNBC cohort in Study GP28328 (atezolizumab in combination with nab-paclitaxel), the most commonly occurring adverse events (≥30% incidence) were fatigue (65.6%); diarrhea (50%); nausea, constipation, and alopecia (40.6% each); peripheral sensory neuropathy (37.5%); pyrexia, cough, and decreased neutrophil count (34.4% each); and peripheral neuropathy and neutropenia (31.3% each). Three TNBC patients (9.4%) had serious adverse events assessed as related to study treatment; these serious adverse events were pyrexia, anemia, and mycoplasmal pneumonia. Refer to the Atezolizumab Investigator's Brochure for additional details.

The risk of overlapping toxicities between atezolizumab and nab-paclitaxel, doxorubicin, and cyclophosphamide is thought to be minimal based on the mechanism of action of each product. Known serious risks associated with nab-paclitaxel include bone marrow suppression (neutropenia, anemia, and thrombocytopenia), elevation of liver enzymes,

"Use of this document is governed by the terms of use on the first page of this document."

alopecia, peripheral neuropathy, infection, and pneumonitis. Known serious risks of doxorubicin include bone marrow suppression (neutropenia, anemia, and thrombocytopenia), alopecia, nausea and vomiting, skin reactions, and cardiotoxicity. Known serious risks associated with cyclophosphamide include bone marrow suppression (neutropenia, anemia, and thrombocytopenia), infection, renal toxicity, cystitis, cardiotoxicity, and pneumonitis. In order to mitigate any potential risks associated with this combination regimen, patients with cardiopulmonary dysfunction will be excluded from the study (see Section 4.1.2), and an independent Data Monitoring Committee (iDMC) will regularly review unblinded safety data (see Section 3.1.2). Cardiac function will also be closely monitored, including regular measurements of left ventricular ejection fraction (LVEF) throughout the study.

This study will enroll patients with cT2–cT4d TNBC tumors who will receive neoadjuvant therapy prior to surgery. Given the relatively poor prognosis for these patients (see Section 3.3.2), the limited availability of targeted agents, and the ability to assess the benefit–risk profile in the neoadjuvant setting (see Section 3.3.4), this population and setting are considered appropriate for trials of novel therapeutic candidates. The benefit-risk ratio for atezolizumab in combination with nab-paclitaxel followed by cyclophosphamide and doxorubicin is expected to be acceptable in this setting.

2. OBJECTIVES AND ENDPOINTS

This study (Study WO39392, also known as IMpassion031) will evaluate the efficacy, safety, and pharmacokinetics of neoadjuvant nab-paclitaxel and atezolizumab followed by doxorubicin and cyclophosphamide with atezolizumab (referred to as atezolizumab + nab-pac–AC) or neoadjuvant nab-paclitaxel and placebo followed by doxorubicin and cyclophosphamide with placebo (referred to as placebo + nab-pac–AC) in patients with T2-4d TNBC. Specific objectives and corresponding endpoints for the study are outlined in Table 1.

Table 1 Objectives and Corresponding Endpoints

Objectives	Corresponding Endpoints
**Primary Efficacy Objective:**	
• To evaluate the efficacy of atezolizumab + nab-pac–AC compared with placebo + nab-pac–AC in the neoadjuvant setting	• pCR defined as eradication of tumor from both breast and lymph nodes (ypT0/is ypN0) in the following:<br>– All patients (ITT population)<br>– Subpopulation of patients with PD-L1–positive tumor status (IC1/2/3)

"Use of this document is governed by the terms of use on the first page of this document."

Objectives	Corresponding Endpoints
**Secondary Efficacy Objectives:**	
• To evaluate the efficacy of atezolizumab + nab-pac–AC compared with placebo + nab-pac–AC in the neoadjuvant setting	• EFS defined as the time from randomization until documented disease recurrence, progression, or death from any cause in all patients (ITT population) and in the subpopulation with PD-L1–positive tumor status<br>• DFS defined as the time from surgery until documented disease recurrence or death from any cause in all patients (ITT population) who undergo surgery and in the subpopulation of patients with PD-L1–positive tumor status who undergo surgery<br>• OS defined as the time from randomization to the date of death from any cause in all patients (ITT population) and in the subpopulation with PD-L1–positive tumor status
• To evaluate PROs of function and HRQoL associated with atezolizumab + nab-pac–AC compared with placebo + nab-pac–AC, measured by the functional and HRQoL scales of the EORTC QLQ-C30	• Mean and mean changes from baseline score in function (role, physical) and GHS/HRQoL by cycle and between treatment arms as assessed by the functional and HRQoL scales of the EORTC QLQ-C30
**Exploratory Efficacy Objectives:**	
• To evaluate the efficacy of atezolizumab + nab-pac–AC compared with placebo + nab-pac–AC in the neoadjuvant setting	• Proportion of patients undergoing breast-conserving surgery<br>• RCB index<br>• Correlation of RCB with other clinical endpoints (if deemed appropriate)
• To evaluate PROs of disease/treatment-related symptoms associated with atezolizumab + nab-pac–AC compared with placebo + nab-pac–AC, as measured by the EORTC QLQ-C30	• Mean and mean changes from baseline score in disease/treatment-related symptoms by cycle and between treatment arms as assessed by all symptom items/scales of the EORTC QLQ-C30
• To evaluate any treatment burden patients may experience associated with the addition of atezolizumab to nab-pac–AC compared with placebo + nab-pac–AC, as measured by a single item (GP5: "I am bothered by side effects of treatment") from the physical well-being subscale of the FACT-G Quality of Life instrument	• Proportion of patients reporting each response option at each assessment timepoint by treatment arm for item GP5 from the FACT-G
• To evaluate and compare between treatment arms patient's health utility as measured by the EQ-5D-5L questionnaire to generate utility scores for use in economic models	• Utility scores of the EQ-5D-5L questionnaire

"Use of this document is governed by the terms of use on the first page of this document."

Objectives	Corresponding Endpoints
**Safety Objective:**	
• To evaluate the safety and tolerability of atezolizumab + nab-pac–AC compared with placebo + nab-pac–AC	• Occurrence and severity of adverse events as defined by NCI CTCAE v4.0
**Pharmacokinetic Objectives:**	
• To characterize the pharmacokinetics of atezolizumab when administered in combination with nab-pac–AC chemotherapy	• Serum concentration of atezolizumab at specified timepoints
**Immunogenicity Objective:**	
• To evaluate the immune response to atezolizumab	• Incidence of ADAs during the study and the prevalence of ADAs at baseline
**Exploratory Immunogenicity Objective:**	
• To evaluate potential effects of ADAs	• Relationship between ADA status and efficacy, safety, and PK endpoints
**Exploratory Biomarker Objective:**	
• To assess predictive, prognostic, and pharmacodynamic exploratory biomarkers in archival and/or fresh tumor tissue and blood and their association with efficacy endpoints including but not limited to pCR	• Relationship between PD-L1 IHC and efficacy endpoints other than pCR<br>• Relationship between tumor derived RNA-based immune gene signatures and efficacy endpoints, including but not limited to pCR<br>• Relationship between tumor-based tumor infiltrating lymphocytes and/or CD8 IHC and efficacy endpoints, including but not limited to pCR<br>• Pharmacodynamic changes in cancer-related immune, stroma and tumor immune biology parameters on-treatment by, but not limited to, gene expression and IHC in baseline, on-treatment, and residual disease tumor tissues<br>• Relationship of baseline and on-treatment plasma biomarkers and efficacy endpoints, including, but not limited to pCR

ADA = anti-drug antibody; atezolizumab + nab-pac–AC = nab-paclitaxel and atezolizumab followed by doxorubicin and cyclophosphamide with atezolizumab; DFS = disease-free survival; EFS = event-free survival; EORTC = European Organisation for Research and Treatment of Cancer; EQ-5D-5L = EuroQoL 5-Dimension, 5-Level; FACT–G = Functional Assessment of Cancer Therapy–General; GHS = global health status; HRQoL = health-related quality of life; IC = tumor-infiltrating immune cell; IHC = immunohistochemistry; ITT = intent to treat; NCI CTCAE = National Cancer Institute Common Terminology Criteria for Adverse Events; OS = overall survival; pCR = pathologic complete response; PD–L1 = programmed death–ligand 1; PK = pharmacokinetics; placebo + nab-pac–AC = neoadjuvant nab-paclitaxel and placebo followed by doxorubicin and cyclophosphamide with placebo; PRO = patient-reported outcome; QLQ-C30 = Quality of Life Questionnaire Core 30; RCB = residual cancer burden.

"Use of this document is governed by the terms of use on the first page of this document."

3. STUDY DESIGN

3.1 DESCRIPTION OF THE STUDY

3.1.1 Overview of Study Design

This is a global Phase III, double-blind, randomized, placebo-controlled study designed to evaluate the efficacy and safety of neoadjuvant treatment with atezolizumab + nab-pac-AC, or placebo + nab-pac–AC in patients eligible for surgery with initial clinically assessed T2-4d TNBC.

Female and male patients aged ≥ 18 years with an Eastern Cooperative Oncology Group (ECOG) performance status of 0 or 1 who have histologically confirmed invasive TNBC with a primary tumor size of > 2 cm are eligible (see Appendix 10).

HER2 and ER/PgR status will be used to define TNBC. HER2 negativity will be defined by central laboratory assessment using in situ hybridization (ISH) or immunohistochemistry (IHC) assays per American Society of Clinical Oncology/College of American Pathologists (ASCO/CAP) criteria (Wolff et al. 2013), and ER/PgR negativity will be defined by using IHC per ASCO/CAP criteria (Hammond et al. 2010). Central laboratory assessment will occur prior to randomization. Patients whose tumors are not confirmed to be triple negative will not be eligible. Patients whose tumor tissue is not evaluable for PD-L1 will not be eligible.

Patients who do not initially meet all eligibility criteria, other than TNBC status, may be rescreened once.

This study has an adaptive design consisting of two stages. Stage 1 is the randomization and treatment of approximately 204 patients. At the end of Stage 1, an interim analysis for efficacy will be done by an iDMC. Then, a recommendation will be made to either continue the study unchanged or to expand the patient population (Stage 2; approximately 120 additional patients). If the recommendation is to expand the patient population into Stage 2, the Sponsor will remain blinded to the results of the interim analysis performed at the end of Stage 1.

Patients who have consented and are eligible will be randomized in a 1:1 ratio to receive either of the following treatment groups:

- **Arm A**: atezolizumab (840 mg) administered via intravenous (IV) infusion Q2W in combination with nab-paclitaxel (125 mg/m^2) administered via IV infusion QW for 12 weeks followed by atezolizumab (840 mg) administered Q2W in combination with doxorubicin (60 mg/m^2) + cyclophosphamide (600 mg/m^2) administered Q2W via IV infusions with filgrastim/pegfilgrastim support for 4 cycles. Patients randomized to the atezolizumab arm will continue to receive unblinded atezolizumab post-surgery at a fixed dose of 1200 mg by IV infusion every 3 weeks (Q3W) for 11 cycles, for a total of approximately 12 months of atezolizumab therapy.

"Use of this document is governed by the terms of use on the first page of this document."

- **Arm B**: placebo administered Q2W via IV infusion in combination with nab-paclitaxel (125 mg/m^2) administered QW via IV infusion for 12 weeks followed by placebo administered Q2W in combination with doxorubicin (60 mg/m^2) + cyclophosphamide (600 mg/m^2) administered Q2W via IV infusions with filgrastim/pegfilgrastim support for 4 cycles. Patients randomized to the placebo arm will be unblinded post-surgery and will continue to be followed.

The study schema is presented in Figure 1.

"Use of this document is governed by the terms of use on the first page of this document."

Figure 1 Study Schema

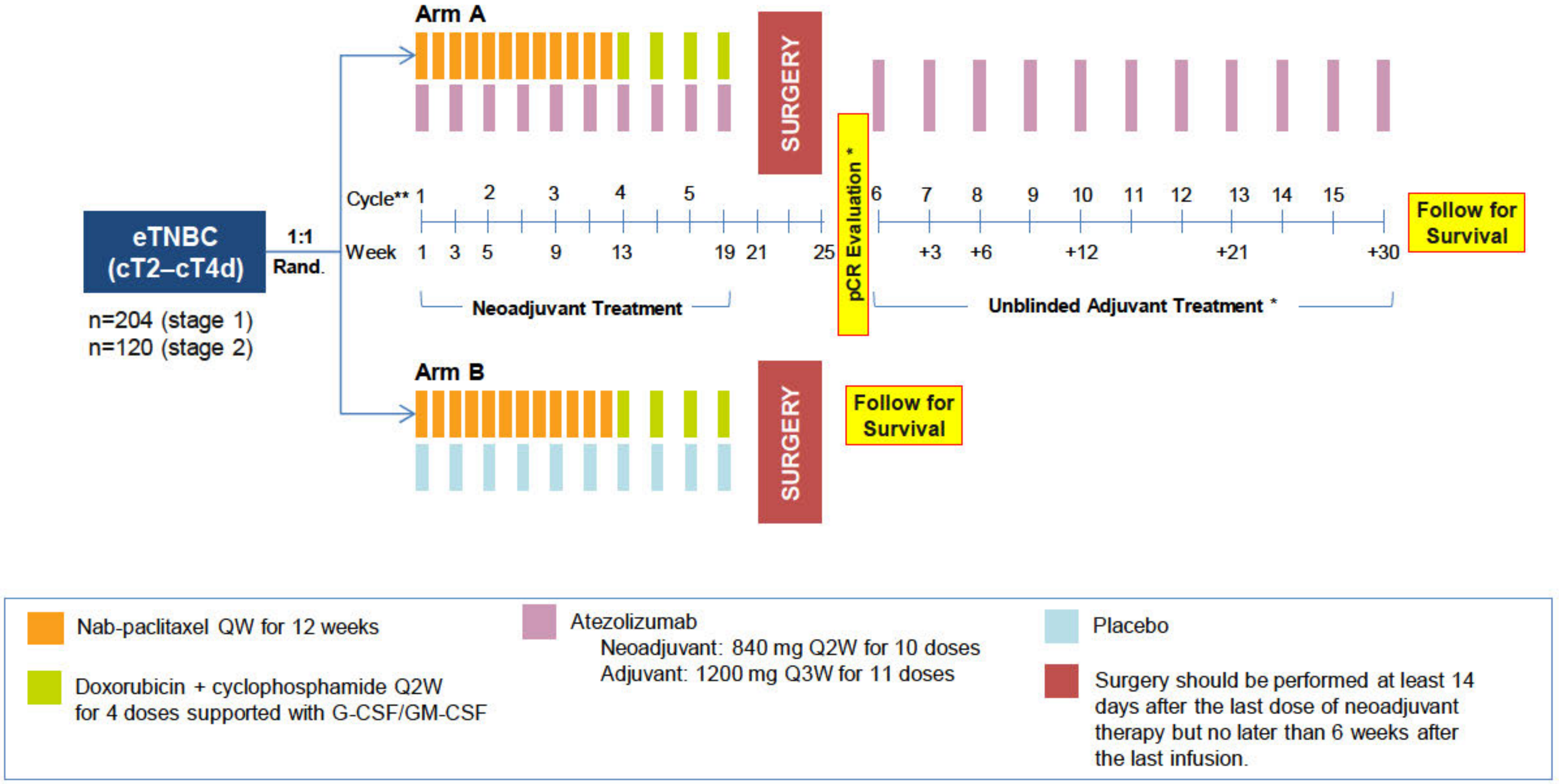

* If no pCR, post-op treatment is allowed in both arms per PI discretion. For patients in Arm A, chemotherapy and/or radiotherapy may be administered concurrently with atezolizumab after discussion with Medical Monitor.
** In the neoadjuvant setting, 1 Cycle = 4 weeks; in the adjuvant setting, 1 Cycle = 3 weeks

G-CSF = granulocyte colony-stimulating factor; GM-CSF = granulocyte-macrophage colony-stimulating factor; pCR = pathologic complete response; QW = weekly; Q2W = every 2 weeks; Q3W = every 3 weeks; TNBC = triple-negative breast cancer.

"Use of this document is governed by the terms of use on the first page of this document."

For patients in Stage 1 of the study, randomization will be stratified by the following factors:

- American Joint Committee on Cancer (AJCC) stage at diagnosis (II vs. III; see below for evaluation and classification of lymph nodes)
- Tumor PD-L1 status (tumor-infiltrating immune cell [IC] IC0 [<1% PD-L1 expressing IC per tumor area] vs. IC1/2/3 [≥1% PD-L1 expressing IC per tumor area])

Depending on the iDMC recommendation, Stage 2 could follow either an all-comer or PD-L1 enrichment design. If an all-comer design is recommended, the patients enrolled in Stage 2 will be stratified the same as patients enrolled in Stage 1. If an enrichment design is recommended, only PD-L1–positive patients will be enrolled, and the AJCC stage at diagnosis will be used as the sole randomization stratification factor.

Patients who discontinue neoadjuvant therapy early as a result of disease progression must be discontinued from all study treatment, will be managed as per local practice, and will be followed for survival only. Patients who discontinue prematurely from the study will not be replaced.

Any patient who receives non-protocol therapy prior to surgery will be discontinued from study treatment and will be managed as per local practice; these patients will remain on study for survival follow-up.

The primary efficacy endpoint (pathologic complete response [pCR]; ypT0/is ypN0) will be established via local review following completion of neoadjuvant therapy and surgery. Pathologists who review study specimens must utilize the evaluations and assessments outlined in the Pathology Manual. Investigator/individual patient and Sponsor unblinding will occur after pCR assessment as detailed in Section 4.2. Surgery should be performed at least 14 days after the last dose of neoadjuvant therapy but no later than 6 weeks after the last infusion. Platelet counts should be checked prior to surgery and should be ≥75,000 cells/μL.

Patients with clinically positive axillary nodes by physical examination or by any radiographic imaging at baseline should undergo fine-needle aspiration or core-needle biopsy prior to randomization followed by axillary lymph node dissection (ALND) at time of definitive surgery. The results of the baseline fine-needle aspiration or core-needle biopsy will determine the nodal staging, so that patients with a positive biopsy result should be staged as lymph-node-positive (N1-N3c) whereas patients with a negative or equivocal biopsy result should be staged as lymph-node-negative (N0) regardless of any other clinical measurements (see Appendix 12).

Baseline fine-needle aspiration (FNA) or core-needle biopsy is not mandatory for clinically/radiologically enlarged/suspicious internal mammary, subpectoral, infraclavicular, or supraclavicular lymph nodes. Investigators are advised to follow local practice guidelines regarding assessment of internal mammary, subpectoral,

"Use of this document is governed by the terms of use on the first page of this document."

infraclavicular, or supraclavicular lymph nodes. Management of internal mammary, subpectoral, infraclavicular, or supraclavicular lymph nodes at the time of definitive surgery will be based on local practice guidelines. Patients with baseline axillary lymph nodes involvement who may also have clinically/radiologically enlarged internal mammary, subpectoral, infraclavicular, or supraclavicular lymph nodes must still undergo ALND as described earlier and in Appendix 11 and 12.

For patients with baseline clinically/radiologically enlarged/suspicious internal mammary, subpectoral, infraclavicular, or supraclavicular lymph nodes, pCR may be reported **only if** all the following conditions are met:

1. Presurgical imaging assessment of internal mammary, subpectoral, infraclavicular, or supraclavicular lymph nodes does not show any enlarged/suspicious lymph node(s) after neoadjuvant study treatment.

AND

2. Pathological evaluation of the breast and all resected lymph node(s) shows no residual invasive disease (i.e., pCR; ypT0/is ypN0).

Postoperative radiotherapy may be administered based on local practice guidelines and after approval from the Medical Monitor has been obtained.

In patients with clinically or fine-needle biopsy (FNA)/core needle biopsy-proven negative axillary nodes at baseline, axillary surgical management after completion of neoadjuvant therapy should include sentinel lymph node biopsy (SLNB) or ALND. If SLNB is conducted, it is strongly recommended that more than one lymph node (two to three minimum) be removed and all patients with positive macrometastases in sentinel nodes should undergo ALND regardless of the number of positive nodes. All patients with T4 tumors should undergo ALND or current standard of care as described in international or national guidelines (see Appendix 11).

Postoperative patient management for those in either treatment arm may include radiotherapy as clinically indicated, and management of patients who do not achieve a pCR should follow current standard-of-care guidelines. For those randomized to receive atezolizumab, patients may receive this therapy simultaneously with atezolizumab.

For those randomized to receive atezolizumab, the first dose of postoperative atezolizumab should be administered within 45 days of surgery.

Efficacy, safety, laboratory measurements, patient-reported outcomes (PROs), and pharmacokinetics will be assessed throughout the study. The first 26 patients enrolled (approximately 13 patients in the control arm and approximately 13 patients in the atezolizumab containing arm) will undergo additional cardiac safety monitoring as part of a cardiac safety cohort. Following completion of study treatment and surgery, all

"Use of this document is governed by the terms of use on the first page of this document."

patients will continue to be followed for efficacy, safety, and PRO objectives until the end of the study. No interim efficacy analyses for early stopping are planned.

Safety assessments will include the occurrence and severity of adverse events and laboratory abnormalities graded per National Cancer Institute Common Terminology Criteria for Adverse Events (NCI CTCAE) Version 4.0. Laboratory safety assessments will include the regular monitoring of hematology and blood chemistry. Serum samples will be collected to monitor chemotherapy and atezolizumab pharmacokinetics and to detect the presence of antibodies to atezolizumab. Patient samples, including tumor tissues, as well as plasma and blood, will be collected for exploratory biomarker assessments.

3.1.2 Independent Data Monitoring Committee

An iDMC will evaluate the primary efficacy endpoint of pCR in the intent-to-treat (ITT) population (defined as all randomized patients) and in the PD-L1–positive subpopulation based on an interim analysis of efficacy data from the Stage 1 patients (approximately 204 enrolled patients). The iDMC will make a recommendation either to continue the study unchanged or to enroll an additional 120 patients (Stage 2). The decision rules to be applied at the interim analysis will be clearly expressed to the iDMC and documented in the iDMC charter so that the study can be conducted with the Sponsor remaining completely blinded to all results at this stage.

The iDMC will evaluate safety data and study conduct on a regular basis during the study until the primary analysis of pCR, which is performed at Stage 1 (approximately 204 patients) or Stage 2 (approximately 324 patients), if the iDMC recommends extending the target population. After which, iDMC review of the study data will be discontinued.

Sponsor affiliates will be excluded from iDMC membership. The iDMC will follow a charter that outlines the iDMC roles and responsibilities.

Unblinded safety data will be reviewed on a regular basis by the iDMC. To assess the potential cardiac toxicity of the combination of anthracyclines and atezolizumab, the iDMC will review data from a cardiac safety cohort involving additional cardiac monitoring in the first 26 patients enrolled (approximately 13 patients in the control arm and approximately 13 patients in the atezolizumab containing arm) after all 26 patients have completed or have discontinued the neoadjuvant portion of the study. A safety data review will also occur when at least 50 patients have received at least 2 cycles of atezolizumab/placebo and doxorubicin and cyclophosphamide (approximately 25 patients in each treatment arm). Subsequent safety reviews will occur approximately once every 6 months during the study until analysis of the primary endpoint.

All summaries and analyses for the iDMC review will be prepared by an independent Data Coordinating Center.

"Use of this document is governed by the terms of use on the first page of this document."

After reviewing the data, the iDMC will provide a recommendation to the Sponsor as described in the iDMC Charter. Final decisions will rest with the Sponsor.

Any outcomes of these data reviews that affect study conduct will be communicated in a timely manner to the investigators for notification of their respective Institutional Review Boards/Ethics Committees (IRBs/ECs).

3.2 END OF STUDY AND LENGTH OF STUDY

The end of the study is defined as the date when the last patient, last visit (LPLV) occurs for evaluation of secondary endpoints. Thirty-six months after randomization of the last patient, all patients will be contacted for final evaluation of the secondary endpoints of EFS, DFS, OS, and PROs. LPLV for Stage 1 is expected to occur approximately 51 months after the first patient is randomized. If the study expands to Stage 2, LPLV may occur at approximately 74 months after the first patient is randomized.

Recruitment for Stage 1 has stopped and was approximately 15 months. The recruitment period may increase to approximately 38 months if the iDMC recommends to expand to Stage 2.

The primary efficacy endpoint, pCR, will be analyzed at the interim analysis once approximately 204 patients enrolled in Stage 1 have received surgery, approximately 6 months after the last of these patients has been randomized. This interim analysis is expected to take place approximately 21 months after the first patient is randomized. If the iDMC recommends to expand to Stage 2, a second (final) pCR analysis will be performed at approximately 44 months after the first patient has been randomized, depending on the selected patient population.

Secondary efficacy endpoints of EFS, DFS, and OS will be analyzed approximately 36 months after randomization of the last patient. The total duration of the study for Stage 1 is expected to be approximately 51 months. If the iDMC recommends to expand enrollment (Stage 2), the duration of the study may increase to approximately 74 months.

The Sponsor has the right to terminate this study, including long-term follow-up, at any time (e.g., if emerging safety signals indicate a potential health hazard to patients).

3.3 RATIONALE FOR STUDY DESIGN

3.3.1 Rationale for Atezolizumab Dose and Schedule

Atezolizumab will be administered at a fixed dose of 840 mg Q2W by IV infusion in combination with nab-pac–AC chemotherapy to align with the chemotherapy schedule (Weeks 1–20). For patients randomized to receive atezolizumab, the atezolizumab dosing regimen will be switched to 1200 mg Q3W for 11 doses after the completion of chemotherapy and surgery; the total duration of atezolizumab treatment will be approximately 12 months.

"Use of this document is governed by the terms of use on the first page of this document."

The average atezolizumab exposure following the 840 mg Q2W dosage is expected to be similar to that of 1200 mg Q3W, the approved dosage for atezolizumab (Tecentriq™ U.S. Package Insert). Anti-tumor activity has been observed across doses ranging from 1 mg/kg to 20 mg/kg Q3W. In Study PCD4989g, the maximum tolerated dose of atezolizumab was not reached and no dose-limiting toxicities were observed at any dose. The fixed dose of 1200 mg Q3W (equivalent to an average body weight–based dose of 15 mg/kg Q3W) was selected on the basis of both nonclinical studies (Deng et al. 2016) and available clinical pharmacokinetic, efficacy, and safety data (refer to the Atezolizumab Investigator's Brochure for details). Based on the understanding of population pharmacokinetic variability and atezolizumab concentration–tumor dynamic relationship, 840 mg Q2W and 1200 mg Q3W are expected to have comparable efficacy and safety profiles.

3.3.2 Rationale for Patient Population and Analysis Groups

Stage 1 of this study will enroll patients with cT2–cT4d operable TNBC, regardless of PD-L1 expression (Sections 4.1.1 and 4.1.2).

TNBC will be defined by prospective central laboratory assessment using ISH or IHC assays for HER2 assessment and IHC assays for ER/PgR assessment per ASCO-CAP guidelines (Hammond et al. 2010; Wolff et al. 2013).

Patients with cT2–cT4d TNBC were selected for this study because increased primary tumor size has been identified as poor prognostic variables and has been associated with decreased disease-free survival (DFS) and increased likelihood of early metastatic disease in patients with TNBC (Pistelli et al. 2013; Rosa Mendoza et al. 2013). Not-yet-published internal analyses from the California Cancer Registry found survival rates in patients with Stage II or III disease to be significantly lower than those of patients diagnosed with Stage I disease (3-year OS rate of 94.2% [95% CI: 93.3, 95.0] for Stage I; 86.2% [95%CI: 85.1, 87.2] for Stage II; and 58.8% [95%CI: 56.3, 61.2] for Stage III; data on file). By selecting a patient population that has an increased rate of disease recurrence (larger tumor size) and poorer clinical outcomes and by further enriching this with patients with node-positive disease, the study will enroll patients with TNBC who have the highest unmet medical need.

3.3.3 Rationale for Comparator of Nab-Paclitaxel followed by Doxorubicin and Cyclophosphamide

Multi-agent chemotherapy regimens have proven beneficial as neoadjuvant/adjuvant therapy for early stage breast cancer, improving both disease-specific and OS outcomes (Berry et al. 2006; NCCN 2016). The most effective combination regimens include anthracyclines, topoisomerase II inhibitors, cyclophosphamide, and taxanes (Peto et al. 2012; Early Breast Cancer Trialists' Collaborative Group 2005). Studies looking at optimizing the dose and schedule of early breast cancer chemotherapy regimens (Citron et al. 2003; Sparano et al. 2008) have established one of the optimal adjuvant regimens as doxorubicin 60 mg/m^2 and cyclophosphamide 600 mg/m^2 administered

"Use of this document is governed by the terms of use on the first page of this document."

Q2W for 4 cycles, followed by QW paclitaxel 80 mg/m^2 for 12 weeks with respect to maximizing efficacy and is included as a preferred option in international guidelines (Senkus et al. 2015; NCCN 2016).

Specifically, in the neoadjuvant setting, anthracycline/taxane chemotherapy-based regimens have routinely been used in TNBC, resulting in reported pCR rates of 41%–48% (Sparano et al. 2008; Sikov et al. 2015; Untch et al. 2016). Three-year EFS rates of 74%–76% have been reported for patients with TNBC who have received neoadjuvant anthracycline/taxane therapy (von Minckwitz et al. 2014; Sikov et al. 2015), leaving room for improvement in outcomes.

The safety of atezolizumab in combination with anthracyclines, cyclophosphamide, and other chemotherapeutic agents is being explored in Study BO29563, a Phase Ib/II study evaluating atezolizumab in combination with either obinutuzumab (G) + bendamustine or obinutuzumab (G) + cyclophosphamide, doxorubicin, vincristine, and prednisone (CHOP) in patients with follicular lymphoma or rituximab + CHOP in patients with diffuse large B-cell lymphoma. The CHOP regimen consists of cyclophosphamide 750 mg/m^2 on Day 1, doxorubicin 50 mg/m^2 on Day 1, vincristine 1.4 mg/m^2 (max. 2 mg) on Day 1, and prednisone 40 mg/m^2 on Days 1–5. During the safety run-in phase, patients received 6 cycles of induction treatment with either Atezo-G-benda or Atezo-G-CHOP. As of 19 July 2016, preliminary data from 7 patients who completed at least 3 cycles of induction treatment indicated that the combination of atezolizumab with G-CHOP was well tolerated. None of the stopping criteria defined for the safety run-in phase of the study were met. Two events of Grade 1 palpitation (1 related, 1 unrelated) were reported; no other cardiac events were reported. There were no treatment-related Grade 3 and Grade 4 adverse events during Cycles 2 and 3 (i.e., the safety observation window). No treatment delays or treatment discontinuations due to adverse events were observed.

For additional information on the safety of atezolizumab in combination with other agents, please refer to the Atezolizumab Investigator’s Brochure.

Nab-Paclitaxel as Neoadjuvant Treatment of Early TNBC

Nab-paclitaxel represents the preferred chemotherapy for combination with immunomodulatory drugs such as atezolizumab because of both 1) the opportunity to avoid immunosuppressive effects from the concurrent steroid use required with other taxanes (Gradishar et al. 2005) and 2) the high rate of tumor cell death achieved by nab-paclitaxel, which can be expected to expose the immune system to high levels of tumor antigens, thereby enhancing the degree and duration of cancer-specific T-cell immunity by inhibition of PD-L1–mediated immune suppression by atezolizumab. Nab-paclitaxel appears to be at least as active as the commonly used paclitaxel in early breast cancer (Untch et al. 2016; Gianni et al. 2016), and data from Study GP28328 suggests that the combination of nab-paclitaxel and atezolizumab is safe and efficacious (Section 1.3 and Adams et al. 2016). Identical chemotherapy backbone treatments were

"Use of this document is governed by the terms of use on the first page of this document."

selected for both the investigational and control arms of the study in order to isolate the effect of atezolizumab upon study endpoints.

The use of nab-paclitaxel in the neoadjuvant setting is supported by two randomized Phase III trials.

The Phase III GeparSepto study ($n = 1206$) compared the efficacy of a regimen of weekly nab-paclitaxel versus paclitaxel followed by epirubicin and cyclophosphamide as neoadjuvant therapy for EBC (Untch et al. 2016). The pCR rate among the cohort of patients with TNBC ($n = 276$) who received nab-paclitaxel was twice that of the women who received paclitaxel (48% vs. 26%; $p = 0.00027$). In the overall patient population, the incidence of Grade 3 or 4 anemia (2% nab-paclitaxel vs. 1% paclitaxel; $p = 0.048$) and peripheral sensory neuropathy Grade 3 or 4 (10% nab-paclitaxel [8% in those receiving 125 mg/m^2 dose] vs. 3% paclitaxel, $p < 0.001$) was significantly higher for nab-paclitaxel. Overall, 26% of patients had 1 or more serious adverse events in the nab-paclitaxel group compared with 21% of patients in the paclitaxel group ($p = 0.057$). The taxane dose was reduced in 30% of patients in the nab-paclitaxel group versus 12% in the paclitaxel group ($p < 0.0001$).

Results were also reported from a separate Phase III study comparing the efficacy of neoadjuvant nab-paclitaxel versus paclitaxel, both followed by anthracycline regimens in 695 women with HER2-negative high-risk breast cancer (Gianni et al. 2016). In the TNBC cohort ($n = 219$), 41.3% (95% CI: 31.9, 51.1) of patients who received the nab-paclitaxel–containing regimen achieved a pCR compared with 35% (95% CI: 26.6, 45.1) of patients who received the paclitaxel-containing regimen ($p = 0.376$; Gianni et al. 2016). The incidence of peripheral neuropathy was 62.6% in the nab-paclitaxel arm versus 53.7% in the paclitaxel arm. Neutrophil count decrease was 41.8% in the nab-paclitaxel arm versus 36.4% in the paclitaxel arm.

When combined, these studies suggest that nab-paclitaxel is at least as effective as paclitaxel; however, long term outcomes from these studies have not yet been reported.

Sequence of Neoadjuvant Chemotherapy

In Study WO39392, neoadjuvant nab-paclitaxel will be administered prior to doxorubicin + cyclophosphamide (AC) chemotherapy. This sequence of chemotherapy agents was selected in order to maximize the potential for a robust, initial immune-response as there are less reports of neutropenia and lower usage of steroids with a nab-paclitaxel compared to AC. In addition, biomarker analyses from Phase I atezolizumab studies have demonstrated activated CD8+ T cell proliferation and the presence of IL-18 peaks three weeks after the first-dose of atezolizumab (Herbst et al. 2014). The combination with nab-paclitaxel, as assessed in Study GP28328 (Adams et al. 2016) has a similar effect, suggesting nab-paclitaxel does not hamper atezolizumab activity and, therefore, may not negatively impact efficacy. The combination of AC and atezolizumab following atezolizumab-nab-paclitaxel has not

"Use of this document is governed by the terms of use on the first page of this document."

yet been investigated and the net impact on immune-response is unknown.
Data supporting the chemotherapy sequence come from several small prospective and retrospective studies that have evaluated the impact of changing the sequence of administration for anthracyclines and taxanes in the neoadjuvant setting for TNBC. While the endpoints measured have varied, these studies demonstrated that reversing the sequence of neoadjuvant breast cancer chemotherapy by starting with the taxane, followed by an anthracycline-containing regimen, is at least as effective as the conventional sequence, with the majority of studies demonstrating no statistically significant differences in clinical outcomes between the two sequences (Sledge et al. 2003; Bines et al. 2014; Earl et al. 2014).

Dose and Schedule of Nab-Paclitaxel

The dose and schedule of nab-paclitaxel (125mg/m^2 QW × 12 cycles) was selected based upon the dosing regimen used in the Phase III GeparSepto study where weekly nab-paclitaxel was found to be superior to weekly paclitaxel in the neoadjuvant setting (described above; Untch et al. 2016)

3.3.4 Rationale for Pathologic Complete Response as Primary Endpoint

The primary objective of Study WO39392 is to evaluate the efficacy of neoadjuvant treatment with atezolizumab + nab-pac–AC compared with placebo + nab-pac–AC in patients with newly diagnosed TNBC as measured by pCR.

pCR was selected as the primary efficacy endpoint; it is a validated, meaningful measure of response to therapy, and on the basis of data from several analyses and clinical trials and meta-analyses, there is an association between the pCR status of a patient and long-term outcomes (Cortazar et al. 2014; Liedtke et al. 2008; von Minckwitz et al. 2012). This association was especially strong in patients with TNBC (EFS hazard ratio [HR] 0.24 [95% CI: 0.18, 0.33]; OS HR 0.16 [95% CI: 0.11, 0.25]) (Cortazar et al. 2014).

pCR will be defined as the absence of residual invasive cancer on hematoxylin and eosin evaluation of the complete resected breast specimen and all sampled regional lymph nodes following completion of neoadjuvant systemic therapy (NAST), in line with the U.S. Food and Drug Administration (FDA) and European Medicines Agency guidance for industry on pCR endpoints (i.e., ypT0/Tis ypN0 in the current AJCC staging system).

3.3.5 Rationale for Biomarker Assessments

Published results suggest that the expression of PD-L1 in tumors correlates with response to anti–PD-1 and anti–PD-L1 therapy (Topalian et al. 2012; Herbst et al. 2014; Borghaei et al. 2015; Fehrenbacher et al. 2016; Herbst et al. 2016; Rosenberg et al. 2016). In the current study, baseline tumor specimens will be collected from patients and tested for PD-L1 expression by a central laboratory during the screening period. Randomization for patients enrolled in Stage 1, (as well as Stage 2 only if all-comer

"Use of this document is governed by the terms of use on the first page of this document."

patients are enrolled), will be stratified by PD-L1 expression as assessed by IHC. In addition to the assessment of PD-L1 status, other exploratory biomarkers potentially related to clinical benefit of atezolizumab, tumor immunobiology, mechanisms of resistance, or tumor type, may be analyzed.

Certain chemotherapies have been shown to be immunomodulatory (Pfirschke et al. 2016). To evaluate the impact of nab-paclitaxel and atezolizumab in the tumor immune microenvironment, optional on-treatment biopsies will be collected 2 weeks after treatment initiation (Day 15). The pharmacodynamic changes in the tumor microenvironment will be evaluated by immunostaining, hematoxylin and eosin evaluation (tumor-infiltrating lymphocytes), and gene expression.

To evaluate mechanisms of resistance to the combination, the residual tumor tissue post-surgery will be evaluated for biomarkers related to tumor and immune biology by gene expression as well as by immunostaining methodologies.

Patients will undergo mandatory tumor biopsy sample collection, if deemed clinically feasible by the investigator, at the time of first evidence of radiographic disease recurrence to evaluate tumor tissue biomarkers related to resistance, disease recurrence, and clinical benefit of atezolizumab.

Blood samples will be collected at baseline and during the study to evaluate changes in biomarkers. Changes in biomarkers such as cytokines associated with T-cell activation, circulating tumor DNA concentration, and T-cell subpopulations may provide evidence of biologic activity of atezolizumab in humans. Correlations between these biomarkers and safety and efficacy endpoints may be explored to identify blood-based biomarkers that might predict which patients are more likely to benefit from atezolizumab.

Tumor and blood samples collected at baseline and, if deemed clinically feasible by the investigator, tumor tissue collected at the time of disease recurrence will be analyzed through use of whole genome sequencing (WGS) and/or next-generation sequencing (NGS) to identify somatic mutations that are predictive of response to study treatment, are associated with recurrence to a more severe disease state, are associated with acquired resistance to study treatment, or can increase the knowledge and understanding of disease biology.

Genomics is increasingly informing researchers' understanding of disease pathobiology. WGS provides a comprehensive characterization of the genome and, along with clinical data collected in this study, may increase the opportunity for developing new therapeutic approaches. Data will be analyzed in the context of this study but will also be explored in aggregate with data from other studies. The availability of a larger dataset will assist in identification of important pathways, guiding the development of new targeted agents.

"Use of this document is governed by the terms of use on the first page of this document."

3.3.6 Rationale for Patient-Reported Outcome Assessments

EBC is largely asymptomatic, with the majority of newly diagnosed patients exhibiting no disease-specific, discernable symptoms (Barrett et al. 2009; Ryerson et al. 2015). Therefore, toxicities, the corresponding treatment-related symptoms, and their impact define the patient experience (i.e., how patients feel and function). These are important aspects to consider to help inform on the overall clinical benefit of a novel drug for this potentially curable indication. Treatment-related symptoms associated with EBC regimens can have significant impact on patient's lives, including their ability to conduct activities of daily living; on physical functioning, and on emotional and social aspects (Petersen et al. 2015). It is therefore critical to document the burden associated with EBC treatment and understand the experience of treatment-related symptoms and their impact directly from patients to further inform the benefit-risk assessment and treatment decision-making (Montazeri 2008; Au et al. 2010; FDA 2013; PDUFA Breast Cancer Workshop 2015; EMA 2016).

A comprehensive assessment of treatment burden from the patient's perspective in this study will be conducted and will include a global assessment of the impact of treatment on patients' functioning (role, physical) and health-related quality of life (HRQoL) as secondary endpoints, as well as the experience of treatment-related symptoms and their associated level of bother and the impact on emotional and social functioning as exploratory endpoints. The GHS/HRQoL, functional, and disease/treatment-related symptom items and scales of the European Organisation for the Research and Treatment of Cancer Quality of Life Questionnaire Core 30 (EORTC QLQ-C30; see Appendix 5), and the treatment bother item GP5 from the Functional Assessment of Cancer Therapy-General (FACT-G; see Appendix 6) Quality of Life Instrument will all be used to assess patients' treatment burden (see Figure 2).

"Use of this document is governed by the terms of use on the first page of this document."

Figure 2 Documenting Treatment Burden in Early Breast Cancer Patients

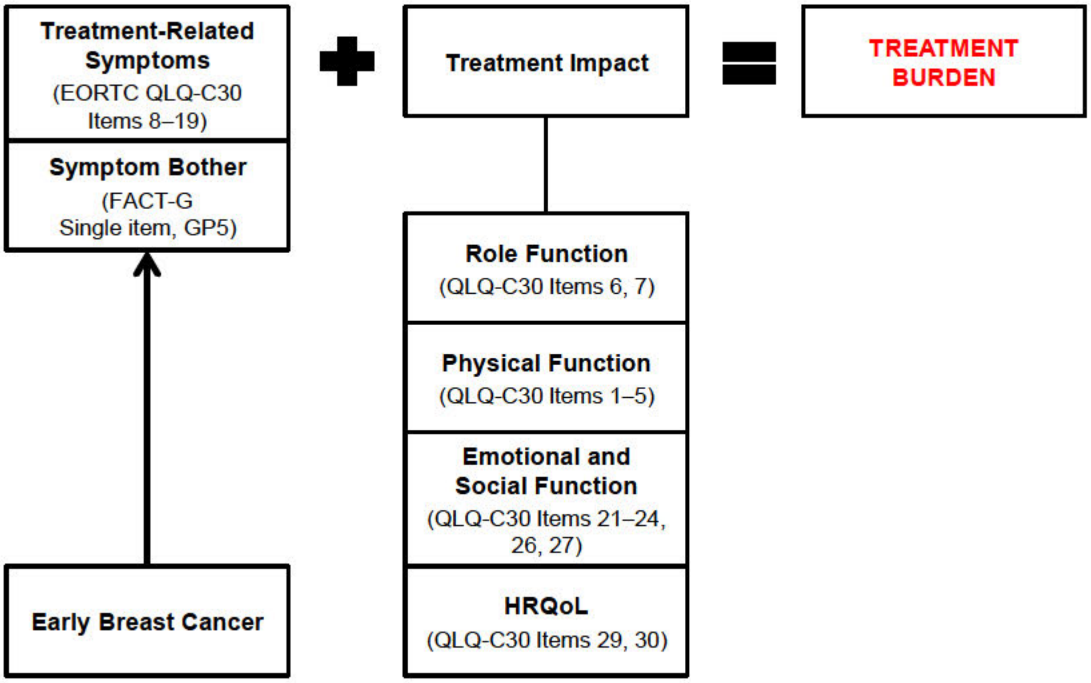

EORTC = European Organisation for Research and Treatment of Cancer; FACT-G = Functional Assessment of Cancer Therapy-General; HRQoL = health-related quality of life; QLQ-C30 = Quality of Life Questionnaire Core 30.

Given the duration of treatment and the potential for long-term treatment impact, all PRO measures will be assessed at each cycle and at specified timepoints after treatment discontinuation as defined in the schedule of activities (Appendix 1). All PRO data collected will be analyzed per published scoring manuals (Cella 1997; Fayers et al. 2001) to support and inform the benefit-risk assessment of atezolizumab therapy.

4. MATERIALS AND METHODS

4.1 PATIENTS

Approximately 204 patients will be enrolled in this study at Stage 1 and approximately 120 additional patients will be enrolled if the iDMC recommends to expand to Stage 2. Enrollment will take place at approximately 75 global sites.

4.1.1 Inclusion Criteria

Patients must meet the following criteria for study entry:

- Signed Informed Consent Form
- Ability to comply with protocol, in the investigator's judgment
- Women or men aged ≥ 18 years

"Use of this document is governed by the terms of use on the first page of this document."

- ECOG performance status of 0 or 1
- Histologically documented TNBC (negative HER2, ER, and PgR status); HER2 negativity will be defined by central laboratory assessment using ISH or IHC assays per ASCO/CAP criteria (Wolff et al. 2013) and ER/PgR negativity will be defined by central laboratory assessment using IHC per ASCO/CAP criteria (Hammond et al. 2010). Central laboratory assessment will occur prior to randomization.

  Patients with multifocal tumors (more than one tumor confined to the same quadrant as the primary tumor) are eligible provided all discrete lesions are sampled and centrally confirmed as TNBC.

- Confirmed tumor PD-L1 evaluation as documented through central testing of a representative tumor tissue specimen.

  In Stage 2, if the iDMC recommendation is to expand to a PD-L1–positive population, only patients with confirmed tumor PD-L1 positive (IC1/2/3) will be considered eligible.

- Primary breast tumor size of > 2 cm by at least one radiographic or clinical measurement
- Stage at presentation: cT2–cT4, cN0–cN3, cM0
- Patient agreement to undergo appropriate surgical management including axillary lymph node surgery and partial or total mastectomy after completion of neoadjuvant treatment
- Baseline LVEF ≥ 53% measured by echocardiogram (ECHO) or multiple-gated acquisition (MUGA) scans
- Adequate hematologic and end-organ function, as defined by the following laboratory results obtained within 14 days prior to the first study treatment:
  - ANC ≥ 1500 cells/μL (without granulocyte colony–stimulating factor [G-CSF] support within 2 weeks prior to Cycle 1, Day 1)
  - Lymphocyte count ≥ 500 cells/μL
  - Platelet count ≥ 100,000 cells/μL (without transfusion within 2 weeks prior to Cycle 1, Day 1)
  - Hemoglobin ≥ 9.0 g/dL
  - AST, ALT, and alkaline phosphatase ≤ 2.5 × the upper limit of normal (ULN)
  - Serum bilirubin ≤ 1.0 × ULN

    Patients with known Gilbert syndrome who have serum bilirubin level ≤ 3 × ULN may be enrolled.

  - For patients not receiving therapeutic anticoagulation: INR or aPTT ≤ 1.5 × ULN within 14 days prior to initiation of study treatment
  - For patients receiving therapeutic anticoagulation: stable anticoagulant regimen and stable INR during the 14 days immediately preceding initiation of study treatment

"Use of this document is governed by the terms of use on the first page of this document."

- Creatinine clearance ≥ 30 mL/min (calculated using the Cockcroft-Gault formula)
- Serum albumin ≥ 25 g/L (≥ 2.5 g/dL)

- Representative formalin-fixed, paraffin-embedded (FFPE) tumor specimen in paraffin blocks (preferred) or at least 20 unstained slides, with an associated pathology report documenting ER, PgR, and HER2 negativity

  Tumor tissue should be of good quality based on total and viable tumor content and must be evaluated for PD-L1 expression prior to enrollment. Patients whose tumor tissue is not evaluable for PD-L1 expression are not eligible.

  If multiple tumor specimens are submitted, patients may be eligible if at least one specimen is evaluable for PD-L1. For the purpose of stratification, the PD-L1 score of the patient will be the maximum PD-L1 score among the samples.

    In Stage 2, if the recommendation from the iDMC is to expand to a PD-L1–positive population, no further stratification based on PD-L1 status will be conducted.

    Acceptable samples include core-needle biopsies for deep tumor tissue (minimum three cores) or excisional, incisional, punch, or forceps biopsies for cutaneous, subcutaneous, or mucosal lesions.

    Fine-needle aspiration, brushing, and cell pellet from cytology specimens are not acceptable.

- For women of childbearing potential: agreement to remain abstinent (refrain from heterosexual intercourse) or use contraceptive methods, and agreement to refrain from donating eggs, as defined below:

  Women must remain abstinent or use contraceptive methods that result in a failure rate of < 1% per year during the treatment period and for at least 5 months after the last dose of atezolizumab, or 1 month after the last dose of nab-paclitaxel, or 6 months after the last dose of doxorubicin, or 12 months after the last dose of cyclophosphamide, whichever is later. Women must refrain from donating eggs during this same period.

  A woman is considered to be of childbearing potential if she is postmenarcheal, has not reached a postmenopausal state (≥ 12 continuous months of amenorrhea with no identified cause other than menopause), and has not undergone surgical sterilization (removal of ovaries and/or uterus).

  Examples of contraceptive methods with a failure rate of < 1% per year, when used consistently and correctly, include combined (estrogen and progestogen containing) hormonal contraception associated with inhibition of ovulation, progestogen-only hormonal contraception associated with inhibition of ovulation, bilateral tubal occlusion; male sterilization; intrauterine devices; intrauterine hormone-releasing system; and sexual abstinence.

"Use of this document is governed by the terms of use on the first page of this document."

The reliability of sexual abstinence should be evaluated in relation to the duration of the clinical study and the preferred and usual lifestyle of the patient. Periodic abstinence (e.g., calendar, ovulation, symptothermal, or postovulation methods) and withdrawal are not acceptable methods of contraception.

- For men: agreement to remain abstinent (refrain from heterosexual intercourse) or use contraceptive measures and agreement to refrain from donating sperm, as defined below:

  With female partners of childbearing potential, men must remain abstinent or use a condom plus an additional contraceptive method that together result in a failure rate of < 1% per year during the treatment period and for 6 months after the last dose of nab-paclitaxel, cyclophosphamide, or doxorubicin. Men must refrain from donating sperm during this same period.

  With pregnant female partners, men must remain abstinent or use a condom during the treatment period and for 6 months after the last dose of nab-paclitaxel, cyclophosphamide, or doxorubicin to avoid exposing the embryo.

  The reliability of sexual abstinence should be evaluated in relation to the duration of the clinical trial and the preferred and usual lifestyle of the patient. Periodic abstinence (e.g., calendar, ovulation, symptothermal, or postovulation methods) and withdrawal are not acceptable methods of contraception.

- Women who are not postmenopausal (≥ 12 months of non–therapy-induced amenorrhea) or have undergone a sterilization procedure must have a negative serum pregnancy test result within 14 days prior to initiation of study drug
- Willingness and ability to comply with scheduled visits, treatment plans, laboratory tests, and other study procedures, including the completion of PRO questionnaires

4.1.2 Exclusion Criteria

Patients who meet any of the following criteria will be excluded from study entry:

- Prior history of invasive breast cancer
- Stage IV (metastatic) breast cancer
- Prior systemic therapy for treatment and prevention of breast cancer
- Previous therapy with anthracyclines or taxanes for any malignancy
- History of ductal carcinoma in situ (DCIS), except for patients treated exclusively with mastectomy > 5 years prior to diagnosis of current breast cancer
- History of pleomorphic lobular carcinoma in situ (LCIS), except for patients surgically managed > 5 years prior to diagnosis of current breast cancer (note that patients with non-pleomorphic LCIS [either untreated or treated with surgery] are allowed)
- Bilateral breast cancer

"Use of this document is governed by the terms of use on the first page of this document."

- Undergone incisional and/or excisional biopsy of primary tumor and/or axillary lymph nodes. Patients who have undergone SLNB at the baseline may be eligible only if the SLNB was free of invasive carcinoma. Any patient with a positive SLN (involved with invasive carcinoma) is ineligible to participate in this study.
- Axillary lymph node dissection prior to initiation of neoadjuvant therapy
- History of other malignancy within 5 years prior to screening, with the exception of those with a negligible risk of metastasis or death (e.g., 5-year OS of > 90%), such as adequately treated carcinoma in situ of the cervix, non-melanoma skin carcinoma, localized prostate cancer, or Stage I uterine cancer
- History of cerebrovascular accident within 12 months prior to randomization
- Cardiopulmonary dysfunction as defined by any of the following prior to randomization:
  - History of NCI CTCAE v4.0 Grade ≥ 3 symptomatic congestive heart failure or New York Heart Association (NYHA) criteria Class ≥ II
  - Angina pectoris requiring anti-anginal medication, serious cardiac arrhythmia not controlled by adequate medication, severe conduction abnormality, or clinically significant valvular disease
  - High-risk uncontrolled arrhythmias (i.e., atrial tachycardia with a heart rate > 100/min at rest, significant ventricular arrhythmia [ventricular tachycardia], or higher-grade atrioventricular [AV]-block [second-degree AV-block Type 2 [Mobitz 2] or third degree AV-block])
  - Significant symptoms (Grade ≥ 2) relating to left ventricular dysfunction, cardiac arrhythmia, or cardiac ischemia
  - Myocardial infarction within 12 months prior to randomization
  - Uncontrolled hypertension (systolic blood pressure > 180 mmHg and/or diastolic blood pressure > 100 mmHg)
  - Evidence of transmural infarction on ECG
  - Requirement for oxygen therapy
- History of severe allergic, anaphylactic, or other hypersensitivity reactions to chimeric or humanized antibodies or fusion proteins
- Known hypersensitivity to biopharmaceuticals produced in Chinese hamster ovary cells
- Known allergy or hypersensitivity to the components of the atezolizumab formulation
- Known allergy or hypersensitivity to the components of the nab-paclitaxel, cyclophosphamide, or doxorubicin formulations
- Known allergy or hypersensitivity to filgrastim or pegfilgrastim formulations

"Use of this document is governed by the terms of use on the first page of this document."

- Active or history of autoimmune disease or immune deficiency, including but not limited to myasthenia gravis, myositis, autoimmune hepatitis, systemic lupus erythematosus, rheumatoid arthritis, inflammatory bowel disease, antiphospholipid syndrome, Wegener granulomatosis, Sjögren syndrome, Guillain-Barré syndrome, or multiple sclerosis (see Appendix 8 for a more comprehensive list of autoimmune diseases and immune deficiencies) with the following exceptions:

  Patients with a history of autoimmune-related hypothyroidism on a stable dose of thyroid replacement hormone may be eligible for this study.

  Patients with controlled Type I diabetes mellitus on a stable dose of insulin regimen may be eligible for this study.

  Patients with eczema, psoriasis, lichen simplex chronicus, or vitiligo with dermatologic manifestations only (e.g., patients with psoriatic arthritis are excluded) are permitted provided all of following conditions are met:

  – Rash must cover $<$10% of body surface area

  – Disease is well controlled at baseline and requires only low-potency topical corticosteroids

  – No occurrence of acute exacerbations of the underlying condition requiring psoralen plus ultraviolet A radiation, methotrexate, retinoids, biologic agents, oral calcineurin inhibitors, or high-potency or oral corticosteroids within the previous 12 months

- History of idiopathic pulmonary fibrosis, organizing pneumonia (e.g., bronchiolitis obliterans), drug-induced pneumonitis, idiopathic pneumonitis, or evidence of active pneumonitis on screening chest computed tomography (CT) scan

  History of radiation pneumonitis in the radiation field (fibrosis) is permitted.

- Positive HIV test at screening

- Active hepatitis B virus (HBV) infection, defined as having a positive hepatitis B surface antigen (HBsAg) test at screening

  Patients with a past or resolved HBV infection, defined as having a negative HBsAg test and a positive total hepatitis B core antibody (HBcAb) test at screening, are eligible for the study if active HBV infection is ruled out on the basis of HBV DNA viral load per local guidelines.

- Active hepatitis C virus (HCV) infection, defined as having a positive HCV antibody test at screening

  Patients who have a positive HCV antibody test are eligible for the study if a polymerase chain reaction (PCR) assay is negative for HCV RNA.

- Active tuberculosis

- Severe infections within 4 weeks prior to initiation of study treatment, including but not limited to hospitalization for complications of infection, bacteremia, or severe pneumonia

"Use of this document is governed by the terms of use on the first page of this document."

- Treatment with therapeutic oral or IV antibiotics within 2 weeks prior to initiation of study treatment

  Patients receiving prophylactic antibiotics (e.g., for prevention of a urinary tract infection or to prevent chronic obstructive pulmonary disease exacerbation) are eligible for the study.

- Major surgical procedure within 4 weeks prior to initiation of study treatment or anticipation of need for a major surgical procedure (other than anticipated breast surgery) during the course of the study
- Prior allogeneic stem cell or solid organ transplantation
- Administration of a live attenuated vaccine within 4 weeks prior to initiation of study treatment or anticipation of need for such a vaccine during the atezolizumab/placebo treatment or within 5 months after the last dose of atezolizumab/placebo

  Patients must agree not to receive live, attenuated influenza vaccine (e.g., FluMist®) within 4 weeks prior to randomization, during treatment or within 5 months following the last dose of atezolizumab (for patients randomized to atezolizumab).

- Any other disease, metabolic dysfunction, physical examination finding, or clinical laboratory finding giving reasonable suspicion of a disease or condition that contraindicates the use of an investigational drug or that may affect the interpretation of the results or render the patient at high risk from treatment complications
- Prior treatment with CD137 agonists or immune checkpoint–blockade therapies, including anti-CD40, anti–CTLA-4, anti–PD-1, and anti–PD-L1 therapeutic antibodies
- Treatment with systemic immunostimulatory agents (including but not limited to interferons, IL-2) within 4 weeks or 5 half-lives of the drug, whichever is longer, prior to initiation of study treatment
- Treatment with systemic immunosuppressive medications (including but not limited to prednisone, cyclophosphamide, azathioprine, methotrexate, thalidomide, and anti–tumor necrosis [anti-TNF] factor agents) within 2 weeks prior to initiation of study treatment or anticipation of need for systemic immunosuppressive medications during the study

  Patients who have received acute, low-dose, systemic immunosuppressant medications (e.g., a one-time dose of dexamethasone for nausea) may be enrolled in the study after discussion with and approval by the Medical Monitor.

  The use of inhaled corticosteroids and mineralocorticoids (e.g., fludrocortisone for adrenal insufficiency) is allowed.

- Pregnant or lactating, or intending to become pregnant during the study

  Women of childbearing potential must have a negative serum pregnancy test result within 14 days prior to initiation of study treatment.

"Use of this document is governed by the terms of use on the first page of this document."

4.2 METHOD OF TREATMENT ASSIGNMENT AND BLINDING

This is a double-blind, randomized, placebo-controlled study.

After written informed consent has been obtained, all screening procedures and assessments have been completed, and eligibility has been established, the study site will enter demographic and baseline characteristics in the interactive voice or web-based response system (IxRS). For those patients who are eligible for enrollment, the study site will obtain the patient's identification number and treatment assignment from the IxRS.

Randomization will occur in a 1:1 ratio using a permuted-block randomization method. Patients will be randomized to one of two treatment arms: atezolizumab + nab-pac–AC or placebo + nab-pac–AC. The randomization scheme is designed to ensure that an approximately equal number of patients will be enrolled in each treatment arm within the categories defined for the following stratification factors at baseline:

- AJCC stage at diagnosis (II vs. III)
- Tumor PD-L1 status (IC0 vs. IC1/2/3) for patients enrolled in Stage 1 and Stage 2 (only if the study continues to enroll all-comer patients)

Patients should receive their first dose of study treatment on the day of randomization if possible. If treatment is not possible, the first dose should occur no later than 14 days after randomization.

The Sponsor and its agents (with the exception of the IxRS service provider [the external independent statistical coordinating center responsible for verifying patient randomization and study treatment kit assignments], pharmacokinetic/pharmacodynamic [PK/PD] laboratory personnel, and the iDMC members); the study site personnel, including the investigator; and the patient will be blinded to treatment assignment prior to unblinding of treatment assignment as detailed below.

The Sponsor and its agents (with the exception of the PD-L1 assay provider and the iDMC members); the study site personnel, including the investigators; and the patients will be blinded to PD-L1 status until all data have been cleaned and verified, and the database has been locked for the primary analysis for pCR or the interim analysis for pCR at the end of Stage 1.

If the iDMC recommends to progress to Stage 2 in all-comer population, the Sponsor and its agents, the study site personnel, including the investigators, and the patients will remain blinded to PD-L1 status until all data have been cleaned and verified, and the database has been locked for the primary analysis of pCR at the end of Stage 2.

"Use of this document is governed by the terms of use on the first page of this document."

If the iDMC recommends to progress to Stage 2 in PD-L1−positive population, the Sponsor and its agents, the study site personnel, including the investigators, and the patients will be aware of the PD-L1 status of patients in screening for Stage 2, so that a patient's eligibility for enrollment can be assessed.

If the iDMC recommends to progress to Stage 2 in PD-L1−positive population, the Sponsor and its agents, the study site personnel, including the investigators, and the patients enrolled in Stage 1 may be informed about their PD-L1 status upon request by the investigator.

While PK samples must be collected from patients assigned to the control arm to maintain the blinding of treatment assignment, PK assay results for these patients are generally not needed for the safe conduct or proper interpretation of this study. Personnel who are responsible for performing PK assays and sample data reconciliation will be unblinded to patients' treatment assignments to identify appropriate PK samples to be analyzed and cleaned. Samples from patients assigned to the control arm will not be analyzed except by request (i.e., to evaluate a possible error in dosing).

Unblinding of Treatment Assignment

A patient's treatment assignment will be unblinded to the site personnel (including the investigator) after assessment of the patient's pCR status, in order to inform further treatment.

Otherwise, unblinding should occur only in exceptional circumstances when knowledge of the actual treatment is absolutely essential for further management of clinically significant adverse events experienced by the patient (i.e., emergency unblinding for safety reasons).

Per health authority reporting requirements, treatment assignment will be unblinded for serious, unexpected study drug-related toxicity (as part of the Investigational New Drug [IND] safety reporting process). In these instances, investigators will not be notified of individual patient's treatment assignment as a matter of course. Emergency unblinding by the investigator should be a last resort performed only in cases when knowledge of treatment assignment will affect the management of a patient who experiences a treatment-emergent adverse event. Investigators are encouraged to consult with the Medical Monitor prior to performing emergency unblinding. If unblinding is necessary for patient safety management, the investigator is credentialed to break the treatment code within the IxRS autonomously by means of a pin code, which is issued to them at the start of the study. All such occurrences should be documented in the study file.

Unblinding should not result in the withdrawal of a patient from the study. Every effort should be made to retain unblinded patients and continue data collection per protocol.

"Use of this document is governed by the terms of use on the first page of this document."

4.3 STUDY TREATMENT

The investigational medicinal products (IMPs) for this study are atezolizumab/placebo and nab-paclitaxel. Additionally, doxorubicin, cyclophosphamide, and filgrastim/pegfilgrastim will be administered as background treatment after nab-paclitaxel.

4.3.1 <u>Formulation, Packaging, and Handling</u>

4.3.1.1 Atezolizumab and Placebo

Atezolizumab will be supplied by the Sponsor as sterile liquid in 20-mL glass vials. The vial is designed to deliver 20 mL (1200 mg) of atezolizumab solution but may contain more than the stated volume to enable delivery of the entire 20-mL volume. Extraction of 14 mL of atezolizumab solution from a 1200-mg vial contains an 840-mg dose. For information on the formulation and handling of atezolizumab, refer to the pharmacy manual and the Atezolizumab Investigator's Brochure.

The placebo will be supplied by the Sponsor and will be identical in appearance to atezolizumab and comprise the same excipients but without atezolizumab drug product. The placebo should be handled, stored, and used in the same manner as atezolizumab.

4.3.1.2 Background Treatment

For information on the formulation, packaging, and handling of nab-paclitaxel, doxorubicin, cyclophosphamide, and filgrastim/pegfilgrastim, see the local prescribing information for the respective treatments as available.

Nab-paclitaxel will be supplied by the Sponsor.

4.3.2 <u>Dosage, Administration, and Compliance</u>

4.3.2.1 Atezolizumab or Placebo

Patients will receive atezolizumab 840 mg or placebo administered by IV infusion Q2W (14 [$\pm$3] days) for 20 weeks (i.e., 10 doses) in combination with nab-paclitaxel, doxorubicin, and cyclophosphamide chemotherapy. Atezolizumab or placebo should be infused over 60 minutes. If the first infusion is tolerated, subsequent infusions can be administered over 30 minutes. Postoperatively (i.e., following unblinding to treatment assignment as specified in Section 4.2), those randomized to the atezolizumab arm will continue to receive maintenance atezolizumab but at a different dose of 1200 mg administered by IV infusion Q3W for 11 doses, for a total of approximately 12 months of atezolizumab treatment; those randomized to the placebo arm will stop receiving placebo.

For more detailed information on drug preparation, storage, and administration, refer to the Atezolizumab Investigator’s Brochure and pharmacy manual.

Administration of atezolizumab or placebo will be performed in a setting with emergency medical facilities and staff who are trained to monitor for and respond to medical emergencies. For anaphylaxis precautions, see Appendix 9.

"Use of this document is governed by the terms of use on the first page of this document."

No premedication will be allowed for the first dose of atezolizumab or placebo. Patients who experience an infusion-related reaction (IRR) with the first infusion may receive premedication with antihistamines or antipyretics or analgesics (e.g., acetaminophen) for subsequent infusions.

The initial dose of atezolizumab or placebo will be delivered over 60 (±15) minutes. Subsequent infusions will be delivered over 30 (±10) minutes if the previous infusion was tolerated without infusion-associated adverse events, or 60 (±15) minutes if the patient experienced an infusion-associated adverse event with the previous infusion. Vital signs are to be measured before, as well as during, and after infusions, if clinically indicated, as outlined in Section 4.5.4.

Guidelines for medical management of IRRs are provided in Appendix 13.

No dose modification for atezolizumab is allowed. Guidelines for treatment interruption or discontinuation due to the development of an adverse event are provided in Appendix 13.

In case of unacceptable toxicity attributed to chemotherapy, atezolizumab/placebo should be held and restarted together with chemotherapy if there is no contraindication.

During the neoadjuvant period, if nab-paclitaxel chemotherapy is discontinued due to any reason (e.g., toxicity), patients can proceed to AC chemotherapy plus atezolizumab/placebo at the discretion of the investigator. If AC chemotherapy has to be discontinued for any reason (e.g., toxicity), the date of surgery can be brought forward and patients can proceed to surgery at the discretion of the investigator. In any of these scenarios all patients who discontinue planned study treatment and proceed to surgery will remain on study for pCR assessment and for follow-up of secondary and exploratory endpoints unless consent from study participation is withdrawn.

In case of toxicities attributed to atezolizumab/placebo that require a dose delay, chemotherapy should be continued independently of atezolizumab/placebo if there is no contraindication. Atezolizumab/placebo may be restarted when the conditions for retreatment have been met. When atezolizumab/placebo is restarted, the infusions should remain synchronized and aligned with the chemotherapy schedule.

Study treatment (including atezolizumab or placebo and chemotherapy, if applicable) will be discontinued in the event of disease progression or disease recurrence while patient is receiving therapy.

Any dose modification should be noted on the Study Drug Administration electronic Case Report Form (eCRF). Cases of accidental dose or medication error, along with any associated adverse events, should be reported as described in Section 5.4.3.4.

"Use of this document is governed by the terms of use on the first page of this document."

4.3.2.2 Background Treatment

Patients will receive nab-paclitaxel (125 mg/m^2) administered via IV infusion weekly for 12 weeks followed by doxorubicin (60 mg/m^2) + cyclophosphamide (600 mg/m^2) administered via IV infusion Q2W with filgrastim/pegfilgrastim support for 4 cycles (i.e., a total of 4 doses of doxorubicin and cyclophosphamide; see Figure 1). The dose of cyclophosphamide should be capped at 1200 mg.

Dose delays and dose reductions for toxicity are permitted. Guidelines for dosage modification and treatment interruption or discontinuation due to the development of an adverse event are provided in Section 5.1.5.

During the neoadjuvant period, if nab-paclitaxel chemotherapy is discontinued due to any reason (e.g., toxicity), patients can proceed to AC chemotherapy plus atezolizumab/placebo at the discretion of the investigator. If AC chemotherapy has to be discontinued for any reason (e.g., toxicity), the date of surgery can be brought forward and patients can proceed to surgery at the discretion of the investigator. In any of these scenarios, all patients who discontinue planned study treatment and proceed to surgery will remain on study for pCR assessment and for follow-up of secondary and exploratory endpoints unless consent from study participation is withdrawn.

Any overdose or incorrect administration of nab-paclitaxel, doxorubicin, or cyclophosphamide should be noted on the respective drug administration eCRF. Adverse events associated with an overdose or incorrect administration of nab-paclitaxel, doxorubicin, or cyclophosphamide should be recorded on the Adverse Event eCRF.

Nab-Paclitaxel

Nab-paclitaxel will be administered as an IV infusion given over 30 minutes. Nab-paclitaxel should be administered after atezolizumab or placebo. The dose of nab-paclitaxel will be 125 mg/m^2 administered via IV infusion over 30 minutes QW for 12 weeks. Doses of nab-paclitaxel should not be administered more frequently than every 7 days.

Sites should follow their institutional standard of care for determining the nab-paclitaxel dose adjustments in the event of patient weight changes. The infusion site should be closely monitored for possible infiltration during drug administration.

Refer to the local prescribing information for more details regarding the preparation and administration of nab-paclitaxel.

"Use of this document is governed by the terms of use on the first page of this document."

Doxorubicin

Doxorubicin should be given as an IV bolus over 3–5 minutes or as an IV infusion given over 15–30 minutes, in accordance with local standards of care. Doxorubicin and cyclophosphamide should be administered after atezolizumab or placebo. The dose of doxorubicin will be 60 mg/m^2 IV. Dose delays and reduction for toxicity are permitted. Doxorubicin will be administered Q2W for 4 doses (dose-dense AC) with filgrastim/pegfilgrastim support.

Refer to the local prescribing information for details regarding the preparation and administration of doxorubicin.

Cyclophosphamide

Cyclophosphamide should be given as an IV bolus over 3–5 minutes or as an IV infusion, in accordance with local standards of care. Doxorubicin and cyclophosphamide should be administered after atezolizumab or placebo. The dose of cyclophosphamide will be 600 mg/m^2 IV. The dose should be capped at 1200 mg. Dose delays and dose reductions for toxicity are permitted. Cyclophosphamide will be administered Q2W for 4 doses (dose-dense AC) with filgrastim/pegfilgrastim support. Note: Oral cyclophosphamide is not permitted.

Chemotherapy-induced nausea and vomiting prophylaxis and treatment should be administered as clinically indicated (see Section 4.4.2 for further guidance). Because systemic corticosteroids may attenuate the potential beneficial immunologic effects of treatment with atezolizumab, alternative agents should be considered when clinically feasible.

Refer to the local prescribing information for details regarding the preparation and administration of cyclophosphamide.

Premedications

In general, chemotherapy supportive care should be administered per ASCO, EORTC, or European Society for Medical Oncology (ESMO) guidelines or local standard of care. For further details regarding permitted therapies, see Section 4.4.1.

4.3.3 Investigational Medicinal Product Accountability

All IMPs required for completion of this study (atezolizumab and nab-paclitaxel) will be provided by the Sponsor where required by local practices.

The study site will acknowledge receipt of IMPs using the IxRS to confirm the shipment condition and content. Any damaged shipments will be replaced.

IMPs either will be disposed of at the study site according to the study site's institutional standard operating procedure or will be returned to the Sponsor with the appropriate documentation. The site's method of IMP destruction must be agreed to by the Sponsor.

"Use of this document is governed by the terms of use on the first page of this document."

The site must obtain written authorization from the Sponsor before any IMP is destroyed, and IMP destruction must be documented on the appropriate form.

Accurate records of all IMPs received at, dispensed from, returned to, and disposed of by the study site should be recorded on the Drug Inventory Log.

4.3.4 Post-Trial Access to Atezolizumab

Currently, the Sponsor does not have any plans to provide atezolizumab or any other study treatments or interventions to patients who have completed the study.
The Sponsor may evaluate whether to continue providing atezolizumab in accordance with the Roche Global Policy on Continued Access to Investigational Medicinal Product, available at the following Web site:

http://www.roche.com/policy_continued_access_to_investigational_medicines.pdf

4.4 CONCOMITANT THERAPY AND ADDITIONAL RESTRICTIONS

Concomitant therapy during the study includes any medication (e.g., prescription drugs, over-the-counter drugs, vaccines, herbal or homeopathic remedies, nutritional supplements) used by a patient in addition to protocol-mandated study treatment from 30 days prior to initiation of study treatment to the treatment discontinuation visit.
All such medications should be reported to the investigator and recorded on the Concomitant Medications eCRF.

4.4.1 Permitted Therapy

Patients are permitted to use the following therapies during the study:

- Oral contraceptives
- Prophylactic or therapeutic anticoagulation therapy (such as warfarin at a stable dose or low-molecular-weight heparin)
- Inactivated influenza vaccinations
- Megestrol administered as an appetite stimulant
- Inhaled corticosteroids (e.g., budesonide)
- Mineralocorticoids (e.g., fludrocortisone)
- Low-dose corticosteroids administered for orthostatic hypotension or adrenocortical insufficiency

Premedication with antihistamines may be administered for the second and subsequent atezolizumab infusions only, at the discretion of the investigator.

"Use of this document is governed by the terms of use on the first page of this document."

G-CSF (i.e., filgrastim or pegfilgrastim) treatment is permitted for patients receiving chemotherapy and is required during the doxorubicin/cyclophosphamide portion of chemotherapy. The primary prophylaxis should be administered per the ASCO, EORTC, or ESMO guidelines or per local standard practice; namely, in patients who are ≥60 years of age and/or with comorbidities (Smith et al. 2006; Crawford et al. 2009; Aapro et al. 2011).

Evidence supporting the use of long-acting (pegylated) forms of G-CSF in patients receiving weekly nab-paclitaxel is limited and investigators should consider giving preference to conventional formulations of G-CSF.

In general, investigators should manage a patient's care with supportive therapies as clinically indicated per local standard practice. Patients who experience infusion-associated symptoms may be treated symptomatically with acetaminophen, ibuprofen, diphenhydramine, and/or H_2-receptor antagonists (e.g., famotidine, cimetidine), or equivalent medications per local standard practice. Serious infusion-associated events manifested by dyspnea, hypotension, wheezing, bronchospasm, tachycardia, reduced oxygen saturation, or respiratory distress should be managed with supportive therapies as clinically indicated (e.g., supplemental oxygen and β_2-adrenergic agonists; see Appendix 9).

4.4.2 Cautionary Therapy

Systemic corticosteroids and TNF-α inhibitors may attenuate potential beneficial immunologic effects of treatment with atezolizumab. Therefore, in situations in which systemic corticosteroids or TNF-α inhibitors would be routinely administered, alternatives, including antihistamines, should be considered. If the alternatives are not feasible, systemic corticosteroids and TNF-α inhibitors may be administered at the discretion of the investigator.

Systemic corticosteroids are recommended, at the discretion of the investigator, for the treatment of specific *immune-mediated* adverse events when associated with atezolizumab or placebo therapy (refer to Appendix 13 for details).

Concomitant use of herbal therapies is not recommended because their pharmacokinetics, safety profiles, and potential drug-drug interactions are generally unknown. However, herbal therapies may be used during the study at the discretion of the investigator.

4.4.3 Potential Drug Interactions with Chemotherapy

The chemotherapeutic agents used in this study are associated with potential drug interactions. The metabolism of nab-paclitaxel is catalyzed by cytochrome P450 (CYP) isoenzymes CYP2C8 and CYP3A4. Doxorubicin is a major substrate of CYP3A4 and CYP2D6 and P-glycoprotein (P-gp). Cyclophosphamide is also activated by CYP.

"Use of this document is governed by the terms of use on the first page of this document."

Caution should be exercised when nab-paclitaxel is concomitantly administered with known inhibitors (e.g., atazanavir, clarithromycin, indinavir, itraconazole, ketoconazole, nefazodone, nelfinavir, ritonavir, saquinavir, and telithromycin); inducers (e.g., rifampin, phenobarbital, phenytoin, St. John's Wort and carbamazepine); and substrates (e.g., midazolam, buspirone, felodipine, lovastatin, eletriptan, sildenafil, simvastatin, and triazolam) of CYP3A4. Caution should also be exercised when nab-paclitaxel is concomitantly administered with known inhibitors (e.g., gemfibrozil), inducers (e.g., rifampin), and substrates (e.g., repaglinide and rosiglitazone) of CYP2C8.

Clinically significant interactions have been reported with inhibitors of CYP3A4, CYP2D6, and/or P-gp (e.g., verapamil). Caution should be exercised and concurrent use of doxorubicin with inhibitors and inducers of CYP3A4, CYP2D6, or P-gp should be avoided.

Similar cautions should be exercised with cyclophosphamide.

There is a moderate to high potential for drug-drug interactions with any medication that is metabolized by or strongly inhibits or induces these enzymes. Therefore, the medications listed above should be avoided when chemotherapy is being administered. If use of one of these medications is necessary, the risks and benefits should be discussed with the Medical Monitor prior to concomitant administration with chemotherapy.

The lists of medications shown above are not necessarily comprehensive. Thus, the investigator should consult the prescribing information for any concomitant medication as well as the Internet references provided below when determining whether a certain medication is metabolized by or strongly inhibits or induces CYP enzymes. In addition, the investigator should contact the Medical Monitor if questions arise regarding medications not listed above.

http://www.fda.gov/downloads/Drugs/GuidanceComplianceRegulatoryInformation/Guidances/UCM292362.pdf

http://medicine.iupui.edu/clinpharm/ddis/table.aspx

4.4.4 Prohibited Therapy

Use of the following concomitant therapies is prohibited as described below:

- Concomitant therapy intended for the treatment of cancer (including, but not limited to, chemotherapy, hormonal therapy, immunotherapy, radiotherapy, and herbal therapy), whether health authority–approved or experimental, is prohibited for various time periods prior to starting study treatment, depending on the agent (see Section 4.1.2), and during study treatment until disease recurrence is documented and the patient has discontinued study treatment, except as outlined below or as described elsewhere in the protocol.
- Investigational therapy is prohibited during the study.

"Use of this document is governed by the terms of use on the first page of this document."

- Live, attenuated vaccines (e.g., FluMist®) are prohibited within 4 weeks prior to initiation of study treatment, during study treatment, and for 5 months after the last dose of study treatment.
- Systemic immunostimulatory agents (including, but not limited to, interferons and IL-2) are prohibited within 4 weeks or 5 half-lives of the drug, whichever is longer, prior to initiation of study treatment and during study treatment because these agents could potentially increase the risk for autoimmune conditions when given in combination with atezolizumab.
- Systemic immunosuppressive medications (including, but not limited to, azathioprine, methotrexate, and thalidomide) are prohibited during study treatment because these agents could potentially alter the efficacy and safety of atezolizumab.

4.5 STUDY ASSESSMENTS

Signed informed consent will be obtained from the patient or patient's legally acceptable representative before any study-specific procedures are performed or any prohibited medications are withheld for purposes of study participation.

The schedule of activities to be performed during the study is provided in Appendix 1. All activities must be performed and documented for each patient. Patients will be closely monitored for safety and tolerability throughout the study. Patients should be assessed for toxicity prior to each dose; dosing will occur only if the clinical assessment and local laboratory test values are acceptable.

If the timing of a protocol-mandated study visit coincides with a holiday and/or weekend that would preclude the visit, the visit should be scheduled on the nearest following feasible date. Any delay visits should be reverted to the original schedule within the next few subsequent visits. For example, no study drug infusion should be skipped due to a visit delay. Assessments scheduled on the day of study drug administration should be performed prior to study drug infusion, unless otherwise noted.

Test results or examinations that are performed as standard of care prior to obtaining informed consent and appropriately within 30 days prior to randomization may be used to satisfy screening requirements rather than repeating required tests; such tests do not need to be repeated for screening.

4.5.1 Informed Consent Forms and Screening Log

Written informed consent for participation in the study must be obtained before performing any study-related procedures (including screening evaluations). Informed Consent Forms for enrolled patients and for patients who are not subsequently enrolled will be maintained at the study site. A separate optional informed consent required for collection of optional research samples (see Section 4.5.15.5).

"Use of this document is governed by the terms of use on the first page of this document."

All screening evaluations must be completed and reviewed to confirm that patients meet all eligibility criteria before enrollment. The investigator will maintain a screening log to record details of all patients screened and to confirm eligibility or record reasons for screening failure, as applicable.

Please see Appendix 1 for the schedule of screening assessments.

4.5.2 Medical History, Concomitant Medication, and Demographic Data

Medical history, including clinically significant diseases, surgeries, cancer history (including prior cancer therapies and procedures), reproductive status, smoking history, and use of alcohol and drugs of abuse, will be recorded at baseline. TNBC history will include surgery and radiation therapy.

In addition, all medications (e.g., prescription drugs, over-the-counter drugs, vaccines, herbal or homeopathic remedies, nutritional supplements) used by the patient within 30 days prior to initiation of study treatment will be recorded. At the time of each follow-up physical examination, an interval medical history should be obtained and any changes in medications and allergies should be recorded.

Demographic data will include age, sex, and self-reported race/ethnicity.

4.5.3 Physical Examinations

A complete physical examination, performed at screening, should include an evaluation of the head, eyes, ears, nose, and throat, and the cardiovascular, dermatological, musculoskeletal, respiratory, gastrointestinal, and neurological systems. Bilateral breast examination, including evaluation of locoregional lymphatics, should be conducted (see Section 4.5.5). Any abnormality identified at baseline should be recorded on the General Medical History and Baseline Conditions eCRF.

Limited, symptom-directed physical examinations should be performed at post-baseline visits and as clinically indicated. Changes from baseline abnormalities should be recorded in patient notes. New or worsened clinically significant abnormalities should be recorded as adverse events on the Adverse Event eCRF.

4.5.4 Vital Signs

Vital signs will include measurements of respiratory rate, pulse rate, systolic and diastolic blood pressure while the patient is in a seated position, and temperature.

At every clinic visit where study treatment is administered, vital signs should be measured within 60 minutes prior to the first infusion and, if clinically indicated, during or after the infusion. In addition, vital signs should be measured at other specified timepoints as outlined in the schedule of activities (see Appendix 1). Vital signs are not

"Use of this document is governed by the terms of use on the first page of this document."

required to be entered into the eCRF unless abnormal and clinically significant, in which case they are to be reported as adverse events.

4.5.5 Locoregional Tumor Status

Physical Examination

Assessment of primary tumor and regional lymph nodes must be done by physical examination during the baseline evaluation, within 3 days prior to each 4-week cycle of study treatment during the neoadjuvant phase, and within 14 days prior to surgery. The tumor site must be marked with a radiopaque marker via radiographic guidance (e.g., ultrasound) prior to initiation of neoadjuvant therapy. Clinical assessment of tumor measurement in the breast and/or lymph nodes should be conducted in a consistent manner at each evaluation. Clinical measurements of tumor in the breast should be performed, preferentially using calipers or a ruler/tape measure. The tumor should be accurately measured in at least one dimension (longest diameter to be recorded) with conventional techniques (positron emission tomography [PET] scan, CT scan, magnetic resonance imaging (MRI), ultrasound, or X-ray). If possible, these measurements should be conducted by the same assessor at baseline and throughout the neoadjuvant phase. Tumor measurements at baseline and within 14 days prior to surgery are to be recorded in the eCRF. The main purpose of performing physical examination prior to each cycle is for patient safety and to rule out progressive disease that would lead to study treatment discontinuation.

Ultrasound of Breast and Axilla

Ultrasound of breast and axilla is mandated at baseline within 28 days prior randomization and within 14 days prior to surgery. If on ultrasound examination there is evidence of suspicious axillary lymph nodes at the baseline examination, then fine-needle aspiration or core-needle biopsy is required. It is strongly recommended that the abnormal lymph node be marked by metallic indicator or other standard approach prior to neoadjuvant therapy. Sonographic tumor measurements are to be recorded in eCRF.

Additional Lymph Node Assessment

Patients with clinically negative axilla (by physical examination and radiographic imaging) may undergo an SLNB procedure prior to NAST if in keeping with local practice.

For patients who undergo SLNB, it is strongly recommended that more than one lymph node (two to three minimum) be removed. If the SLNB procedure conducted prior to NAST reveals a lymph node involved with cancer, the patient is not a candidate for the study and should not be enrolled.

Mammogram

Bilateral mammogram must be obtained at baseline. The unaffected breast should have been imaged within 60 days prior to randomization. The affected breast should be imaged within 28 days prior to randomization. Subsequent mammograms are optional

"Use of this document is governed by the terms of use on the first page of this document."

during neoadjuvant treatment and prior to surgery and should be performed per investigator's discretion. Optional procedure mammograms are not required to be entered into the eCRF. Bilateral mammogram is mandated at study completion/early termination visit and every 12 months (±4 weeks) during the follow-up period. Patients who have undergone mastectomy do not require mammograms of reconstructed breast(s).

Additional Breast Imaging

Additional breast imaging such as MRI are per investigator discretion. MRI examination is not mandated by the protocol and should be per local practice. If MRI is conducted, suggested timelines for MRI are within 28 days prior to protocol therapy, after Cycle 3, and 14 days prior to surgery.

Surgical Treatment Plan

A surgeon with experience of breast cancer surgery should evaluate patients. The proposed surgical treatment plan at baseline should be documented and reported in the eCRF. Patients should be reassessed after completion of neoadjuvant therapy/prior to surgery. The patient should be evaluated for the surgical treatment the surgeon feels is technically a candidate to undergo after completion of NAST as well as for the planned surgical treatment. These treatments should be documented and reported in the eCRF.

4.5.6 Distant Sites Tumor Assessment

Baseline distant sites tumor staging procedures should be performed in alignment with National Comprehensive Cancer Network (NCCN) or national guidelines, within 28 days prior to randomization.

As a reference, as per NCCN guidelines, staging procedures are based on clinical stage:

- For Stage II and Stage IIIA: Bone scan is to be performed in presence of bone pain and/or elevated alkaline phosphatase; abdominal/pelvic CT scan in case of elevated alkaline phosphatase, abnormal liver function tests, abdominal symptoms or abnormal physical examination; and chest CT scan.
- For Stage IIIB and Stage IIIC: Bone scan and CT scan of chest, abdomen, and pelvis should be conducted for all patients.

In addition, liver function tests, bone scans, chest X-ray/diagnostic CT scan, liver imaging, and/or other radiographic modalities may be considered when clinically indicated to exclude metastatic disease.

4.5.7 Disease Follow-Up and Confirmation of Disease Progression or Recurrence

During the neoadjuvant treatment, diagnosis of disease progression or second primary breast cancer should be supported by clinical, laboratory, radiological, and/or histological findings. Post-operatively, all patients must be followed to assess disease recurrence, second primary cancer, and survival. The designation of disease recurrence, whether

"Use of this document is governed by the terms of use on the first page of this document."

local, regional or distant, or a diagnosis of a second primary cancer can be made only when clinical, laboratory, radiological and/or histological findings support the diagnosis.

During the post-operative portion of this study, disease status should be clinically evaluated and documented every 3 months for up to 3 years and at intervals of every 6 months thereafter.

The diagnosis of a breast cancer progression, recurrence, or second primary tumor should be confirmed histologically whenever clinically possible. Given the mechanism by which immune-modulating therapies work, patients with radiographic lesions suspicious for disease recurrence are strongly recommended to undergo biopsy for histologic confirmation of cancer versus immune-mediated inflammatory process.

Some patients may experience a suspicious recurrence that leads to death relatively quickly, without the possibility of confirming relapse of disease. Efforts should be made to obtain an autopsy report in such cases.

The earliest date of diagnosis of disease progression, recurrent disease, or a diagnosis of a second primary cancer should be used and recorded. This date should be based on objective clinical, radiological, histological, or cytological evidence.

Recurrent disease includes local, regional, or distant recurrence and contralateral breast cancer. While ipsilateral or contralateral in situ disease and second primary non-breast cancers (including in situ carcinomas and non-melanoma skin cancers) will not be counted as progressive disease or recurrent disease, these events should be recorded. Patients who have a diagnosis of in situ breast disease or second (non-breast) malignancies should be maintained on a regular follow-up schedule whenever possible in order to fully capture any subsequent recurrent disease events.

The definitions of and procedures for confirming disease recurrence, death, and other noteworthy events on follow-up are provided in Table 2.

"Use of this document is governed by the terms of use on the first page of this document."

Table 2 Definitions of and Procedures for Confirming Disease Recurrence, Death, and Other Noteworthy Events on Follow-Up

**a) Local invasive recurrence**	Ipsilateral breast after previous lumpectomy	• Defined as evidence of invasive tumor (except DCIS and LCIS) in the ipsilateral breast after lumpectomy. Patients who develop clinical evidence of tumor recurrence in the remainder of the ipsilateral breast should have a biopsy of the suspicious lesion to confirm the diagnosis.<br>• Confirmed by positive histology or cytology
	Ipsilateral after previous mastectomy	• Defined as evidence of invasive tumor in any soft tissue or skin of the ipsilateral chest wall. This includes the area bounded by the midline of the sternum, extending superiorly to the clavicle and inferiorly to the costal margin. Soft tissue recurrences in this area extending into the bony chest wall or across the midline will be considered as evidence of local recurrence.<br>• Confirmed by positive histology or cytology
**b) Regional recurrence**	• Defined as the development of tumor in the ipsilateral internal mammary lymph nodes, ipsilateral axillary lymph nodes, or supraclavicular lymph nodes as well as extranodal soft tissue of the ipsilateral axilla. Regional recurrence does not include tumor in the opposite breast.<br>• Confirmed by positive histology or cytology, or radiologic evidence (especially in case of PET activity or visible internal mammary lymph nodes on CT scan or MRI if no biopsy was performed)	

"Use of this document is governed by the terms of use on the first page of this document."

Table 2 Definitions of and Procedures for Confirming Disease Recurrence, Death, and Other Noteworthy Events on Follow-Up (cont.)

**c) Distant recurrence**	• Defined as evidence of tumor in all areas, with the exception of those described in a) and b) above<br>• Confirmed by the following criteria:<br>Skin, subcutaneous tissue, and lymph nodes (other than local or regional)<br>Positive cytology, aspirate, or biopsy, OR<br>Radiological (CT scan, MRI, PET scan, or ultrasound) evidence of metastatic disease<br>Bone<br>X-ray, CT scan, or MRI evidence of lytic or blastic lesions consistent with bone metastasis, OR<br>Bone scan (requires additional radiological investigation, alone not acceptable in case of diagnostic doubt), OR<br>Biopsy proof of bone metastases or cytology<br>Bone marrow<br>Positive cytology or histology or MRI<br>Lung<br>Radiologic (CT or PET scan) evidence of multiple pulmonary nodules consistent with pulmonary metastases<br>Positive cytology or histology in case of diagnostic doubt (particularly for solitary lung lesions) if a biopsy is not performed. Serial scans should be obtained if possible to document stability or progression.<br>Proof of neoplastic pleural effusions should be established by cytology or pleural biopsy.<br>Liver<br>Radiologic evidence consistent with liver metastases, OR<br>Liver biopsy or fine-needle aspiration<br>NOTE: If radiological findings are not definitive (especially with solitary liver nodules), a liver biopsy is recommended; however, if a biopsy is not performed, serial scans should be obtained if possible to document stability or progression.<br>Central nervous system<br>Positive MRI or CT scan, usually in a patient with neurologic symptoms, OR<br>Biopsy or cytology in case of inconclusive imaging (e.g., for a diagnosis of meningeal involvement) and, depending from the general status of the patient, additional investigations (including cytology of the cerebrospinal fluid)

"Use of this document is governed by the terms of use on the first page of this document."

Table 2 Definitions of and Procedures for Confirming Disease Recurrence, Death, and Other Noteworthy Events on Follow-Up (cont.)

**d) Contralateral invasive breast cancer**	• Confirmed by positive cytology or histology
**e) Death from any cause**	• Any death occurring without prior breast cancer recurrence or second (non-breast) malignancy is considered an event for the following endpoints: EFS, DFS, and OS.

CT = computed tomography; DCIS = ductal carcinoma in situ; DFS = disease-free survival; EFS = event-free survival; iDFS = invasive disease-free survival; LCIS = lobular carcinoma in situ; MRI = magnetic resonance imaging; OS = overall survival; PET = positron emission tomography.

4.5.8 Surgical Specimen Pathology

Primary endpoint of the study (pCR) will be as identified by local pathology review. Guidelines regarding pathology specimen preparation, labeling, and review as well as calculation of the RCB index are outlined in the Pathology Manual. The Sponsor will prospectively collect Local Pathology Reports.

4.5.9 Laboratory, Biomarker, and Other Biological Samples

Samples for the following laboratory tests will be sent to the study site's local laboratory for analysis as outlined in the schedule of activities (see Appendix 1) and as clinically indicated:

- Hematology: WBC count, RBC count, hemoglobin, hematocrit, platelet count, differential count (neutrophils, eosinophils, basophils, monocytes, lymphocytes, other cells)
- Chemistry panel (serum or plasma): sodium, potassium, magnesium, chloride, bicarbonate or total CO_2, glucose, BUN or urea, creatinine, total protein, albumin, phosphorus, calcium, total bilirubin, alkaline phosphatase, ALT, AST, LDH
- Coagulation: INR, aPTT
- Thyroid function testing: thyroid-stimulating hormone, free triiodothyronine (T3) (or total T3 for sites where free T3 is not performed), free thyroxine
- HIV serology
- HBV serology: HBsAg, hepatitis B surface antibody, total HBcAb

  If a patient has a negative HBsAg test and a positive total HBcAb test at screening, an HBV DNA test must be performed to rule out active HBV infection on the basis of HBV viral load per local guidelines.
- HCV serology: HCV antibody

  If a patient has a positive HCV antibody test at screening, an HCV RNA test should be performed to rule out active HCV infection prior to initiation of study treatment.

"Use of this document is governed by the terms of use on the first page of this document."

- Pregnancy test

  All women of childbearing potential will have a serum pregnancy test at screening. Urine pregnancy tests will be performed at specified subsequent visits. If a urine pregnancy test is positive, it must be confirmed by a serum pregnancy test.

  A woman is considered to be of childbearing potential if she is postmenarcheal, has not reached a postmenopausal state (≥12 continuous months of amenorrhea with no identified cause other than menopause), and has not undergone surgical sterilization (removal of ovaries and/or uterus).

- Urinalysis (pH, specific gravity, glucose, protein, ketones, and blood); dipstick permitted

The following samples will be sent to one or several central laboratories or to the Sponsor for analysis:

- Cardiac troponin T (cTnT; for cardiac safety cohort only) (see Appendix 4)
- Serum samples for analysis of autoantibodies: anti-nuclear antibody, anti–double-stranded DNA, circulating anti-neutrophil cytoplasmic antibody, and perinuclear anti-neutrophil cytoplasmic antibody
- Serum samples for atezolizumab PK analysis through use of a validated assay
- Serum samples for assessment of ADAs to atezolizumab through use of a validated assay
- Blood and plasma samples for exploratory research on biomarkers
- Tumor tissue sample collected at baseline for determination of PD-L1 expression; for confirmation of HER2, ER, and PgR negativity; and for exploratory research on biomarkers

  A representative FFPE tumor specimen in a paraffin block (preferred) or at least 20 slides containing unstained, freshly cut, serial sections must be submitted along with an associated pathology report prior to study enrollment. After signing of the Informed Consent Form, retrieval and submission of a tumor sample can occur outside the 28-day screening period.

  Samples must contain a minimum of 50 viable tumor cells that preserve cellular context and tissue architecture regardless of needle gauge or retrieval method. Tumor tissue should be of good quality based on total and viable tumor content. Acceptable samples include those from resections, core-needle biopsies (at least three cores, embedded in a single paraffin block), or excisional, incisional, punch, or forceps biopsies. For multifocal tumors, three cores should be submitted for the main lesion, while one core is sufficient for each of the other foci. Fine-needle aspiration (defined as samples that do not preserve tissue architecture and yield cell suspension and/or smears), brushing, and cell pellets from cytology samples are not acceptable.

"Use of this document is governed by the terms of use on the first page of this document."

If tissue is unavailable and surgery has not occurred, a pretreatment tumor biopsy is required or a tumor tissue specimen should be prepared from the excised tumor. Tissue should meet the tumor tissue requirements described in the eligibility criteria. Remaining tumor tissue for enrolled patients will be returned to the site upon request or within 18 months after final closure of the study database, whichever occurs first. Remaining tumor tissue for patients who are not enrolled in the study will be returned to the site no later than 6 weeks after eligibility determination.

- Tumor samples for biomarker evaluation will be collected to promote, facilitate, and improve the treatment and mode of action of atezolizumab/abraxane and atezolizumab/doxorubicin/cyclophosphamide. An optional core-needle biopsy will be requested predose at Week 3 (±3 days). Tissue will also be collected at eventual surgical resection.
- Tumor tissue sample collected at the time of recurrence, if deemed clinically feasible by the investigator, for exploratory research on biomarkers

  Biopsies should be performed within 40 days after recurrence or prior to the next anti-cancer therapy, whichever is sooner. Acceptable samples include those from resections, core-needle biopsies (at least two cores preferred), or excisional, incisional, punch, or forceps biopsies.

Exploratory biomarker research may include, but will not be limited to, analysis of genes or gene signatures associated with tumor immunobiology, PD-L1, lymphocytes, T-cell receptor repertoire, or cytokines associated with T-cell activation and may involve DNA or RNA extraction, analysis of somatic mutations, and use of WGS or NGS.

NGS may be performed by Foundation Medicine on the samples obtained at disease relapse after surgical resection. If performed by Foundation Medicine, the investigator can obtain results from these analyses in the form of an NGS report, which is available upon request directly from Foundation Medicine. The investigator may share and discuss the results with the patient, unless the patient chooses otherwise.
The Foundation Medicine NGS assay has not been cleared or approved by health authorities. The NGS report is generated for research purposes and is not provided for the purpose of guiding future treatment decisions.

For sampling procedures, storage conditions, and shipment instructions, see the laboratory manual.

Unless the patient gives specific consent for his or her leftover samples to be stored for optional exploratory research (see Section 4.5.14), biological samples will be destroyed when the final Clinical Study Report has been completed, with the following exceptions:

- Serum samples collected for PK analysis and/or immunogenicity analysis may be needed for additional immunogenicity characterization and PK and immunogenicity assay development and validation; therefore, these samples will be destroyed no later than 5 years after the final Clinical Study Report has been completed.

"Use of this document is governed by the terms of use on the first page of this document."

- Blood samples collected for WGS will be stored until they are no longer needed or until they are exhausted.
- Leftover plasma, blood, and tumor samples collected during the study will be destroyed no later than 15 years after the final Clinical Study Report has been completed or in accordance to local regulations.

When a patient withdraws from the study, samples collected prior to the date of withdrawal may still be analyzed, unless the patient specifically requests that the samples be destroyed or local laws require destruction of the samples. However, if samples have been tested prior to withdrawal, results from those tests will remain as part of the overall research data.

Data arising from sample analysis will be subject to the confidentiality standards described in Section 8.4.

Given the complexity and exploratory nature of exploratory biomarker analyses, data derived from these analyses will generally not be provided to study investigators or patients unless required by law, with the exception of the report from Foundation Medicine for the samples obtained at disease relapse. The aggregate results of any conducted research will be available in accordance with the effective Sponsor policy on study data publication.

4.5.10 Electrocardiograms

A 12-lead ECG is required at screening and when clinically indicated (see Appendix 1). ECGs for each patient should be obtained from the same machine wherever possible. Lead placement should be as consistent as possible. ECG recordings must be performed after the patient has been resting in a supine position for at least 10 minutes.

For safety monitoring purposes, the investigator must review, sign, and date all ECG tracings. Paper copies of ECG tracings will be kept as part of the patient's permanent study file at the site. Any morphologic waveform changes or other ECG abnormalities must be documented on the eCRF.

4.5.11 Echocardiograms or Multiple-Gated Acquisition Scans

LVEF will be assessed by echocardiography (preferably) or MUGA scan at specified timepoints, as outlined in the schedule of activities (see Appendix 1 and Appendix 4) and as clinically indicated. Patients should be reassessed with the same technique used for baseline cardiac evaluation throughout the study.

"Use of this document is governed by the terms of use on the first page of this document."

4.5.12 Pulmonary Function Tests (Spirometry)

Pulmonary function evaluations will be conducted via spirometry at baseline, and if clinically indicated, repeat tests may be conducted by the investigator as part of a clinical workup to evaluate pulmonary symptoms. Baseline pulmonary function evaluations will include assessments of forced vital capacity (FVC), forced expiratory volume 1 (FEV1), and forced expiratory flow 25%–75% (FEF25-75).

4.5.13 Patient-Reported Outcomes

To more fully characterize the clinical profile of atezolizumab, PRO data will be obtained through use of the following instruments: EORTC QLQ-C30; item GP5 of the FACT-G, Quality of Life instrument; and the EuroQoL 5-Dimension, 5-Level (EQ-5D-5L).

The PRO instruments, translated as required in the local language, will be distributed by the investigator staff and completed on paper in their entirety by the patient at the investigational site. To ensure instrument validity and that data standards meet health authority requirements, questionnaires must be completed by the patient at the start of the clinic visit before discussion of the patient's health state, laboratory results, or health record; before administration of study treatment; and/or prior to the performance of any other study assessments that could bias the patient's responses. If the patient is unable to complete the measure on her or his own, interviewer assessment is allowed but may only be conducted by a member of the clinic staff who reads the questionnaire items to the patient verbatim; no interpretation, rephrasing, or rewording of the questions is allowed during interview-assisted completion.

Study personnel should review all questionnaires for completeness before the patient leaves the investigational site, and the hard copy originals of the questionnaires must be maintained as part of the patient's medical record at the site for source data verification. These originals should have the respondent's initials, study patient number and date and time of completion recorded in compliance with good clinical practice. Sites will enter patient responses to the PRO questionnaires into the electronic data capture (EDC) system.

All patients will begin completion of the questionnaires with the EORTC QLQ-C30, followed by the FACT-G single item GP5, and then the EQ-5D-5L at timepoints corresponding with in-clinic visits; both while receiving study treatment and after treatment discontinuation. Refer to Appendix 1 for the frequency and timing of PRO assessments.

4.5.13.1 EORTC QLQ-C30

The EORTC QLQ-C30 is a validated, reliable self-report measure (Aaronson et al. 1993; see Appendix 5). It consists of 30 questions that assess 5 aspects of patient functioning (physical, emotional, role, cognitive, and social), 3 symptom scales (fatigue, nausea and vomiting, pain), global health status (GHS), HRQoL, and 6 single items (dyspnea, insomnia, appetite loss, constipation, diarrhea, and financial difficulties) with a recall

"Use of this document is governed by the terms of use on the first page of this document."

period of the previous week. Scale scores can be obtained for the multi-item scales. The EORTC QLQ-C30 module takes approximately 10 minutes to complete.

4.5.13.2 FACT-G Single Item GP5

The FACT-G instrument Version 4 (see Appendix 6) is a validated and reliable 27-item questionnaire comprised of 4 subscales that measure physical (7 items), social/family (7 items), emotional (6 items) and functional well-being (7 items), and is considered appropriate for use with patients with any form of cancer (Cella et al. 1993; Webster et al. 1999). In this study, the single item GP5 ("I am bothered by side effects of treatment") from the physical well-being subscale of the FACT-G has been selected for individual item analysis to document the level of bother of symptoms on patient's lives. Patients will assess how true the statement "I am bothered by side effects of treatment" has been for them in the previous 7 days on a 5-point scale (0, not at all; 1, a little bit; 2, somewhat; 3, quite a bit; 4, very much). The single item GP5 from the FACT-G takes less than a minute to complete.

4.5.13.3 EQ-5D-5L

The EQ-5D-5L, is a validated self-report health status questionnaire that is used to calculate a health status utility score for use in health economic analyses (EuroQol Group 1990; Brooks 1996; Herdman et al. 2011; Janssen et al. 2013; see Appendix 7). There are two components to the EQ-5D-5L: a five-item health state profile that assesses mobility, self-care, usual activities, pain/discomfort, and anxiety/depression, as well as a visual analogue scale that measures health state. Published weighting systems allow for creation of a single composite score of the patient's health utility. Utility scores will be used in this study for informing pharmacoeconomic evaluations. As such, the utility results will not be included in the CSR. The EQ-5D-5L takes approximately 3 minutes to complete.

4.5.14 Mandatory Samples for Whole Genome Sequencing

At participating sites, blood samples will be collected for DNA extraction to enable WGS to identify germline mutations that are predictive of response to study drug, are associated with progression to a more severe disease state, are associated with susceptibility to developing adverse events, or can increase the knowledge and understanding of disease biology. The blood samples may be sent to one or more laboratories for analysis.

Collection and submission of WGS samples is contingent upon the review and approval of the exploratory research by each site's IRB/EC and, if applicable, an appropriate regulatory body. If a site has not been granted approval for WGS sampling, this section of the protocol (Section 4.5.14) will not be applicable at that site.

Genomics is increasingly informing researcher's understanding of disease pathobiology. WGS provides a comprehensive characterization of the genome and, along with clinical data collected in this study, may increase the opportunity for

"Use of this document is governed by the terms of use on the first page of this document."

developing new therapeutic approaches. Data will be analyzed in the context of this study but will also be explored in aggregate with data from other studies. The availability of a larger dataset will assist in identification of important pathways, guiding the development of new targeted agents.

For sampling procedures, storage conditions, and shipment instructions, see the laboratory manual.

Blood samples collected for WGS are to be stored until they are no longer needed or until they are exhausted. However, the storage period will be in accordance with the IRB/EC-approved Informed Consent Form and applicable laws (e.g., health authority requirements).

Patient medical information associated with WGS specimens is confidential and may be disclosed to third parties only as permitted by the Informed Consent Form (or separate authorization for use and disclosure of personal health information) signed by the patient, unless permitted or required by law.

Given the complexity and exploratory nature of the analyses, data derived from WGS specimens will generally not be provided to study investigators or patients unless required by law. The aggregate results of any conducted research will be available in accordance with the effective Sponsor policy on study data publication.

4.5.15 Optional Samples for Research Biosample Repository

4.5.15.1 Overview of the Research Biosample Repository

The Research Biosample Repository (RBR) is a centrally administered group of facilities used for the long-term storage of human biologic specimens, including body fluids, solid tissues, and derivatives thereof (e.g., DNA, RNA, proteins, peptides). The collection, storage, and analysis of RBR specimens will facilitate the rational design of new pharmaceutical agents and the development of diagnostic tests, which may allow for individualized drug therapy for patients in the future.

Specimens for the RBR will be collected from patients who give specific consent to participate in this optional research. RBR specimens will be used to achieve the following objectives:

- To study the association of biomarkers with efficacy, adverse events, or disease recurrence
- To increase knowledge and understanding of disease biology
- To study drug response, including drug effects and the processes of drug absorption and disposition
- To develop biomarker or diagnostic assays and establish the performance characteristics of these assays

"Use of this document is governed by the terms of use on the first page of this document."

4.5.15.2 Approval by the Institutional Review Board or Ethics Committee

Collection and submission of biological samples to the RBR is contingent upon the review and approval of the exploratory research and the RBR portion of the Informed Consent Form by each site's IRB/EC and, if applicable, an appropriate regulatory body. If a site has not been granted approval for RBR sampling, this section of the protocol (Section 4.5.15) will not be applicable at that site.

4.5.15.3 Sample Collection

The following samples will be stored in the RBR and used for research purposes, including, but not limited to, research on biomarkers related to atezolizumab or diseases:

- Whole blood sample for DNA isolation collected from patients who have consented to optional RBR sampling at Cycle 1, Day 1 (see Appendix 1)

  If, however, the RBR genetic blood sample is not collected during the scheduled visit, it may be collected as soon as possible (after randomization) during the conduct of the clinical study.

  Collection of whole blood will enable the evaluation of single nucleotide polymorphisms in genes associated with immune biology including but not restricted to the target and pathway associated genes such as PD-L1, PD-1, and B7.1 as well as IL-8, IL-6, and related cytokines. The sample may be processed using techniques such as kinetic PCR and DNA sequencing.

- Tumor tissue samples from biopsies performed at the investigator's discretion during the study
- Leftover blood, plasma, and tumor tissue samples (with the exception of leftover tissue from FFPE blocks, which will be returned to sites) and any derivatives thereof (e.g., DNA, RNA, proteins, peptides), including leftover tissue samples from additional tumor biopsies or medically indicated procedures (e.g., bronchoscopy, esophagogastroduodenoscopy, colonoscopy) performed at the investigator's discretion during the course of the study

The above samples may be sent to one or more laboratories for analysis of germline mutations or somatic mutations via WGS, NGS, or other genomic analysis methods.

Genomics is increasingly informing researchers' understanding of disease pathobiology. WGS provides a comprehensive characterization of the genome and, along with clinical data collected in this study, may increase the opportunity for developing new therapeutic approaches. Data will be analyzed in the context of this study but will also be explored in aggregate with data from other studies. The availability of a larger dataset will assist in identification of important pathways, guiding the development of new targeted agents.

For sampling procedures, storage conditions, and shipment instructions, see the laboratory manual.

"Use of this document is governed by the terms of use on the first page of this document."

RBR specimens are to be stored until they are no longer needed or until they are exhausted. However, the RBR storage period will be in accordance with the IRB/EC-approved Informed Consent Form and applicable laws (e.g., health authority requirements).

4.5.15.4 Confidentiality

Specimens and associated data will be labeled with a unique patient identification number.

Patient medical information associated with RBR specimens is confidential and may be disclosed to third parties only as permitted by the Informed Consent Form (or separate authorization for use and disclosure of personal health information) signed by the patient, unless permitted or required by law.

Given the complexity and exploratory nature of the analyses of RBR specimens, data derived from these analyses will generally not be provided to study investigators or patients unless required by law. The aggregate results of any conducted research will be available in accordance with the effective Sponsor policy on study data publication.

Data generated from RBR specimens must be available for inspection upon request by representatives of national and local health authorities, and Sponsor monitors, representatives, and collaborators, as appropriate.

Any inventions and resulting patents, improvements, and/or know-how originating from the use of the RBR data will become and remain the exclusive and unburdened property of the Sponsor, except where agreed otherwise.

4.5.15.5 Consent to Participate in the Research Biosample Repository

The Informed Consent Form will contain a separate section that addresses participation in the RBR. The investigator or authorized designee will explain to each patient the objectives, methods, and potential hazards of participation in the RBR. Patients will be told that they are free to refuse to participate and may withdraw their specimens at any time and for any reason during the storage period. A separate, specific signature will be required to document a patient's agreement to provide optional RBR specimens. Patients who decline to participate will not provide a separate signature.

The investigator should document whether or not the patient has given consent to participate and (if applicable) the date(s) of consent, by completing the RBR Research Sample Informed Consent eCRF.

In the event of an RBR participant's death or loss of competence, the participant's specimens and data will continue to be used as part of the RBR research.

"Use of this document is governed by the terms of use on the first page of this document."

4.5.15.6 Withdrawal from the Research Biosample Repository

Patients who give consent to provide RBR specimens have the right to withdraw their specimens from the RBR at any time for any reason. After withdrawal of consent, any remaining samples will be destroyed or will no longer be linked to the patient. However, if RBR samples have been tested prior to withdrawal of consent, results from those tests will remain as part of the overall research data. If a patient wishes to withdraw consent to the testing of his or her samples during the study, the investigator must inform the Medical Monitor in writing of the patient's wishes through use of the appropriate RBR Subject Withdrawal Form and must enter the date of withdrawal on the RBR Research Sample Withdrawal of Informed Consent eCRF. If a patient wishes to withdraw consent to the testing of his or her RBR samples after closure of the site, the investigator must inform the Sponsor by emailing the study number and patient number to the following email address:

global_rcr-withdrawal@roche.com

A patient's withdrawal from Study WO39392 does not, by itself, constitute withdrawal of specimens from the RBR. Likewise, a patient's withdrawal from the RBR does not constitute withdrawal from Study WO39392.

4.5.15.7 Monitoring and Oversight

RBR specimens will be tracked in a manner consistent with Good Clinical Practice by a quality-controlled, auditable, and appropriately validated laboratory information management system to ensure compliance with data confidentiality as well as adherence to authorized use of specimens as specified in this protocol and in the Informed Consent Form. Sponsor monitors and auditors will have direct access to appropriate parts of records relating to patient participation in the RBR for the purposes of verifying the data provided to the Sponsor. The site will permit monitoring, audits, IRB/EC review, and health authority inspections by providing direct access to source data and documents related to the RBR samples.

4.6 TREATMENT, PATIENT, STUDY, AND SITE DISCONTINUATION

4.6.1 Study Treatment Discontinuation

Patients must permanently discontinue study treatment (atezolizumab) if they experience any of the following:

- Intolerable toxicity related to study treatment, including development of an immune-mediated adverse event determined by the investigator to be unacceptable given the individual patient’s potential response to therapy and severity of the event
- Any adverse event that requires study treatment discontinuation per the guidelines in Section 5.1 and the Atezolizumab Investigator's Brochure
- Any medical condition that may jeopardize the patient’s safety if he or she continues study treatment
- Investigator or Sponsor determines it is in the best interest of the patient

"Use of this document is governed by the terms of use on the first page of this document."

- Use of another non-protocol anti-cancer therapy
- Pregnancy
- Confirmation of disease progression or disease recurrence
- Withdrawal by patient

The primary reason for study treatment discontinuation should be documented on the appropriate eCRF. Patients who discontinue study treatment prematurely will not be replaced.

Patients who discontinue study treatment early because of an unacceptable toxicity should continue to be followed for both resolution of toxicity and disease recurrence as specified in Appendix 1. Patients must discontinue study treatment if they experience disease progression or recurrence. After disease progression or recurrence, patients will be followed until the next treatment and then for survival until study completion. Patients who are discontinued because of investigator decision will be followed for disease recurrence, new anti-cancer therapy, and survival (see Section 4.6.2 for further details).

4.6.2 Patient Discontinuation from Study

Patients have the right to voluntarily withdraw from the study at any time for any reason. In addition, the investigator has the right to withdraw a patient from the study at any time. Reasons for withdrawal from the study may include, but are not limited to, the following:

- Patient withdrawal of consent

  From study treatment and/or procedures only (i.e., survival follow-up permitted), and or

  From survival follow-up (patient does not want to be contacted)

- Study termination (see Section 4.6.3) or site closure (see Section 4.6.4)
- Investigator or Sponsor determines it is in the best interest of the patient
- Patient non-compliance, defined as failure to comply with protocol requirements as determined by the investigator or Sponsor

Every effort should be made to obtain information on patients who withdraw from the study. The primary reason for withdrawal from the study should be documented on the appropriate eCRF. Patients who withdraw from the study will not be replaced.

4.6.3 Study Discontinuation

The Sponsor has the right to terminate this study at any time. Reasons for terminating the study may include, but are not limited to, the following:

- The incidence or severity of adverse events in this or other studies indicates a potential health hazard to patients.
- Patient enrollment is unsatisfactory.

"Use of this document is governed by the terms of use on the first page of this document."

The Sponsor will notify the investigator if the Sponsor decides to discontinue the study.

Patients who are withdrawn or withdraw from study participation (and not just study treatment) will not be followed up for any reason after consent has been withdrawn.

4.6.4 Site Discontinuation

The Sponsor has the right to close a site at any time. Reasons for closing a site may include, but are not limited to, the following:

- Excessively slow recruitment
- Poor protocol adherence
- Inaccurate or incomplete data recording
- Non-compliance with the International Council for Harmonisation (ICH) guideline for Good Clinical Practice
- No study activity (i.e., all patients have completed the study and all obligations have been fulfilled)

5. ASSESSMENT OF SAFETY

5.1 SAFETY PLAN

The safety plan for patients in this study is based on clinical experience with atezolizumab and nab-paclitaxel, doxorubicin, and cyclophosphamide in completed and ongoing studies. The anticipated important safety risks are outlined below (see Sections 5.1.1–5.1.4). Guidelines for management of patients who experience specific adverse events are provided in Table 4 (see Section 5.1.5).

Measures will be taken to ensure the safety of patients participating in this study, including the use of stringent inclusion and exclusion criteria (see Section 4.1) and close monitoring of patients during the study as indicated below. Administration of atezolizumab will be performed in a monitored setting in which there is immediate access to trained personnel and adequate equipment and medicine to manage potentially serious reactions. Adverse events will be reported as described in Sections 5.2–5.6.

An iDMC (see Section 3.1.2) has also been incorporated into the trial design to periodically review aggregate safety data.

The potential safety issues anticipated in this trial, as well as measures intended to avoid or minimize such toxicities, are outlined in the following sections.

5.1.1 Risks Associated with Atezolizumab

Atezolizumab has been associated with risks such as the following: IRRs and *immune-mediated* hepatitis, pneumonitis, colitis, pancreatitis, diabetes mellitus, hypothyroidism, hyperthyroidism, adrenal insufficiency, hypophysitis, Guillain-Barré

"Use of this document is governed by the terms of use on the first page of this document."

syndrome, myasthenic syndrome or myasthenia gravis, meningoencephalitis, myocarditis, nephritis, and myositis. *Immune-mediated reactions may involve any organ system and may lead to hemophagocytic lymphohistiocytosis and macrophage activation syndrome (considered to be potential risks for atezolizumab).* Adverse events observed with atezolizumab in combination with chemotherapy and/or targeted therapies are consistent with the known risks of each component of the combination treatment. Refer to Appendix 13 of the protocol and Section 6 of the Atezolizumab Investigator's Brochure for a detailed description of anticipated safety risks for atezolizumab.

5.1.2 Risks Associated with Nab-Paclitaxel

In clinical studies and post-marketing experience, nab-paclitaxel has been associated with alopecia, myelosuppression (primarily neutropenia [including febrile neutropenia], anemia, thrombocytopenia), neuropathy, cranial nerve palsies, hypersensitivity reactions, pneumonitis, gastrointestinal events (i.e., nausea, vomiting, diarrhea), myalgia, arthralgia, cardiotoxicity (myocardial disorders, cardiac failure, angina, tachycardia, ventricular arrhythmia), cystoid macular edema, Stevens-Johnson syndrome/toxic epidermal necrolysis, infections including sepsis, depression, infusion site reactions/extravasation, hepatic toxicity (drug-induced liver injury), acute renal failure, hemolytic-uremic syndrome, drug-induced lupus erythematous *and tumor lysis syndrome.*

Patients will be monitored for nab-paclitaxel–related adverse events, including hematologic, gastrointestinal, hepatic toxicities, and peripheral neuropathy.

For more details regarding the safety profile of nab-paclitaxel, refer to the local prescribing information. Other specific instructions can be found in Sections 4.4.3 and 5.1.5 for nab-paclitaxel.

5.1.3 Risks Associated with Doxorubicin

Doxorubicin is known to cause serious cardiomyopathy; arrhythmias, including life-threatening arrhythmias; congestive cardiac failure; increased incidence of secondary acute myelogenous leukemia and myelodysplastic syndrome; extravasation and tissue necrosis; severe myelosuppression resulting in serious infection, septic shock, need for transfusions, hospitalization, and death; tumor lysis syndrome; radiation sensitization and radiation recall; embryofetal toxicity; alopecia; and vomiting, nausea, and other gastrointestinal effects.

Refer to the doxorubicin Summary of Product Characteristics (package insert) for more details regarding the full safety profile of doxorubicin, including boxed warnings and contraindications.

5.1.3.1 Cardiomyopathy

Patients treated with doxorubicin are at risk of developing cumulative dose-related myocardial damage. Significant cardiac events, including acute heart failure and LVEF

"Use of this document is governed by the terms of use on the first page of this document."

of <40%, have been observed in clinical trials of doxorubicin. Cardiomyopathy may develop during treatment or up to several years after completion of treatment. There is an additive or potentially synergistic increase in the risk of cardiomyopathy in patients who have received radiotherapy to the mediastinum or concomitant therapy with other known cardiotoxic agents such as cyclophosphamide.

Patients must meet specified LVEF requirements to be included in this study (see Section 4.1.1).

Left ventricular function will be monitored by measurement of ejection fraction using ECHO or MUGA scans as described in Section 4.5.10 and the schedule of activities (see Appendix 1).

5.1.4 Risks Associated with Cyclophosphamide

Cyclophosphamide has been associated with myelosuppression sometimes leading to severe immunosuppression and infections that can be serious and sometimes fatal; hemorrhagic cystitis, pyelitis, ureteritis, and hematuria; myocarditis, myopericarditis, pericardial effusion, arrhythmias, and congestive heart failure; pulmonary toxicity including pneumonitis, pulmonary fibrosis, and pulmonary veno-occlusive disease leading to respiratory failure; impairment of spermatogenesis; secondary malignancies; veno-occlusive liver disease; embryo-fetal toxicity; alopecia; and nausea, vomiting, and diarrhea.

Refer to the cyclophosphamide Summary of Product Characteristics (package insert) for more details regarding the full safety profile of cyclophosphamide, including boxed warnings and contraindications.

5.1.5 Management of Patients Who Experience Specific Adverse Events

There will be no dose modifications for atezolizumab or placebo in this study.

For guidelines regarding the management of atezolizumab-specific adverse events, please refer to Appendix 13 of the protocol and Section 6 of the Atezolizumab Investigator's Brochure.

Study treatment may be temporarily suspended in patients experiencing toxicity considered to be related to study treatment. If atezolizumab/placebo is withheld for > 12 weeks after the event onset, the patient will be discontinued from atezolizumab/placebo. If the investigator believes the patient is likely to derive clinical benefit and the Medical Monitor is in agreement, atezolizumab/placebo treatment can be resumed after being withheld for >12 weeks. If a patient needs to be tapered off corticosteroids that were used to treat adverse events, the study treatment may be withheld for >12 weeks. The investigator and the Medical Monitor will determine the acceptable length of treatment interruption.

"Use of this document is governed by the terms of use on the first page of this document."

Dose interruptions for reasons other than toxicity (e.g., surgical procedures) may be allowed with Medical Monitor approval. The investigator and the Medical Monitor will determine the acceptable length of treatment interruption.

For guidelines regarding the management (i.e., dose modification and treatment interruption rules) of doxorubicin- or cyclophosphamide-associated toxicities, please refer to the respective local prescribing information for each agent.

For nab-paclitaxel–specific management guidelines, please see below. For further guidance, please refer to local prescribing information for nab-paclitaxel.

Table 3 Dose Levels for Nab-Paclitaxel (Weekly Dose)

	Dose Level 0 (Starting Dose)	Dose Level −1	Dose Level −2
Nab-paclitaxel weekly dose	125 mg/m^2	100 mg/m^2	Discontinue nab-paclitaxel

Table 4 Guidelines for Management of Patients Who Experience Nab-Paclitaxel–Specific Adverse Events

Event	Action to Be Taken
**Hepatotoxicity: Bilirubin or AST or ALT Increased (Grades 2–4)**	
	• Hold nab-paclitaxel until bilirubin returns to baseline grade and AST and ALP have returned to Grade ≤ 1 (check weekly).
Grade 2	• Decrease nab-paclitaxel one dose level.
Grade 3	• Decrease nab-paclitaxel one dose level.
Grade 4	• Discontinue nab-paclitaxel.
**Neurologic Disorders**	
Neuropathy, Grade 2	• Withhold nab-paclitaxel if persistent > 7 days or caused the next cycle to be delayed.<br>• Resume nab-paclitaxel if event resolves to Grade ≤ 1 and decrease by 1 dose level.<br>• If persistent after 3 weeks of withholding nab-paclitaxel, discontinue nab-paclitaxel.
Neuropathy, Grade 3 or 4	• If first episode, withhold nab-paclitaxel. If persistent for > 7 days or caused the next cycle to be delayed, resume nab-paclitaxel if event resolves to Grade ≤ 1 on Day 1 of the next cycle, and decrease by 1 dose level.<br>• If second episode, discontinue nab-paclitaxel permanently.

"Use of this document is governed by the terms of use on the first page of this document."

Table 4 Guidelines for Management of Patients Who Experience Nab-Paclitaxel–Specific Adverse Events (cont.)

Event	Action to Be Taken
**Hematologic Toxicity**	
Neutrophils count decreased	
Grade 2	• Maintain nab-paclitaxel dose and add G–CSF.
Grade 3 or 4	• Hold nab-paclitaxel, give G–CSF, and observe recovery period:<br>– If recovery to ANC ≥ 1500 cells/mm^3 occurs within 1 week, resume nab-paclitaxel at the same dose and continue G–CSF as required.<br>– If recovery to ANC ≥ 1500 cells/mm^3 takes more than 1 week, decrease nab-paclitaxel 1 dose level and continue G-CSF as required.
Platelet count decreased	
Grade 2 or 3	• If resolves prior to next treatment cycle, maintain nab-paclitaxel dose.<br>• If requires a delay in the administration of next treatment cycle:<br>– Hold nab-paclitaxel until platelets ≥ 75,000 cells/mm3.<br>– If recovery takes 1 week, maintain nab-paclitaxel dose.<br>– If recovery takes 2–3 weeks, decrease 1 dose level.
Grade 4	• Hold nab-paclitaxel until platelets ≥ 75,000 cells/mm^3.<br>• Decrease nab-paclitaxel one dose level.

G-CSF = granulocyte colony–stimulating factor.

5.2 SAFETY PARAMETERS AND DEFINITIONS

Safety assessments will consist of monitoring and recording protocol-defined adverse events (AEs), including but not restricted to serious adverse events (SAEs) and adverse events of special interest, performing protocol-specified safety laboratory assessments, measuring protocol-specified vital signs, and conducting other protocol-specified tests that are deemed critical to the safety evaluation of the study.

Certain types of events require immediate reporting to the Sponsor, as outlined in Section 5.4.

"Use of this document is governed by the terms of use on the first page of this document."

5.2.1 Adverse Events

According to the ICH guideline for Good Clinical Practice, an adverse event is any untoward medical occurrence in a clinical investigation subject administered a pharmaceutical product, regardless of causal attribution. An adverse event can therefore be any of the following:

- Any unfavorable and unintended sign (including an abnormal laboratory finding), symptom, or disease temporally associated with the use of a medicinal product, whether or not considered related to the medicinal product
- Any new disease or exacerbation of an existing disease (a worsening in the character, frequency, or severity of a known condition), except as described in Section 5.3.5.9
- Recurrence of an intermittent medical condition (e.g., headache) not present at baseline
- Any deterioration in a laboratory value or other clinical test (e.g., ECG, X-ray) that is associated with symptoms or leads to a change in study treatment or concomitant treatment or discontinuation from study treatment
- Adverse events that are related to a protocol-mandated intervention, including those that occur prior to assignment of study treatment (e.g., screening invasive procedures such as biopsies)

5.2.2 Serious Adverse Events (Immediately Reportable to the Sponsor)

A serious adverse event is any adverse event that meets any of the following criteria:

- Is fatal (i.e., the adverse event actually causes or leads to death)
- Is life threatening (i.e., the adverse event, in the view of the investigator, places the patient at immediate risk of death)

  This does not include any adverse event that had it occurred in a more severe form or was allowed to continue might have caused death.
- Requires or prolongs inpatient hospitalization (see Section 5.3.5.11)
- Results in persistent or significant disability/incapacity (i.e., the adverse event results in substantial disruption of the patient’s ability to conduct normal life functions)
- Is a congenital anomaly/birth defect in a neonate/infant born to a mother exposed to study treatment
- Is a significant medical event in the investigator's judgment (e.g., may jeopardize the patient or may require medical/surgical intervention to prevent one of the outcomes listed above)

"Use of this document is governed by the terms of use on the first page of this document."

The terms "severe" and "serious" are not synonymous. Severity refers to the intensity of an adverse event (e.g., rated as mild, moderate, or severe, or according to NCI CTCAE; see Section 5.3.3); the event itself may be of relatively minor medical significance (such as severe headache without any further findings).

Severity and seriousness need to be independently assessed for each adverse event recorded on the eCRF.

Serious adverse events are required to be reported by the investigator to the Sponsor immediately (i.e., no more than 24 hours after learning of the event; see Section 5.4.2 for reporting instructions).

5.2.3 Adverse Events of Special Interest (Immediately Reportable to the Sponsor)

Adverse events of special interest are required to be reported by the investigator to the Sponsor immediately (i.e., no more than 24 hours after learning of the event; see Section 5.4.2 for reporting instructions). Adverse events of special interest for this study include the following:

- Cases of potential drug-induced liver injury that include an elevated ALT or AST in combination with either an elevated bilirubin or clinical jaundice, as defined by Hy's Law (see Section 5.3.5.7) and based on the following observations:

  Treatment-emergent ALT or AST $> 3 \times$ ULN (or baseline value if baseline value was above the ULN) in combination with total bilirubin $> 2 \times$ ULN (of which $\geq 35\%$ is direct bilirubin)

  Treatment-emergent ALT or AST $> 3 \times$ ULN (or baseline value if baseline value was above the ULN) in combination with clinical jaundice

- Suspected transmission of an infectious agent by the study treatment, as defined below:

  Any organism, virus, or infectious particle (e.g., prion protein–transmitting transmissible spongiform encephalopathy), pathogenic or non-pathogenic, is considered an infectious agent. A transmission of an infectious agent may be suspected from clinical symptoms or laboratory findings that indicate an infection in a patient exposed to a medicinal product. This term applies only when a contamination of study treatment is suspected.

- Pneumonitis
- Colitis
- Endocrinopathies: diabetes mellitus, pancreatitis, adrenal insufficiency, hyperthyroidism, and hypophysitis
- Hepatitis, including AST or ALT $> 10 \times$ ULN
- Systemic lupus erythematosus

"Use of this document is governed by the terms of use on the first page of this document."

- Neurological disorders: Guillain-Barré syndrome, myasthenic syndrome or myasthenia gravis, and meningoencephalitis
- Events suggestive of hypersensitivity, infusion-related reactions (IRRs), cytokine release syndrome, influenza-like illness, *and* systemic inflammatory response syndrome
- Nephritis
- Ocular toxicities (e.g., uveitis, retinitis)
- Myositis
- Myopathies, including rhabdomyolysis
- Grade ≥2 cardiac disorders (e.g., atrial fibrillation, myocarditis, pericarditis)
- Vasculitis

5.2.4 Selected Adverse Events

Additional data will be collected for the selected adverse events in the following sections.

5.2.4.1 Cardiac, General

Symptomatic left ventricular systolic dysfunction should be reported as a serious adverse event. If the diagnosis is heart failure, it should be reported as such and not in terms of the individual signs and symptoms thereof.

Heart failure should be graded according to the NCI CTCAE v4.0 for "heart failure" (Grade 2, 3, 4, or 5) and in addition according to the NYHA classification.

Heart failure occurring during the study and post-study (see Section 5.6) must be reported, irrespective of causal relationship, and followed until one of the following occurs: resolution or improvement to baseline status, no further improvement can be expected, or death.

5.2.4.2 Asymptomatic Left Ventricular Systolic Dysfunction

Asymptomatic declines in LVEF should generally not be reported as adverse events because LVEF data are collected separately in the eCRF. Exceptions to this rule are as follows:

- An asymptomatic decline in LVEF ≥10 percentage points from baseline to an LVEF <50% must be reported as an adverse event with the term "asymptomatic ejection fraction decreased" as per NCI CTCAE v4.0.
- An asymptomatic decline in LVEF requiring treatment delay or leading to discontinuation of atezolizumab, nab-paclitaxel, doxorubicin, or cyclophosphamide must also be reported.

"Use of this document is governed by the terms of use on the first page of this document."

5.3 METHODS AND TIMING FOR CAPTURING AND ASSESSING SAFETY PARAMETERS

The investigator is responsible for ensuring that all adverse events (see Section 5.2.1 for definition) are recorded on the Adverse Event eCRF and reported to the Sponsor in accordance with instructions provided in this section and in Sections 5.5–5.6.

For each adverse event recorded on the Adverse Event eCRF, the investigator will make an assessment of seriousness (see Section 5.2.2 for seriousness criteria), severity (see Section 5.3.3), and causality (see Section 5.3.4).

5.3.1 Adverse Event Reporting Period

Investigators will seek information on adverse events at each patient contact. All adverse events, whether reported by the patient or noted by study personnel, will be recorded in the patient's medical record and on the Adverse Event eCRF.

After informed consent has been obtained **but prior to initiation of study treatment**, only serious adverse events caused by a protocol-mandated intervention (e.g., invasive procedures such as biopsies, discontinuation of medications) should be reported (see Section 5.4.2 for instructions for reporting serious adverse events).

After initiation of study treatment, all adverse events will be reported until 30 days after the last dose of study treatment or until initiation of new anti-cancer therapy, whichever occurs first, and serious adverse events and adverse events of special interest will continue to be reported until 90 days after the last dose of study treatment or until initiation of new anti-cancer therapy, whichever occurs first.

Study drug–related SAEs will be recorded indefinitely (even if the study has been closed). Instructions for reporting adverse events that occur after the adverse event reporting period are provided in Section 5.6.

5.3.2 Eliciting Adverse Event Information

A consistent methodology of non-directive questioning should be adopted for eliciting adverse event information at all patient evaluation timepoints. Examples of non-directive questions include the following:

"How have you felt since your last clinic visit?"

"Have you had any new or changed health problems since you were last here?"

5.3.3 Assessment of Severity of Adverse Events

The adverse event severity grading scale for the NCI CTCAE (v4.0) will be used for assessing adverse event severity. Table 5 will be used for assessing severity for adverse events that are not specifically listed in the NCI CTCAE.

"Use of this document is governed by the terms of use on the first page of this document."

Table 5 Adverse Event Severity Grading Scale for Events Not Specifically Listed in NCI CTCAE

Grade	Severity
1	Mild; asymptomatic or mild symptoms; clinical or diagnostic observations only; or intervention not indicated
2	Moderate; minimal, local, or non-invasive intervention indicated; or limiting age-appropriate instrumental activities of daily living [a]
3	Severe or medically significant, but not immediately life-threatening; hospitalization or prolongation of hospitalization indicated; disabling; or limiting self-care activities of daily living [b,c]
4	Life-threatening consequences or urgent intervention indicated [d]
5	Death related to adverse event [d]

NCI CTCAE = National Cancer Institute Common Terminology Criteria for Adverse Events.

Note: Based on the most recent version of NCI CTCAE (v4.0), which can be found at: http://ctep.cancer.gov/protocolDevelopment/electronic_applications/ctc.htm

[a] Instrumental activities of daily living refer to preparing meals, shopping for groceries or clothes, using the telephone, managing money, and so on.

[b] Examples of self-care activities of daily living include bathing, dressing and undressing, feeding oneself, using the toilet, and taking medications, as performed by patients who are not bedridden.

[c] If an event is assessed as a "significant medical event," it must be reported as a serious adverse event (see Section 5.4.2 for reporting instructions), per the definition of serious adverse event in Section 5.2.2.

[d] Grade 4 and 5 events must be reported as serious adverse events (see Section 5.4.2 for reporting instructions) per the definition of serious adverse event in Section 5.2.2.

5.3.4 Assessment of Causality of Adverse Events

Investigators should use their knowledge of the patient, the circumstances surrounding the event, and an evaluation of any potential alternative causes to determine whether an adverse event is considered to be related to study treatment, indicating "yes" or "no" accordingly. The following guidance should be taken into consideration (see also Table 6):

- Temporal relationship of event onset to the initiation of study treatment
- Course of the event, with special consideration of the effects of dose reduction, discontinuation of study treatment, or reintroduction of study treatment (as applicable)
- Known association of the event with study treatment or with similar treatments
- Known association of the event with the disease under study
- Presence of risk factors in the patient or use of concomitant medications known to increase the occurrence of the event
- Presence of non-treatment-related factors that are known to be associated with the occurrence of the event

"Use of this document is governed by the terms of use on the first page of this document."

Table 6 Causal Attribution Guidance

Is the adverse event suspected to be caused by study treatment on the basis of facts, evidence, science-based rationales, and clinical judgment?	
YES	There is a plausible temporal relationship between the onset of the adverse event and administration of study treatment, and the adverse event cannot be readily explained by the patient's clinical state, intercurrent illness, or concomitant therapies; and/or the adverse event follows a known pattern of response to study treatment; and/or the adverse event abates or resolves upon discontinuation of study treatment or dose reduction and, if applicable, reappears upon re-challenge.
NO	An adverse event will be considered related, unless it fulfills the criteria specified below: Evidence exists that the adverse event has an etiology other than study treatment (e.g., preexisting medical condition, underlying disease, intercurrent illness, or concomitant medication); and/or the adverse event has no plausible temporal relationship to administration of study treatment (e.g., cancer diagnosed 2 days after first dose of study treatment).

For patients receiving combination therapy, causality will be assessed individually for each protocol-mandated therapy.

5.3.5 Procedures for Recording Adverse Events

Investigators should use correct medical terminology/concepts when recording adverse events on the Adverse Event eCRF. Avoid colloquialisms and abbreviations.

Only one adverse event term should be recorded in the event field on the Adverse Event eCRF.

5.3.5.1 Infusion-Related Reactions

Adverse events that occur during or within 24 hours after study treatment administration and are judged to be related to study treatment infusion should be captured as a diagnosis (e.g., "infusion-related reaction") on the Adverse Event eCRF. If possible, avoid ambiguous terms such as "systemic reaction." Associated signs and symptoms should be recorded on the dedicated Infusion-Related Reaction eCRF. If a patient experiences both a local and systemic reaction to the same dose of study treatment, each reaction should be recorded separately on the Adverse Event eCRF, with signs and symptoms also recorded separately on the dedicated Infusion-Related Reaction eCRF.

5.3.5.2 Diagnosis versus Signs and Symptoms

For adverse events other than IRRs (see Section 5.3.5.1), a diagnosis (if known) should be recorded on the Adverse Event eCRF rather than individual signs and symptoms (e.g., record only liver failure or hepatitis rather than jaundice, asterixis, and elevated transaminases). However, if a constellation of signs and/or symptoms cannot be medically characterized as a single diagnosis or syndrome at the time of reporting, each

"Use of this document is governed by the terms of use on the first page of this document."

individual event should be recorded on the Adverse Event eCRF. If a diagnosis is subsequently established, all previously reported adverse events based on signs and symptoms should be nullified and replaced by one adverse event report based on the single diagnosis, with a starting date that corresponds to the starting date of the first symptom of the eventual diagnosis.

5.3.5.3 Adverse Events That Are Secondary to Other Events

In general, adverse events that are secondary to other events (e.g., cascade events or clinical sequelae) should be identified by their primary cause, with the exception of severe or serious secondary events. A medically significant secondary adverse event that is separated in time from the initiating event should be recorded as an independent event on the Adverse Event eCRF. For example:

- If vomiting results in mild dehydration with no additional treatment in a healthy adult, only vomiting should be reported on the eCRF.
- If vomiting results in severe dehydration, both events should be reported separately on the eCRF.
- If a severe gastrointestinal hemorrhage leads to renal failure, both events should be reported separately on the eCRF.
- If dizziness leads to a fall and consequent fracture, all three events should be reported separately on the eCRF.
- If neutropenia is accompanied by an infection, both events should be reported separately on the eCRF.

All adverse events should be recorded separately on the Adverse Event eCRF if it is unclear as to whether the events are associated.

5.3.5.4 Persistent or Recurrent Adverse Events

A persistent adverse event is one that extends continuously, without resolution, between patient evaluation timepoints. Such events should only be recorded once on the Adverse Event eCRF. The initial severity (intensity or grade) of the event will be recorded at the time the event is first reported. If a persistent adverse event becomes more severe, the most extreme severity should also be recorded on the Adverse Event eCRF. If the event becomes serious, it should be reported to the Sponsor immediately (i.e., no more than 24 hours after learning that the event became serious; see Section 5.4.2 for reporting instructions). The Adverse Event eCRF should be updated by changing the event from "non-serious" to "serious," providing the date that the event became serious, and completing all data fields related to serious adverse events.

A recurrent adverse event is one that resolves between patient evaluation timepoints and subsequently recurs. Each recurrence of an adverse event should be recorded as a separate event on the Adverse Event eCRF.

"Use of this document is governed by the terms of use on the first page of this document."

5.3.5.5 Abnormal Laboratory Values

Not every laboratory abnormality qualifies as an adverse event. A laboratory test result must be reported as an adverse event if it meets any of the following criteria:

- Is accompanied by clinical symptoms
- Results in a change in study treatment (e.g., dosage modification, treatment interruption, or treatment discontinuation)
- Results in a medical intervention (e.g., potassium supplementation for hypokalemia) or a change in concomitant therapy
- Is clinically significant in the investigator's judgment

  Note: For oncology trials, certain abnormal values may not qualify as adverse events.

It is the investigator's responsibility to review all laboratory findings. Medical and scientific judgment should be exercised in deciding whether an isolated laboratory abnormality should be classified as an adverse event.

If a clinically significant laboratory abnormality is a sign of a disease or syndrome (e.g., alkaline phosphatase and bilirubin $5 \times$ ULN associated with cholestasis), only the diagnosis (i.e., cholestasis) should be recorded on the Adverse Event eCRF.

If a clinically significant laboratory abnormality is not a sign of a disease or syndrome, the abnormality itself should be recorded on the Adverse Event eCRF, along with a descriptor indicating whether the test result is above or below the normal range (e.g., "elevated potassium," as opposed to "abnormal potassium"). If the laboratory abnormality can be characterized by a precise clinical term per standard definitions, the clinical term should be recorded as the adverse event. For example, an elevated serum potassium level of 7.0 mEq/L should be recorded as "hyperkalemia."

Observations of the same clinically significant laboratory abnormality from visit to visit should only be recorded once on the Adverse Event eCRF (see Section 5.3.5.4 for details on recording persistent adverse events).

5.3.5.6 Abnormal Vital Sign Values

Not every vital sign abnormality qualifies as an adverse event. A vital sign result must be reported as an adverse event if it meets any of the following criteria:

- Is accompanied by clinical symptoms
- Results in a change in study treatment (e.g., dosage modification, treatment interruption, or treatment discontinuation)
- Results in a medical intervention or a change in concomitant therapy
- Is clinically significant in the investigator's judgment

"Use of this document is governed by the terms of use on the first page of this document."

It is the investigator's responsibility to review all vital sign findings. Medical and scientific judgment should be exercised in deciding whether an isolated vital sign abnormality should be classified as an adverse event.

If a clinically significant vital sign abnormality is a sign of a disease or syndrome (e.g., high blood pressure), only the diagnosis (i.e., hypertension) should be recorded on the Adverse Event eCRF.

Observations of the same clinically significant vital sign abnormality from visit to visit should only be recorded once on the Adverse Event eCRF (see Section 5.3.5.4 for details on recording persistent adverse events).

5.3.5.7 Abnormal Liver Function Tests

The finding of an elevated ALT or AST ($> 3 \times$ ULN) in combination with either an elevated total bilirubin ($> 2 \times$ ULN) or clinical jaundice in the absence of cholestasis or other causes of hyperbilirubinemia is considered to be an indicator of severe liver injury (as defined by Hy's Law). Therefore, investigators must report as an adverse event the occurrence of either of the following:

- Treatment-emergent ALT or AST $> 3 \times$ ULN (or baseline value if baseline value was above the ULN) in combination with total bilirubin $> 2 \times$ ULN (of which $\geq$35% is direct bilirubin)
- Treatment-emergent ALT or AST $> 3 \times$ ULN (or baseline value if baseline value was above the ULN) in combination with clinical jaundice

The most appropriate diagnosis or (if a diagnosis cannot be established) the abnormal laboratory values should be recorded on the Adverse Event eCRF (see Section 5.3.5.2) and reported to the Sponsor immediately (i.e., no more than 24 hours after learning of the event), either as a serious adverse event or an adverse event of special interest (see Section 5.4.2).

5.3.5.8 Deaths

For this protocol, mortality is an efficacy endpoint. Deaths that occur during the protocol-specified adverse event reporting period (see Section 5.3.1) that are attributed by the investigator solely to recurrence and progression of breast cancer should be recorded on the Death Attributed to Progressive Disease eCRF. All other on-study deaths, regardless of relationship to study treatment, must be recorded on the Adverse Event eCRF and immediately reported to the Sponsor (see Section 5.4.2). An independent monitoring committee will monitor the frequency of deaths from all causes.

Death should be considered an outcome and not a distinct event. The event or condition that caused or contributed to the fatal outcome should be recorded as the single medical concept on the Adverse Event eCRF. Generally, only one such event should be reported. If the cause of death is unknown and cannot be ascertained at the time of reporting, **"unexplained death"** should be recorded on the Adverse Event eCRF. If the

"Use of this document is governed by the terms of use on the first page of this document."

cause of death later becomes available (e.g., after autopsy), "unexplained death" should be replaced by the established cause of death. The term "sudden death" should not be used unless combined with the presumed cause of death (e.g., "sudden cardiac death").

Deaths that occur after the adverse event reporting period should be reported as described in Section 5.6.

5.3.5.9 Preexisting Medical Conditions

A preexisting medical condition is one that is present at the screening visit for this study. Such conditions should be recorded on the General Medical History and Baseline Conditions eCRF.

A preexisting medical condition should be recorded as an adverse event only if the frequency, severity, or character of the condition worsens during the study. When recording such events on the Adverse Event eCRF, it is important to convey the concept that the preexisting condition has changed by including applicable descriptors (e.g., "more frequent headaches").

5.3.5.10 Lack of Efficacy or Worsening of TNBC

Events that are clearly consistent with the expected pattern of progression of the underlying disease should not be recorded as adverse events. These data will be captured as efficacy assessment data only. Instances of disease recurrence will be verified by pathologic confirmation if clinically feasible. In rare cases, the determination of clinical progression will be based on symptomatic deterioration. However, every effort should be made to document progression through use of objective criteria. If there is any uncertainty as to whether an event is due to disease progression, it should be reported as an adverse event.

5.3.5.11 Hospitalization or Prolonged Hospitalization

Any adverse event that results in hospitalization (i.e., inpatient admission to a hospital) or prolonged hospitalization should be documented and reported as a serious adverse event (per the definition of serious adverse event in Section 5.2.2), except as outlined below.

An event that leads to hospitalization under the following circumstances should not be reported as an adverse event or a serious adverse event:

- Hospitalization for respite care
- Planned hospitalization required by the protocol (e.g., for study treatment administration or performance of an efficacy measurement for the study)

"Use of this document is governed by the terms of use on the first page of this document."

- Hospitalization for a preexisting condition, provided that all of the following criteria are met:

    The hospitalization was planned prior to the study or was scheduled during the study when elective surgery became necessary because of the expected normal progression of the disease.

    The patient has not experienced an adverse event.

- Hospitalization due solely to recurrence/progression of the underlying cancer

An event that leads to hospitalization under the following circumstances is not considered to be a serious adverse event, but should be reported as an adverse event instead:

- Hospitalization that was necessary because of patient requirement for outpatient care outside of normal outpatient clinic operating hours

5.3.5.12 Cases of Accidental Overdose or Medication Error

Accidental overdose and medication error (hereafter collectively referred to as "special situations"), are defined as follows:

- Accidental overdose: accidental administration of a drug in a quantity that is higher than the assigned dose
- Medication error: accidental deviation in the administration of a drug

    In some cases, a medication error may be intercepted prior to administration of the drug.

Special situations are not in themselves adverse events but may result in adverse events. Each adverse event associated with a special situation should be recorded separately on the Adverse Event eCRF. If the associated adverse event fulfills seriousness criteria, the event should be reported to the Sponsor immediately (i.e., no more than 24 hours after learning of the event; see Section 5.4.2).
For atezolizumab and nab-paclitaxel, adverse events associated with special situations should be recorded as described below for each situation:

- Accidental overdose: Enter the adverse event term. Check the "Accidental overdose" and "Medication error" boxes.
- Medication error that does not qualify as an overdose: Enter the adverse event term. Check the "Medication error" box.
- Medication error that qualifies as an overdose: Enter the adverse event term. Check the "Accidental overdose" and "Medication error" boxes.

In addition, all special situations associated with atezolizumab and nab-paclitaxel, regardless of whether they result in an adverse event, should be recorded on the Adverse Event eCRF as described below:

- Accidental overdose: Enter the drug name and "accidental overdose" as the event term. Check the "Accidental overdose" and "Medication error" boxes.

"Use of this document is governed by the terms of use on the first page of this document."

- Medication error that does not qualify as an overdose: Enter the name of the drug administered and a description of the error (e.g., wrong dose administered, wrong dosing schedule, incorrect route of administration, wrong drug, expired drug administered) as the event term. Check the "Medication error" box.
- Medication error that qualifies as an overdose: Enter the drug name and "accidental overdose" as the event term. Check the "Accidental overdose" and "Medication error" boxes. Enter a description of the error in the additional case details.
- Intercepted medication error: Enter the drug name and "intercepted medication error" as the event term. Check the "Medication error" box. Enter a description of the error in the additional case details.

As an example, an accidental overdose that resulted in a headache would require the completion of two Adverse Event eCRF pages, one to report the accidental overdose and one to report the headache. The "Accidental overdose" and "Medication error" boxes would need to be checked on both eCRF pages.

5.3.5.13 Patient-Reported Outcome Data

Adverse event reports will not be derived from PRO data by the Sponsor, and safety analyses will not be performed using PRO data. However, if any PRO responses suggestive of a possible adverse event are identified during site review of the PRO data, the investigator will determine whether the criteria for an adverse event have been met and, if so, will report the event on the Adverse Event eCRF.

5.4 IMMEDIATE REPORTING REQUIREMENTS FROM INVESTIGATOR TO SPONSOR

Certain events require immediate reporting to allow the Sponsor to take appropriate measures to address potential new risks in a clinical trial. The investigator must report such events to the Sponsor immediately; under no circumstances should reporting take place more than 24 hours after the investigator learns of the event. The following is a list of events that the investigator must report to the Sponsor within 24 hours after learning of the event, regardless of relationship to study treatment:

- Serious adverse events (see Section 5.4.2 for further details)
- Adverse events of special interest (see Section 5.4.2 for further details)
- Pregnancies (see Section 5.4.3 for further details)

For serious adverse events and adverse events of special interest, the investigator must report new significant follow-up information for these events to the Sponsor immediately (i.e., no more than 24 hours after becoming aware of the information). New significant information includes the following:

- New signs or symptoms or a change in the diagnosis
- Significant new diagnostic test results
- Change in causality based on new information

"Use of this document is governed by the terms of use on the first page of this document."

- Change in the event's outcome, including recovery
- Additional narrative information on the clinical course of the event

Investigators must also comply with local requirements for reporting serious adverse events to the local health authority and IRB/EC.

5.4.1 Emergency Medical Contacts

Medical Monitor Contact Information for All Sites

Primary Medical Monitor: [redacted], *M.D.*

Mobile Telephone No./SMS: [redacted]

Secondary Medical Monitor/
Roche Medical Responsible: [redacted], M.D.

Telephone No.: [redacted]

Mobile Telephone No./SMS: [redacted]

To ensure the safety of study patients, an Emergency Medical Call Center Help Desk will access the Roche Medical Emergency List, escalate emergency medical calls, provide medical translation service (if necessary), connect the investigator with a Roche Medical Responsible (listed above and/or on the Roche Medical Emergency List), and track all calls. The Emergency Medical Call Center Help Desk will be available 24 hours per day, 7 days per week. Toll-free numbers for the Help Desk, as well as Medical Monitor and Medical Responsible contact information, will be distributed to all investigators.

5.4.2 Reporting Requirements for Serious Adverse Events and Adverse Events of Special Interest

5.4.2.1 Events That Occur prior to Study Treatment Initiation

After informed consent has been obtained but prior to initiation of study treatment, only serious adverse events caused by a protocol-mandated intervention should be reported. The Serious Adverse Event/Adverse Event of Special Interest Reporting Form provided to investigators should be completed and submitted to the Sponsor or its designee immediately (i.e., no more than 24 hours after learning of the event), either by faxing or by scanning and emailing the form using the fax number or email address provided to investigators.

5.4.2.2 Events That Occur after Study Treatment Initiation

After initiation of study treatment, all adverse events will be reported until 30 days after the last dose of study treatment or until initiation of new systemic anti-cancer therapy, whichever occurs first, and serious adverse events and adverse events of special interest will be reported until 90 days after the last dose of study treatment or until initiation of new systemic anti-cancer therapy, whichever occurs first. Investigators should record all case details that can be gathered immediately (i.e., within 24 hours

"Use of this document is governed by the terms of use on the first page of this document."

after learning of the event) on the Adverse Event eCRF and submit the report via the EDC system. A report will be generated and sent to Roche Safety Risk Management by the EDC system.

In the event that the EDC system is unavailable, the Serious Adverse Event/Adverse Event of Special Interest Reporting Form provided to investigators should be completed and submitted to the Sponsor or its designee immediately (i.e., no more than 24 hours after learning of the event), either by faxing or by scanning and emailing the form using the fax number or email address provided to investigators. Once the EDC system is available, all information will need to be entered and submitted via the EDC system.

Instructions for reporting serious adverse events that occur > 90 days after the last dose of study treatment are provided in Section 5.6.

5.4.3 Reporting Requirements for Pregnancies

5.4.3.1 Pregnancies in Female Patients

Female patients of childbearing potential will be instructed to immediately inform the investigator if they become pregnant during the study or within 5 months after the last dose of atezolizumab, 1 month after the last dose of nab-paclitaxel, 6 months after the last dose of doxorubicin, or 12 months after the last dose of cyclophosphamide, whichever is later. A Clinical Trial Pregnancy Reporting Form should be completed and submitted to the Sponsor or its designee immediately (i.e., no more than 24 hours after learning of the pregnancy), either by faxing or by scanning and emailing the form using the fax number or email address provided to investigators. Pregnancy should not be recorded on the Adverse Event eCRF. The investigator should discontinue study treatment and counsel the patient, discussing the risks of the pregnancy and the possible effects on the fetus. Monitoring of the patient should continue until conclusion of the pregnancy. Any serious adverse events associated with the pregnancy (e.g., an event in the fetus, an event in the mother during or after the pregnancy, or a congenital anomaly/birth defect in the child) should be reported on the Adverse Event eCRF. In addition, the investigator will submit a Clinical Trial Pregnancy Reporting Form when updated information on the course and outcome of the pregnancy becomes available.

5.4.3.2 Pregnancies in Female Partners of Male Patients

Male patients will be instructed through the Informed Consent Form to immediately inform the investigator if their partner becomes pregnant during the study or within 6 months after the last dose of nab-paclitaxel, cyclophosphamide, or doxorubicin. A Clinical Trial Pregnancy Reporting Form should be completed and submitted to the Sponsor or its designee immediately (i.e., no more than 24 hours after learning of the pregnancy), either by faxing or by scanning and emailing the form using the fax number or email address provided to investigators. Attempts should be made to collect and report details of the course and outcome of any pregnancy in the partner of a male patient exposed to study treatment. When permitted by the site, the pregnant partner would need to sign an Authorization for Use and Disclosure of Pregnancy Health

"Use of this document is governed by the terms of use on the first page of this document."

information to allow for follow-up on her pregnancy. If the authorization has been signed, the investigator should submit a Clinical Trial Pregnancy Reporting Form when updated information on the course and outcome of the pregnancy becomes available. An investigator who is contacted by the male patient or his pregnant partner may provide information on the risks of the pregnancy and the possible effects on the fetus, to support an informed decision in cooperation with the investigator and/or obstetrician.

5.4.3.3 Abortions

A spontaneous abortion should be classified as a serious adverse event (as the Sponsor considers abortions to be medically significant), recorded on the Adverse Event eCRF, and reported to the Sponsor immediately (i.e., no more than 24 hours after learning of the event; see Section 5.4.2).

If a therapeutic or elective abortion was performed because of an underlying maternal or embryofetal toxicity, the toxicity should be classified as a serious adverse event, recorded on the Adverse Event eCRF, and reported to the Sponsor immediately (i.e., no more than 24 hours after learning of the event; see Section 5.4.1). A therapeutic or elective abortion performed for reasons other than an underlying maternal or embryofetal toxicity is not considered an adverse event.

All abortions should be reported as pregnancy outcomes on the paper Clinical Trial Pregnancy Reporting Form.

5.4.3.4 Congenital Anomalies/Birth Defects

Any congenital anomaly/birth defect in a child born to a female patient exposed to study treatment or the female partner of a male patient exposed to study treatment should be classified as a serious adverse event, recorded on the Adverse Event eCRF, and reported to the Sponsor immediately (i.e., no more than 24 hours after learning of the event; see Section 5.4.2).

5.5 FOLLOW-UP OF PATIENTS AFTER ADVERSE EVENTS

5.5.1 Investigator Follow-Up

The investigator should follow each adverse event until the event has resolved to baseline grade or better, the event is assessed as stable by the investigator, the patient is lost to follow-up, or the patient withdraws consent. Every effort should be made to follow all serious adverse events considered to be related to study treatment or trial-related procedures until a final outcome can be reported.

During the study period, resolution of adverse events (with dates) should be documented on the Adverse Event eCRF and in the patient's medical record to facilitate source data verification.

All pregnancies reported during the study should be followed until pregnancy outcome.

"Use of this document is governed by the terms of use on the first page of this document."

5.5.2 Sponsor Follow-Up

For serious adverse events, adverse events of special interest, and pregnancies, the Sponsor or a designee may follow up with the investigator by telephone, fax, electronic mail, and/or a monitoring visit to obtain additional case details and outcome information (e.g., from hospital discharge summaries, consultant reports, autopsy reports) in order to perform an independent medical assessment of the reported case.

5.6 ADVERSE EVENTS THAT OCCUR AFTER THE ADVERSE EVENT REPORTING PERIOD

After the end of the reporting period for all serious adverse events and adverse events of special interest (defined as 90 days after the last dose of study treatment), all deaths, regardless of cause, should be reported through use of the Long-Term Survival Follow-Up eCRF.

In addition, if the investigator becomes aware of a serious adverse event that is believed to be related to prior exposure to study treatment, the event should be reported through use of the Adverse Event eCRF. However, if the EDC system is not available, the investigator should report these events directly to the Sponsor or its designee, either by faxing or by scanning and emailing the Serious Adverse Event/Adverse Event of Special Interest Reporting Form using the fax number or email address provided to investigators.

5.7 EXPEDITED REPORTING TO HEALTH AUTHORITIES, INVESTIGATORS, INSTITUTIONAL REVIEW BOARDS, AND ETHICS COMMITTEES

The Sponsor will promptly evaluate all serious adverse events and adverse events of special interest against cumulative product experience to identify and expeditiously communicate possible new safety findings to investigators, IRBs, ECs, and applicable health authorities based on applicable legislation.

To determine reporting requirements for single adverse event cases, the Sponsor will assess the expectedness of these events using the following reference documents:

- Atezolizumab Investigator's Brochure
- Local prescribing information (e.g., Section 4.8 of E.U. Summary of Product Characteristics for nab-paclitaxel)

The Sponsor will compare the severity of each event and the cumulative event frequency reported for the study with the severity and frequency reported in the applicable reference document.

Reporting requirements will also be based on the investigator's assessment of causality and seriousness, with allowance for upgrading by the Sponsor as needed.

"Use of this document is governed by the terms of use on the first page of this document."

An iDMC will monitor the incidence of these expected events during the study. An aggregate report of any clinically relevant imbalances that do not favor the test product will be submitted to health authorities.

6. STATISTICAL CONSIDERATIONS AND ANALYSIS PLAN

The primary analysis population for efficacy is the ITT population, defined as all randomized patients, and the PD-L1–positive subpopulation, defined as patients in the ITT population whose PD-L1 status is IC1/2/3 at the time of randomization. Patients will be assigned to the treatment group to which they were randomized.

The PRO-evaluable population is defined as patients in the ITT population with baseline PRO assessment.

The primary analysis population for safety is the safety-evaluable population, defined as all patients who received at least one dose of study medication. Patients will be assigned to treatment groups as treated, and all patients who received any dose of atezolizumab will be included in the atezolizumab treatment arm.

6.1 DETERMINATION OF SAMPLE SIZE

The study will first randomize approximately N1 = 204 patients in Stage 1 (1:1 randomization ratio). Based on information from these patients and a pre-specified adaptive rule, the decision will be made regarding whether or not to randomize approximately N2 = 120 patients in Stage 2 (1:1 randomization ratio).

Details of the adaptive design as well as the statistical procedures to control the overall one-sided type I error at $\alpha = 0.025$ are described in the sections below. Additional details are provided in Appendix 14 and Appendix 15.

6.1.1 Adaptive Design

The design is a two-stage adaptive enrichment design for the co-primary endpoints of pCR in the ITT (F) and PD-L1–postive (S) population at the overall one-sided significance level $\alpha = 0.025$. The design closely follows the general statistical principles for adaptive enrichment designs described in Wassmer et al. 2016.

The study will first randomize approximately $N_1 = 204$ patients in Stage 1 (1:1 randomization ratio). Based on information from these patients and a pre-specified adaptive rule, one of the following decisions will be taken:

1. Not initiate Stage 2 due to efficacy in F, S, or both populations.
2. Not initiate Stage 2 due to lack of efficacy in both F and S.
3. Continue the trial to Stage 2. In Stage 2, another approximately $N_2 = 120$ patients will be randomized (1:1 randomization ratio). Based on data from Stage 1, a decision will be taken to either randomize patients from the ITT (F) or from the PD-L1–positive (S) population only in Stage 2.

"Use of this document is governed by the terms of use on the first page of this document."

This design has two sources of multiplicity: a) Two co-primary populations and b) two analyses (Stage 1 data only, Stage 1 and Stage 2 data combined). To protect the familywise one-sided type I error of the trial (α) for this study at 0.025 in the strong sense, the co-primary endpoints will be tested using Simes' method at each stage (Wassmer et al. 2016) and p-values will be combined across stages using the weighted inverse normal combination test with pre-defined weights (Wassmer et al. 2016) $w_1 = \sqrt{N_1/(N_1+N_2)}$ and $w_2 = \sqrt{N_2/(N_1+N_2)}$ for Stage 1 and Stage 2, respectively, with $N_1 = 204$ and $N_2 = 120$ denoting the planned sample sizes for both stages. Of note, the planned sample size rather than the actual number of recruited patients will be used for the determination of the weights to protect type I error. If $p_1$and p_2 denote the p-values based on Stage 1 or Stage 2 data only (for the null hypotheses $H_0^{\{F\}}$, $H_0^{\{S\}}$ or the intersection null hypothesis $H_0^{\{F,S\}}$, respectively), then the combination p-value is defined as $p_{comb} = 1 - \Phi\{w_1 \cdot \Phi^{-1}(1-p_1) + w_2 \cdot \Phi^{-1}(1-p_2)\}$ (Wassmer et al. 2016).

Further details regarding the testing procedure are provided in Appendix 14 and Appendix 15, as well as the Statistical Analysis Plan.

6.1.2 Type I Error Control

The pre-defined one-sided type I error for the interim analysis at Stage 1 is α_1=0.0125 (i.e., 50% of the total type I error). The co-primary endpoints in F and S at Stage 1 will be tested using a closed testing procedure using Simes' test for the intersection hypothesis. As stated in Wassmer et al. 2016, test statistics in F and S are always positively correlated, hence type I error rate control of the Simes test can be guaranteed in general.

Importantly, the p-value p_1 based on Stage 1 data only and the combination p-value p_{comb} have the same joint distribution under the null hypothesis as the p-values from a group-sequential test with two stages at information times $t_1 = w_1$ and $t_2 = 1$. Thus standard statistical software for group sequential designs can be used for the determination of critical values for the adaptive design (Wassmer et al. 2016). As the study design uses a critical value of α_1=0.0125 (i.e., 50% of the total type I error) for Stage 1, this implies that a critical value of α_{comb}=0.0184 can be applied to the combination p-values.

6.2 SUMMARIES OF CONDUCT OF STUDY

For all randomized patients (i.e., ITT population), a participant flowchart for depicting the progress of subjects through the phases of the trial will be provided by treatment arm, including a complete description of patient disposition specifying the number of randomized patients and completed and discontinued patients from trial treatment and study with reasons for premature discontinuation. Documented protocol deviations including those related to study inclusion/exclusion criteria, conduct of the study, patient management, or patient assessment will be also tabulated by treatment arm.

"Use of this document is governed by the terms of use on the first page of this document."

6.3 SUMMARIES OF TREATMENT GROUP COMPARABILITY

Demographic variables such as age, sex, race/ethnicity, and baseline characteristics (in particular, stratification variables) will be summarized by treatment arm for the ITT as well as PD-L1–positive population. Only descriptive analyses are planned; no formal statistical tests will be applied. Continuous variables will be reported and summarized by use of standard measures of central tendency and dispersion (mean, standard deviation, median, and range including minimum and maximum), and categorical (i.e., discrete) data will be reported and summarized by frequencies and percentages.

The baseline value of any variable will be defined as the last available value prior to the first administration of study treatment.

6.4 EFFICACY ANALYSES

Details of the analyses, including methods for handling missing data, are specified in the Statistical Analysis Plan (SAP).

6.4.1 Primary Efficacy Endpoint: Pathologic Complete Response

The primary efficacy objective for this study is to evaluate the efficacy of neoadjuvant atezolizumab + nab-pac–AC compared with placebo + nab-pac–AC in patients with T2-4d TNBC, as measured by pCR defined as eradication of tumor from both breast and lymph nodes (ypT0/is ypN0). The primary efficacy endpoint will be established following completion of neoadjuvant therapy and surgery.

The ITT as well as PD-L1–positive populations will be used for the primary analysis of pCR. In the primary analysis, patients whose pCR assessment was missing will be counted as not achieving a pCR. An estimate of the pCR rate and its 95% CI (Clopper and Pearson 1934) will be calculated for each treatment arm. The difference in pCR rates will be provided with 95% CI, using the normal approximation to the binomial distribution. For the ITT population, the Cochran-Mantel-Haenszel χ^2 test stratified according to tumor PD-L1 status (IC0 vs. IC1/2/3) and clinical stage at presentation (Stage II vs. III) will be used to test pCR rates between treatment groups at a two-sided significance level of 5%. For the PD-L1–positive population, similar test will be used with stratification only for clinical stage at presentation. An unstratified χ^2 versions of these tests will also be provided as a sensitivity analysis.

6.4.2 Secondary Efficacy Endpoints

The secondary efficacy endpoints are the following:

- EFS in all patients (ITT population) and in the subpopulation with PD-L1–positive tumor status
- DFS in all patients (ITT population) who undergo surgery and in the subpopulation of patients with PD-L1-positive tumor status who undergo surgery

"Use of this document is governed by the terms of use on the first page of this document."

- OS in all patients (ITT population) and in the PD-L1−positive subpopulation
- PROs of function (role, physical) and HRQoL

6.4.2.1 Event-Free Survival

EFS is defined as the time from randomization to the first documented occurrence of disease recurrence, disease progression, or death from any cause.

Patients without an event at the time of the analysis will be censored on the date on which they are last known to be alive and event free, on or before the clinical data cutoff date for the respective analysis. Patients with no post-baseline information will be censored at the date of randomization.

EFS will be compared between treatment arms with an unstratified log-rank test. The HR for EFS will be estimated using a stratified Cox proportional hazards model. The 95% CI for the HR will be provided. Kaplan-Meier methodology will be used to estimate the median EFS (if reached) for each treatment arm, and Kaplan-Meier curves will be produced. Brookmeyer-Crowley methodology will be used to construct the 95% CI for the median EFS for each treatment arm (Brookmeyer and Crowley 1982). The Kaplan-Meier approach will be used to estimate 3-year EFS rates and corresponding 95% CIs for each treatment arm.

The stratification factors for all analyses based on the ITT population will be: AJCC disease stage (II vs. III) and PD-L1 status (IC0 vs. IC1/2/3).

The stratification factors for EFS analysis in the PD-L1−positive subpopulation will be AJCC disease stage (II vs. III).

Results from unstratified analyses will also be provided.

6.4.2.2 Disease-Free Survival

DFS is defined as the time from surgery (i.e., the first date of no disease) to the first documented disease recurrence or death from any cause, whichever occurs first. DFS will be analyzed with the use of the same methodology as specified for EFS for both the ITT population and the PD-L1−positive subpopulation. The DFS analysis will be performed approximately 36 months after the randomization of the last patient.

Patients who do not undergo surgery at the end of neoadjuvant treatment will be excluded from the analysis of DFS. Patients without a DFS event at the time of analysis will be censored at the date when they were last known to be alive and event free. Patients who do not have information after surgery will be censored at the date of surgery.

6.4.2.3 Overall Survival

OS is defined as the time from the date of randomization to the date of death due to any cause. Patients who are not reported as having died at the time of analyses will be

"Use of this document is governed by the terms of use on the first page of this document."

censored at the date when they were last known to be alive. Patients who do not have information after baseline will be censored at the date of randomization. OS will be analyzed with the use of the same methodology as specified for EFS for both the ITT population and the PD-L1–positive subpopulation.

6.4.2.4 Patient-Reported Outcomes of Role and Physical Function and GHS/HRQoL—EORTC Data

The primary patient-reported endpoints are mean and mean changes from baseline score in function (role, physical) and GHS/HRQoL. Summary statistics (mean, standard deviation, median, and range) of absolute scores and mean changes from baseline will be calculated for the functional (role [Question {Q}6, Q7], physical [Q1–Q5]) and the GHS/HRQoL (Q29, Q30) scales of the EORTC QLQ-C30 at each assessment timepoint for each arm. The mean (and 95% CI) and median of the absolute scores and the changes from baseline will be reported for interval and continuous variables. Previously published minimally important differences will be used to identify meaningful change from baseline within each treatment group on the functional and GHS/HRQoL scales (Osoba et al. 1998; Cocks et al. 2011).

The EORTC QLQ-C30 (Version 3) data will be scored according to the EORTC scoring manual (Fayers et al. 2001). PRO completion, compliance rates, and reasons for missing data will be summarized at each timepoint by treatment arm. Details of the analyses, including methods for handling missing data, will be specified in the SAP.

6.4.3 Exploratory Efficacy Endpoints

The analyses of proportion of patients undergoing breast-conserving surgery, RCB index, and correlation of RCB with other clinical endpoints (if deemed appropriate) will be detailed in the SAP.

6.4.3.1 Patient-Reported Outcomes of Disease/Treatment-Related Symptoms, Emotional and Social Function—EORTC Data

Summary statistics (mean, standard deviation, median, and range) of absolute scores and mean changes from baseline will be calculated for all disease/treatment-related symptom items and scales, and the emotional, social function scales of the EORTC QLQ-C30 (see Appendix 5) at each assessment timepoint for each arm.

6.4.3.2 Patient-Reported Outcome of Treatment Bother—FACT-G, GP5 Single Item Data

A descriptive analysis of absolute scores and the proportion of patients selecting each response option at each assessment timepoint by treatment arm will be reported for item GP5 ("I am bothered by side effects of treatment") from the FACT-G physical well-being subscale (see Appendix 6). Item GP5 from version 4 of the FACT-G questionnaire will be scored according to the FACIT scoring manual (Cella 1997).

"Use of this document is governed by the terms of use on the first page of this document."

6.4.3.3 Health Economic EQ-5D-5L Data

Health utility data from the EQ-5D-5L (see Appendix 7) will be evaluated in pharmacoeconomic models. The results from the health economic data analyses will be reported separately from the clinical study report.

6.5 SAFETY ANALYSES

Safety analyses will be performed on the safety population.

Safety will be assessed through summaries of adverse events, changes in laboratory test results, changes in vital signs, study treatment exposures, and immunogenicity as measured by anti-drug antibodies (ADAs) and will be presented by treatment arm.

Verbatim descriptions of adverse events will be mapped to Medical Dictionary for Regulatory Activities (MedDRA) thesaurus terms and graded according to NCI CTCAE v4.0.

Adverse events will be summarized by mapped term and appropriate thesaurus level, and adverse event severity will be graded according to NCI CTCAE v4.0.

The treatment-emergent adverse events leading to withdrawal of study drug, leading to dose reduction or interruption, related to study drug, severe (i.e., Grade $\geq$3), fatal (i.e., Grade 5), serious, and of special interest will be also summarized.

All deaths and causes of death will be summarized.

Laboratory data with values outside of the normal ranges will be identified. Relevant laboratory values will be summarized by treatment arm over time, with NCI CTCAE v4.0 Grade 3 and Grade 4 values identified, where appropriate. Changes from baseline in NCI CTCAE v4.0 grade (i.e., shift tables) will be also provided by treatment arm. Of note, abnormal laboratory data that are clinically significant will be reported as adverse events and summarized in the adverse event tables.

Vital signs and other physical findings will be summarized by treatment arm.

6.6 PHARMACOKINETIC ANALYSES

Atezolizumab serum concentration data (C_{min} and C_{max}) will be tabulated and summarized. Descriptive statistics will include means, medians, ranges, SDs, coefficient of variation (%CV), and others as appropriate.

Additional PK and PD analyses will be conducted as appropriate.

6.7 IMMUNOGENICITY ANALYSES

The immunogenicity analyses will include patients with at least one post-baseline ADA assessment, with patients grouped according to treatment received.

"Use of this document is governed by the terms of use on the first page of this document."

The numbers and proportions of ADA-positive patients and ADA-negative patients at baseline (baseline prevalence) and after baseline (post-baseline incidence) will be summarized by treatment group. When determining post-baseline incidence, patients are considered to be ADA positive if they are ADA negative or are missing data at baseline but develop an ADA response following study drug exposure (treatment-induced ADA response), or if they are ADA positive at baseline and the titer of one or more post-baseline samples is at least 0.60 titer unit greater than the titer of the baseline sample (treatment-enhanced ADA response). Patients are considered to be ADA negative if they are ADA negative or are missing data at baseline and all post-baseline samples are negative, or if they are ADA positive at baseline but do not have any post-baseline samples with a titer that is at least 0.60 titer unit greater than the titer of the baseline sample (treatment unaffected).

The relationship between ADA status and safety, efficacy, and pharmacokinetics may be investigated.

6.8 BIOMARKER ANALYSES

Exploratory biomarker analyses will be performed in baseline pretreatment, on-treatment, residual-disease, and post-recurrence samples in an effort to understand the association of these markers with study treatment outcome, including efficacy and/or adverse events. The biomarkers may include but will not be limited to PD-L1 and other biomarkers in tumor and blood, as defined by IHC, quantitative reverse transcription PCR, NGS, or other methods. Results will be presented in a separate report.

WGS data will be analyzed in the context of this study and explored in aggregate with data from other studies to increase researchers' understanding of disease pathobiology and guide the development of new therapeutic approaches.

6.9 INTERIM ANALYSES

6.9.1 Planned Interim Analysis

Up to a total of two analyses of pCR endpoints will be performed.

An interim efficacy analysis will be initiated for this study after all of the patients enrolled in Stage 1 (approximately 204) have received surgery or discontinued early from the neo-adjuvant phase.

The interim analysis will be performed by an independent Data Coordinating Center (iDCC) and evaluated by an independent Data Monitoring Committee (iDMC). Depending on iDMC recommendation at the interim analysis, the study could continue enrolling approximately 120 patients in Stage 2 and primary efficacy pCR analysis will then be conducted after all of these patients have received surgery or discontinued early from the neo-adjuvant phase. The decision rules to be applied at the interim analysis will be clearly expressed to the iDMC and documented in the iDMC charter, as well as Appendix 14 and the Statistical Analysis Plan, so that the study can be conducted with

"Use of this document is governed by the terms of use on the first page of this document."

the Sponsor remaining completely blinded to all results at this stage. Provisions will be in place to ensure the study continues to meet the highest standards of integrity when an optional interim analysis is executed. Interactions between the iDMC and Sponsor will be carried out as specified in the iDMC charter.

The boundaries for statistical significance at the interim and primary analysis for pCR, as well as methods to control for the overall type I error are discussed in Section 6.1.2.

6.9.2 Optional Interim Analysis

To adapt to information that may emerge during the course of this study,the Sponsor may choose to conduct optional interim analyses before the final analysis for EFS, DFS, and OS, if needed (e.g., for regulatory or publication purposes). The decision to conduct an optional interim analysis, along with the rationale, timing, and statistical details for the analysis, will be documented in the Sponsor's trial master file prior to the conduct of the interim analysis.

7. DATA COLLECTION AND MANAGEMENT

7.1 DATA QUALITY ASSURANCE

The Sponsor will be responsible for data management of this study, including quality checking of the data. Data entered manually will be collected via EDC through use of eCRFs. Sites will be responsible for data entry into the EDC system. In the event of discrepant data, the Sponsor will request data clarification from the sites, which the sites will resolve electronically in the EDC system.

The Sponsor will produce an EDC Study Specification document that describes the quality checking to be performed on the data. Central laboratory data and any other externally generated electronic study data will be sent directly to the Sponsor, using the Sponsor's standard procedures to handle and process the electronic transfer of these data.

eCRFs and correction documentation will be maintained in the EDC system's audit trail. System backups for data stored by the Sponsor and records retention for the study data will be consistent with the Sponsor's standard procedures.

PRO data will be collected on paper questionnaires. The data from the questionnaires will be entered into the EDC system by site staff.

7.2 ELECTRONIC CASE REPORT FORMS

eCRFs are to be completed through use of a Sponsor-designated EDC system. Sites will receive training and have access to a manual for appropriate eCRF completion. eCRFs will be submitted electronically to the Sponsor and should be handled in accordance with instructions from the Sponsor.

"Use of this document is governed by the terms of use on the first page of this document."

All eCRFs should be completed by designated, trained site staff. eCRFs should be reviewed and electronically signed and dated by the investigator or a designee.

At the end of the study, the investigator will receive patient data for his or her site in a readable format on a compact disc that must be kept with the study records. Acknowledgement of receipt of the compact disc is required.

7.3 SOURCE DATA DOCUMENTATION

Study monitors will perform ongoing source data verification and review to confirm that critical protocol data (i.e., source data) entered into the eCRFs by authorized site personnel are accurate, complete, and verifiable from source documents.

Source documents (paper or electronic) are those in which patient data are recorded and documented for the first time. They include, but are not limited to, hospital records, clinical and office charts, laboratory notes, memoranda, PRO questionnaires, evaluation checklists, pharmacy dispensing records, recorded data from automated instruments, copies of transcriptions that are certified after verification as being accurate and complete, microfiche, photographic negatives, microfilm or magnetic media, X-rays, patient files, and records kept at pharmacies, laboratories, and medico-technical departments involved in a clinical trial.

Before study initiation, the types of source documents that are to be generated will be clearly defined in the Trial Monitoring Plan. This includes any protocol data to be entered directly into the eCRFs (i.e., no prior written or electronic record of the data) and considered source data.

Source documents that are required to verify the validity and completeness of data entered into the eCRFs must not be obliterated or destroyed and must be retained per the policy for retention of records described in Section 7.5.

To facilitate source data verification and review, the investigators and institutions must provide the Sponsor direct access to applicable source documents and reports for trial-related monitoring, Sponsor audits, and IRB/EC review. The study site must also allow inspection by applicable health authorities.

7.4 USE OF COMPUTERIZED SYSTEMS

When clinical observations are entered directly into a study site's computerized medical record system (i.e., in lieu of original hardcopy records), the electronic record can serve as the source document if the system has been validated in accordance with health authority requirements pertaining to computerized systems used in clinical research. An acceptable computerized data collection system allows preservation of the original entry of data. If original data are modified, the system should maintain a viewable audit trail that shows the original data as well as the reason for the change, name of the person making the change, and date of the change.

"Use of this document is governed by the terms of use on the first page of this document."

7.5 RETENTION OF RECORDS

Records and documents pertaining to the conduct of this study and the distribution of IMP, including eCRFs, Informed Consent Forms, laboratory test results, and medication inventory records, must be retained by the Principal Investigator for 15 years after completion or discontinuation of the study or for the length of time required by relevant national or local health authorities, whichever is longer. After that period of time, the documents may be destroyed, subject to local regulations.

No records may be disposed of without the written approval of the Sponsor. Written notification should be provided to the Sponsor prior to transferring any records to another party or moving them to another location.

Roche will retain study data for 25 years after the final Clinical Study Report has been completed or for the length of time required by relevant national or local health authorities, whichever is longer.

8. ETHICAL CONSIDERATIONS

8.1 COMPLIANCE WITH LAWS AND REGULATIONS

This study will be conducted in full conformance with the ICH E6 guideline for Good Clinical Practice and the principles of the Declaration of Helsinki, or the applicable laws and regulations of the country in which the research is conducted, whichever affords the greater protection to the individual. The study will comply with the requirements of the ICH E2A guideline (Clinical Safety Data Management: Definitions and Standards for Expedited Reporting). Studies conducted in the United States or under a U.S. IND application will comply with U.S. FDA regulations and applicable local, state, and federal laws. Studies conducted in the European Union or European Economic Area will comply with the E.U. Clinical Trial Directive (2001/20/EC) and applicable local, regional, and national laws.

8.2 INFORMED CONSENT

The Sponsor’s sample Informed Consent Form (and ancillary sample Informed Consent Forms such as a Child’s Informed Assent Form or Mobile Nursing Informed Consent Form, if applicable) will be provided to each site. If applicable, it will be provided in a certified translation of the local language. The Sponsor or its designee must review and approve any proposed deviations from the Sponsor's sample Informed Consent Forms or any alternate consent forms proposed by the site (collectively, the "Consent Forms") before IRB/EC submission. The final IRB/EC–approved Consent Forms must be provided to the Sponsor for health authority submission purposes according to local requirements.

If applicable, the Informed Consent Form will contain separate sections for any optional procedures. The investigator or authorized designee will explain to each patient the objectives, methods, and potential risks associated with each optional procedure.

"Use of this document is governed by the terms of use on the first page of this document."

Patients will be told that they are free to refuse to participate and may withdraw their consent at any time for any reason. A separate, specific signature will be required to document a patient's agreement to participate in optional procedures. Patients who decline to participate will not provide a separate signature.

The Consent Forms must be signed and dated by the patient or the patient's legally authorized representative before his or her participation in the study. The case history or clinical records for each patient shall document the informed consent process and that written informed consent was obtained prior to participation in the study.

The Consent Forms should be revised whenever there are changes to study procedures or when new information becomes available that may affect the willingness of the patient to participate. The final revised IRB/EC-approved Consent Forms must be provided to the Sponsor for health authority submission purposes.

Patients must be re-consented to the most current version of the Informed Consent Forms (or to a significant new information/findings addendum in accordance with applicable laws and IRB/EC policy) during their participation in the study. For any updated or revised Consent Forms, the case history or clinical records for each patient shall document the informed consent process and that written informed consent was obtained using the updated/revised Consent Forms for continued participation in the study.

A copy of each signed Consent Form must be provided to the patient or the patient's legally authorized representative. All signed and dated Consent Forms must remain in each patient's study file or in the site file and must be available for verification by study monitors at any time.

For sites in the United States, each Consent Form may also include patient authorization to allow use and disclosure of personal health information in compliance with the U.S. Health Insurance Portability and Accountability Act (HIPAA) of 1996. If the site utilizes a separate Authorization Form for patient authorization for use and disclosure of personal health information under the HIPAA regulations, the review, approval, and other processes outlined above apply except that IRB review and approval may not be required per study site policies.

8.3 INSTITUTIONAL REVIEW BOARD OR ETHICS COMMITTEE

This protocol, the Informed Consent Forms, any information to be given to the patient, and relevant supporting information must be submitted to the IRB/EC by the Principal Investigator and reviewed and approved by the IRB/EC before the study is initiated. In addition, any patient recruitment materials must be approved by the IRB/EC.

The Principal Investigator is responsible for providing written summaries of the status of the study to the IRB/EC annually or more frequently in accordance with the requirements,

"Use of this document is governed by the terms of use on the first page of this document."

policies, and procedures established by the IRB/EC. Investigators are also responsible for promptly informing the IRB/EC of any protocol amendments (see Section 9.6).

In addition to the requirements for reporting all adverse events to the Sponsor, investigators must comply with requirements for reporting serious adverse events to the local health authority and IRB/EC. Investigators may receive written IND safety reports or other safety-related communications from the Sponsor. Investigators are responsible for ensuring that such reports are reviewed and processed in accordance with health authority requirements and the policies and procedures established by their IRB/EC, and archived in the site's study file.

8.4 CONFIDENTIALITY

The Sponsor maintains confidentiality standards by coding each patient enrolled in the study through assignment of a unique patient identification number. This means that patient names are not included in data sets that are transmitted to any Sponsor location.

Patient medical information obtained by this study is confidential and may be disclosed to third parties only as permitted by the Informed Consent Form (or separate authorization for use and disclosure of personal health information) signed by the patient, unless permitted or required by law.

Medical information may be given to a patient's personal physician or other appropriate medical personnel responsible for the patient's welfare, for treatment purposes.

Given the complexity and exploratory nature of exploratory biomarker analyses, data derived from these analyses will generally not be provided to study investigators or patients unless required by law. The aggregate results of any conducted research will be available in accordance with the effective Roche policy on study data publication (see Section 9.5).

Data generated by this study must be available for inspection upon request by representatives of national and local health authorities, Sponsor monitors, representatives, and collaborators, and the IRB/EC for each study site, as appropriate.

8.5 FINANCIAL DISCLOSURE

Investigators will provide the Sponsor with sufficient, accurate financial information in accordance with local regulations to allow the Sponsor to submit complete and accurate financial certification or disclosure statements to the appropriate health authorities. Investigators are responsible for providing information on financial interests during the course of the study and for 1 year after completion of the study (i.e., LPLV).

"Use of this document is governed by the terms of use on the first page of this document."

9. <u>STUDY DOCUMENTATION, MONITORING, AND ADMINISTRATION</u>

9.1 STUDY DOCUMENTATION

The investigator must maintain adequate and accurate records to enable the conduct of the study to be fully documented, including, but not limited to, the protocol, protocol amendments, Informed Consent Forms, and documentation of IRB/EC and governmental approval. In addition, at the end of the study, the investigator will receive the patient data, including an audit trail containing a complete record of all changes to data.

9.2 PROTOCOL DEVIATIONS

The investigator should document and explain any protocol deviations. The investigator should promptly report any deviations that might have an impact on patient safety and data integrity to the Sponsor and to the IRB/EC in accordance with established IRB/EC policies and procedures. The Sponsor will review all protocol deviations and assess whether any represent a serious breach of Good Clinical Practice guidelines and require reporting to health authorities. As per the Sponsor's standard operating procedures, prospective requests to deviate from the protocol, including requests to waive protocol eligibility criteria, are not allowed.

9.3 *MANAGEMENT OF STUDY QUALITY*

The Sponsor will implement a system to manage the quality of the study, focusing on processes and data that are essential to ensuring patient safety and data integrity. The Sponsor will identify potential risks associated with critical trial processes and data and will implement plans for evaluating and controlling these risks. Risk evaluation and control will include the selection of risk-based parameters (e.g., adverse event rate, protocol deviation rate) and the establishment of quality tolerance limits for these parameters. Detection of deviations from quality tolerance limits will trigger an evaluation to determine if action is needed. Details on the establishment and monitoring of quality tolerance limits will be provided in a Quality Tolerance Limit Management Plan.

9.4 SITE INSPECTIONS

Site visits will be conducted by the Sponsor or an authorized representative for inspection of study data, patients' medical records, and eCRFs. The investigator will permit national and local health authorities; Sponsor monitors, representatives, and collaborators; and the IRBs/ECs to inspect facilities and records relevant to this study.

"Use of this document is governed by the terms of use on the first page of this document."

9.5 ADMINISTRATIVE STRUCTURE

This study will be sponsored and managed by F. Hoffmann-La Roche Ltd. The Sponsor will provide clinical operations management, data management, and medical monitoring.

Approximately 75 sites globally will participate in the study with approximately 204 patients randomized in Stage 1 and approximately 120 patients in Stage 2. Randomization will occur through the IxRS.

Central facilities will be used for study assessments throughout the study (e.g., specified laboratory tests and PK analyses). Accredited local laboratories will be used for routine monitoring; local laboratory ranges will be collected.

An iDMC will evaluate safety and efficacy data during the study (see Section 3.1.2).

9.6 PUBLICATION OF DATA AND PROTECTION OF TRADE SECRETS

Regardless of the outcome of a trial, the Sponsor is dedicated to openly providing information on the trial to healthcare professionals and to the public, at scientific congresses, in clinical trial registries, and in peer-reviewed journals. The Sponsor will comply with all requirements for publication of study results. Study data may be shared with others who are not participating in this study (see Section 8.4 for details), and redacted Clinical Study Reports and other summary reports will be made available upon request, provided the requirements of Roche's global policy on data sharing have been met. For more information, refer to the Roche Global Policy on Sharing of Clinical Trials Data at the following website:

http://www.roche.com/roche_global_policy_on_sharing_of_clinical_study_information.pdf

The results of this study may be published or presented at scientific congresses. For all clinical trials in patients involving an IMP for which a marketing authorization application has been filed or approved in any country, the Sponsor aims to submit a journal manuscript reporting primary clinical trial results within 6 months after the availability of the respective Clinical Study Report. In addition, for all clinical trials in patients involving an IMP for which a marketing authorization application has been filed or approved in any country, the Sponsor aims to publish results from analyses of additional endpoints and exploratory data that are clinically meaningful and statistically sound.

The investigator must agree to submit all manuscripts or abstracts to the Sponsor prior to submission for publication or presentation. This allows the Sponsor to protect proprietary information and to provide comments based on information from other studies that may not yet be available to the investigator.

In accordance with standard editorial and ethical practice, the Sponsor will generally support publication of multicenter trials only in their entirety and not as individual center data. In this case, a coordinating investigator will be designated by mutual agreement.

"Use of this document is governed by the terms of use on the first page of this document."

Authorship will be determined by mutual agreement and in line with International Committee of Medical Journal Editors authorship requirements. Any formal publication of the study in which contribution of Sponsor personnel exceeded that of conventional monitoring will be considered as a joint publication by the investigator and the appropriate Sponsor personnel.

Any inventions and resulting patents, improvements, and/or know-how originating from the use of data from this study will become and remain the exclusive and unburdened property of the Sponsor, except where agreed otherwise.

9.7 PROTOCOL AMENDMENTS

Any protocol amendments will be prepared by the Sponsor. Protocol amendments will be submitted to the IRB/EC and to regulatory authorities in accordance with local regulatory requirements.

Approval must be obtained from the IRB/EC and regulatory authorities (as locally required) before implementation of any changes, except for changes necessary to eliminate an immediate hazard to patients or changes that involve logistical or administrative aspects only (e.g., change in Medical Monitor or contact information).

"Use of this document is governed by the terms of use on the first page of this document."

10. REFERENCES

Adams S, Diamond J, Hamilton E, et al. Abstract P2-11-06: Safety and clinical activity of atezolizumab (anti-PDL1) in combination with nab-paclitaxel in patients with metastatic triple-negative breast cancer. Cancer Res 2016;76(Suppl):P2-11-06.

Aapro MS, Bohlius J, Cameron DA, et al. 2010 update of EORTC guidelines for the use of granulocyte-colony stimulating factor to reduce the incidence of chemotherapy-induced febrile neutropenia in adult patients with lymphoproliferative disorders and solid tumours. Eur J Cancer 2011;47:8–32.

Aaronson NK, Ahmedzai S, Bergman B, et al. The European Organization for Research and Treatment of Cancer QLQ-C30: a quality-of-life instrument for use in international clinical trials in oncology. J Natl Cancer Inst 1993;85:365–76.

Adams S, Diamond J, Hamilton E, et al. Phase Ib trial of atezolizumab in combination with nab-paclitaxel in patients with metastatic triple-negative breast cancer (mTNBC) Poster presented at: ASCO 2016 June 3–7, 2016; Chicago, IL.

Apetoh L, Ghiringhelli F, Tesniere A, et al. Toll-like receptor 4-dependent contribution of the immune system to anticancer chemotherapy and radiotherapy. Nat Med 2007;13:1050–9.

Au HJ, Ringash J, Brundage M, et al. Added value of health-related quality of life measurement in cancer clinical trials: the experience of the NCIC CTG. Expert Rev Pharmacoecon Outcomes Res 2010;10:119–28.

Barrett T, Bowden DJ, Greenberg DC, et al. Radiological staging in breast cancer: which asymptomatic patients to image and how. Brit J Cancer 2009;101:1522–28.

Berry DA, Cirrincione C, Henderson IC, et al. Estrogen-receptor status and outcomes of modern chemotherapy for patients with node-positive breast cancer. JAMA 2006;295:1658–67.

Bines J, Earl H, Buzaid AC, et al. Anthracyclines and taxanes in the neo/adjuvant treatment of breast cancer: does the sequence matter? Ann Oncol 2014;25:1079–85.

Blank C, Gajewski TF, Mackensen A. Interaction of PD-L1 on tumor cells with PD-1 on tumor-specific T cells as a mechanism of immune evasion: implications for tumor immunotherapy. Cancer Immunol Immunother 2005;54:307–14.

Blank C, Mackensen A. Contribution of the PD-L1/PD-1 pathway to T-cell exhaustion: an update on implications for chronic infections and tumor evasion. Cancer Immunol Immunother 2007;56:739–45.

Borghaei H, Paz-Ares L, Horn L, et al. Nivolumab versus docetaxel in advanced nonsquamous non–small-cell lung cancer. N Engl J Med 2015;373:1627–39.

Brannath W, Gutjahr G, Bauer P. Probabilistic foundation of confirmatory adaptive designs. J Am Stat Assoc. 2012;107:824–32.

"Use of this document is governed by the terms of use on the first page of this document."

Brookmeyer R, Crowley J. A confidence interval for the median survival time. Biometrics 1982;38:29–41.

Brooks R. EuroQol: the current state of play. Health Policy 1996;37:53–72.

Budd GT, Barlow WE, Moore HCF. et al. SWOG S0221: a phase III trial comparing chemotherapy schedules in high-risk early-stage breast cancer. J Clin Oncol 2015;33:58–64.

Butte MJ, Keir ME, Phamduy TB, et al. Programmed death-1 ligand 1 interacts specifically with the B7-1 costimulatory molecule to inhibit T cell responses. Immunity 2007;27:111–22.

Cella DF, Tulsky DS, Gray G, et al. The Functional Assessment of Cancer Therapy (FACT) scale: development and validation of the general measure. J Clin Oncol 1993;11:570–9.

Cella D. Manual of the functional assessment of chronic illness therapy (FACIT) measurement system version 4. Evanston Northwestern Healthcare and Northwestern University, Evanston IL:Center on Outcomes, Research and Education (CORE), 1997.

Chen DS, Irving BA, Hodi FS, et al. Molecular pathways: next-generation immunotherapy—inhibiting programmed death-ligand 1 and programmed death-1. Clin Cancer Res 2012;18:6580–7.

Citron ML, Berry DA, Cirrincione C, et al. Randomized trial of dose-dense versus conventionally scheduled and sequential versus concurrent combination chemotherapy as postoperative adjuvant treatment of node-positive primary breast cancer: first report of Intergroup Trial C9741/Cancer and Leukemia Group B Trial 9741. J Clin Oncol 2003;21:2444–8.

Clopper CJ, Pearson ES. The use of confidence or fiducial limits illustrated in the case of the binomial. Biometrika 1934;26:404–13.

Cocks K, King MT, Velikova G, et al. Evidence-based guidelines for determination of sample size and interpretation of the European Organization for the Research and Treatment of Cancer Quality of Life Questionnaire Core 30. J Clin Oncol 2011;29:89–96.

Cortazar P, Zhang L, Untch M, et al. Pathological complete response and long-term clinical benefit in breast cancer: the CTNeoBC pooled analysis. Lancet 2014;384:164–72.

Crawford J, Caserta C, Roila F, et al., on behalf of the ESMO Guidelines Working Group. Hematopoietic growth factors: ESMO recommendations for the applications. Ann Oncol 2009;20:162–5.

"Use of this document is governed by the terms of use on the first page of this document."

[CTFG] Clinical Trial Facilitation Group. Recommendations related to contraception and pregnancy testing in clinical trials [resource on the Internet]. 2014 [cited 23 March 2017] Available from: http://www.hma.eu/fileadmin/dateien/Human_Medicines/01-About_HMA/Working_Groups/CTFG/2014_09_HMA_CTFG_Contraception.pdf.

Deng R, Bumbaca D, Pastuskovas CV, et al. Preclinical pharmacokinetics, pharmacodynamics, tissue distribution, and tumor penetration of anti-PD-L1 monoclonal antibody, an immune checkpoint inhibitor. MAbs 2016;8:593–603.

Dent R, Trudeau M, Pritchard KI, et al. Triple-negative breast cancer: clinical features and patterns of recurrence. Clin Cancer Res 2007;13:4429–34.

Earl HM, Vallier AL, Hiller L, et al. Effects of the addition of gemcitabine, and paclitaxel-first sequencing, in neoadjuvant sequential epirubicin, cyclophosphamide, and paclitaxel for women with high-risk early breast cancer (Neo-tAnGo): an open-label, 2 × 2 factorial randomised phase 3 trial. Lancet Oncol 2014;15:201–12.

Early Breast Cancer Trialists' Collaborative Group. Effects of chemotherapy and hormonal therapy for early breast cancer on recurrence and 15-year survival: an overview of the randomised trials. Lancet 2005;365:1687–1717.

[EMA] European Medicines Agency. Appendix 2 to the guideline on the evaluation of anticancer medicinal products in man: the use of patient-reported outcome (PRO) measures in oncology studies [resource on the Internet]. 2016. Available from: http://www.ema.europa.eu/docs/en_GB/document_library/Other/2016/04/WC500205159.pdf.

Emens LA, Cruz C, Eder JP, et al. Long-term clinical outcomes and biomarker analyses of atezolizumab therapy for patients with metastatic triple-negative breast cancer: a phase 1 study. JAMA Oncol. 2018.

EuroQol Group. EuroQol: a new facility for the measurement of health-related quality of life. Health Policy 1990;16:199–208.

Fayers PM, Aaronson NK, Bjordal K, et al. The EORTC QLQ-C30 scoring manual (3rd edition). Brussels:European Organisation for Research and Treatment of Cancer, 2001.

[FDA] United States Food and Drug Administration. Structured approach to benefit-risk assessment in drug regulatory decision-making. PDUFA implementation plan: fiscal years 2013–2017. February 2013. Available at: http://www.fda.gov/downloads/ForIndustry/UserFees/PrescriptionDrugUserFee/UCM329758.pdf.

Fehrenbacher L, Spira A, Ballinger M, et al. Atezolizumab versus docetaxel for patients with previously treated non-small-cell lung cancer (POPLAR): a multicentre, open-label, phase 2 randomised controlled trial. Lancet 2016;387:1837–46.

"Use of this document is governed by the terms of use on the first page of this document."

Ferlay J, Soerjomataram I, Ervik M, et al. GLOBOCAN 2012 v1.0, Cancer Incidence and Mortality Worldwide: IARC CancerBase No. 11 [resource on the Internet]. Lyon, France: International Agency for Research on Cancer; 2013. Available from: http://globocan.iarc.fr.

Gianni L, Mansutti M, Anton A, et. al. ETNA (Evaluating Treatment with Neoadjuvant Abraxane) randomized phase III study comparing neoadjuvant nab-paclitaxel (nab-P) versus paclitaxel (P) both followed by anthracycline regimens in women with HER2-negative high-risk breast cancer: a MICHELANGO study. J Clin Oncol 2016;34 (suppl; abstr 502).

Gradishar WJ, Tjulandin S, Davidson N, et al. Phase III trial of nanoparticle albumin-bound paclitaxel compared with polyethylated castor oil–based paclitaxel in women with breast cancer. J Clin Oncol 2005;23:7794–803.

Haffty B, Yang Q, Reiss M, et al. Locoregional relapse and distant metastasis in consevatively manager triple-negative early-stage breast cancer. J Clin Oncol 2006;10:5652–7.

Hammond MEH, Hayes DF, Dowsett M, et al. American Society of Clinical Oncology/College of American Pathologists guideline recommendations for immunohistochemical testing of estrogen and progesterone receptors in breast cancer. J Clin Oncol 2010;28:2784–95.

Herbst RS, Baas P, Kim D-W, et al. Pembrollizumab versus docetaxel for previously treated PD-L1-positive, advanced non-small-cell lung cancer (KEYNOTE-010): a randomised controlled trial. Lancet 2016;387;1540–50.

Herbst RS, Soria JC, Kowanetz M et al. Predictive correlates of response to the anti-PD-L1 antibody atezolizumab in cancer patients. Nature 2014;515:563–7.

Herdman M, Gudex C, Lloyd A, et al. Development and preliminary testing of the new five-level version of EQ-5D (EQ-5D-5L). Qual Life Res 2011;20:1727–36.

Hodi FS, O'Day SJ, McDermott DF, et al. Improved survival with ipilimumab in patients with metastatic melanoma. N Engl J Med 2010;363:711–23.

Howlader N, Noone AM, Krapcho M, et al. SEER Cancer Statistics Review, 1975–2013, National Cancer Institute. Bethesda, MD, http://seer.cancer.gov/csr/1975_2013/, based on November 2015 SEER data submission, posted to the SEER web site, April 2016.

Janssen MF, Pickard AS, Golicki D, et al. Measurement properties of the EQ-5D-5L compared to the EQ-5D-3L across eight patient groups: a multi-country study. Qual Life Res 2013;22:1717–27.

Jemal A, Bray F, Center MM, et al. Global Cancer Statistics. CA Cancer J Clin 2011;61:69–90.

Jennison C, Turnbull BW. Group sequential methods with applications to clinical trials. Boca Raton, FL: Chapman-Hall/CRC, 1999.

"Use of this document is governed by the terms of use on the first page of this document."

Kantoff PW, Higano CS, Shore ND, et al. Sipuleucel-T immunotherapy for castration-resistant prostate cancer. N Engl J Med 2010;363:411–22.

Kassam F, Enright K, Dent R, et al. Survival outcomes for patients with metastatic triple-negative breast cancer: implications for clinical practice and trial design. Clin Breast Cancer 2009;9:29–33.

Keir ME, Butte MJ, Freeman GJ, et al. PD-1 and its ligands in tolerance and immunity. Annual Rev Immunol 2008;26:677–704.

Lee DW, Santomasso BD, Locke FL, et al. ASTCT consensus grading for cytokine release syndrome and neurologic toxicity associated with immune effector cells. Biol Blood Marrow Transplant 2019;25:625–38.

Lehmann BD, Bauer JA, Chen X, et al. Identification of human triple-negative breast cancer subtypes and preclinical models for selection of targeted therapies. J Clin Invest 2011;121:2750–67.

Liedtke C, Mazouni C, Hess K, et al. Response to neoadjuvant therapy and long-term survival in patients with triple-negative breast cancer. J Clin Oncol 2008;26:1275-81.

Merritt RE, Mahtabifard A, Yamada RE, et al. Cisplatin augments cytotoxic T-lymphocyte-mediated antitumor immunity in poorly immunogenic murine lung cancer. J Thorac Cardiovasc Surg 2003;126:1609–17.

Montazeri A. Health-related quality of life in breast cancer patients: a bibliographic review of the literature from 1974 to 2007. J Exp Clin Canc Res 2008;27:1–31.

[NCCN] National Comprehensive Cancer Network. NCCN Guidelines: Breast Cancer v2 [resource on the Internet]. 2016 Available from: http://www.nccn.org/professionals/physician_gls/pdf/breast.pdf.

Osoba D, Rodrigues G, Myles J, et al. Interpreting the significance of changes in health-related quality of life score. J Clin Oncol 1998;16:139–44.

PDUFA Breast Cancer Workshop conducted on 2 April 2015. The voice of the patient report: Breast cancer. USFDA's patient-focused drug development initiative. 2015:1–25.

Petersen JA, Gauthier MA, Piault E, et al. Abstract P1-10-20: importance of the patient voice in drug development: early-stage breast cancer and measurement gaps concerning the treatment experience. Cancer Res 2015:1–5.

Peto R, Davies C, Godwin J, et al. Comparisons between different polychemotherapy regimens for early breast cancer: meta-analyses of long-term outcome among 100,000 women in 123 randomised trials. Lancet 2012;379:432–44.

Pfirschke C, Engblom C, Rickelt S, et al. Immunogenic chemotherapy sensitizes tumors to checkpoint blockade therapy. Immunity 2016;44:343–54.

"Use of this document is governed by the terms of use on the first page of this document."

Pistelli M, Pagliacci A, Battelli N, et al. Prognostic factors in early-stage triple-negative breast cancer: lessons and limits from clinical practice. Anticancer Res 2013;33:2737–42.

Rosa Mendoza ES, Moreno E, Caguioa PB, et al. Predictors of early distant metastasis in women with breast cancer. J Cancer Res Clin Oncol 2013;139:645–52.

Rosenberg JE, Hoffman-Censits J, Powles T, et al. Atezolizumab in patients with locally advanced and metastatic urothelial carcinoma who have progressed following treatment with platinum-based chemotherapy: a single-arm, multicentre, phase 2 trial. Lancet 2016;387:1909–20.

Ryerson AB, Miller J, Eheman CR, et al. Reported breast symptoms in the national breast cancer and cervical cancer early detection program. Cancer Causes Control 2015;26:1–14.

Sant M, Allemani C, Capocaccia R, et al. Stage at diagnosis is a key explanation of differences in breast cancer survival across Europe. Int J Cancer 2003;106:416–22.

Schmid P, Adams S, Rugo HS, et al. Atezolizumab and nab-paclitaxel in advanced triple-negative breast cancer. N Engl J Med 2018;379:2108–21.

Senkus E, Kyriakides S, Ohno S, et al. Primary breast cancer: ESMO clinical practice guidelines for diagnosis, treatment, and follow-up. Ann Oncol 2015;26 (s5);8–30.

Sikov WM, Berry DA, Perou CM, et al. Impact of the addition of carboplatin and/or bevacizumab to neoadjuvant once-per-week paclitaxel followed by dose-dense doxorubicin and cyclophosphamide on pathologic complete response rates in Stage II to III triple-negative breast cancer: CALGB 40603 (Alliance). J Clin Oncol 2015;33:13–21.

Simes RJ. An improved Bonferroni procedure for multiple tests of significance. Biometrika. 1986;73:751–4.

Sledge GW, Neuberg D, Bernardo P, et al. Phase III trial of doxorubicin, paclitaxel, and the combination of doxorubicin and paclitaxel as front-line chemotherapy for metastatic breast cancer: an intergroup trial (E1193). J Clin Oncol 2003;21:588–92.

Smith TJ, Khatcheressian J, Lyman GH, et al. 2006 update of recommendations for the use of white blood cell growth factors: an evidence-based clinical practice guideline. J Clin Oncol 2006:3187–205.

Sparano JA, Wang M, Martino S, et al. Weekly paclitaxel in the adjuvant treatment of breast cancer. N Engl J Med 2008;358:1663–71.

Tan D, Marchio C, Jones, R, et al. Triple-negative breast cancer: molecular profiling and prognostic impact in adjuvant anthracycline-treated patients. Breast Cancer Res Treat 2008;111:27–44.

TECENTRIQ™ (atezolizumab) injection, Package Insert, Genentech, Inc.

"Use of this document is governed by the terms of use on the first page of this document."

Topalian SL, Hodi FS, Brahmer JR, et al. Safety, activity, and immune correlates of anti–PD-1 antibody in cancer. N Engl J Med 2012;366:2443–54.

Untch M, Jackisch C, Schneeweiss A, et al. Nab-paclitaxel versus solvent-based paclitaxel in neoadjuvant chemotherapy for early breast cancer (GeparSepto—GBG 69): a randomised, phase 3 trial. Lancet Oncol 2016;17(3):345–56.

Von Minckwitz G, Loibl S, Untch M. What is the current standard of care for anti-HER2 neoadjuvant therapy in breast cancer? Oncology (Williston Park). 2012;26:20-6.

Von Minckwitz G., Schneeweiss A., Loibl S, et.al. Neoadjuvant carboplatin in patients with triple-negative and HER2-positive early breast cancer (GeparSixto; GBG 66): a randomized phase 2 trial. Lancet Oncol 2014; 15:747-56.

Wassmer G, Brannath W. Group sequential and confirmatory adaptive designs in clinical trials. Heidelberg: Springer, 2016.

Webster K, Odom L, Peterman A. et al. The Functional Assessment of Chronic Illness Therapy (FACIT) measurement system: Validation of version 4 of the core questionnaire. Quality of Life Research 1999;8:604.

Wolff AC, Hammond MEH, Hicks DG, et al. Recommendations for human epidermal growth factor receptor 2 testing in breast cancer: American Society of Clinical Oncology/College of American Pathologists clinical practice guideline update. J Clin Oncol 2013;31:3997–4013.

Yang J, Riella LV, Chock S. The novel costimulatory programmed death ligand 1/B7.1 pathway is functional in inhibiting alloimmune responses in vivo. J Immunol 2011;187:1113–9.

"Use of this document is governed by the terms of use on the first page of this document."

Appendix 1
Schedule of Activities (for Both Stage 1 and Stage 2)

	Screening [a]	Treatment: Neoadjuvant Treatment (28-Day Cycles) (Cycles 1–5; Weeks 1–20)	Treatment: Pre-Surgery Visit/ Surgery [b]	Treatment: Arm A: Adjuvant Treatment Arm B: Monitoring (21-Day Cycles) (Cycles 6–16)	Completion of Study Therapy/Early Term. Visit [c]	
	Days –28 to –1	Day 1 (±3 days)		Day 1 (±3 days)	≤30 Days after Last Dose (Arm A) or Last Monitoring Visit (Arm B)	Survival Follow-Up [d]
Informed consent	x [e]					
Baseline tumor tissue sample submission for HER2 and ER/PgR determination and exploratory biomarkers (mandatory)	x [f]					
Demographic data	x					
Medical history and baseline conditions	x					
Disease status assessments [g]	x	x	x [g]	x [g]	x [g]	x [g]
Tumor Staging [h]	x					
Ultrasound [g]	x		x			
EORTC QLQ-C30, EQ-5D-5L [i]		x		x	x	x [j]
FACT-G, Single Item GP5 [i]		x [k]		x	x	x [j]
Vital signs [l]	x	On each infusion day	x	x [m]	x	
Weight	x	x		x [m]		

"Use of this document is governed by the terms of use on the first page of this document."

	Screening [a]	Treatment: Neoadjuvant Treatment (28-Day Cycles) (Cycles 1–5; Weeks 1–20)	Treatment: Pre-Surgery Visit/ Surgery [b]	Treatment: Arm A: Adjuvant Treatment Arm B: Monitoring (21-Day Cycles) (Cycles 6–16)	Completion of Study Therapy/Early Term. Visit [c]	
	Days –28 to –1	Day 1 (±3 days)		Day 1 (±3 days)	≤30 Days after Last Dose (Arm A) or Last Monitoring Visit (Arm B)	Survival Follow-Up [d]
Height	x					
Complete physical examination [n]	x					
Limited physical examination [o]		x [g]	x	x [m]	x	
ECOG Performance Status [p]	x	x	x	x	x	
ECG (12-lead) [q]	x	As clinically indicated				
ECHO or MUGA scan [r]	x	As described in footnote “r”			x	x
Spirometry (FVC, FEV_1, FEV_1:FVC, FEF_{25-75})	x	As clinically indicated				
Hematology [s]	x [t]	On each infusion day	x	x [m]	x	
Chemistry [u]	x [t]	On each infusion day	x	x [m]	x	
Pregnancy test [v]	x [t]	x [v]		x [v]	x [v]	x [v]
Coagulation (INR, aPTT)	x [t]		x		x	
TSH, free T3 (or total T3 [w]), free T4	x [t]	x [w]			x	
Viral serology [x]	x [t]					

"Use of this document is governed by the terms of use on the first page of this document."

	Screening [a]	Treatment: Neoadjuvant Treatment (28-Day Cycles) (Cycles 1–5; Weeks 1–20)	Treatment: Pre-Surgery Visit/ Surgery [b]	Treatment: Arm A: Adjuvant Treatment Arm B: Monitoring (21-Day Cycles) (Cycles 6–16)	Completion of Study Therapy/Early Term. Visit [c]	
	Days –28 to –1	Day 1 (±3 days)		Day 1 (±3 days)	≤30 Days after Last Dose (Arm A) or Last Monitoring Visit (Arm B)	Survival Follow-Up [d]
Urinalysis [y]	x [t]	As clinically indicated				
Serum PK sample for atezolizumab		See Appendix 2 for detailed schedule				
Serum ADA sample for atezolizumab		See Appendix 2 for detailed schedule				
Blood and plasma samples for biomarkers		See Appendix 2 for detailed schedule				
Blood sample for RBR (optional) [z]		x				
Tumor tissue (fresh sample preferred) at screening, on-study, and time of disease recurrence [aa]	See Appendix 3 for detailed schedule					
Radiographic assessments (e.g., CT scan, MRI, PET scan)	As clinically indicated					
Bilateral mammogram	x [bb]				x	x [cc]
Concomitant medications	x [dd]	x		x [ee]	x [ee]	x [g, ff]
Adverse events [gg]	x [gg]	x [gg]		x	x	x [gg]
Study treatment administration [hh]		x		x [ii, m]		

"Use of this document is governed by the terms of use on the first page of this document."

		Treatment				
	Screening [a]	Neoadjuvant Treatment (28-Day Cycles) (Cycles 1–5; Weeks 1–20)	Pre-Surgery Visit/ Surgery [b]	Arm A: Adjuvant Treatment Arm B: Monitoring (21-Day Cycles) (Cycles 6–16)	Completion of Study Therapy/Early Term. Visit [c]	
	Days –28 to –1	Day 1 (±3 days)		Day 1 (±3 days)	≤30 Days after Last Dose (Arm A) or Last Monitoring Visit (Arm B)	Survival Follow-Up [d]
Survival follow-up and anti-cancer treatment						x [d, jj]

AC = doxorubicin + cyclophosphamide; ADA = anti-drug antibody; CT = computed tomography; ECHO = echocardiogram; ECOG = Eastern Cooperative Oncology Group; eCRF = electronic Case Report Form; EORTC QLQ-C30 = European Organisation for Research and Treatment of Cancer Quality of Life Questionnaire Core 30; EQ-5D-5L = EuroQoL 5-Dimension, 5-Level; ER = estrogen receptor; FACT-G = Functional Assessment of Cancer Therapy-General; FCV = Forced Vital Capacity; FEV_1 = Forced Expiratory Volume 1; FEF_{25-75} = Forced Expiratory Flow 25%–75%; FFPE = formalin-fixed, paraffin-embedded; HBcAb = hepatitis B core antibody; HBsAb = hepatitis B surface antibody; HBsAg = hepatitis B surface antigen; HBV = hepatitis B virus; HCV = hepatitis C virus; HER2 = human epidermal growth factor receptor 2; MRI = magnetic resonance imaging; MUGA = multiple-gated acquisition; PD-L1 = programmed death–ligand-1; PET = positron emission tomography; PgR = progesterone receptor; PK = pharmacokinetic; PRO = patient-reported outcome; RBR = Research Biosample Repository; T3 = triiodothyronine; T4 = thyroxine; Term. = termination; TSH = thyroid-stimulating hormone.

Notes: On treatment days, all assessments should be performed prior to dosing, unless otherwise specified.

Assessments shaded in gray should be performed as scheduled, but the associated data do not need to be recorded on the eCRF (except in the case of an adverse event).

If treatment is withheld (e.g., by adverse events or delays in initiating post-surgical therapy), the schedule of assessments should be held accordingly (e.g., Day 1 of Week 21 = first administration of atezolizumab post-surgery or Day 1 of Cycle 6).

[a] Results of standard-of-care tests or examinations performed prior to obtaining informed consent and within 30 days prior to Day 1 may be used; such tests do not need to be repeated for screening.

[b] Pre-surgical visit and associated assessments should occur within 14 days of surgery. Surgery should be conducted no earlier than 14 days and no later than 6 weeks after last dose of neoadjuvant therapy. Platelet counts should be checked prior to surgery and should be ≥75,000 cells/μL.

"Use of this document is governed by the terms of use on the first page of this document."

[c] Patients who discontinue study treatment will return to the clinic for a treatment discontinuation visit not more than 30 days after the last dose of study treatment.

[d] The survival follow-up period begins from the date of treatment completion/early termination visit, and has a duration of up to approximately 51 months from the date of randomization of the first patient in Stage 1). This may increase to approximately 74 months if the study includes Stage 2 patients. Visit windows are ±28 days for quarterly and semiannual assessments.

e Informed consent must be documented before any study-specific screening procedure is performed and may be obtained more than 28 days before initiation of study treatment.

[f] After signing of the Informed Consent Form, retrieval and submission of tumor tissue sample can occur outside the 28-day screening period. Tumor tissue should be of good quality based on total and viable tumor content (sites will be informed if the quality of the submitted specimen is inadequate to determine tumor PD-L1 status). An FFPE block or at least 20 unstained slides should be provided. Fine-needle aspiration, brushing, cell pellets from pleural effusion, and lavage samples are not acceptable. For core-needle biopsy specimens, at least three cores should be submitted for evaluation. Retrieval of tumor sample can occur outside the 28-day screening period.

[g] Assessment of primary tumor and regional lymph nodes should be done by physical examination at baseline and prior to administration of each cycle of study treatment during neoadjuvant therapy. Additionally, standard breast imaging modality should include an ultrasound of breast and axilla disease. (Physical examination and ultrasound is mandatory within 28 days prior to randomization and within 14 days pre-surgery.) At baseline, if there is evidence of suspicious axillary lymph nodes, then fine-needle aspiration is required. Ultrasound-detected axillary lymph nodes suspicious of malignancy include those with cortical thickness >2 mm. Disease status based on all available clinical assessments should be documented every 3 months during adjuvant study treatment and follow-up up to 3 years after surgery and every 6 months thereafter. In addition, liver function tests, bone scans, chest X-ray/diagnostic CT scan, liver imaging, and/or other radiographic modalities may be considered when clinically indicated to exclude metastatic disease; these assessments should be performed within a timeline as per current local standard of practice. Whenever possible, disease recurrence should be confirmed pathologically. If disease recurrence is diagnosed at any time during the study, patients will discontinue scheduled study assessments and will be followed for survival, anti-cancer medications, and new relapse events.

[h] See Section 4.5.6 in protocol.

[i] All PRO assessments (EORTC QLQ-C30, followed by the FACT-G single item GP5, and then the EQ-5D-5L questionnaires) must be completed by the patient at the investigational site at the start of the clinic visit before discussion of the patient's health state, lab results or health record, before administration of study treatment, and/or prior to the performance of any other study assessments that could bias the patient's responses. Interview assessment by a member of the clinical staff will be allowed if the patient is not able to complete the measure on her or his own. Study personnel should review all questionnaires for completeness before the patient leaves the investigational site.

The EORTC QLQ-C30 and EQ-5D-5L questionnaires will be completed by patients at baseline (Cycle 1, Day 1) and on Day 1 of every cycle thereafter. The FACT-G, single item GP5 will not be completed by patients at the baseline visit (Cycle 1, Day 1).

"Use of this document is governed by the terms of use on the first page of this document."

[j] Patients who discontinue study treatment for any reason will continue to complete the EORTC QLQ-C30, FACT-G single item GP5, and EQ-5D-5L questionnaires in-clinic during the survival follow-up period at the following timepoints: every 3 months (±28 days) for Year 1, every 6 months (± 28 days) for years 2-3, and then annually (±28 days) thereafter.

[k] While on study treatment, all patients will complete the FACT-G, single item GP5 beginning on Cycle 2, Day 1 and at Day 1 of every cycle thereafter.

[l] Includes respiratory rate, pulse rate, systolic and diastolic blood pressure while the patient is in a seated position, and temperature. Record abnormalities observed at baseline on the General Medical History and Baseline Conditions eCRF. At subsequent visits, record new or worsened clinically significant abnormalities on the Adverse Event eCRF. For the first infusion, vital signs should be measured within 60 minutes prior to the infusion and, if clinically indicated, every 15 (±5) minutes during and 30 (±10) minutes after the infusion. For subsequent infusions, vital signs should be measured within 60 minutes prior to the infusion and, if clinically indicated or if symptoms occurred during the previous infusion, during and 30 (±10) minutes after the infusion.

[m] For patients in Arm A only.

[n] Includes evaluation of the head, eyes, ears, nose, throat, and the cardiovascular, dermatological, musculoskeletal, respiratory, gastrointestinal, and neurological systems. Record abnormalities observed at baseline on the General Medical History and Baseline Conditions eCRF. At subsequent visits, record new or worsened clinically significant abnormalities on the Adverse Event eCRF.

[o] Perform a limited, symptom-directed examination at specified timepoints and as clinically indicated at other timepoints. Record new or worsened clinically significant abnormalities on the Adverse Event eCRF.

[p] See Appendix 10.

[q] ECG recordings will be obtained during screening and as clinically indicated. Patients should be resting in a supine position for at least 10 minutes prior to ECG recording.

[r] Cardiac monitoring (ECHO or MUGA scan) will be performed on all patients enrolled in the study. ECHO is the preferred method. The same method used for a given patient at screening should be used throughout the study. ECHO or MUGA scan should be obtained at **baseline** and **after the second dose of** AC (which would correspond to Week 16 ± 1 week if no study treatment interruptions or discontinuations have occurred) during neoadjuvant study treatment. During the adjuvant (Arm A) or monitoring (Arm B) phase of the study, ECHO or MUGA scan should be obtained at **Cycle 6, Day 1** (which would approximately correspond to Week 21 ± 1 week if no study treatment interruptions or discontinuations have occurred), and **every 3 months afterwards** while on the Adjuvant/Monitoring Phase (which would correspond to Cycle 10, Day 1 and Cycle 14, Day 1, or approximately Week 33 ±1 week, and Week 45 ±1 week respectively, if no treatment interruptions or discontinuations have occurred). ECHO or MUGA scan should be obtained at the **completion visit** (or the **early termination visit**) if not performed within the previous 6 weeks. During the survival follow-up period, ECHO or MUGA scan should be obtained **annually until the end of study**. For additional cardiac screening tests for patients in the cardiac safety cohort, please see Appendix 4.

[s] Hematology includes WBC count, RBC count, hemoglobin, hematocrit, platelet count, and differential count (neutrophils, eosinophils, basophils, monocytes, lymphocytes, other cells).

"Use of this document is governed by the terms of use on the first page of this document."

[t] Screening laboratory test results must be obtained within 14 days prior to initiation of study treatment.

[u] Chemistry panel (serum or plasma) includes sodium, potassium, chloride, bicarbonate or total CO2, glucose, BUN or urea, creatinine, total protein, albumin, calcium, total bilirubin, alkaline phosphatase, ALT, AST, and LDH. Magnesium and phosphorus should be included at screening and as clinically indicated during study treatment.

[v] All women of childbearing potential will have a serum pregnancy test at screening. Urine pregnancy tests will be performed at the following specified subsequent visits for women of child-bearing potential (including premenopausal women who have had tubal ligation) and women not meeting the definition of postmenopausal: Day 1 of Cycles 1 – 5; Day 1 of Cycles 6, 8, 10, 12, 14, and 16; at treatment discontinuation (unless administered within 30 days); and at 3 months and 6 months after treatment discontinuation. For all other women, documentation must be present in medical history confirming that the patient is not of childbearing potential. If a urine pregnancy test is positive, it must be confirmed by a serum pregnancy test.

[w] TSH, free T3 (or total T3 for sites where free T3 is not performed), and free T4 will be assessed on Day 1 of Cycle 1 and every fourth cycle thereafter.

[x] At screening, patients will be tested for HIV, HBsAg, HBsAb, total HBcAb, and HCV antibody. If a patient has a negative HBsAg test and a positive total HBcAb test at screening, an HBV DNA test should be performed to rule out active HBV infection prior to initiation of study treatment. If a patient has a positive HCV antibody test at screening, an HCV RNA test should be performed to rule out active HCV infection prior to initiation of study treatment.

[y] Includes pH, specific gravity, glucose, protein, ketones, and blood; dipstick permitted.

[z] Not applicable for a site that has not been granted approval for RBR sampling. Performed only for patients at participating sites who have provided written informed consent to participate. Whole blood for DNA isolation will be collected from patients who have consented to optional RBR sampling at Week 1, Day 1. If, however, the RBR genetic blood sample is not collected during the scheduled visit, it may be collected as soon as possible (after randomization) during the conduct of the clinical study.

[aa] Tumor tissue should be of good quality based on total and viable tumor content (sites will be informed if the quality of the submitted specimen is inadequate to determine tumor PD-L1 status). For tissue sample provided at screening, an FFPE block or at least 20 unstained slides should be provided. Retrieval of tumor screening sample can occur outside the 28-day screening period. Fine-needle aspiration, brushing, cell pellets from pleural effusion, and lavage samples are not acceptable. For core-needle biopsy specimens, at least three cores should be submitted for screening and on-study evaluation. At least two cores should be submitted for disease recurrence specimens. See Section 4.5.8 and Appendix 3 for specific tissue sample requirements for each time point.

[bb] The unaffected breast should have been imaged within 60 days prior to randomization. The affected breast should be imaged within 28 days prior to randomization.

[cc] Mammograms of any remaining breast tissue should be performed at least annually.

"Use of this document is governed by the terms of use on the first page of this document."

[dd] Includes any medication (e.g., prescription drugs, over-the-counter drugs, vaccines, herbal or homeopathic remedies, nutritional supplements) used by a patient in addition to protocol-mandated study treatment from 30 days prior to initiation of study treatment (for the purposes of screening) until the treatment discontinuation visit. Record all prior anti-cancer therapies.

[ee] To be collected for both study arms. For patients in Arm B (Monitoring), only medications given for reportable adverse events as per protocol (see Section 5.3.1) as well as new anti-cancer treatments should be collected.

[ff] Medications related to the treatment of serious adverse events are to be reported during the follow-up period, as well as new anti-cancer treatments.

[gg] After informed consent has been obtained but prior to initiation of study treatment, only serious adverse events caused by a protocol-mandated intervention should be reported. After initiation of study treatment, all adverse events will be reported until 30 days after the last dose of study treatment or until initiation of new anti-cancer therapy, whichever occurs first, and serious adverse events and adverse events of special interest will continue to be reported until 90 days after the last dose of study treatment or until initiation of new anti-cancer therapy, whichever occurs first. After this period, all deaths, regardless of cause, should be reported. In addition, the Sponsor should be notified if the investigator becomes aware of any serious adverse event that is believed to be related to prior exposure to study treatment (see Section 5.6). The investigator should follow each adverse event until the event has resolved to baseline grade or better, the event is assessed as stable by the investigator, the patient is lost to follow-up, or the patient withdraws consent. Every effort should be made to follow all serious adverse events considered to be related to study treatment or trial-related procedures until a final outcome can be reported.

[hh] The initial dose of atezolizumab will be delivered over 60 ($\pm$15) minutes. Subsequent infusions will be delivered over 30 ($\pm$10) minutes if the previous infusion was tolerated without infusion-associated adverse events, or 60 ($\pm$15) minutes if the patient experienced an infusion-associated adverse event with the previous infusion.

[ii] Study drug administration during the maintenance phase for the atezolizumab-containing arm only.

[jj] After treatment discontinuation, information on survival follow-up and new anti-cancer therapy (including targeted therapy and immunotherapy) will be collected via telephone calls, patient medical records, and/or clinic visits (unless the patient withdraws consent or the Sponsor terminates the study). If a patient requests to be withdrawn from follow-up, this request must be documented in the source documents and signed by the investigator. If the patient withdraws from study, the study staff may use a public information source (e.g., county records) to obtain information about survival status only, where allowable by local regulation.

"Use of this document is governed by the terms of use on the first page of this document."

Appendix 2
Schedule of Pharmacokinetic, Immunogenicity, and Fluid Biomarker Samples

Visit	Time	Sample
Day 1 of Week 1<br>(i.e., Cycle 1, Day 1)	Prior to the first dose	• Atezolizumab PK (serum)<br>• Atezolizumab ADA (serum)<br>• Biomarker (blood and plasma)<br>• Optional RBR (whole blood)<br>• Autoantibodies [b]
	30 min (± 10 min) after completion of atezolizumab infusion	• Atezolizumab PK (serum)
Day 1 of Weeks 7 and 15<br>(i.e., Cycle 2, Day 15; and Cycle 4, Day 15)	Predose	• Biomarkers (plasma)
Day 1 of Weeks 5 (i.e., Cycle 2, Day 1), 9 (Cycle 3, Day 1), 13 (Cycle 4, Day 1), 21 [a] (Cycle 6, Day 1), 27 [a] (Cycle 8, Day 1), 39 [a] (Cycle12, Day1), and 51 [a] (Cycle 16, Day1)	Predose	• Atezolizumab PK (serum)<br>• Atezolizumab ADA (serum)
Surgical intervention	Pre-surgery	• Biomarkers (plasma and blood)
At the time of first clinical visit post-surgical intervention	NA	• Biomarkers (plasma)
Treatment discontinuation visit (≤ 30 days after last dose) [a]	NA	• Atezolizumab PK (serum)<br>• Atezolizumab ADA (serum)
120 ± 30 days after last dose of atezolizumab [a]	NA	• Atezolizumab PK (serum)<br>• Atezolizumab ADA (serum)
At disease recurrence	NA	• Biomarkers (plasma and blood)

ADA = anti-drug antibody; NA = not applicable; PK = pharmacokinetic; RBR = Research Biosample Repository; WGS = whole genome sequencing.

Notes: Except for Day 1 of Cycle 1, all other study visits and assessments during the treatment period should be performed within ± 3 days of the scheduled date. Study assessments may be delayed or moved ahead of the window to accommodate holidays, vacations, and unforeseen delays.
If treatment is withheld (e.g., due to adverse events), the pharmacokinetic, immunogenicity, and biomarker samples during the neoadjuvant phase should be taken during the same cycle as shown in the table above (e.g., if the dose on Day 1 of Week 5 is delayed, the atezolizumab PK [serum] sample and atezolizumab ADA [serum] sample scheduled for predose should still be taken on Day 1 of Week 5). The only exception is on Day 1 of Week 1 (Cycle 1, Day 1) when the sample should only be taken on the day of the patient's first dose of the study.
Biomarker (blood) sample may include whole blood WGS. Please see lab manual for detailed collection instructions.

[a] Following unblinding at Week 20, PK, and ADA sample collections will be limited to the atezolizumab arm such that no PK and ADA samples will be collected for the placebo arm at Weeks 21, 27, 39, 51, treatment discontinuation after unblinding, and 120 (± 30) days after last dose of atezolizumab.

"Use of this document is governed by the terms of use on the first page of this document."

[b] Autoantibodies will be collected prior to the first dose and at the time of any immune-mediated adverse event. Autoantibodies to be collected include the following: anti-nuclear antibody, anti–double-stranded DNA, circulating anti-neutrophil cytoplasmic antibody, and perinuclear anti-neutrophil cytoplasmic antibody.

"Use of this document is governed by the terms of use on the first page of this document."

Appendix 3
Tumor Tissue Requirements

Timepoint	Fresh vs. Archival	Number of Slides/Cores
Screening [a]	Fresh or archival	At least 20 slides fresh from a tissue block with at least 3 cores
Week 3, Day 1 (i.e., Cycle 1, Day 15) (optional)	Fresh	Tissue block with at least 3 cores
Surgical resection	Fresh	Tissue block with at least 3 cores or equivalent
Post-surgical disease relapse	Fresh	Tissue block with at least 3 cores

TNBC=triple-negative breast cancer.

[a] Patients with multifocal tumors (more than one tumor confined to the same quadrant as the primary tumor) are eligible provided all discrete lesions are sampled and centrally confirmed as TNBC.

"Use of this document is governed by the terms of use on the first page of this document."

Appendix 4 Cardiac Safety Cohort Cardiac Assessments

Figure 1: Cardiac Monitoring Cardiac Safety Cohort

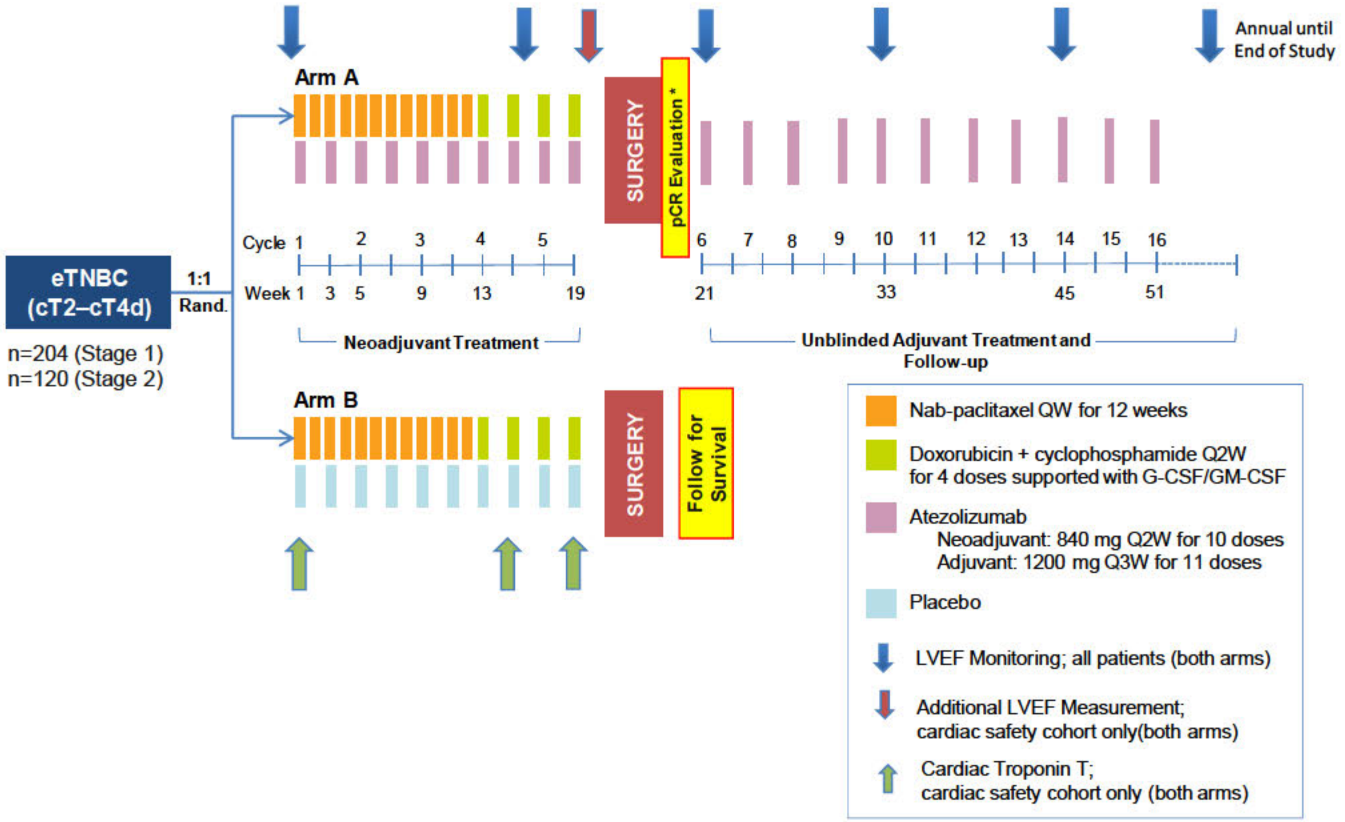

G-CSF = granulocyte colony-stimulating factor; GM-CSF = granulocyte-macrophage colony-stimulating factor; pCR = pathologic complete response; QW = weekly; Q2W = every 2 weeks; Q3W = every 3 weeks; TNBC = triple-negative breast cancer.

"Use of this document is governed by the terms of use on the first page of this document."

Note:

The first 26 patients enrolled (approximately 13 patients in the control arm and approximately 13 patients in the atezolizumab arm) will be part of a cardiac safety cohort and will undergo additional cardiac safety monitoring during the doxorubicin + cyclophosphamide portion of neoadjuvant treatment.

Cardiac monitoring (ECHO or MUGA scan) will be performed on all patients enrolled in the study. ECHO is the preferred method. The same method used for a given patient at screening should be used throughout the study.

For all patients, ECHO or MUGA scan will be obtained at baseline and after the second dose of AC (which would correspond to Week 16 ± 1 week if no study treatment interruptions or discontinuations had occurred) during neoadjuvant study treatment. During the adjuvant (arm A) or monitoring (arm B) phase of the study, ECHO or MUGA scan should be obtained at Cycle 6 Day 1 (which would approximately correspond to Week 21 ± 1 week if no study treatment interruptions or discontinuations had occurred), and every 3 months afterwards while on the adjuvant / monitoring phase (which would correspond to Cycle 10 Day 1 and Cycle 14 Day 1, or approximately Week 33 ± 1 week, and Week 45 ± 1 week respectively, if no treatment interruptions or discontinuations had occurred). ECHO or MUGA scan should be obtained at the **completion visit** (or the **early termination visit**) if not performed within the previous 6 weeks. During the survival follow-up period, ECHO or MUGA scan should be obtained annually until the end of study (for further details see the SOA Appendix 1). Patients in the cardiac safety cohort will receive an additional scan after the final dose of AC (Week 20 ± 1 week if no study treatment interruptions or discontinuations had occurred; prior to surgery).

Patients in the cardiac safety cohort will also have cardiac troponin T (cTnT) levels drawn at baseline and within 30 minutes prior to the administration of atezolizumab at week 16 and 19. If levels are found to be elevated, patients should undergo evaluation by a cardiologist and treatment decisions should be discussed with the Medical Monitor

"Use of this document is governed by the terms of use on the first page of this document."

Appendix 5
European Organisation for Research and Treatment of Cancer Quality of Life Questionnaire Core 30

EORTC QLQ-C30 (version 3)

We are interested in some things about you and your health. Please answer all of the questions yourself by circling the number that best applies to you. There are no "right" or "wrong" answers. The information that you provide will remain strictly confidential.

Please fill in your initials: |_|_|_|_|
Your birthdate (Day, Month, Year): |_|_|_|_|_|_|_|_|
Today's date (Day, Month, Year): 31 |_|_|_|_|_|_|_|_|

	Not at All	A Little	Quite a Bit	Very Much
1. Do you have any trouble doing strenuous activities, like carrying a heavy shopping bag or a suitcase?	1	2	3	4
2. Do you have any trouble taking a long walk?	1	2	3	4
3. Do you have any trouble taking a short walk outside of the house?	1	2	3	4
4. Do you need to stay in bed or a chair during the day?	1	2	3	4
5. Do you need help with eating, dressing, washing yourself or using the toilet?	1	2	3	4

During the past week:	Not at All	A Little	Quite a Bit	Very Much
6. Were you limited in doing either your work or other daily activities?	1	2	3	4
7. Were you limited in pursuing your hobbies or other leisure time activities?	1	2	3	4
8. Were you short of breath?	1	2	3	4
9. Have you had pain?	1	2	3	4
10. Did you need to rest?	1	2	3	4
11. Have you had trouble sleeping?	1	2	3	4
12. Have you felt weak?	1	2	3	4
13. Have you lacked appetite?	1	2	3	4
14. Have you felt nauseated?	1	2	3	4
15. Have you vomited?	1	2	3	4
16. Have you been constipated?	1	2	3	4

Specimen

"Use of this document is governed by the terms of use on the first page of this document."

During the past week:	Not at All	A Little	Quite a Bit	Very Much
17. Have you had diarrhea?	1	2	3	4
18. Were you tired?	1	2	3	4
19. Did pain interfere with your daily activities?	1	2	3	4
20. Have you had difficulty in concentrating on things, like reading a newspaper or watching television?	1	2	3	4
21. Did you feel tense?	1	2	3	4
22. Did you worry?	1	2	3	4
23. Did you feel irritable?	1	2	3	4
24. Did you feel depressed?	1	2	3	4
25. Have you had difficulty remembering things?	1	2	3	4
26. Has your physical condition or medical treatment interfered with your family life?	1	2	3	4
27. Has your physical condition or medical treatment interfered with your social activities?	1	2	3	4
28. Has your physical condition or medical treatment caused you financial difficulties?	1	2	3	4

For the following questions please circle the number between 1 and 7 that best applies to you

29. How would you rate your overall health during the past week?

1	2	3	4	5	6	7
Very poor						Excellent

30. How would you rate your overall quality of life during the past week?

1	2	3	4	5	6	7
Very poor						Excellent

© Copyright 1995 EORTC Quality of Life Group. All rights reserved. Version 3.0

Specimen

"Use of this document is governed by the terms of use on the first page of this document."

Appendix 6
FACT-G Single Item GP5

GP5 (Version 4)

Below is a list of statements that other people with your illness have said are important. **Please circle or mark one number per line to indicate your response as it applies to the past 7 days.**

		Not at all	A little bit	Some-what	Quite a bit	Very much
GP5	I am bothered by side effects of treatment........................	0	1	2	3	4

DRAFT

"Use of this document is governed by the terms of use on the first page of this document."

Appendix 7
EuroQol 5-Dimension, 5-Level Questionnaire: EQ-5D-5L

EQ-5D-5L

SAMPLE

Health Questionnaire

English version for the UK

UK (English) v.2 © 2009 EuroQol Group. EQ-5D™ is a trade mark of the EuroQol Group

"Use of this document is governed by the terms of use on the first page of this document."

Under each heading, please tick the ONE box that best describes your health TODAY

MOBILITY
I have no problems in walking about ❑
I have slight problems in walking about ❑
I have moderate problems in walking about ❑
I have severe problems in walking about ❑
I am unable to walk about ❑

SELF-CARE
I have no problems washing or dressing myself ❑
I have slight problems washing or dressing myself ❑
I have moderate problems washing or dressing myself ❑
I have severe problems washing or dressing myself ❑
I am unable to wash or dress myself ❑

USUAL ACTIVITIES *(e.g. work, study, housework, family or leisure activities)*
I have no problems doing my usual activities ❑
I have slight problems doing my usual activities ❑
I have moderate problems doing my usual activities ❑
I have severe problems doing my usual activities ❑
I am unable to do my usual activities ❑

PAIN / DISCOMFORT
I have no pain or discomfort ❑
I have slight pain or discomfort ❑
I have moderate pain or discomfort ❑
I have severe pain or discomfort ❑
I have extreme pain or discomfort ❑

ANXIETY / DEPRESSION
I am not anxious or depressed ❑
I am slightly anxious or depressed ❑
I am moderately anxious or depressed ❑
I am severely anxious or depressed ❑
I am extremely anxious or depressed ❑

UK (English) v.2 © 2009 EuroQol Group. EQ-5D™ is a trade mark of the EuroQol Group

SAMPLE

"Use of this document is governed by the terms of use on the first page of this document."

- We would like to know how good or bad your health is TODAY.
- This scale is numbered from 0 to 100.
- 100 means the <u>best</u> health you can imagine.
  0 means the <u>worst</u> health you can imagine.
- Mark an X on the scale to indicate how your health is TODAY.
- Now, please write the number you marked on the scale in the box below.

YOUR HEALTH TODAY = ☐

The best health you can imagine

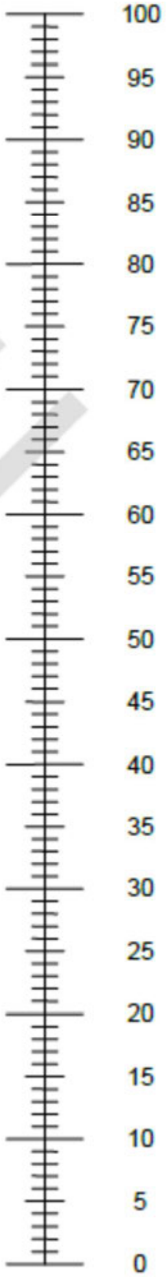

The worst health you can imagine

SAMPLE

UK (English) v.2 © 2009 EuroQol Group. EQ-5D™ is a trade mark of the EuroQol Group

"Use of this document is governed by the terms of use on the first page of this document."

Appendix 8
Preexisting Autoimmune Diseases

Subjects should be carefully questioned regarding their history of acquired or congenital immune deficiencies or autoimmune disease. Subjects with any history of immune deficiencies or autoimmune disease listed in the table below are excluded from participating in the study. Possible exceptions to this exclusion could be subjects with a medical history of such entities as atopic disease or childhood arthralgias where the clinical suspicion of autoimmune disease is low. Patients with a history of autoimmune-related hypothyroidism on a stable dose of thyroid replacement hormone may be eligible for this study. In addition, transient autoimmune manifestations of an acute infectious disease that resolved upon treatment of the infectious agent are not excluded (e.g., acute Lyme arthritis). Contact the Medical Monitor regarding any uncertainty over autoimmune exclusions.

Autoimmune Diseases and Immune Deficiencies

• Acute disseminated encephalomyelitis<br>• Addison disease<br>• Ankylosing spondylitis<br>• Antiphospholipid antibody syndrome<br>• Aplastic anemia<br>• Autoimmune hemolytic anemia<br>• Autoimmune hepatitis<br>• Autoimmune hypoparathyroidism<br>• Autoimmune hypophysitis<br>• Autoimmune myocarditis<br>• Autoimmune oophoritis<br>• Autoimmune orchitis<br>• Autoimmune thrombocytopenic purpura<br>• Behçet disease<br>• Bullous pemphigoid<br>• Chronic fatigue syndrome<br>• Chronic inflammatory demyelinating polyneuropathy<br>• Churg-Strauss syndrome<br>• Crohn disease	• Dermatomyositis<br>• Diabetes mellitus type 1<br>• Dysautonomia<br>• Epidermolysis bullosa acquisita<br>• Gestational pemphigoid<br>• Giant cell arteritis<br>• Goodpasture syndrome<br>• Graves disease<br>• Guillain-Barré syndrome<br>• Hashimoto disease<br>• IgA nephropathy<br>• Inflammatory bowel disease<br>• Interstitial cystitis<br>• Kawasaki disease<br>• Lambert-Eaton myasthenia syndrome<br>• Lupus erythematosus<br>• Lyme disease - chronic<br>• Meniere syndrome<br>• Mooren ulcer<br>• Morphea<br>• Multiple sclerosis<br>• Myasthenia gravis	• Neuromyotonia<br>• Opsoclonus myoclonus syndrome<br>• Optic neuritis<br>• Ord thyroiditis<br>• Pemphigus<br>• Pernicious anemia<br>• Polyarteritis nodosa<br>• Polyarthritis<br>• Polyglandular autoimmune syndrome<br>• Primary biliary cirrhosis<br>• Psoriasis<br>• Reiter syndrome<br>• Rheumatoid arthritis<br>• Sarcoidosis<br>• Scleroderma<br>• Sjögren syndrome<br>• Stiff-Person syndrome<br>• Takayasu arteritis<br>• Ulcerative colitis<br>• Vitiligo<br>• Vogt-Koyanagi-Harada disease<br>• Wegener granulomatosis

"Use of this document is governed by the terms of use on the first page of this document."

Appendix 9
Anaphylaxis Precautions

EQUIPMENT NEEDED

- Oxygen
- Epinephrine for subcutaneous, intravenous, and/or endotracheal use in accordance with standard practice
- Antihistamines
- Corticosteroids
- Intravenous infusion solutions, tubing, catheters, and tape

PROCEDURES

In the event of a suspected anaphylactic reaction during study treatment infusion, the following procedures should be performed:

1. Stop the study treatment infusion.
2. Maintain an adequate airway.
3. Administer antihistamines, epinephrine, or other medications as required by patient status and directed by the physician in charge.
4. Continue to observe the patient and document observations.

"Use of this document is governed by the terms of use on the first page of this document."

Appendix 10
Eastern Cooperative Oncology Group Scale of Performance Status

Grade	Performance Status
0	Fully active, able to carry on all pre-disease performance without restriction
1	Restricted in physically strenuous activity but ambulatory and able to carry out work of a light or sedentary nature, eg, light house work, office work
2	Ambulatory and capable of all selfcare but unable to carry out any work activities. Up and about more than 50% of waking hours
3	Capable of only limited selfcare, confined to bed or chair more than 50% of waking hours
4	Completely disabled. Cannot carry on any selfcare. Totally confined to bed or chair

Oken MM, Creech RH, Tormey DC, et al: Toxicity and response criteria of the eastern cooperative oncology group. Am J Clin Oncol 1982;5:649–55.

"Use of this document is governed by the terms of use on the first page of this document."

Appendix 11
Guideline on the Management of Axillary Lymph Nodes for Study WO39392 (Impassion031)

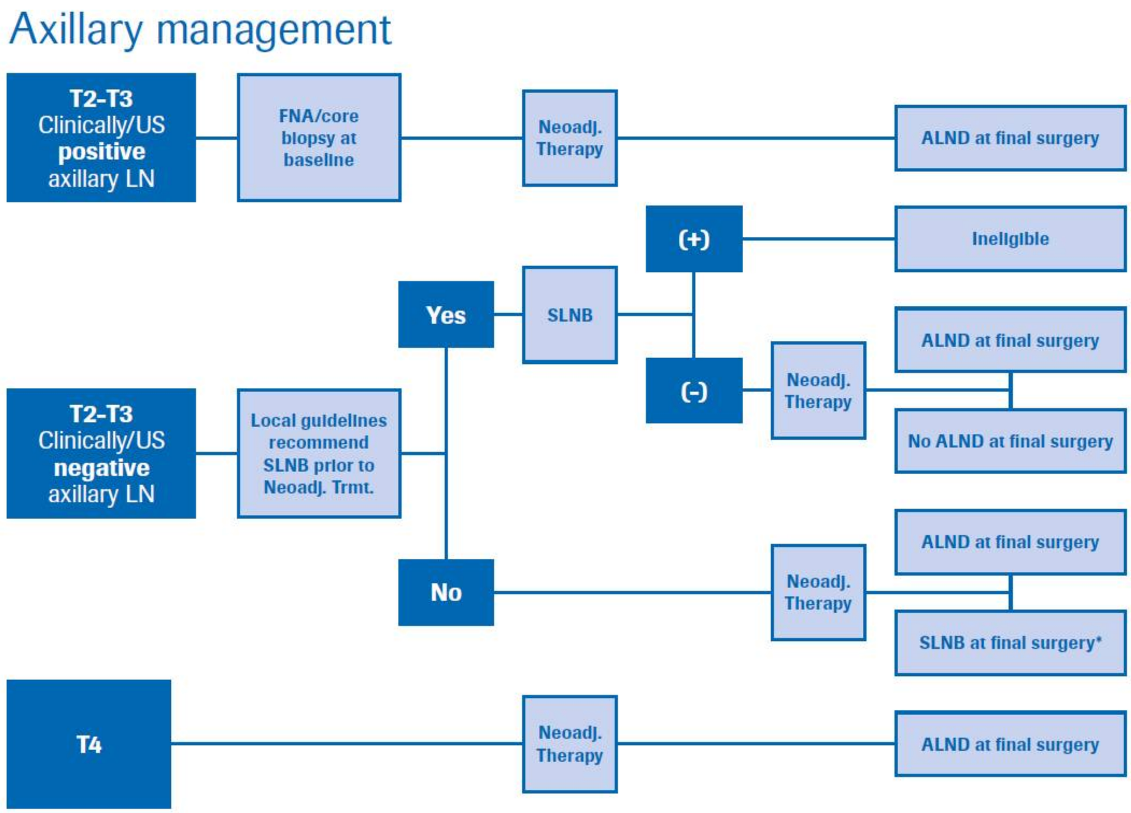

ALND = axillary lymph node dissection;FNA = fine-needle aspiration; LN = lymph node; Neoadj. = neoadjuvant; SLNB = sentinel lymph node biopsy; Trmt. = treatment; US = ultrasonography.

Notes: For patients with T2-T3 disease who have clinical or ultrasound detected enlarged axillary lymph nodes but the result of the core biopsy or FNA is negative, the investigator may follow their local practice guidelines for the management of axilla.

All patients with T4 lesions who have clinically or ultrasound detected enlarged LNs must undergo core biopsy/FNA for histological confirmation of LN involvement.

* If SLNB is conducted, it is strongly recommended that more than one lymph node (two to three minimum) be removed and that all patients with positive macrometastases in sentinel nodes should undergo ALND regardless of the number of positive nodes.

"Use of this document is governed by the terms of use on the first page of this document."

Appendix 12
Criteria for Baseline Nodal Staging and Implications on Post-Neoadjuvant Axillary Lymph Node Management for Study WO39392 (IMpassion031)

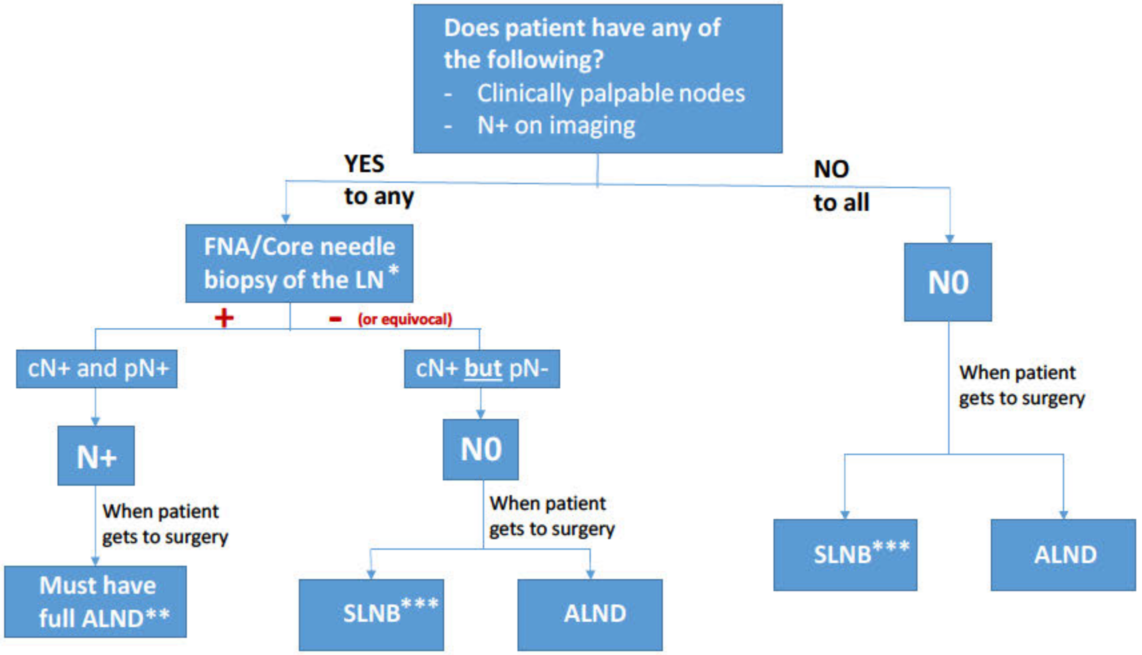

ALND = axillary lymph node dissection; FNA = fine-needle aspiration; LN = lymph node; SLNB = sentinel lymph node biopsy.

* Baseline FNA or core-needle biopsy is not mandatory for clinically/radiologically enlarged/suspicious internal mammary, subpectoral infraclavicular, or supraclavicular lymph nodes. Investigators are advised to follow local practice guidelines regarding assessment of internal mammary, subpectoral, infraclavicular, or supraclavicular lymph nodes.

** Management of internal mammary, subpectoral, infraclavicular or supraclavicular lymph nodes at the time of definitive surgery will be based on local practice guidelines. Patients with baseline axillary lymph nodes involvement who may also have clinically/radiologically enlarged internal mammary, subpectoral, infraclavicular, or supraclavicular lymph nodes must still undergo ALND as described in Section 3.1.1.

*** If SLNB is conducted, it is strongly recommended that more than one lymph node (two to three minimum) be removed and that all patients with positive macrometastases in sentinel nodes should undergo ALND regardless of the number of positive nodes.

"Use of this document is governed by the terms of use on the first page of this document."

Appendix 13
Risks Associated with Atezolizumab and Guidelines for Management of Adverse Events Associated with Atezolizumab

Toxicities associated or possibly associated with atezolizumab treatment should be managed according to standard medical practice. Additional tests, such as autoimmune serology or biopsies, should be used to evaluate for a possible immunogenic etiology.

Although most *immune-mediated* adverse events observed with immunomodulatory agents have been mild and self-limiting, such events should be recognized early and treated promptly to avoid potential major complications. Discontinuation of atezolizumab may not have an immediate therapeutic effect, and in severe cases, *immune-mediated* toxicities may require acute management with topical corticosteroids, systemic corticosteroids, or other immunosuppressive agents.

The investigator should consider the benefit–risk balance a given patient may be experiencing prior to further administration of atezolizumab. In patients who have met the criteria for permanent discontinuation, resumption of atezolizumab may be considered if the patient is deriving benefit and has fully recovered from the *immune-mediated* event. Patients can be re-challenged with atezolizumab only after approval has been documented by both the investigator (or an appropriate delegate) and the Medical Monitor.

PULMONARY EVENTS

Dyspnea, cough, fatigue, hypoxia, pneumonitis, and pulmonary infiltrates have been associated with the administration of atezolizumab. Patients will be assessed for pulmonary signs and symptoms throughout the study and will also have computed tomography (CT) scans of the chest performed at every tumor assessment.

All pulmonary events should be thoroughly evaluated for other commonly reported etiologies such as pneumonia or other infection, lymphangitic carcinomatosis, pulmonary embolism, heart failure, chronic obstructive pulmonary disease, or pulmonary hypertension. Management guidelines for pulmonary events are provided in Table 1.

"Use of this document is governed by the terms of use on the first page of this document."

Table 1 Management Guidelines for Pulmonary Events, Including Pneumonitis

Event	Management
Pulmonary event, Grade 1	• Continue atezolizumab and monitor closely.<br>• Re-evaluate on serial imaging.<br>• Consider patient referral to pulmonary specialist.
Pulmonary event, Grade 2	• Withhold atezolizumab for up to 12 weeks after event onset. [a]<br>• Refer patient to pulmonary and infectious disease specialists and consider bronchoscopy or BAL.<br>• Initiate treatment with corticosteroids equivalent to 1–2 mg/kg/day oral prednisone.<br>• If event resolves to Grade 1 or better, resume atezolizumab. [b]<br>• If event does not resolve to Grade 1 or better while withholding atezolizumab, permanently discontinue atezolizumab and contact Medical Monitor. [c]<br>• For recurrent events, treat as a Grade 3 or 4 event.
Pulmonary event, Grade 3 or 4	• Permanently discontinue atezolizumab and contact Medical Monitor. [c]<br>• Bronchoscopy or BAL is recommended.<br>• Initiate treatment with corticosteroids equivalent to 1–2 mg/kg/day oral prednisone.<br>• If event does not improve within 48 hours after initiating corticosteroids, consider adding an immunosuppressive agent.<br>• If event resolves to Grade 1 or better, taper corticosteroids over ≥ 1 month.

BAL = bronchoscopic alveolar lavage.

[a] Atezolizumab may be withheld for a longer period of time (i.e., > 12 weeks after event onset) to allow for corticosteroids (if initiated) to be reduced to ≤ 10 mg/day oral prednisone or equivalent. The acceptable length of the extended period of time must be agreed upon by the investigator and the Medical Monitor.

[b] If corticosteroids have been initiated, they must be tapered over ≥ 1 month to ≤ 10 mg/day oral prednisone or equivalent before atezolizumab can be resumed.

[c] Resumption of atezolizumab may be considered in patients who are deriving benefit and have fully recovered from the *immune-mediated* event. Patients can be re-challenged with atezolizumab only after approval has been documented by both the investigator (or an appropriate delegate) and the Medical Monitor.

"Use of this document is governed by the terms of use on the first page of this document."

HEPATIC EVENTS

Immune-mediated hepatitis has been associated with the administration of atezolizumab. Eligible patients must have adequate liver function, as manifested by measurements of total bilirubin and hepatic transaminases, and liver function will be monitored throughout study treatment. Management guidelines for hepatic events are provided in Table 2.

Patients with right upper-quadrant abdominal pain and/or unexplained nausea or vomiting should have liver function tests (LFTs) performed immediately and reviewed before administration of the next dose of study drug.

For patients with elevated LFTs, concurrent medication, viral hepatitis, and toxic or neoplastic etiologies should be considered and addressed, as appropriate.

Table 2 Management Guidelines for Hepatic Events

Event	Management
Hepatic event, Grade 1	• Continue atezolizumab.<br>• Monitor LFTs until values resolve to within normal limits.
Hepatic event, Grade 2	**All events:**<br>• Monitor LFTs more frequently until return to baseline values.<br>**Events of > 5 days' duration:**<br>• Withhold atezolizumab for up to 12 weeks after event onset [a]<br>• Initiate treatment with corticosteroids equivalent to 1–2 mg/kg/day oral prednisone.<br>• If event resolves to Grade 1 or better, resume atezolizumab. [b]<br>• If event does not resolve to Grade 1 or better while withholding atezolizumab, permanently discontinue atezolizumab and contact Medical Monitor. [c]

LFT = liver function test.

[a] Atezolizumab may be withheld for a longer period of time (i.e., > 12 weeks after event onset) to allow for corticosteroids (if initiated) to be reduced to ≤ 10 mg/day oral prednisone or equivalent. The acceptable length of the extended period of time must be agreed upon by the investigator and the Medical Monitor.

[b] If corticosteroids have been initiated, they must be tapered over ≥ 1 month to ≤ 10 mg/day oral prednisone or equivalent before atezolizumab can be resumed.

[c] Resumption of atezolizumab may be considered in patients who are deriving benefit and have fully recovered from the *immune-mediated* event. Patients can be re-challenged with atezolizumab only after approval has been documented by both the investigator (or an appropriate delegate) and the Medical Monitor.

"Use of this document is governed by the terms of use on the first page of this document."

Table 2 Management Guidelines for Hepatic Events (cont.)

Event	Management
Hepatic event, Grade 3 or 4	• Permanently discontinue atezolizumab and contact Medical Monitor. [c]<br>• Consider patient referral to gastrointestinal specialist for evaluation and liver biopsy to establish etiology of hepatic injury.<br>• Initiate treatment with corticosteroids equivalent to 1–2 mg/kg/day oral prednisone.<br>• If event does not improve within 48 hours after initiating corticosteroids, consider adding an immunosuppressive agent.<br>• If event resolves to Grade 1 or better, taper corticosteroids over ≥ 1 month.

LFT = liver function test.

[a] Atezolizumab may be withheld for a longer period of time (i.e., > 12 weeks after event onset) to allow for corticosteroids (if initiated) to be reduced to ≤ 10 mg/day oral prednisone or equivalent. The acceptable length of the extended period of time must be agreed upon by the investigator and the Medical Monitor.

[b] If corticosteroids have been initiated, they must be tapered over ≥ 1 month to ≤ 10 mg/day oral prednisone or equivalent before atezolizumab can be resumed.

[c] Resumption of atezolizumab may be considered in patients who are deriving benefit and have fully recovered from the *immune-mediated* event. Patients can be re-challenged with atezolizumab only after approval has been documented by both the investigator (or an appropriate delegate) and the Medical Monitor.

GASTROINTESTINAL EVENTS

Immune-mediated colitis has been associated with the administration of atezolizumab. Management guidelines for diarrhea or colitis are provided in Table 3.

All events of diarrhea or colitis should be thoroughly evaluated for other more common etiologies. For events of significant duration or magnitude or associated with signs of systemic inflammation or acute-phase reactants (e.g., increased C-reactive protein, platelet count, or bandemia): Perform sigmoidoscopy (or colonoscopy, if appropriate) with colonic biopsy, with three to five specimens for standard paraffin block to check for inflammation and lymphocytic infiltrates to confirm colitis diagnosis.

"Use of this document is governed by the terms of use on the first page of this document."

Table 3 Management Guidelines for Gastrointestinal Events (Diarrhea or Colitis)

Event	Management
Diarrhea or colitis, Grade 1	• Continue atezolizumab.<br>• Initiate symptomatic treatment.<br>• Endoscopy is recommended if symptoms persist for > 7 days.<br>• Monitor closely.
Diarrhea or colitis, Grade 2	• Withhold atezolizumab for up to 12 weeks after event onset. [a]<br>• Initiate symptomatic treatment.<br>• Patient referral to GI specialist is recommended.<br>• For recurrent events or events that persist > 5 days, initiate treatment with corticosteroids equivalent to 1–2 mg/kg/day oral prednisone.<br>• If event resolves to Grade 1 or better, resume atezolizumab. [b]<br>• If event does not resolve to Grade 1 or better while withholding atezolizumab, permanently discontinue atezolizumab and contact Medical Monitor. [c]
Diarrhea or colitis, Grade 3	• Withhold atezolizumab for up to 12 weeks after event onset. [a]<br>• Refer patient to GI specialist for evaluation and confirmatory biopsy.<br>• Initiate treatment with corticosteroids equivalent to 1–2 mg/kg/day IV methylprednisolone and convert to 1–2 mg/kg/day oral prednisone or equivalent upon improvement.<br>• If event resolves to Grade 1 or better, resume atezolizumab. [b]<br>• If event does not resolve to Grade 1 or better while withholding atezolizumab, permanently discontinue atezolizumab and contact Medical Monitor. [c]

GI = gastrointestinal.

[a] Atezolizumab may be withheld for a longer period of time (i.e., > 12 weeks after event onset) to allow for corticosteroids (if initiated) to be reduced to ≤ 10 mg/day oral prednisone or equivalent. The acceptable length of the extended period of time must be agreed upon by the investigator and the Medical Monitor.

[b] If corticosteroids have been initiated, they must be tapered over ≥ 1 month to ≤ 10 mg/day oral prednisone or equivalent before atezolizumab can be resumed.

[c] Resumption of atezolizumab may be considered in patients who are deriving benefit and have fully recovered from the *immune-mediated* event. Patients can be re-challenged with atezolizumab only after approval has been documented by both the investigator (or an appropriate delegate) and the Medical Monitor.

"Use of this document is governed by the terms of use on the first page of this document."

Table 3 Management Guidelines for Gastrointestinal Events (Diarrhea or Colitis) (cont.)

Event	Management
Diarrhea or colitis, Grade 4	• Permanently discontinue atezolizumab and contact Medical Monitor. [c]<br>• Refer patient to GI specialist for evaluation and confirmation biopsy.<br>• Initiate treatment with corticosteroids equivalent to 1–2 mg/kg/day IV methylprednisolone and convert to 1–2 mg/kg/day oral prednisone or equivalent upon improvement.<br>• If event does not improve within 48 hours after initiating corticosteroids, consider adding an immunosuppressive agent.<br>• If event resolves to Grade 1 or better, taper corticosteroids over ≥ 1 month.

GI = gastrointestinal.

[a] Atezolizumab may be withheld for a longer period of time (i.e., > 12 weeks after event onset) to allow for corticosteroids (if initiated) to be reduced to ≤ 10 mg/day oral prednisone or equivalent. The acceptable length of the extended period of time must be agreed upon by the investigator and the Medical Monitor.

[b] If corticosteroids have been initiated, they must be tapered over ≥ 1 month to ≤ 10 mg/day oral prednisone or equivalent before atezolizumab can be resumed.

[c] Resumption of atezolizumab may be considered in patients who are deriving benefit and have fully recovered from the *immune-mediated* event. Patients can be re-challenged with atezolizumab only after approval has been documented by both the investigator (or an appropriate delegate) and the Medical Monitor.

ENDOCRINE EVENTS

Thyroid disorders, adrenal insufficiency, diabetes mellitus, and pituitary disorders have been associated with the administration of atezolizumab. Management guidelines for endocrine events are provided in Table 4.

Patients with unexplained symptoms such as headache, fatigue, myalgias, impotence, constipation, or mental status changes should be investigated for the presence of thyroid, pituitary, or adrenal endocrinopathies. The patient should be referred to an endocrinologist if an endocrinopathy is suspected. Thyroid-stimulating hormone (TSH) and free triiodothyronine and thyroxine levels should be measured to determine whether thyroid abnormalities are present. Pituitary hormone levels and function tests (e.g., TSH, growth hormone, luteinizing hormone, follicle-stimulating hormone, testosterone, prolactin, adrenocorticotropic hormone [ACTH] levels, and ACTH stimulation test) and magnetic resonance imaging (MRI) of the brain (with detailed pituitary sections) may help to differentiate primary pituitary insufficiency from primary adrenal insufficiency.

"Use of this document is governed by the terms of use on the first page of this document."

Table 4 Management Guidelines for Endocrine Events

Event	Management
Asymptomatic hypothyroidism	• Continue atezolizumab.<br>• Initiate treatment with thyroid replacement hormone.<br>• Monitor TSH weekly.
Symptomatic hypothyroidism	• Withhold atezolizumab.<br>• Initiate treatment with thyroid replacement hormone.<br>• Monitor TSH weekly.<br>• Consider patient referral to endocrinologist.<br>• Resume atezolizumab when symptoms are controlled and thyroid function is improving.
Asymptomatic hyperthyroidism	**TSH ≥ 0.1 mU/L and < 0.5 mU/L:**<br>• Continue atezolizumab.<br>• Monitor TSH every 4 weeks.<br>**TSH < 0.1 mU/L:**<br>• Follow guidelines for symptomatic hyperthyroidism.
Symptomatic hyperthyroidism	• Withhold atezolizumab.<br>• Initiate treatment with anti-thyroid drug such as methimazole or carbimazole as needed.<br>• Consider patient referral to endocrinologist.<br>• Resume atezolizumab when symptoms are controlled and thyroid function is improving.<br>• Permanently discontinue atezolizumab and contact Medical Monitor for life-threatening *immune-mediated* hyperthyroidism. [c]

MRI = magnetic resonance imaging; TSH = thyroid-stimulating hormone.

[a] Atezolizumab may be withheld for a longer period of time (i.e., > 12 weeks after event onset) to allow for corticosteroids (if initiated) to be reduced to ≤ 10 mg/day oral prednisone or equivalent. The acceptable length of the extended period of time must be agreed upon by the investigator and the Medical Monitor.

[b] If corticosteroids have been initiated, they must be tapered over ≥ 1 month to ≤ 10 mg/day oral prednisone or equivalent before atezolizumab can be resumed.

[c] Resumption of atezolizumab may be considered in patients who are deriving benefit and have fully recovered from the *immune-mediated* event. Patients can be re-challenged with atezolizumab only after approval has been documented by both the investigator (or an appropriate delegate) and the Medical Monitor.

"Use of this document is governed by the terms of use on the first page of this document."

Table 4 Management Guidelines for Endocrine Events (cont.)

Event	Management
Symptomatic adrenal insufficiency, Grade 2–4	• Withhold atezolizumab for up to 12 weeks after event onset. [a]<br>• Refer patient to endocrinologist.<br>• Perform appropriate imaging.<br>• Initiate treatment with corticosteroids equivalent to 1–2 mg/kg/day IV methylprednisolone and convert to 1–2 mg/kg/day oral prednisone or equivalent upon improvement.<br>• If event resolves to Grade 1 or better and patient is stable on replacement therapy, resume atezolizumab. [b]<br>• If event does not resolve to Grade 1 or better or patient is not stable on replacement therapy while withholding atezolizumab, permanently discontinue atezolizumab and contact Medical Monitor. [c]
Hyperglycemia, Grade 1 or 2	• Continue atezolizumab.<br>• Investigate for diabetes. If patient has Type 1 diabetes, treat as a Grade 3 event. If patient does not have Type 1 diabetes, treat as per institutional guidelines.<br>• Monitor for glucose control.
Hyperglycemia, Grade 3 or 4	• Withhold atezolizumab.<br>• Initiate treatment with insulin.<br>• Monitor for glucose control.<br>• Resume atezolizumab when symptoms resolve and glucose levels are stable.

MRI = magnetic resonance imaging; TSH = thyroid-stimulating hormone.

[a] Atezolizumab may be withheld for a longer period of time (i.e., > 12 weeks after event onset) to allow for corticosteroids (if initiated) to be reduced to ≤ 10 mg/day oral prednisone or equivalent. The acceptable length of the extended period of time must be agreed upon by the investigator and the Medical Monitor.

[b] If corticosteroids have been initiated, they must be tapered over ≥ 1 month to ≤ 10 mg/day oral prednisone or equivalent before atezolizumab can be resumed.

[c] Resumption of atezolizumab may be considered in patients who are deriving benefit and have fully recovered from the *immune-mediated* event. Patients can be re-challenged with atezolizumab only after approval has been documented by both the investigator (or an appropriate delegate) and the Medical Monitor.

"Use of this document is governed by the terms of use on the first page of this document."

Table 4 Management Guidelines for Endocrine Events (cont.)

Event	Management
Hypophysitis (pan-hypopituitarism), Grade 2 or 3	• Withhold atezolizumab for up to 12 weeks after event onset. [a]<br>• Refer patient to endocrinologist.<br>• Perform brain MRI (pituitary protocol).<br>• Initiate treatment with corticosteroids equivalent to 1–2 mg/kg/day IV methylprednisolone and convert to 1–2 mg/kg/day oral prednisone or equivalent upon improvement.<br>• Initiate hormone replacement if clinically indicated.<br>• If event resolves to Grade 1 or better, resume atezolizumab. [b]<br>• If event does not resolve to Grade 1 or better while withholding atezolizumab, permanently discontinue atezolizumab and contact Medical Monitor. [c]<br>• For recurrent hypophysitis, treat as a Grade 4 event.
Hypophysitis (pan-hypopituitarism), Grade 4	• Permanently discontinue atezolizumab and contact Medical Monitor. [c]<br>• Refer patient to endocrinologist.<br>• Perform brain MRI (pituitary protocol).<br>• Initiate treatment with corticosteroids equivalent to 1–2 mg/kg/day IV methylprednisolone and convert to 1–2 mg/kg/day oral prednisone or equivalent upon improvement.<br>• Initiate hormone replacement if clinically indicated.

MRI = magnetic resonance imaging; TSH = thyroid-stimulating hormone.

[a] Atezolizumab may be withheld for a longer period of time (i.e., > 12 weeks after event onset) to allow for corticosteroids (if initiated) to be reduced to ≤ 10 mg/day oral prednisone or equivalent. The acceptable length of the extended period of time must be agreed upon by the investigator and the Medical Monitor.

[b] If corticosteroids have been initiated, they must be tapered over ≥ 1 month to ≤ 10 mg/day oral prednisone or equivalent before atezolizumab can be resumed.

[c] Resumption of atezolizumab may be considered in patients who are deriving benefit and have fully recovered from the *immune-mediated* event. Patients can be re-challenged with atezolizumab only after approval has been documented by both the investigator (or an appropriate delegate) and the Medical Monitor.

"Use of this document is governed by the terms of use on the first page of this document."

OCULAR EVENTS

An ophthalmologist should evaluate visual complaints (e.g., uveitis, retinal events). Management guidelines for ocular events are provided in Table 5.

Table 5 Management Guidelines for Ocular Events

Event	Management
Ocular event, Grade 1	• Continue atezolizumab.<br>• Patient referral to ophthalmologist is strongly recommended.<br>• Initiate treatment with topical corticosteroid eye drops and topical immunosuppressive therapy.<br>• If symptoms persist, treat as a Grade 2 event.
Ocular event, Grade 2	• Withhold atezolizumab for up to 12 weeks after event onset. [a]<br>• Patient referral to ophthalmologist is strongly recommended.<br>• Initiate treatment with topical corticosteroid eye drops and topical immunosuppressive therapy.<br>• If event resolves to Grade 1 or better, resume atezolizumab. [b]<br>• If event does not resolve to Grade 1 or better while withholding atezolizumab, permanently discontinue atezolizumab and contact Medical Monitor. [c]
Ocular event, Grade 3 or 4	• Permanently discontinue atezolizumab and contact Medical Monitor. [c]<br>• Refer patient to ophthalmologist.<br>• Initiate treatment with corticosteroids equivalent to 1–2 mg/kg/day oral prednisone.<br>• If event resolves to Grade 1 or better, taper corticosteroids over ≥ 1 month.

[a] Atezolizumab may be withheld for a longer period of time (i.e., > 12 weeks after event onset) to allow for corticosteroids (if initiated) to be reduced to ≤ 10 mg/day oral prednisone or equivalent. The acceptable length of the extended period of time must be agreed upon by the investigator and the Medical Monitor.

[b] If corticosteroids have been initiated, they must be tapered over ≥ 1 month to ≤ 10 mg/day oral prednisone or equivalent before atezolizumab can be resumed.

[c] Resumption of atezolizumab may be considered in patients who are deriving benefit and have fully recovered from the *immune-mediated* event. Patients can be re-challenged with atezolizumab only after approval has been documented by both the investigator (or an appropriate delegate) and the Medical Monitor.

"Use of this document is governed by the terms of use on the first page of this document."

IMMUNE-MEDIATED MYOCARDITIS

Immune-mediated myocarditis has been associated with the administration of atezolizumab. *Immune-mediated* myocarditis should be suspected in any patient presenting with signs or symptoms suggestive of myocarditis, including, but not limited to, *laboratory (e.g., B-type natriuretic peptide) or cardiac imaging abnormalities,* dyspnea, chest pain, palpitations, fatigue, decreased exercise tolerance, or syncope. *Immune-mediated* myocarditis needs to be distinguished from myocarditis resulting from infection (commonly viral, e.g., in a patient who reports a recent history of gastrointestinal illness), ischemic events, underlying arrhythmias, exacerbation of preexisting cardiac conditions, or progression of malignancy.

All patients with possible myocarditis should be urgently evaluated by performing cardiac enzyme assessment, an ECG, a chest X-ray, an echocardiogram, and a cardiac MRI as appropriate per institutional guidelines. A cardiologist should be consulted. An endomyocardial biopsy may be considered to enable a definitive diagnosis and appropriate treatment, if clinically indicated.

Patients with signs and symptoms of myocarditis, in the absence of an identified alternate etiology, should be treated according to the guidelines in Table 6.

"Use of this document is governed by the terms of use on the first page of this document."

Table 6 Management Guidelines for *Immune-Mediated* Myocarditis

Event	Management
Immune-mediated myocarditis, Grade 1	• Refer patient to cardiologist.<br>• Initiate treatment as per institutional guidelines.
Immune-mediated myocarditis, Grade 2	• Withhold atezolizumab for up to 12 weeks after event onset [a] and contact Medical Monitor.<br>• Refer patient to cardiologist.<br>• Initiate treatment as per institutional guidelines and consider antiarrhythmic drugs, temporary pacemaker, ECMO, or VAD as appropriate.<br>• Consider treatment with corticosteroids equivalent to 1–2 mg/kg/day IV methylprednisolone or equivalent and convert to 1–2 mg/kg/day oral prednisone upon improvement.<br>• If event resolves to Grade 1 or better, resume atezolizumab. [b]<br>• If event does not resolve to Grade 1 or better while withholding atezolizumab, permanently discontinue atezolizumab and contact Medical Monitor. [c]
Immune-mediated myocarditis, Grade 3–4	• Permanently discontinue atezolizumab and contact Medical Monitor. [c]<br>• Refer patient to cardiologist.<br>• Initiate treatment as per institutional guidelines and consider antiarrhythmic drugs, temporary pacemaker, ECMO, or VAD as appropriate.<br>• Initiate treatment with corticosteroids equivalent to 1–2 mg/kg/day IV methylprednisolone and convert to 1–2 mg/kg/day oral prednisone or equivalent upon improvement.<br>• If event does not improve within 48 hours after initiating corticosteroids, consider adding an immunosuppressive agent.<br>• If event resolves to Grade 1 or better, taper corticosteroids over ≥ 1 month.

ECMO = extracorporeal membrane oxygenation; VAD = ventricular assist device.

[a] Atezolizumab may be withheld for a longer period of time (i.e., > 12 weeks after event onset) to allow for corticosteroids (if initiated) to be reduced to ≤ 10 mg/day oral prednisone or equivalent. The acceptable length of the extended period of time must be agreed upon by the investigator and the Medical Monitor.

[b] If corticosteroids have been initiated, they must be tapered over ≥ 1 month to ≤ 10 mg/day oral prednisone or equivalent before atezolizumab can be resumed.

[c] Resumption of atezolizumab may be considered in patients who are deriving benefit and have fully recovered from the *immune-mediated* event. Patients can be re-challenged with atezolizumab only after approval has been documented by both the investigator (or an appropriate delegate) and the Medical Monitor.

"Use of this document is governed by the terms of use on the first page of this document."

INFUSION-RELATED REACTIONS *AND CYTOKINE-RELEASE SYNDROME*

No premedication is indicated for the administration of Cycle 1 of atezolizumab. However, patients who experience an infusion-related reaction (IRR) *or cytokine-release syndrome (CRS)* with atezolizumab may receive premedication with antihistamines, anti-pyretics, *and/or* analgesics (e.g., acetaminophen) for subsequent infusions. Metamizole (dipyrone) is prohibited in treating atezolizumab-associated IRRs because of its potential for causing agranulocytosis.

IRRs are known to occur with the administration of monoclonal antibodies and have been reported with atezolizumab. These reactions, which are thought to be due to release of cytokines and/or other chemical mediators, occur within 24 hours of atezolizumab administration and are generally mild to moderate in severity.

CRS is defined as a supraphysiologic response following administration of any immune therapy that results in activation or engagement of endogenous or infused T cells and/or other immune effector cells. Symptoms can be progressive, always include fever at the onset, and may include hypotension, capillary leak (hypoxia), and end-organ dysfunction (Lee et al. 2019). CRS has been well documented with chimeric antigen receptor T-cell therapies and bispecific T-cell engager antibody therapies but has also been reported with immunotherapies that target PD-1 or PD-L1 (Rotz et al. 2017; Adashek and Feldman 2019), including atezolizumab.

There may be significant overlap in signs and symptoms of IRRs and CRS, and in recognition of the challenges in clinically distinguishing between the two, consolidated guidelines for medical management of IRRs *and CRS* are provided in Table 7.

"Use of this document is governed by the terms of use on the first page of this document."

Table 7 Management Guidelines for Infusion-Related Reactions *and Cytokine-Release Syndrome*

Event	Management
Grade 1 [a]<br>*Fever [b] with or without constitutional symptoms*	• *Immediately interrupt infusion.*<br>• *Upon symptom resolution, wait for 30 minutes and then restart infusion at* half the rate being given at the time of event onset.<br>• If the infusion is tolerated at the reduced rate for 30 minutes, the infusion rate may be increased to the original rate.<br>• *If symptoms recur, discontinue infusion of this dose.*<br>• *Administer symptomatic treatment, [c] including maintenance of IV fluids for hydration.*<br>• *In case of rapid decline or prolonged CRS (>2 days) or in patients with significant symptoms and/or comorbidities, consider managing as per Grade 2.*<br>• *For subsequent infusions, consider administration of oral premedication with antihistamines, anti-pyretics, and/or analgesics, and monitor closely for IRRs and/or CRS.*

"Use of this document is governed by the terms of use on the first page of this document."

Table 7 Management Guidelines for Infusion-Related Reactions and Cytokine-Release Syndrome (cont.)

Grade 2 [a]<br>*Fever [b] with hypotension not requiring vasopressors*<br>***and/or***<br>*Hypoxia requiring low-flow oxygen [d] by nasal cannula or blow-by*	• I*mmediately i*nterrupt infusion.<br>• *Upon symptom resolution, wait for 30 minutes and then restart infusion at half the rate being given at the time of event onset.*<br>• *If symptoms recur, discontinue infusion of this dose.*<br>• *Administer symptomatic treatment.* [c]<br>• *For hypotension, administer IV fluid bolus as needed.*<br>• *Monitor cardiopulmonary and other organ function closely (in the ICU, if appropriate). Administer IV fluids as clinically indicated, and manage constitutional symptoms and organ toxicities as per institutional practice.*<br>• *Rule out other inflammatory conditions that can mimic CRS (e.g., sepsis). If no improvement within 24 hours, initiate workup and assess for signs and symptoms of HLH or MAS as described in this appendix.*<br>• *Consider IV corticosteroids (e.g., methylprednisolone 2 mg/kg/day or dexamethasone 10 mg every 6 hours).*<br>• *Consider anti-cytokine therapy.* [e]<br>• *Consider hospitalization until complete resolution of symptoms. If no improvement within 24 hours, manage as per Grade 3, that is, hospitalize patient (monitoring in the ICU is recommended), permanently discontinue atezolizumab, and contact Medical Monitor.*<br>• *If symptoms resolve to Grade 1 or better for 3 consecutive days, the next dose of atezolizumab may be administered.*<br>• For subsequent infusions, consider administration of oral premedication with antihistamines, anti-pyretics, and/or analgesics and monitor closely for IRRs *and/or CRS*.<br>• *If symptoms do not resolve to Grade 1 or better for 3 consecutive days, contact Medical Monitor.*

"Use of this document is governed by the terms of use on the first page of this document."

Table 7 Management Guidelines for Infusion-Related Reactions and Cytokine-Release Syndrome (cont.)

Grade 3 [a]<br>*Fever [b] with hypotension requiring a vasopressor (with or without vasopressin)*<br>***and/or***<br>*Hypoxia requiring high-flow oxygen [d] by nasal cannula, face mask, non-rebreather mask, or Venturi mask*	• Permanently discontinue atezolizumab and contact Medical Monitor. [f]<br>• *Administer symptomatic treatment.* [c]<br>• *For hypotension, administer IV fluid bolus and vasopressor as needed.*<br>• *Monitor cardiopulmonary and other organ function closely; monitoring in the ICU is recommended. Administer IV fluids as clinically indicated, and manage constitutional symptoms and organ toxicities as per institutional practice.*<br>• *Rule out other inflammatory conditions that can mimic CRS (e.g., sepsis). If no improvement within 24 hours, initiate workup and assess for signs and symptoms of HLH or MAS as described in this appendix.*<br>• *Administer IV corticosteroids (e.g., methylprednisolone 2 mg/kg/day or dexamethasone 10 mg every 6 hours).*<br>• *Consider anti-cytokine therapy.* [e]<br>• *Hospitalize patient until complete resolution of symptoms. If no improvement within 24 hours, manage as per Grade 4, that is, admit patient to ICU and initiate hemodynamic monitoring, mechanical ventilation, and/or IV fluids and vasopressors as needed; for patients who are refractory to anti-cytokine therapy, experimental treatments may be considered at the discretion of the investigator and in consultation with the Medical Monitor.*
Grade 4 [a]<br>*Fever [b] with hypotension requiring multiple vasopressors (excluding vasopressin)*<br>***and/or***<br>*Hypoxia requiring oxygen by positive pressure (e.g., CPAP, BiPAP, intubation and mechanical ventilation)*	• *Permanently discontinue atezolizumab and contact Medical Monitor.* [f]<br>• *Administer symptomatic treatment.* [c]<br>• *Admit patient to ICU and initiate hemodynamic monitoring, mechanical ventilation, and/or IV fluids and vasopressors as needed. Monitor other organ function closely. Manage constitutional symptoms and organ toxicities as per institutional practice.*<br>• *Rule out other inflammatory conditions that can mimic CRS (e.g., sepsis). If no improvement within 24 hours, initiate workup and assess for signs and symptoms of HLH or MAS as described in this appendix.*<br>• *Administer IV corticosteroids (e.g., methylprednisolone 2 mg/kg/day or dexamethasone 10 mg every 6 hours).*<br>• *Consider anti-cytokine therapy.* [e] *For patients who are refractory to anti-cytokine therapy, experimental treatments* [g] *may be considered at the discretion of the investigator and in consultation with the Medical Monitor.*<br>• *Hospitalize patient until complete resolution of symptoms.*

"Use of this document is governed by the terms of use on the first page of this document."

Table 7 Management Guidelines for Infusion-Related Reactions and Cytokine-Release Syndrome (cont.)

ASTCT = American Society for Transplantation and Cellular Therapy; BiPAP = bi-level positive airway pressure; CAR = chimeric antigen receptor; CPAP = continuous positive airway pressure; CRS = cytokine-release syndrome; CTCAE = Common Terminology Criteria for Adverse Events; eCRF = electronic Case Report Form; HLH = hemophagocytic lymphohistiocytosis; ICU = intensive care unit; IRR = infusion-related reaction*; MAS = macrophage activation syndrome; NCCN = National Cancer Comprehensive Network; NCI = National Cancer Institute*.

Note: The management guidelines have been adapted from NCCN guidelines for management of CAR T-cell–related toxicities (Version 2.2019).

[a] *Grading system for management guidelines is based on ASTCT consensus grading for CRS. NCI CTCAE v4.0 should be used when reporting severity of IRRs, CRS, or organ toxicities associated with CRS on the Adverse Event eCRF. Organ toxicities associated with CRS should not influence overall CRS grading.*

[b] *Fever is defined as temperature ≥ 38°C not attributable to any other cause. In patients who develop CRS and then receive anti-pyretic, anti-cytokine, or corticosteroid therapy, fever is no longer required when subsequently determining event severity (grade). In this case, the grade is driven by the presence of hypotension and/or hypoxia.*

[c] *Symptomatic treatment may include oral or IV antihistamines, anti-pyretics, analgesics, bronchodilators, and/or oxygen. For bronchospasm, urticaria, or dyspnea, additional treatment may be administered as per institutional practice.*

[d] *Low flow is defined as oxygen delivered at ≤ 6 L/min, and high flow is defined as oxygen delivered at > 6 L/min.*

[e] *There are case reports where anti-cytokine therapy has been used for treatment of CRS with immune checkpoint inhibitors (Rotz et al. 2017; Adashek and Feldman 2019), but data are limited, and the role of such treatment in the setting of antibody-associated CRS has not been established.*

[f] Resumption of atezolizumab may be considered in patients who are deriving benefit and have fully recovered from the event. Patients can be re-challenged with atezolizumab only after approval has been documented by both the investigator (or an appropriate delegate) and the Medical Monitor. *For subsequent infusions, administer oral premedication with antihistamines, anti-pyretics, and/or analgesics, and monitor closely for IRRs and/or CRS. Premedication with corticosteroids and extending the infusion time may also be considered after consulting the Medical Monitor and considering the benefit–risk ratio.*

[g] *Refer to Riegler et al. (2019) for information on experimental treatments for CRS.*

"Use of this document is governed by the terms of use on the first page of this document."

PANCREATIC EVENTS

Symptoms of abdominal pain associated with elevations of amylase and lipase, suggestive of pancreatitis, have been associated with the administration of atezolizumab. The differential diagnosis of acute abdominal pain should include pancreatitis. Appropriate work-up should include an evaluation for ductal obstruction, as well as serum amylase and lipase tests. Management guidelines for pancreatic events, including pancreatitis, are provided in Table 8.

Table 8 Management Guidelines for Pancreatic Events, Including Pancreatitis

Event	Management
Amylase and/or lipase elevation, Grade 2	• Continue atezolizumab.<br>• Monitor amylase and lipase weekly.<br>• For prolonged elevation (e.g., > 3 weeks), consider treatment with 10 mg/day oral prednisone or equivalent.
Amylase and/or lipase elevation, Grade 3 or 4	• Withhold atezolizumab for up to 12 weeks after event onset. [a]<br>• Refer patient to GI specialist.<br>• Monitor amylase and lipase every other day.<br>• If no improvement, consider treatment with 1–2 mg/kg/day oral prednisone or equivalent.<br>• If event resolves to Grade 1 or better, resume atezolizumab. [b]<br>• If event does not resolve to Grade 1 or better while withholding atezolizumab, permanently discontinue atezolizumab and contact Medical Monitor. [c]<br>• For recurrent events, permanently discontinue atezolizumab and contact Medical Monitor. [c]

GI = gastrointestinal.

[a] Atezolizumab may be withheld for a longer period of time (i.e., > 12 weeks after event onset) to allow for corticosteroids (if initiated) to be reduced to ≤ 10 mg/day oral prednisone or equivalent. The acceptable length of the extended period of time must be agreed upon by the investigator and the Medical Monitor.

[b] If corticosteroids have been initiated, they must be tapered over ≥ 1 month to ≤ 10 mg/day oral prednisone or equivalent before atezolizumab can be resumed.

[c] Resumption of atezolizumab may be considered in patients who are deriving benefit and have fully recovered from the *immune-mediated* event. Patients can be re-challenged with atezolizumab only after approval has been documented by both the investigator (or an appropriate delegate) and the Medical Monitor.

"Use of this document is governed by the terms of use on the first page of this document."

Table 8 Management Guidelines for Pancreatic Events, Including Pancreatitis (cont.)

Event	Management
Immune-mediated pancreatitis, Grade 2 or 3	• Withhold atezolizumab for up to 12 weeks after event onset. [a]<br>• Refer patient to GI specialist.<br>• Initiate treatment with corticosteroids equivalent to 1–2 mg/kg/day IV methylprednisolone and convert to 1–2 mg/kg/day oral prednisone or equivalent upon improvement.<br>• If event resolves to Grade 1 or better, resume atezolizumab. [b]<br>• If event does not resolve to Grade 1 or better while withholding atezolizumab, permanently discontinue atezolizumab and contact Medical Monitor. [c]<br>• For recurrent events, permanently discontinue atezolizumab and contact Medical Monitor. [c]
Immune-mediated pancreatitis, Grade 4	• Permanently discontinue atezolizumab and contact Medical Monitor. [c]<br>• Refer patient to GI specialist.<br>• Initiate treatment with corticosteroids equivalent to 1–2 mg/kg/day IV methylprednisolone and convert to 1–2 mg/kg/day oral prednisone or equivalent upon improvement.<br>• If event does not improve within 48 hours after initiating corticosteroids, consider adding an immunosuppressive agent.<br>• If event resolves to Grade 1 or better, taper corticosteroids over ≥ 1 month.

GI = gastrointestinal.

[a] Atezolizumab may be withheld for a longer period of time (i.e., > 12 weeks after event onset) to allow for corticosteroids (if initiated) to be reduced to ≤ 10 mg/day oral prednisone or equivalent. The acceptable length of the extended period of time must be agreed upon by the investigator and the Medical Monitor.

[b] If corticosteroids have been initiated, they must be tapered over ≥ 1 month to ≤ 10 mg/day oral prednisone or equivalent before atezolizumab can be resumed.

[c] Resumption of atezolizumab may be considered in patients who are deriving benefit and have fully recovered from the *immune-mediated* event. Patients can be re-challenged with atezolizumab only after approval has been documented by both the investigator (or an appropriate delegate) and the Medical Monitor.

"Use of this document is governed by the terms of use on the first page of this document."

DERMATOLOGIC EVENTS

Treatment-emergent rash has been associated with atezolizumab. The majority of cases of rash were mild in severity and self limited, with or without pruritus.
A dermatologist should evaluate persistent and/or severe rash or pruritus. A biopsy should be considered unless contraindicated. Management guidelines for dermatologic events are provided in Table 9.

Table 9 Management Guidelines for Dermatologic Events

Event	Management
Dermatologic event, Grade 1	• Continue atezolizumab.<br>• Consider treatment with topical corticosteroids and/or other symptomatic therapy (e.g., antihistamines).
Dermatologic event, Grade 2	• Continue atezolizumab.<br>• Consider patient referral to dermatologist.<br>• Initiate treatment with topical corticosteroids.<br>• Consider treatment with higher-potency topical corticosteroids if event does not improve.
Dermatologic event, Grade 3	• Withhold atezolizumab for up to 12 weeks after event onset. [a]<br>• Refer patient to dermatologist.<br>• Initiate treatment with corticosteroids equivalent to 10 mg/day oral prednisone, increasing dose to 1–2 mg/kg/day if event does not improve within 48–72 hours.<br>• If event resolves to Grade 1 or better, resume atezolizumab. [b]<br>• If event does not resolve to Grade 1 or better while withholding atezolizumab, permanently discontinue atezolizumab and contact Medical Monitor. [c]
Dermatologic event, Grade 4	• Permanently discontinue atezolizumab and contact Medical Monitor. [c]

[a] Atezolizumab may be withheld for a longer period of time (i.e., $>$ 12 weeks after event onset) to allow for corticosteroids (if initiated) to be reduced to $\leq$ 10 mg/day oral prednisone or equivalent. The acceptable length of the extended period of time must be agreed upon by the investigator and the Medical Monitor.

[b] If corticosteroids have been initiated, they must be tapered over $\geq$ 1 month to $\leq$ 10 mg/day oral prednisone or equivalent before atezolizumab can be resumed.

[c] Resumption of atezolizumab may be considered in patients who are deriving benefit and have fully recovered from the *immune-mediated* event. Patients can be re-challenged with atezolizumab only after approval has been documented by both the investigator (or an appropriate delegate) and the Medical Monitor.

"Use of this document is governed by the terms of use on the first page of this document."

NEUROLOGIC DISORDERS

Myasthenia gravis and Guillain-Barré syndrome have been observed with single-agent atezolizumab. Patients may present with signs and symptoms of sensory and/or motor neuropathy. Diagnostic work-up is essential for an accurate characterization to differentiate between alternative etiologies. Management guidelines for neurologic disorders are provided in Table 10.

Table 10 Management Guidelines for Neurologic Disorders

Event	Management
Immune-mediated neuropathy, Grade 1	• Continue atezolizumab.<br>• Investigate etiology.
Immune-mediated neuropathy, Grade 2	• Withhold atezolizumab for up to 12 weeks after event onset. [a]<br>• Investigate etiology.<br>• Initiate treatment as per institutional guidelines.<br>• If event resolves to Grade 1 or better, resume atezolizumab. [b]<br>• If event does not resolve to Grade 1 or better while withholding atezolizumab, permanently discontinue atezolizumab and contact Medical Monitor. [c]
Immune-mediated neuropathy, Grade 3 or 4	• Permanently discontinue atezolizumab and contact Medical Monitor. [c]<br>• Initiate treatment as per institutional guidelines.
Myasthenia gravis and Guillain-Barré syndrome (any grade)	• Permanently discontinue atezolizumab and contact Medical Monitor. [c]<br>• Refer patient to neurologist.<br>• Initiate treatment as per institutional guidelines.<br>• Consider initiation of corticosteroids equivalent to 1–2 mg/kg/day oral or IV prednisone.

[a] Atezolizumab may be withheld for a longer period of time (i.e., > 12 weeks after event onset) to allow for corticosteroids (if initiated) to be reduced to ≤ 10 mg/day oral prednisone or equivalent. The acceptable length of the extended period of time must be agreed upon by the investigator and the Medical Monitor.

[b] If corticosteroids have been initiated, they must be tapered over ≥ 1 month to ≤ 10 mg/day oral prednisone or equivalent before atezolizumab can be resumed.

[c] Resumption of atezolizumab may be considered in patients who are deriving benefit and have fully recovered from the *immune-mediated* event. Patients can be re-challenged with atezolizumab only after approval has been documented by both the investigator (or an appropriate delegate) and the Medical Monitor.

"Use of this document is governed by the terms of use on the first page of this document."

IMMUNE-MEDIATED MENINGOENCEPHALITIS

Immune-mediated meningoencephalitis is an identified risk associated with the administration of atezolizumab. *Immune-mediated* meningoencephalitis should be suspected in any patient presenting with signs or symptoms suggestive of meningitis or encephalitis, including, but not limited to, headache, neck pain, confusion, seizure, motor or sensory dysfunction, and altered or depressed level of consciousness. Encephalopathy from metabolic or electrolyte imbalances needs to be distinguished from potential meningoencephalitis resulting from infection (bacterial, viral, or fungal) or progression of malignancy, or secondary to a paraneoplastic process.

All patients being considered for meningoencephalitis should be urgently evaluated with a CT scan and/or MRI scan of the brain to evaluate for metastasis, inflammation, or edema. If deemed safe by the treating physician, a lumbar puncture should be performed and a neurologist should be consulted.

Patients with signs and symptoms of meningoencephalitis, in the absence of an identified alternate etiology, should be treated according to the guidelines in Table 11.

Table 11 Management Guidelines for *Immune-Mediated* Meningoencephalitis

Event	Management
Immune-mediated meningoencephalitis, all grades	• Permanently discontinue atezolizumab and contact Medical Monitor. [a]<br>• Refer patient to neurologist.<br>• Initiate treatment with corticosteroids equivalent to 1–2 mg/kg/day IV methylprednisolone or equivalent and convert to 1–2 mg/kg/day oral prednisone upon improvement.<br>• If event does not improve within 48 hours after initiating corticosteroids, consider adding an immunosuppressive agent.<br>• If event resolves to Grade 1 or better, taper corticosteroids over ≥ 1 month.

[a] Resumption of atezolizumab may be considered in patients who are deriving benefit and have fully recovered from the *immune-mediated* event. Patients can be re-challenged with atezolizumab only after approval has been documented by both the investigator (or an appropriate delegate) and the Medical Monitor.

RENAL EVENTS

Immune-mediated nephritis has been associated with the administration of atezolizumab. Eligible patients must have adequate renal function. *Renal* function, including serum creatinine, should be monitored throughout study treatment. Patients with abnormal renal function should be evaluated and treated for other more common etiologies

"Use of this document is governed by the terms of use on the first page of this document."

(including prerenal and postrenal causes, and concomitant medications such as non-steroidal anti-inflammatory drugs). Refer the patient to a renal specialist if clinically indicated. A renal biopsy may be required to enable a definitive diagnosis and appropriate treatment.

Patients with signs and symptoms of nephritis, in the absence of an identified alternate etiology, should be treated according to the guidelines in Table 12.

Table 12 Management Guidelines for Renal Events

Event	Management
Renal event, Grade 1	• Continue atezolizumab.<br>• Monitor kidney function, including creatinine, closely until values resolve to within normal limits or to baseline values.
Renal event, Grade 2	• Withhold atezolizumab for up to 12 weeks after event onset. [a]<br>• Refer patient to renal specialist.<br>• Initiate treatment with corticosteroids equivalent to 1–2 mg/kg/day oral prednisone.<br>• If event resolves to Grade 1 or better, resume atezolizumab. [b]<br>• If event does not resolve to Grade 1 or better while withholding atezolizumab, permanently discontinue atezolizumab and contact Medical Monitor. [c]
Renal event, Grade 3 or 4	• Permanently discontinue atezolizumab and contact Medical Monitor.<br>• Refer patient to renal specialist and consider renal biopsy.<br>• Initiate treatment with corticosteroids equivalent to 1–2 mg/kg/day oral prednisone.<br>• If event does not improve within 48 hours after initiating corticosteroids, consider adding an immunosuppressive agent.<br>• If event resolves to Grade 1 or better, taper corticosteroids over ≥ 1 month.

[a] Atezolizumab may be withheld for a longer period of time (i.e., > 12 weeks after event onset) to allow for corticosteroids (if initiated) to be reduced to the equivalent of ≤ 10 mg/day oral prednisone. The acceptable length of the extended period of time must be agreed upon by the investigator and the Medical Monitor.

[b] If corticosteroids have been initiated, they must be tapered over ≥ 1 month to the equivalent of ≤ 10 mg/day oral prednisone before atezolizumab can be resumed.

[c] Resumption of atezolizumab may be considered in patients who are deriving benefit and have fully recovered from the *immune-mediated* event. Patients can be re-challenged with atezolizumab only after approval has been documented by both the investigator (or an appropriate delegate) and the Medical Monitor.

"Use of this document is governed by the terms of use on the first page of this document."

IMMUNE-MEDIATED MYOSITIS

Immune-mediated myositis has been associated with the administration of atezolizumab. Myositis or inflammatory myopathies are a group of disorders sharing the common feature of inflammatory muscle injury; dermatomyositis and polymyositis are among the most common disorders. Initial diagnosis is based on clinical (muscle weakness, muscle pain, skin rash in dermatomyositis), biochemical (serum creatine kinase increase), and imaging (electromyography/MRI) features, and is confirmed with a muscle biopsy.

Patients with signs and symptoms of myositis, in the absence of an identified alternate etiology, should be treated according to the guidelines in Table 13.

Table 13 Management Guidelines for *Immune-Mediated* Myositis

Event	Management
Immune-mediated myositis, Grade 1	• Continue atezolizumab.<br>• Refer patient to rheumatologist or neurologist.<br>• Initiate treatment as per institutional guidelines.
Immune-mediated myositis, Grade 2	• Withhold atezolizumab for up to 12 weeks after event onset [a] and contact Medical Monitor.<br>• Refer patient to rheumatologist or neurologist.<br>• Initiate treatment as per institutional guidelines.<br>• Consider treatment with corticosteroids equivalent to 1–2 mg/kg/day IV methylprednisolone and convert to 1–2 mg/kg/day oral prednisone or equivalent upon improvement.<br>• If corticosteroids are initiated and event does not improve within 48 hours after initiating corticosteroids, consider adding an immunosuppressive agent.<br>• If event resolves to Grade 1 or better, resume atezolizumab. [b]<br>• If event does not resolve to Grade 1 or better while withholding atezolizumab, permanently discontinue atezolizumab and contact Medical Monitor. [c]

[a] Atezolizumab may be withheld for a longer period of time (i.e., > 12 weeks after event onset) to allow for corticosteroids (if initiated) to be reduced to the equivalent of ≤ 10 mg/day oral prednisone. The acceptable length of the extended period of time must be agreed upon by the investigator and the Medical Monitor.

[b] If corticosteroids have been initiated, they must be tapered over ≥ 1 month to the equivalent of ≤ 10 mg/day oral prednisone before atezolizumab can be resumed.

[c] Resumption of atezolizumab may be considered in patients who are deriving benefit and have fully recovered from the *immune-mediated* event. Patients can be re-challenged with atezolizumab only after approval has been documented by both the investigator (or an appropriate delegate) and the Medical Monitor.

"Use of this document is governed by the terms of use on the first page of this document."

Table 13 Management Guidelines for *Immune-Mediated* Myositis (cont.)

Immune-mediated myositis, Grade 3	• Withhold atezolizumab for up to 12 weeks after event onset [a] and contact Medical Monitor.<br>• Refer patient to rheumatologist or neurologist.<br>• Initiate treatment as per institutional guidelines.<br>• Respiratory support may be required in more severe cases.<br>• Initiate treatment with corticosteroids equivalent to 1–2 mg/kg/day IV methylprednisolone, or higher-dose bolus if patient is severely compromised (e.g., cardiac or respiratory symptoms, dysphagia, or weakness that severely limits mobility); convert to 1–2 mg/kg/day oral prednisone or equivalent upon improvement.<br>• If event does not improve within 48 hours after initiating corticosteroids, consider adding an immunosuppressive agent.<br>• If event resolves to Grade 1 or better, resume atezolizumab. [b]<br>• If event does not resolve to Grade 1 or better while withholding atezolizumab, permanently discontinue atezolizumab and contact Medical Monitor. [c]<br>• For recurrent events, treat as a Grade 4 event.
Immune-mediated myositis, Grade 4	• Permanently discontinue atezolizumab and contact Medical Monitor. [c]<br>• Refer patient to rheumatologist or neurologist.<br>• Initiate treatment as per institutional guidelines.<br>• Respiratory support may be required in more severe cases.<br>• Initiate treatment with corticosteroids equivalent to 1–2 mg/kg/day IV methylprednisolone, or higher-dose bolus if patient is severely compromised (e.g., cardiac or respiratory symptoms, dysphagia, or weakness that severely limits mobility); convert to 1–2 mg/kg/day oral prednisone or equivalent upon improvement.<br>• If event does not improve within 48 hours after initiating corticosteroids, consider adding an immunosuppressive agent.<br>• If event resolves to Grade 1 or better, taper corticosteroids over ≥ 1 month.

[a] Atezolizumab may be withheld for a longer period of time (i.e., > 12 weeks after event onset) to allow for corticosteroids (if initiated) to be reduced to the equivalent of ≤ 10 mg/day oral prednisone. The acceptable length of the extended period of time must be agreed upon by the investigator and the Medical Monitor.

[b] If corticosteroids have been initiated, they must be tapered over ≥ 1 month to the equivalent of ≤ 10 mg/day oral prednisone before atezolizumab can be resumed.

[c] Resumption of atezolizumab may be considered in patients who are deriving benefit and have fully recovered from the *immune-mediated* event. Patients can be re-challenged with atezolizumab only after approval has been documented by both the investigator (or an appropriate delegate) and the Medical Monitor.

"Use of this document is governed by the terms of use on the first page of this document."

HEMOPHAGOCYTIC LYMPHOHISTIOCYTOSIS AND MACROPHAGE ACTIVATION SYNDROME

Immune-mediated reactions may involve any organ system and may lead to hemophagocytic lymphohistiocytosis (HLH) and macrophage activation syndrome (MAS), which are considered to be potential risks for atezolizumab.

Patients with suspected HLH should be diagnosed according to published criteria by McClain and Eckstein (2014). A patient should be classified as having HLH if five of the following eight criteria are met:

- *Fever ≥38.5°C*
- *Splenomegaly*
- *Peripheral blood cytopenia consisting of at least two of the following:*
  - *Hemoglobin <90 g/L (9 g/dL) (<100 g/L [10 g/dL] for infants <4 weeks old)*
  - *Platelet count <100 × 10^9/L (100,000/μL)*
  - *ANC <1.0 × 10^9/L (1000/μL)*
- *Fasting triglycerides >2.992 mmol/L (265 mg/dL) and/or fibrinogen <1.5 g/L (150 mg/dL)*
- *Hemophagocytosis in bone marrow, spleen, lymph node, or liver*
- *Low or absent natural killer cell activity*
- *Ferritin >500 mg/L (500 ng/mL)*
- *Soluble interleukin 2 (IL-2) receptor (soluble CD25) elevated ≥2 standard deviations above age-adjusted laboratory-specific norms*

Patients with suspected MAS should be diagnosed according to published criteria for systemic juvenile idiopathic arthritis by Ravelli et al. (2016). A febrile patient should be classified as having MAS if the following criteria are met:

- *Ferritin >684 mg/L (684 ng/mL)*
- *At least two of the following:*
  - *Platelet count ≤181 × 10^9/L (181,000/μL)*
  - *AST ≥48 U/L*
  - *Triglycerides >1.761 mmol/L (156 mg/dL)*
  - *Fibrinogen ≤3.6 g/L (360 mg/dL)*

Patients with suspected HLH or MAS should be treated according to the guidelines in Table 14.

"Use of this document is governed by the terms of use on the first page of this document."

Table 14 Management Guidelines for Suspected Hemophagocytic Lymphohistiocytosis or Macrophage Activation Syndrome

Event	Management
Suspected HLH or MAS	• Permanently discontinue atezolizumab and contact Medical Monitor.<br>• Consider patient referral to hematologist.<br>• Initiate supportive care, including intensive care monitoring if indicated per institutional guidelines.<br>• Consider initiation of IV corticosteroids and/or an immunosuppressive agent.<br>• If event does not improve within 48 hours after initiating corticosteroids, consider adding an immunosuppressive agent.<br>• If event resolves to Grade 1 or better, taper corticosteroids over ≥1 month.

HLH = hemophagocytic lymphohistiocytosis; MAS = macrophage activation syndrome.

REFERENCES

Adashek ML, Feldman M. Cytokine release syndrome resulting from anti–programmed death-1 antibody: raising awareness among community oncologist. J Oncol Practice 2019;15:502–4.

McClain KL, Eckstein O. Clinical features and diagnosis of hemophagocytic lymphohistiocytosis. Up to Date [resource on the Internet]. 2014 [updated 29 October 2018; cited: 17 May 2019]. Available from: https://www.uptodate.com/contents/clinical-features-and-diagnosis-of-hemophagocytic-lymphohistiocytosis.

Ravelli A, Minoia F, Davi S, et al. 2016 classification criteria for macrophage activation syndrome complicating systemic juvenile idiopathic arthritis: a European League Against Rheumatism/American College of Rheumatology/Paediatric Rheumatology International Trials Organisation Collaborative Initiative. Ann Rheum Dis 2016;75:481–9.

Riegler LL, Jones GP, Lee DW. Current approaches in the grading and management of cytokine release syndrome after chimeric antigen receptor T-cell therapy. Ther Clin Risk Manag 2019;15:323–35.

Rotz SJ, Leino D, Szabo S, et al. Severe cytokine release syndrome in a patient receiving PD-1-directed therapy. Pediatr Blood Cancer 2017;64:e26642.

"Use of this document is governed by the terms of use on the first page of this document."

Appendix 14
Statistical Detail of the Adaptive Design for Study WO39392 (IMpassion031)

The intent-to-treat (ITT) as well as PD-L1−positive population will be used for the primary analysis of the co-primary endpoints of pathological complete response (pCR) in these populations as follows. The **overall one-sided type I error** for testing the co-primary endpoints of pCR is $\boldsymbol{\alpha = 0.025}$.

Two main sources for multiplicity in statistical testing from the current design are:

- Multiple target populations, namely ITT and PD-L1−positive populations
- Study having two stages with target population at Stage 2 depending on results from Stage 1

Simes' closed testing procedure will be used to address multiplicity in target populations and weighted inverse p-value combination method is applied to account for the adaptive choice of target population at Stage 2 as follows.

At Stage 1

For the interim analysis at end of Stage 1, the co-primary endpoints of pCR in the ITT and PD-L1−positive population will be tested at the pre-defined one-sided type I error of 0.0125 (i.e., 50% of the total type I error) following Simes' procedure. This involves the following p-values:

- $\mathbf{p_1^{\{F\}}}$ is the p-value from the **one-sided** Cochran-Mantel-Haenszel (CMH) χ^2 test for difference in pCR rates between treatment groups in the **ITT population** (Δ_F) stratified according to tumor PD-L1 status (IC0 vs. IC1/2/3) and clinical stage at presentation (Stage II vs. III). The associated elementary null hypothesis is denoted as $H_0^{\{F\}}$:$\Delta_F \leq 0$.
- $\mathbf{p_1^{\{S\}}}$ is the p-value from the CMH χ^2 test for difference in pCR rates between treatment groups in the **PD-L1−positive population** (Δ_S) stratified according to clinical stage at presentation (Stage II vs. III). The associated elementary null hypothesis is denoted as $H_0^{\{S\}}$:$\Delta_S \leq 0$.
- $\mathbf{p_1^{\{F,S\}}} = \min\left\{2\min\left(p_1^{\{S\}}, p_1^{\{F\}}\right), \max\left(p_1^{\{S\}}, p_1^{\{F\}}\right)\right\}$, p-value based on Simes' method for the intersection hypothesis $H_0^{\{F,S\}}$, which specifies that there is no proportion difference in either F or S.

In addition, the observed difference in pCR rates between arms in S, denoted as $\widehat{\Delta}_1^S$ and its complementary sub-population of patients with PD-L1 IC0 (C), denoted as $\widehat{\Delta}_1^C$ will also be computed.

"Use of this document is governed by the terms of use on the first page of this document."

Based on $p_1^{\{F\}}$, $p_1^{\{S\}}$, $p_1^{\{F,S\}}$, $\widehat{\Delta}_1^S$ and $\widehat{\Delta}_1^C$, the following decision could be made at Stage 1:

- If $p_1^{\{F,S\}} \leq \alpha_1$, the analysis at Stage 1 is considered the primary efficacy analysis for pCR with evidence for efficacy declared in either F (if $p_1^{\{F\}} \leq \alpha_1$) or S (if $p_1^{\{S\}} \leq \alpha_1$) or both, the study will therefore not enroll extra patients in Stage 2.
- Otherwise, if both $\widehat{\Delta}_1^S$ and $\widehat{\Delta}_1^C$ are below the pre-specified thresholds, $d_S = 0.12$ and $d_C = 0.1$, respectively, the study will also not enroll extra patients in Stage 2 for lack of efficacy.
- Otherwise, if $\widehat{\Delta}_1^S \geq d_S$ and $\widehat{\Delta}_1^C < d_C$, the study will enroll only patients in S at Stage 2, with S being the only target population in the primary efficacy analysis for pCR.
- Otherwise, if $\widehat{\Delta}_1^S < d_S$ and $\widehat{\Delta}_1^C \geq d_C$, the study will enroll all-comer patients (F) in Stage 2; however, only F will be the target population in the primary efficacy analysis for pCR.
- If $\widehat{\Delta}_1^S \geq d_S$ and $\widehat{\Delta}_1^C \geq d_C$, the study will enroll all-comer patients in Stage 2, and the co-primary efficacy endpoints of pCR in F and S will be considered in the primary efficacy analysis for pCR.

At Stage 2 (if needed)

If the study advances to Stage 2, at the primary efficacy analysis for pCR to protect the overall α the corresponding boundary for statistical significance is α_2=0.0184, accounting for the fact that and overall $\alpha_1 = 0.0125$ having been spent at Stage 1 and the information fraction at Stage 1, namely $w_1 = N_1/(N_1 + N_2)$. Indeed, the p-value p_1 based on Stage 1 data only and the combination p-value p_2 have the same joint distribution under the null hypothesis as the p-values from a group-sequential test with two stages at information times $t_1 = w_1$ and $t_2 = 1$. Thus, standard statistical software for group sequential designs can be used for the determination of critical values for the adaptive design (Wassmer and Brannath 2016, Section 6.2.4).

Final test(s) involving the target population(s) selected at Stage 1 will be based on weighted inverse normal p-value combination method (Wassmer and Brannath 2016) as follows:

Let $w_1 = \sqrt{N_1/(N_1 + N_2)}$ and $w_2 = \sqrt{N_2/(N_1 + N_2)}$ be the a priori chosen weights associated with Stage 1 and Stage 2, respectively.

If both F and S are selected as the co-primary target populations, the following quantities are computed:

- $\mathbf{p_2^{\{S\}}}$ and $\mathbf{p_2^{\{F\}}}$ are defined and derived similarly as $\mathbf{p_1^{\{S\}}}$ and $\mathbf{p_2^{\{F\}}}$, respectively, with derivation based solely on patients enrolled in Stage 2.

"Use of this document is governed by the terms of use on the first page of this document."

- $\mathbf{p_2^{\{F,S\}}} = \min\left\{2\min\left(p_2^{\{S\}}, p_2^{\{F\}}\right), \max\left(p_2^{\{S\}}, p_2^{\{F\}}\right)\right\}$, p-value based on Simes' method for the intersection hypothesis $H_0^{\{F,S\}}$ based only on patients enrolled in Stage 2.
- $\mathbf{p_{12}^{\{S\}}} = 1 - \Phi\left\{w_1 \cdot \Phi^{-1}\left(1 - p_1^{\{S\}}\right) + w_2 \cdot \Phi^{-1}\left(1 - p_2^{\{S\}}\right)\right\}$, the combined p-value for $H_0^{\{S\}}$ from stage 1 and 2 following the weighted inverse normal combination method, where Φ denotes the cumulative distribution function of a standard normal variate.
- $\mathbf{p_{12}^{\{F\}}} = 1 - \Phi\left\{w_1 \cdot \Phi^{-1}\left(1 - p_1^{\{F\}}\right) + w_2 \cdot \Phi^{-1}\left(1 - p_2^{\{F\}}\right)\right\}$, the combined p-value for $H_0^{\{F\}}$ from Stage 1 and 2 following the weighted inverse normal combination method.
- $\mathbf{p_{12}^{\{F,S\}}} = 1 - \Phi\left\{w_1 \cdot \Phi^{-1}\left(1 - p_1^{\{F,S\}}\right) + w_2 \cdot \Phi^{-1}\left(1 - p_2^{\{F,S\}}\right)\right\}$, the combined p-value for $H_0^{\{F,S\}}$ from Stage 1 and 2 following the weighted inverse normal combination method.

If either F or S, but not both of them, is selected as the only target population, there remains only one elementary null hypothesis, which is also the intersection hypothesis. In general, let Q be the selected target population, that is, Q being either S or F the following quantities are computed:

- $\mathbf{p_2^{\{Q\}}}$ is derived as above and $\mathbf{p_2^{\{F,S\}}} = p_2^{\{Q\}}$.
- $\mathbf{p_{12}^{\{Q\}}}$ and $\mathbf{p_{12}^{\{F,S\}}}$ are then computed based on $(p_1^{\{Q\}}, p_2^{\{Q\}})$ and $(p_1^{\{F,S\}}, p_2^{\{F,S\}})$, respectively, as mentioned above. Please note that $p_1^{\{F,S\}}$ is the same as when both F and S are selected as the target populations. In general $p_1^{\{F,S\}}$ is invariant to selection of the target population(s) for the primary efficacy analysis for pCR.

In all cases, at the primary efficacy analysis for pCR, the null hypothesis associated with a selected target population (Q) is rejected if both $p_{12}^{\{Q\}} \leq \alpha_2$ and $p_{12}^{\{F,S\}} \leq \alpha_2$ following Simes' procedure.

N_2 was fixed at 120 to achieve optimal statistical operating characteristics across several simulation scenarios as provided in Appendix 15.

A summary table that presents the number and proportion of pCR in each treatment arm, together with the 2-sided 95% CIs with use of the Clopper–Pearson method (Clopper and Pearson 1934) will be produced by stage and overall for both the ITT and PD-L1–positive populations. Confidence intervals for the difference in pCR rate between the two arms will be determined using the normal approximation to the binomial distribution.

"Use of this document is governed by the terms of use on the first page of this document."

Appendix 15
Statistical Performance of the Adaptive Design

The performance of the proposed design depends on several factors, including the underlying proportion of (pCR) responders in each treatment arms for the Subgroup S (PD-L1 IC1/2/3) and the complement Group C (PD-L1 IC0). Simulations were carried out to evaluate the operation characteristics of the current design under a variety of conditions as follows.

Three simulation scenarios were considered with numerical details provided in Table 1 and results in Tables 2, 3, and 4.

- **Subgroup S with very strong credentials**

  Subgroup S has very strong credentials when convincing evidence indicates that the benefits of the treatment are limited to the Subgroup S.

- **Subgroup S with strong credentials**

  Subgroup S has strong credentials when evidence for the predictive ability of the subgroup is convincing enough to assume that the treatment is more likely to be effective (and is probably more effective) in the Subgroup S than in the complement Subgroup C, but the evidence is not sufficiently compelling to rule out a clinically meaningful effect in complement Subgroup C.

- **Subgroup S with weak credentials**

  If the Subgroup S has weak credentials when convincing evidence for predictive value of the Subgroup S is lacking and the treatment is expected to be broadly effective.

Table 1 Definition of Weak, Strong, and Very Strong Credentials on Subgroup S

Subgroup S Credentials	pCR proportion under treatment in Full population F (π_F)[a]	pCR Proportion under treatment in Subgroup S (π_S)	pCR Proportion under treatment in complement Subgroup C (π_C)	pCR Proportion response under Control ($\pi_{control}$)
Weak	0.68 (Δ_F=0.2)	0.68 (Δ_S=0.20)	0.68 (Δ_C=0.20)	0.48
Strong	0.6376 (Δ_F=0.1576)	0.68 (Δ_S=0.20)	0.60 (Δ_C=0.12)	0.48
Very Strong	0.5952 (Δ_F=0.1152)	0.68 (Δ_S=0.20)	0.52 (Δ_C=0.04)	0.48

[a] Assuming a prevalence of 0.47 of Subgroup S in the full population.

"Use of this document is governed by the terms of use on the first page of this document."

Table 2 Relative Frequencies of Decisions at Stage 1 under Weak, Strong, and Very Strong Credentials on Subgroup S

Subgroup S Credentials	Δ_S	Δ_C	Relative Frequency[a,b,c]				
			Stop for efficacy	Stop for lack of efficacy	Continue only S	Continue only F	Continue S and F
Weak	0.20	0.20	0.64	0.04	0.08	0.14	0.10
Strong	0.20	0.12	0.46	0.10	0.22	0.11	0.11
Very Strong	0.20	0.04	0.33	0.17	0.38	0.05	0.06

[a] Assuming a prevalence of 0.47 of Subgroup S in the full population.
[b] Assuming probability of drop-out of 0.05 for each treatment arm.
[c] Frequency based on 100000 simulations.

Table 3 Rejection Probabilities under Weak, Strong, and Very Strong Credentials on Subgroup S

Subgroup S Credentials	Δ_S	Δ_C	Probability[a,b,c]		
			Reject $H_0^{\{F\}}$	Reject $H_0^{\{S\}}$	Reject $H_0^{\{F\}}$ or $H_0^{\{S\}}$ i.e., Overall Power
Weak	0.20	0.20	0.80	0.49	0.88
Strong	0.20	0.12	0.55	0.57	0.76
Very Strong	0.20	0.04	0.28	0.62	0.67

[a] Assuming a prevalence of 0.47 of Subgroup S in the full population.
[b] Assuming probability of drop-out of 0.05 for each treatment arm.
[c] Probability based on 100000 simulations.

"Use of this document is governed by the terms of use on the first page of this document."

Table 4 Conditional Rejection Probabilities if Stage 2 Activated under Weak, Strong, and Very Strong Credentials on Subgroup S

Subgroup S Credentials	Δ_S	Δ_C	Conditional Probability[a,b,c,d] (if Stage 2 activated)		
			Reject $H_0^{\{F\}}$	Reject $H_0^{\{S\}}$	Reject $H_0^{\{F\}}$ or $H_0^{\{S\}}$ i.e., Overall "Go" Power
Weak	0.20	0.20	0.54	0.36	0.74
Strong	0.20	0.12	0.29	0.51	0.68
Very Strong	0.20	0.04	0.10	0.63	0.68

[a] Assuming a prevalence of 0.47 of Subgroup S in the full population.

[b] Assuming probability of drop-out of 0.05 for each treatment arm.

[c] Probability based on 100000 simulations.

[d] Conditional probability of event happening once the trial continues to enroll patients in Stage 2 ("go").

"Use of this document is governed by the terms of use on the first page of this document."

STATISTICAL ANALYSIS PLAN

TITLE: **A PHASE III RANDOMIZED STUDY TO INVESTIGATE THE EFFICACY AND SAFETY OF ATEZOLIZUMAB (ANTI–PD-L1 ANTIBODY) IN COMBINATION WITH NEOADJUVANT ANTHRACYCLINE /NAB-PACLITAXEL-BASED CHEMOTHERAPY COMPARED WITH PLACEBO AND CHEMOTHERAPY IN PATIENTS WITH PRIMARY INVASIVE TRIPLE-NEGATIVE BREAST CANCER**

PROTOCOL NUMBER: WO39392

STUDY DRUG: Atezolizumab (MPDL3280A; RO5541267)

VERSION NUMBER: 2

IND NUMBER: 123277

EUDRACT NUMBER: 2016-004734-22

SPONSOR: F. Hoffmann-La Roche Ltd

PLAN PREPARED BY: [redacted], Ph.D.

DATE FINAL: Version 1: 6 December 2017

DATE AMENDED: Version 2: See electronic date stamp below

STATISTICAL ANALYSIS PLAN AMENDMENT APPROVAL

Name	Reason for Signing	Date and Time (UTC)
[redacted]	Company Signatory	06-Nov-2018 13:15:32

CONFIDENTIAL

This is a F. Hoffmann-La Roche Ltd document that contains confidential information. Nothing herein is to be disclosed without written consent from F. Hoffmann-La Roche Ltd.

"Use of this document is governed by the terms of use on the first page of this document."

STATISTICAL ANALYSIS PLAN AMENDMENT RATIONALE

This Statistical Analysis Plan (SAP) Version 2 for Study WO39392 (IMpassion031) has been amended to incorporate an adaptive, two-stage design that uses accumulating data to modify aspects of the study as it continues, without undermining the validity and integrity of the trial.

> Study WO39392 is currently designed (protocol version 4) to randomize approximately 204 patients in total (approximately 102 patients per arm) to control the type I error for the primary endpoint of pathological complete response (pCR) at the 5% level of significance (two-sided) in the intent-to-treat (ITT) population. However, we have observed strong evidence of difference in efficacy effect across the programmed death–ligand 1 (PD-L1) subgroups of atezolizumab plus nab-paclitaxel arm in metastatic triple negative breast cancer (TNBC) study (WO29522) that could impact the WO39392 study. To address the uncertainty surrounding design choices based on the effect of PD-L1 and to allow review of accumulating information during the ongoing WO39392 study, we plan to change the current fixed design to an adaptive design. The proposed adaptive design consists of two trial stages. After an interim analysis at the end of stage 1 (i.e., approximately 204 patients), recommendations will be made to either continue the study unchanged or to expand the target population for a subsequent stage 2 of the trial (i.e., approximately 120 additional patients).
>
> To maintain the integrity of the trial, an independent Data Coordinating Center will perform the interim analysis and an independent Data Monitoring Committee (iDMC) will evaluate the interim analysis results. The iDMC will provide a recommendation as to whether to continue the study unchanged or expand to stage 2. The decision rule to be applied at the interim analysis will be clearly expressed to the iDMC and documented in the iDMC charter so that the study can be conducted with the Sponsor remaining completely blinded to all results unless iDMC recommendation is not to expand into stage 2. The iDMC will also monitor safety and study conduct for stage 2 of Study WO39392.

To reflect these changes, this SAP has been amended alongside the study protocol. Additional minor changes have been made to improve clarity and consistency.

"Use of this document is governed by the terms of use on the first page of this document."

TABLE OF CONTENTS

1. BACKGROUND 5

2. STUDY DESIGN 5

2.1 Protocol Synopsis 5

2.2 Outcome Measures 5

2.2.1 Primary Efficacy Outcomes 5

2.2.2 Secondary Efficacy Outcomes 6

2.2.3 Safety Outcomes 6

2.2.4 Pharmacokinetic Outcomes 6

2.2.5 Immunogenicity Outcomes 6

2.3 Determination of Sample Size 6

3. STUDY CONDUCT 7

3.1 Randomization 7

3.2 Data Monitoring 8

4. STATISTICAL METHODS 8

4.1 Definition of Analysis Populations 8

4.2 Analyses of Study Conduct 8

4.3 Analyses of Treatment Group Comparability 9

4.4 Analyses of Efficacy 9

4.4.1 Primary Efficacy Endpoint 9

4.4.1.1 Primary Analyses 9

4.4.2 Secondary Efficacy Endpoints 12

4.4.2.1 Event-Free Survival 12

4.4.2.2 Disease-Free Survival 12

4.4.2.3 Overall Survival 13

4.4.2.4 Patient-Reported Outcomes of Role and Physical Function and Global Health Status/ Health-Related Quality of Life–EORTC Data 13

4.4.3 Exploratory Efficacy Analyses 14

4.4.3.1 Patient-Reported Outcomes of Disease/Treatment Symptoms, Emotional and Social Function – EORTC Data 14

"Use of this document is governed by the terms of use on the first page of this document."

4.4.3.2 Patient-Reported Outcome of Treatment Bother – FACT-G, GP5 Data 14
4.4.3.3 Health Economic EQ-5D-5L Data 14
4.4.3.4 Proportion of Patients Undergoing Breast-Conserving Surgery 14
4.4.3.5 Residual Cancer Burden Index 14
4.5 Analyses of Safety 15
4.5.1 Exposure of Study Medication 15
4.5.2 Adverse Events 15
4.5.3 Laboratory Data 16
4.5.4 Vital Signs 16
4.6 Pharmacokinetic Analyses 16
4.7 Immunogenicity Analyses 16
4.8 Biomarker Analyses 17
4.9 Exploratory Analyses 17
4.9.1 Exploratory Biomarker Analyses 17
4.9.2 Subgroup Analyses 17
4.10 Interim Analyses 18
4.10.1 Planned Interim Analysis 18
4.10.2 Optional Interim Analysis 19

5. REFERENCES 20

LIST OF FIGURES

Figure 1 Flowchart of the Decision Rules 19

LIST OF APPENDICES

Appendix 1 Protocol Synopsis 21
Appendix 2 Schedule of Activities (for Both Stage 1 and Stage 2) 32
Appendix 3 Adaptive Multiple Test Design 39

"Use of this document is governed by the terms of use on the first page of this document."

1. BACKGROUND

This Statistical Analysis Plan (SAP) describes the analyses that are planned to be performed for Study WO39392 (also known as IMpassion031).

2. STUDY DESIGN

2.1 PROTOCOL SYNOPSIS

Study WO39392 (IMpassion031) is a global Phase III, double-blind, randomized, placebo-controlled study designed to evaluate the efficacy and safety of neoadjuvant treatment with TECENTRIQ®(atezolizumab) and nab-paclitaxel followed by doxorubicin and cyclophosphamide with atezolizumab compared with neoadjuvant treatment with placebo and nab-paclitaxel followed by doxorubicin and cyclophosphamide with placebo in patients with primary invasive early triple-negative breast cancer (TNBC), that are eligible for surgery. The study will enroll patients with initial clinically assessed T2-4d TNBC.

The study design consists of two trial stages, 1 and 2, with an a priori fixed sample size for both stages. In stage 1, approximately 204 patients will be randomized in a 1:1 ratio to the atezolizumab arm or the placebo arm as described in Section 3.1.

After an interim analysis conducted at the completion of stage 1 (i.e., once all patients enrolled in stage 1 have had surgery and pCR assessment), a decision will be made to either continue the study unchanged or to expand patient enrolment for a subsequent stage 2 of the trial (i.e., approximately 120 additional patients). If after the first stage, the trial is not read out for efficacy or lack of efficacy, the sponsor will proceed into the second stage with the selected population. Approximately 120 additional patients will be randomized in a 1:1 ratio to the atezolizumab arm or the placebo arm. At the conclusion of stage 2, the primary analysis of pCR is conducted by combining the p-values of both stage 1 and 2 into a single test statistic using a predefined combination function.

Note that the primary analysis of pCR could occur at the end of stage 1 (early stop for efficacy) projected to take place approximately 21 months after the first patient has been randomized or at the end of stage 2 projected to take place approximately 35–44 months after the first patient has been randomized depending on the targeted population selected at the end of stage 1.

The protocol synopsis for Study WO39392 including a description of the study design is in Appendix 1.

2.2 OUTCOME MEASURES

2.2.1 Primary Efficacy Outcomes

The co-primary efficacy endpoint for this study is pCR defined as eradication of invasive tumor from both breast and lymph nodes (ypT0/is ypN0) in the intent-to-treat (ITT)

"Use of this document is governed by the terms of use on the first page of this document."

population (full population) and in the subpopulation with programmed death-ligand 1 (PD-L1)–positive tumor status (tumor-infiltrating immune cell [IC] IC1/2/3) (see Section 4.1). Patients whose pCR assessment was missing will be counted as not achieving a pCR.

2.2.2 Secondary Efficacy Outcomes

- Event-free survival (EFS) defined as the time from randomization until documented disease recurrence, progression, or death from any cause in all patients and in the subpopulation with PD-L1–positive tumor status. More details for EFS definition are given in Section 4.4.2.1.
- Disease-free survival (DFS) defined as the time from surgery until documented disease recurrence or death from any cause in all patients (ITT population) who undergo surgery and in the subpopulation of patients with PD-L1–positive tumor status who undergo surgery. More details for DFS definition are given in Section 4.4.2.1.
- Overall survival (OS) defined as the time from randomization to the date of death from any cause in all patients and in the subpopulation with PD-L1–positive tumor status.
- Mean and mean changes from baseline score in function (role, physical) and global health status (GHS)/ health-related quality of life (HRQoL) by cycle and between treatment arms as assessed by the functional and HRQoL scales of the European Organisation for Research and Treatment of Cancer (EORTC) Quality of Life Questionnaire Core 30 (QLQ C30).

2.2.3 Safety Outcomes

- Incidence, nature, and severity of adverse events graded according to National Cancer Institute Common Terminology Criteria in Adverse Events, Version 4.0 (NCI CTCAE v4.0).
- Changes in vital signs and clinical laboratory results.

2.2.4 Pharmacokinetic Outcomes

Serum concentration of atezolizumab at specified timepoints (see Appendix 2).

2.2.5 Immunogenicity Outcomes

Incidence of anti-drug antibodies (ADAs) during the study and the prevalence of ADAs at baseline.

2.3 DETERMINATION OF SAMPLE SIZE

The study will randomize approximately 204 patients in stage 1 (approximately 102 patients per arm) and approximately 120 patients in stage 2 (approximately 60 patients per arm).

The performance of the study design depends on several factors including the underlying proportion of (pCR) responders in each treatment arms for the subgroup S

"Use of this document is governed by the terms of use on the first page of this document."

(PD-L1 IC1/2/3) and the complement group C (PD-L1 IC0). Simulations were carried out to evaluate the operation characteristics of the current design under a variety of conditions including the overall power defined as the probability to reject any null hypothesis at any analysis time point in the study, which is observed to be at least 68% across the simulation scenarios under investigation. More details are provided in Appendix 1.

3. STUDY CONDUCT

3.1 RANDOMIZATION

Randomization will occur in a 1:1 ratio using a permuted-block randomization method. Patients will be randomized to one of two treatment arms:

- **Arm A**: atezolizumab (840 mg fixed dose) administered via intravenous (IV) infusion every two weeks (q2w) in combination with nab-pac (125 mg/m^2) administered via IV infusion every week (qw) for 12 weeks followed by atezolizumab (840 mg fixed dose) administered q2w in combination with doxorubicin (60 mg/m^2)+cyclophosphamide (600 mg/m^2) administered q2w via IV infusions with filgrastim/ pegfilgrastim support for 4 cycles. Patients randomized to the atezolizumab arm will continue to receive unblinded atezolizumab post-surgery at a fixed dose of 1200 mg by IV infusion every 3 weeks (q3w) for 11 cycles, for a total of approximately 12 months of atezolizumab therapy.
- **Arm B**: placebo administered q2w via IV infusion in combination with nab-paclitaxel (125 mg/m^2) administered qw via IV infusion for 12 weeks followed by placebo administered q2w in combination with doxorubicin (60 mg/m^2)+cyclophosphamide (600 mg/m^2) administered q2w via IV infusions with filgrastim/ pegfilgrastim support for 4 cycles. Patients randomized to the placebo arm will be unblinded post-surgery and will continue to be followed up as per the schedule of activities outlined in the study protocol.

Postoperative patient management for those in either treatment arm may include radiotherapy as clinically indicated, and management of patients who do not achieve a pCR should follow current standard-of-care guidelines. For patients in Arm A, chemotherapy and/or radiotherapy may be administered concurrently with atezolizumab after discussion with Medical Monitor.

The randomization scheme is designed to ensure that an approximately equal number of patients will be enrolled in each treatment arm within the categories defined for the following stratification factors at baseline:

- American Joint Committee on Cancer (AJCC) Stage at diagnosis (II vs. III) for patients enrolled in both stages 1 and 2.
- Tumor infiltrating immune cells PD-L1 status (IC0 vs. IC1/2/3) for patients enrolled in stage 1 as well as stage 2 IF the study continues to enroll all-comer patients in stage 2.

"Use of this document is governed by the terms of use on the first page of this document."

3.2 DATA MONITORING

An iDMC will evaluate the primary efficacy endpoint of pCR in the ITT and PD-L1−positive populations at the prespecified interim analysis based on data of approximately 204 patients enrolled in stage 1 and will make a recommendation to either complete the study unchanged or to expand the target population for the subsequent stage 2 of the trial (i.e., approximately 120 additional patients). The decision rules to be applied at the interim analysis will be clearly expressed to the iDMC and documented in the iDMC charter so that the study can be conducted with the Sponsor remaining completely blinded to all results at this stage.

The iDMC will also evaluate safety data and study conduct on a regular basis during the study until the primary analysis of pCR, which is performed at stage 1 (approximately 204 patients) or stage 2 (approximately 324 patients), if the iDMC recommends extending the target population after which, iDMC review of the study data will be discontinued.

Sponsor affiliates will be excluded from iDMC membership. All summaries and analyses for the iDMC review will be prepared by an independent Data Coordinating Center. The iDMC will follow a charter that outlines the iDMC's roles and responsibilities.

4. STATISTICAL METHODS

The analyses outlined in this SAP supersede those specified in the protocol for regulatory filing purposes.

4.1 DEFINITION OF ANALYSIS POPULATIONS

The primary analysis population for efficacy is the ITT population (full population), defined as all randomized patients, and the PD-L1−positive subpopulation, defined as patients in the ITT population whose PD-L1 status is IC1/2/3 at the time of randomization. Patients will be assigned to the treatment group to which they were randomized.

The primary analysis population for safety is the safety evaluable population defined as all patients who received at least one dose of study medication. Patients will be assigned to treatment groups as treated, and all patients who received any dose of atezolizumab will be included in the atezolizumab treatment arm.

4.2 ANALYSES OF STUDY CONDUCT

For all randomized patients (i.e., ITT population), a participant flowchart for depicting the progress of subjects through the phases of the trial will be provided by treatment arm and study stage (i.e., 1 and 2) of enrollment, including a complete description of patient disposition specifying the number of randomized patients and completed and discontinued patients from trial treatment and study with reasons for premature discontinuation. Documented major protocol deviations including those related to study

"Use of this document is governed by the terms of use on the first page of this document."

inclusion/ exclusion criteria, conduct of the study, patient management, or patient assessment will be also tabulated by treatment arm and study stage of enrollment.

4.3 ANALYSES OF TREATMENT GROUP COMPARABILITY

Demographic variables such as age, sex, race/ethnicity, and baseline characteristics (in particular, stratification variables) will be summarized by treatment arm and study stage (i.e., 1 and 2) of enrollment for the ITT as well as PD-L1−positive population. Only descriptive analyses are planned; no formal statistical tests will be applied. Continuous variables will be reported and summarized by use of standard measures of central tendency and dispersion (mean, SD, median, and range including minimum and maximum), and categorical (i.e., discrete) data will be reported and summarized by frequencies and percentages.

The baseline value of any variable will be defined as the last available value prior to the first administration of study treatment.

4.4 ANALYSES OF EFFICACY

4.4.1 Primary Efficacy Endpoint

4.4.1.1 Primary Analyses

The ITT as well as PD-L1−positive population will be used for the primary analysis of the co-primary endpoints of pCR in these populations as follows. The **overall one-sided type I error** for testing the co-primary endpoints of pCR is $\boldsymbol{\alpha = 0.025}$.

Two main sources for multiplicity in statistical testing from the current design are:

- Multiple target populations, namely ITT and PD-L1−positive populations.
- Study having 2 stages with target population at stage 2 depending on results from stage 1.

Simes' closed testing procedure will be used to address multiplicity in target populations and weighted inverse p-value combination method is applied to account for the adaptive choice of target population at stage 2 as follows.

At Stage 1

For the interim analysis at end of stage 1, the co-primary endpoints of pCR in the ITT and PD-L1−positive population will be tested at the pre-defined one-sided type I error of 0.0125 (i.e. 50% of the total type I error) following Simes' procedure. This involves the following p-values:

- $\boldsymbol{p_1^{\{F\}}}$ is the p-value from the **one-sided** Cochran-Mantel-Haenszel (CMH) χ^2 test for difference in pCR rates between treatment groups in the **ITT population** (Δ_F) stratified according to tumor PD-L1 status (IC0 vs. IC1/2/3) and clinical stage at presentation (Stage II vs. III). The associated elementary null hypothesis is denoted as $\mathrm{H}_0^{\{\mathrm{F}\}}$:$\Delta_F \leq 0$.

"Use of this document is governed by the terms of use on the first page of this document."

- $\boldsymbol{p_1^{\{S\}}}$ is the p-value from the CMH χ^2 test for difference in pCR rates between treatment groups in the **PD-L1–positive population** (Δ_S) stratified according to clinical stage at presentation (Stage II vs. III). The associated elementary null hypothesis is denoted as $\mathrm{H}_0^{\{S\}}$:$\Delta_S \leq 0$.
- $\boldsymbol{p_1^{\{F,S\}}} = min\left\{2min\left(p_1^{\{S\}}, p_1^{\{F\}}\right), max\left(p_1^{\{S\}}, p_1^{\{F\}}\right)\right\}$, p-value based on Simes' method for the intersection hypothesis $H_0^{\{F,S\}}$, which specifies that there is no proportion difference in either F or S.

In addition, the observed difference in pCR rates between arms in S, denoted as $\widehat{\Delta}_1^S$ and its complementary sub-population of patients with PD-L1 IC0 (C), denoted as $\widehat{\Delta}_1^C$ will also be computed.

Based on $p_1^{\{F\}}$, $p_1^{\{S\}}$, $p_1^{\{F,S\}}$, $\widehat{\Delta}_1^S$ and $\widehat{\Delta}_1^C$, the following decision could be made at stage 1:

1. If $p_1^{\{F,S\}} \leq \alpha_1$, the analysis at stage 1 is considered the primary efficacy analysis for pCR with evidence for efficacy declared in either F (if $p_1^{\{F\}} \leq \alpha_1$) or S (if $p_1^{\{S\}} \leq \alpha_1$) or both, the study will therefore not enroll extra patients in stage 2.
2. Otherwise, if both $\widehat{\Delta}_1^S$ and $\widehat{\Delta}_1^C$ are below the pre-specified thresholds, $d_S = 0.12$ and $d_C = 0.1$, respectively, the study will also not enroll extra patients in stage 2 for lack of efficacy.
3. Otherwise, if $\widehat{\Delta}_1^S \geq d_S$ and $\widehat{\Delta}_1^C < d_C$, the study will enroll only patients in S at stage 2, with S being the only target population in the primary efficacy analysis for pCR.
4. Otherwise, if $\widehat{\Delta}_1^S < d_S$ and $\widehat{\Delta}_1^C \geq d_C$, the study will enroll all-comer patients (F) in stage 2; however, only F will be the target population in the primary efficacy analysis for pCR.
5. If $\hat{\Delta}_1^S \geq d_S$ and $\hat{\Delta}_1^C \geq d_C$, the study will enroll all-comer patients in stage 2, and the co-primary efficacy endpoints of pCR in F and S will be considered in the primary efficacy analysis for pCR.

At Stage 2 (if needed)

If the study advances to stage 2, at the primary efficacy analysis for pCR to protect the overall α the corresponding boundary for statistical significance is α_2=0.0184, accounting for the fact that and overall $\alpha_1 = 0.0125$ having been spent at stage 1 and the information fraction at stage 1, namely $w_1 = N_1/(N_1 + N_2)$. Indeed, the p-value p_1 based on stage 1 data only and the combination p-value p_2 have the same joint distribution under the null hypothesis as the p-values from a group-sequential test with two stages at information times $t_1 = w_1$ and $t_2 = 1$. Thus standard statistical software for group sequential designs can be used for the determination of critical values for the adaptive design (Wassmer and Brannath 2016, Section 6.2.4).

"Use of this document is governed by the terms of use on the first page of this document."

Final test(s) involving the target population(s) selected at stage 1 will be based on weighted inverse normal p-value combination method (Wassmer and Brannath 2016) as follows:

Let $w_1 = \sqrt{N_1/(N_1 + N_2)}$ and $w_2 = \sqrt{N_2/(N_1 + N_2)}$ be the a priori chosen weights associated with stage 1 and stage 2, respectively.

If both F and S are selected as the co-primary target populations, the following quantities are computed:

- $\boldsymbol{p}_{\boldsymbol{2}}^{\{S\}}$ and $\boldsymbol{p}_{\boldsymbol{2}}^{\{F\}}$ are defined and derived similarly as $\boldsymbol{p}_{\boldsymbol{1}}^{\{S\}}$ and $\boldsymbol{p}_{\boldsymbol{2}}^{\{F\}}$, respectively, with derivation based solely on patients enrolled in stage 2.
- $\boldsymbol{p}_{\boldsymbol{2}}^{\{F,S\}} = min\left\{2min\left(p_2^{\{S\}}, p_2^{\{F\}}\right), max\left(p_2^{\{S\}}, p_2^{\{F\}}\right)\right\}$, p-value based on Simes' method for the intersection hypothesis $H_0^{\{F,S\}}$ based only on patients enrolled in stage 2.
- $\boldsymbol{p}_{\boldsymbol{12}}^{\{S\}} = 1 - \Phi\left\{w_1 \cdot \Phi^{-1}\left(1 - p_1^{\{S\}}\right) + w_2 \cdot \Phi^{-1}\left(1 - p_2^{\{S\}}\right)\right\}$, the combined p-value for $\mathrm{H}_0^{\{\mathrm{S}\}}$ from stage 1 and 2 following the weighted inverse normal combination method, where Φ denotes the cumulative distribution function of a standard normal variate.
- $\boldsymbol{p}_{\boldsymbol{12}}^{\{F\}} = 1 - \Phi\left\{w_1 \cdot \Phi^{-1}\left(1 - p_1^{\{F\}}\right) + w_2 \cdot \Phi^{-1}\left(1 - p_2^{\{F\}}\right)\right\}$, the combined p-value for $\mathrm{H}_0^{\{\mathrm{F}\}}$ from stage 1 and 2 following the weighted inverse normal combination method.
- $\boldsymbol{p}_{\boldsymbol{12}}^{\{F,S\}} = 1 - \Phi\left\{w_1 \cdot \Phi^{-1}\left(1 - p_1^{\{F,S\}}\right) + w_2 \cdot \Phi^{-1}\left(1 - p_2^{\{F,S\}}\right)\right\}$, the combined p-value for $\mathrm{H}_0^{\{\mathrm{F,S}\}}$ from stage 1 and 2 following the weighted inverse normal combination method.

If either F or S, but not both of them, is selected as the only target population, there remains only one elementary null hypothesis, which is also the intersection hypothesis. In general, let Q be the selected target population, that is, Q being either S or F the following quantities are computed:

- $\boldsymbol{p}_{\boldsymbol{2}}^{\{Q\}}$ is derived as above and $\boldsymbol{p}_{\boldsymbol{2}}^{\{F,S\}} = p_2^{\{Q\}}$.
- $\boldsymbol{p}_{\boldsymbol{12}}^{\{Q\}}$ and $\boldsymbol{p}_{\boldsymbol{12}}^{\{F,S\}}$ are then computed based on $(p_1^{\{Q\}}, p_2^{\{Q\}})$ and $(p_1^{\{F,S\}}, p_2^{\{F,S\}})$, respectively, as mentioned above. Please note that $p_1^{\{F,S\}}$ is the same as when both F and S are selected as the target populations. In general $p_1^{\{F,S\}}$ is invariant to selection of the target population(s) for the primary efficacy analysis for pCR.

In all cases, at the primary efficacy analysis for pCR, the null hypothesis associated with a selected target population (Q) is rejected if both $p_{12}^{\{Q\}} \leq \alpha_2$ and $p_{12}^{\{F,S\}} \leq \alpha_2$ following Simes' procedure.

Operating characteristics of the current design across several simulation scenarios are provided in Appendix 1.

"Use of this document is governed by the terms of use on the first page of this document."

A summary table that presents the number and proportion of pCR in each treatment arm, together with the 2-sided 95% CIs with use of the Clopper–Pearson method (Clopper and Pearson 1934) will be produced by stage and overall for both the ITT and PD-L1–positive populations. Confidence intervals for the difference in pCR rate between the two arms will be determined using the normal approximation to the binomial distribution.

4.4.2 Secondary Efficacy Endpoints

4.4.2.1 Event-Free Survival

Event-free survival (EFS) is defined as the time from randomization to the first documented occurrence of disease recurrence, disease progression, or death from any cause. EFS events covered under “disease recurrence” will include local, regional, or distant recurrence and contralateral breast cancer. Ipsilateral or contralateral in situ disease and second primary non-breast cancers will not be counted as EFS events, even though they will be recorded in the electronic Case Report Form accordingly.

Patients without an event at the time of the analysis will be censored on the date on which they are last known to be alive and event free, on or before the clinical data cutoff date for the respective analysis. Patients with no post-baseline information will be censored at the date of randomization.

EFS will be compared between treatment arms with use of the stratified log-rank test. The hazard ratio (HR) for EFS will be estimated using a stratified Cox proportional hazards model. The 95% CI for the HR will be provided. Kaplan–Meier methodology will be used to estimate the median EFS (if reached) for each treatment arm, and Kaplan-Meier curves will be produced. The Brookmeyer–Crowley methodology will be used to construct the 95% CI for the median EFS for each treatment arm (Brookmeyer and Crowley 1982). The Kaplan–Meier approach will be used to estimate 3-year EFS rates and corresponding 95% CIs for each treatment arm.

The stratification factors for all analyses based on the ITT population will be: tumor stage (AJCC) at baseline (Stage II vs. III) and PD-L1 status (IC0 vs. IC1/2/3).

The stratification factors for EFS analysis in the PD-L1–positive subpopulation will be AJCC at baseline (Stage II vs. III).

Results from unstratified analyses will also be provided.

4.4.2.2 Disease-Free Survival

DFS is defined as the time from surgery (i.e., the first date of no disease) to the first documented disease recurrence or death from any cause, whichever occurs first. DFS will be analyzed with the use of the same methodology as specified for EFS for both the ITT population and the PD-L1 positive subpopulation.

"Use of this document is governed by the terms of use on the first page of this document."

The DFS analysis will be performed approximately 36 months after the randomization of the last patient.

Patients who do not undergo surgery at the end of neoadjuvant treatment will be excluded from the analysis of DFS. Patients without a DFS event at the time of analysis will be censored at the date when they were last known to be alive and event free. Patients who do not have information after surgery will be censored at the date of surgery.

4.4.2.3 Overall Survival

Overall Survival (OS) is defined as the time from the date of randomization to the date of death due to any cause. Patients who are not reported as having died at the time of analyses will be censored at the date when they were last known to be alive. Patients who do not have information after baseline will be censored at the date of randomization. OS will be analyzed with the use of the same methodology as specified for EFS for both the ITT population and the PD-L1−positive subpopulation.

4.4.2.4 Patient-Reported Outcomes of Role and Physical Function and Global Health Status/ Health-Related Quality of Life–EORTC Data

The EORTC QLQ-C30 (version 3) data will be scored according to the EORTC scoring manual (Fayers et al. 2001). Missing data will be assessed and reported by cycle. In the event of incomplete data, if the scale has more than 50% of the constituent items completed, a pro-rated score will be computed consistent with the scoring manual and validation papers of the measure. For subscales with less than 50% of the items completed, the subscale will be considered missing. Patient-reported outcomes (PRO) completion, compliance rates, and reasons for missing data will be summarized at each timepoint by treatment arm.

The primary patient-reported endpoints are mean and mean changes from baseline score in function (role, physical) and GHS/ HRQoL. Summary statistics (mean, SD, median, and range) of linearly transformed absolute scores and mean changes from baseline will be calculated for the functional (role [Question {Q}6, Q7], physical [Q1−Q5]) and the GHS/ HRQoL (Q29, Q30) scales of the EORTC QLQ-C30 at each assessment time point for each arm. The mean change from baseline (and 95% CI) will be assessed on patients with at least one post-baseline measurement. Previously published meaningful thresholds of change important differences will be used to identify clinically meaningful change from baseline within each treatment group on the functional and GHS/ HRQoL scales (Osoba et al. 1998, Cocks et al. 2011).

Longitudinal analysis will be conducted to estimate the effect difference on PRO repeated responses over a selected period of time and between the treatment arms. Mixed effect models on a set of covariates (baseline domain score, patient demographic, and clinical variables) will be conducted. Change from baseline at subsequent cycles

"Use of this document is governed by the terms of use on the first page of this document."

will be presented by treatment arm and will include least squares mean (LS Mean), difference in LS Mean between two treatment arms, and 95% CIs for the differences. The standard error will also be calculated for each LS Mean.

4.4.3 Exploratory Efficacy Analyses

4.4.3.1 Patient-Reported Outcomes of Disease/Treatment Symptoms, Emotional and Social Function – EORTC Data

Summary statistics (mean, SD, median, and range) of linearly transformed absolute scores and mean changes from baseline will be calculated for all disease/treatment-related symptom items and scales, and the emotional, social function scales of the EORTC QLQ-C30 at each assessment timepoint for each arm. The analyses of this exploratory endpoint will be analogous to the secondary PRO endpoints (see Section 4.4.2.4 for further details).

4.4.3.2 Patient-Reported Outcome of Treatment Bother – FACT-G, GP5 Data

A descriptive analysis of absolute scores and the proportion of patients selecting each response option at each assessment time-point by treatment arm will be reported for item GP5 ("I am bothered by side effects of treatment") from the Functional Assessment of Cancer Therapy-General (FACT-G) physical well-being subscale. Item GP5 from version 4 of the FACT-G questionnaire will be scored according to the FACIT scoring manual (Cella 1997).

4.4.3.3 Health Economic EQ-5D-5L Data

Health utility data from the EuroQoL 5 Dimension, 5 Level (EQ-5D-5L), will be evaluated in pharmacoeconomic models. The results from the health economic data analyses will be reported separately from the clinical study report.

4.4.3.4 Proportion of Patients Undergoing Breast-Conserving Surgery

The proportion of patients undergoing breast-conserving surgery will be compared between the treatment arms using the same methodology for pCR analysis in Section 4.4.1.1, for the subpopulation of patients having surgery.

4.4.3.5 Residual Cancer Burden Index

Pathological review of the primary tumor site and sampled lymph nodes by the local pathologists at surgery will also include the calculation of the Residual Cancer Burden (RCB) index. Such index is computed by taking into account the primary tumor bed area, overall cancer cellularity, the percentage of in situ disease, number of positive lymph nodes and the diameter of the largest lymph node metastasis (Symmans et al. 2007). Pathological complete response would correspond to RCB=0, whereas minimal, moderate, and extensive residual disease would be indicated by RCB-I, RCB-II, and RCB-III, respectively.

The following analyses will be carried out for the population of patient with evaluable RCB index.

"Use of this document is governed by the terms of use on the first page of this document."

A summary table that presents the number and proportion of RCB-0, -I, -II and -III in each treatment arm, together with the respective 2-sided 95% CIs with use of the Clopper-Pearson method (Clopper and Pearson 1934) will be produced.

Comparison between treatment arms will be done using ordered logit (proportional-odds) regression model (Peter McCullagh 1980) stratified for the stratification factors as for the primary pCR analysis.

4.4.3.5.1 Association between RCB Index and EFS, OS

The analyses for EFS, DFS and OS, described in Section 4.4.2.1, Section 4.4.2.2, and Section 4.4.2.3 respectively, will be repeated for the subgroups of patients with RCB-0, RCB-I, RCB-II, and RCB-III.

4.5 ANALYSES OF SAFETY

Safety analyses will be performed on the safety population defined as all patients who received any dose of study medication.

Safety will be assessed through summaries of AEs, changes in laboratory test results, changes in vital signs, study treatment exposures, and immunogenicity as measured by ADAs and will be presented by treatment arm.

4.5.1 Exposure of Study Medication

Study drug exposure, including but not limited to treatment duration, number of cycles, and dose intensity, will be summarized with descriptive statistics for each study treatment on each treatment arm if deemed appropriate.

4.5.2 Adverse Events

Verbatim descriptions of AEs will be mapped to Medical Dictionary for Regulatory Activities (MedDRA) thesaurus terms and graded according to NCI CTCAE v4.0.

Treatment-emergent adverse events (TEAEs), defined as events occurring on or after the first dose of study treatment will be summarized by MedDRA term, appropriate MedDRA levels, and NCI CTCAE v4.0 grade, regardless of relationship to study drug as assessed by the investigator. For each patient, if multiple incidences of the same AEs occur, the maximum severity reported will be used in the summaries.

The following TEAEs will be summarized separately:

- AEs leading to withdrawal of study drug,
- AEs leading to dose reduction or interruption,
- Grade $\geq$ 3 AEs, Grade 5 AEs, serious adverse events (SAEs), and
- Adverse events of special interest (AESIs).

All deaths and causes of death will be summarized.

"Use of this document is governed by the terms of use on the first page of this document."

4.5.3 Laboratory Data

Laboratory data with values outside of the normal ranges will be identified. Relevant laboratory values will be summarized by treatment arm over time, with NCI CTCAE v4.0 Grade 3 and Grade 4 values identified, where appropriate. Changes from baseline in NCI CTCAE v4.0 grade (i.e., shift tables) will be also provided by treatment arm. Of note, abnormal laboratory data that are clinically significant will be reported as adverse events and summarized in the adverse event tables.

A Hy's law analysis will be provided.

The finding of an elevated ALT or AST (>3×**baseline value**) in combination with either an elevated total bilirubin (>2×ULN) **or clinical jaundice in the absence of cholestasis or other causes of hyperbilirubinemia** is considered to be an indicator of severe liver injury (as defined by Hy's Law). Therefore, investigators must report as an adverse event the occurrence of either of the following:

- Treatment-emergent ALT or AST >3 × ULN (or baseline value if baseline value was above the ULN) in combination with total bilirubin >2 × ULN (of which ≥35% is direct bilirubin).
- Treatment-emergent ALT or AST >3 × ULN (or baseline value if baseline value was above the ULN) in combination with clinical jaundice.

4.5.4 Vital Signs

Changes in selected vital signs will be summarized by treatment arm and by change over time including change from baseline.

4.6 PHARMACOKINETIC ANALYSES

Atezolizumab serum concentration data (C_{min} and C_{max}) will be tabulated and summarized. Descriptive statistics will include means, medians, ranges, and SDs, as appropriate.

Additional pharmacokinetic and pharmacodynamic analyses will be conducted as appropriate.

4.7 Immunogenicity Analyses

The immunogenicity analyses will include patients with at least one post-baseline ADA assessment, with patients grouped according to treatment received.

The numbers and proportions of ADA-positive patients and ADA-negative patients at baseline (baseline prevalence) and after baseline (post-baseline incidence) will be summarized by treatment group. When determining post-baseline incidence, patients are considered to be ADA-positive if they are ADA-negative or are missing data at baseline but develop an ADA response following study drug exposure (treatment induced ADA response), or if they are ADA-positive at baseline and the titer of one or

"Use of this document is governed by the terms of use on the first page of this document."

more post-baseline samples is at least 0.60 titer unit greater than the titer of the baseline sample (treatment-enhanced ADA response). Patients are considered to be ADA-negative if they are ADA-negative or are missing data at baseline and all post baseline samples are negative, or if they are ADA-positive at baseline but do not have any post-baseline samples with a titer that is at least 0.60 titer unit greater than the titer of the baseline sample (treatment unaffected).

The relationship between ADA status and safety, efficacy, and pharmacokinetics may be investigated.

4.8 BIOMARKER ANALYSES

EFS, DFS, and OS will be assessed in the PD-L1 IC1/2/3 population as secondary endpoints as described in Sections 2.2.2, 4.4.2.1, and 4.4.2.3 respectively.

4.9 EXPLORATORY ANALYSES

4.9.1 Exploratory Biomarker Analyses

Exploratory biomarker analyses will be performed in baseline pretreatment, on-treatment, residual-disease, and post recurrence samples in an effort to understand the association of these markers with study treatment outcome, including efficacy and/or adverse events. The biomarkers may include but will not be limited to PD-L1-expressing tumor cells, tumor infiltrating immune cells, RNA-based T-effector signature and other biomarkers in tumor and blood, as defined by immunohistochemistry, quantitative reverse transcription polymerase chain reaction, next-generation DNA and RNA sequencing, or other methods. Pharmacodynamic changes of these biomarkers at baseline, on-treatment samples and at surgical resection.

Circulating tumor DNA (ctDNA) will be evaluated in post-surgery plasma samples and compared between control and experimental arms.

Whole genome sequencing data will be analyzed in the context of this study and explored in aggregate with data from other studies to increase researchers' understanding of disease pathobiology and guide the development of new therapeutic approaches.

Results from these analyses will be presented in a separate report.

4.9.2 Subgroup Analyses

To assess the consistency of study results in subgroups defined by demographic and baseline characteristics (including subtypes of triple-negative breast cancer [TNBC]), pCR, EFS, DFS, and OS in these subgroups will be examined. Summaries of EFS, DFS and OS, including unstratified HRs estimated from Cox proportional hazards models and Kaplan-Meier estimates of the median (if reached), will be produced separately for each level of the categorical variables.

"Use of this document is governed by the terms of use on the first page of this document."

4.10 INTERIM ANALYSES

4.10.1 <u>Planned Interim Analysis</u>

An interim analysis will be performed and reviewed by the iDMC at the end of stage 1 (i.e., once all of the approximately 204 patients enrolled in stage 1 have had surgery and pCR assessment). At this interim analysis, a decision concerning the target population for the subsequent stage of the trial has to be made if efficacy or lack thereof cannot be declared after stage 1 (as described in Section 4.4.1.1). This decision is based on the observed pCR proportions (in the ITT and PD-L1–positive populations) in the first stage of the trial and entails either of the following outcomes:

1. The analysis at stage 1 is considered the primary efficacy analysis for pCR with statistical evidence for efficacy in either the ITT (F) or PD-L1–positive population (S) or both. The study will therefore not enroll extra patients in stage 2.

2. If no statistical evidence for efficacy can be found in both F and S, and both the observed difference in pCR proportion between treatment arms in PD-L1-positive population (S) (denoted $\widehat{\Delta}_1^S$) and its complementary (C, IC0 patients) (denoted $\widehat{\Delta}_1^C$) are below the pre-specified threshold, d_S and d_C as specified in Section 4.4.1, respectively, the study will not enroll additional patients in stage 2. Hence, the analysis at stage 1 is considered the primary efficacy analysis for pCR.

3. The study will enroll only patients in S at stage 2, with S being the only target population in the primary efficacy analysis of pCR.

   If $\widehat{\Delta}_1^S \geq d_S$ and $\widehat{\Delta}_1^C < d_C$, the study will enroll only PD-L1-positive patients in stage 2.

4. The study will enroll all-comer patients (F) at stage 2 with F being the only target population in the primary efficacy analysis of pCR.

   If $\widehat{\Delta}_1^S < d_S$ and $\widehat{\Delta}_1^C \geq d_C$, the study will enroll all-comer patients in stage 2; however, only the pCR endpoint for F will be considered in the primary efficacy analysis of pCR.

5. The study will enroll all-comer patients at stage 2, and the co-primary efficacy endpoints of pCR in F and S will be considered in the primary efficacy analysis of pCR.

   If $\hat{\Delta}_1^S \geq d_S$ and $\hat{\Delta}_1^C \geq d_C$, the study will enroll all-comer patients in stage 2, and the co-primary efficacy endpoint of pCR in F and S will be considered in the primary efficacy analysis of pCR.

However, the integrity of the trial is not dependent on strict adherence to these thresholds, i.e. the proposed p-value combination test protects type I error regardless of how the decision regarding stage 2 is made (Brannath, Gutjahr, and Bauer 2012).

A graphical presentation of the decision rules is given in Figure 1 below with more details including selection and determination of the stage-wise boundaries for statistical significance provided in Section 4.4.1.1.

"Use of this document is governed by the terms of use on the first page of this document."

Figure 1 Flowchart of the Decision Rules

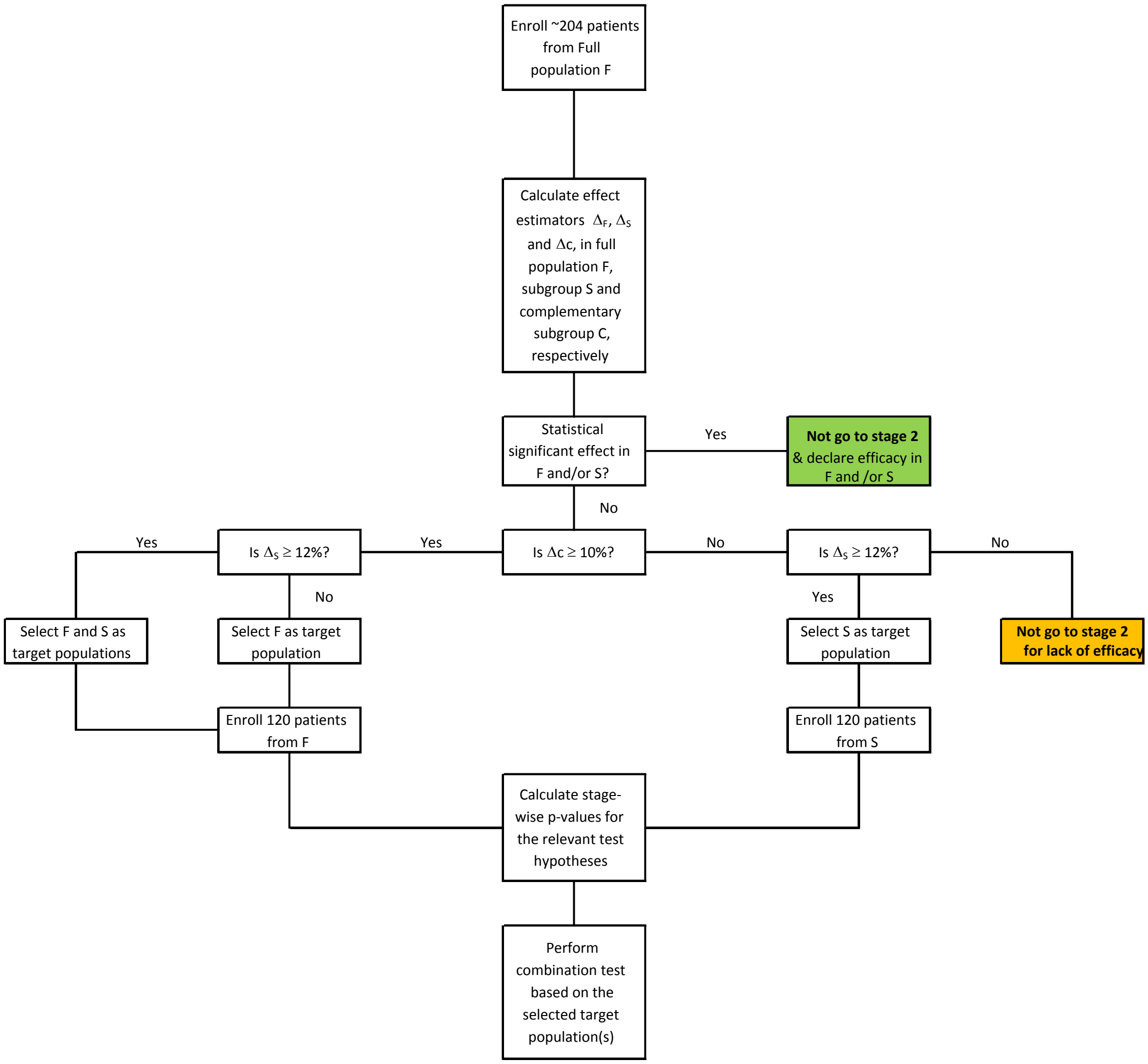

Although these guidelines are in place, the iDMC has authority to deviate from these guidelines if safety or additional efficacy analyses indicate that a different recommendation is more appropriate.

4.10.2 Optional Interim Analysis

To adapt to information that may emerge during the course of this study, the Sponsor may choose to conduct optional interim efficacy analyses after the primary analysis for pCR and before the final analysis for EFS, DFS and OS, if needed (e.g., for regulatory or publication purposes). The decision to conduct an optional interim analysis, along with the rationale, timing, and statistical details for the analysis, will be documented in the Sponsor's trial master file prior to the conduct of the interim analysis.

"Use of this document is governed by the terms of use on the first page of this document."

5. REFERENCES

Brannath W, Gutjahr G, and Bauer P. Probabilistic foundation of confirmatory adaptive designs. J Am Stat Assoc, 2012;107:824–832.

Brookmeyer R and Crowley J. A confidence interval for the median survival time. Biometrics 1982;38:29–4.

Cella D. Manual of the Functional Assessment of Chronic Illness Therapy (FACIT) Measurement System. Center on Outcomes, Research and Education (CORE), Evanston Northwestern Healthcare and Northwestern University, Evanston IL, Version 4, 1997.

Clopper CJ, Pearson ES. The use of confidence or fiducial limits illustrated in the case of the binomial. Biometrika 1934;26:404–13.

Cocks K., King MT, Velikova G, et al. Evidence-based guidelines for determination of sample size and interpretation of the European Organization for the Research and Treatment of Cancer Quality of Life Questionnaire Core 30. J Clin Oncol 2011;29:89–96.

Fayers PM, Aaronson NK, Bjordal K, et al. On behalf of the EORTC Quality of Life Group. The EORTC QLQ-C30 scoring manual (3rd Edition). Published by: European Organisation for Research and Treatment of Cancer, Brussels, 2001.

Jennison C, Turnbull BW. Group sequential methods with applications to clinical trials. Chapman and Hall/CRC, 1999.

Osoba D, Rodrigues G, Myles J, et al. Interpreting the significance of changes in health-related quality of life score. J Clin Oncol 1998;16:139–44.

Peter McCullagh. Regression Models for Ordinal Data. J R Stat Soc Series B Stat Methodol. 1980 42 (2):109-42.

Symmans WF, Peintinger F, Hatzis C, et al. Measurement of residual breast cancer burden to predict survival after neoadjuvant chemotherapy. J Clin Oncol. 2007 Oct 1;25(28):4414–22.

Wassmer G, Brannath W. Group sequential and confirmatory adaptive designs in clinical trials. Heidelberg: Springer, 2016.

"Use of this document is governed by the terms of use on the first page of this document."

Appendix 1 Protocol Synopsis

**TITLE:**	**A PHASE III RANDOMIZED STUDY TO INVESTIGATE THE EFFICACY AND SAFETY OF ATEZOLIZUMAB (ANTI–PD-L1 ANTIBODY) IN COMBINATION WITH NEOADJUVANT ANTHRACYCLINE/NAB-PACLITAXEL-BASED CHEMOTHERAPY COMPARED WITH PLACEBO AND CHEMOTHERAPY IN PATIENTS WITH PRIMARY INVASIVE TRIPLE-NEGATIVE BREAST CANCER**
**PROTOCOL NUMBER:**	WO39392
**VERSION NUMBER:**	5
**EUDRACT NUMBER:**	2016-004734-22
**IND NUMBER:**	123277
**TEST PRODUCT:**	Atezolizumab (RO5541267)
**PHASE:**	III
**INDICATION:**	Triple-negative breast cancer
**SPONSOR:**	F. Hoffmann-La Roche Ltd

Objectives and Endpoints

Study WO39392 (also known as IMpassion031) will evaluate the efficacy, safety, and pharmacokinetics of neoadjuvant nab-paclitaxel and atezolizumab followed by doxorubicin and cyclophosphamide with atezolizumab (referred to as atezolizumab + nab-pac–AC) or neoadjuvant nab-paclitaxel and placebo followed by doxorubicin and cyclophosphamide with placebo (referred to as placebo + nab-pac–AC) in patients with T2–4d triple-negative breast cancer (TNBC). Specific objectives and corresponding endpoints for the study are as follows:

Objectives	Corresponding Endpoints
**Primary Efficacy Objective:**	
• To evaluate the efficacy of atezolizumab + nab-pac–AC compared with placebo + nab-pac–AC in the neoadjuvant setting	• pCR defined as eradication of tumor from both breast and lymph nodes (ypT0/is ypN0) *in the following:*<br>– *All patients (ITT population)*<br>– *Subpopulation of patients with PD-L1–positive tumor status (IC1/2/3)*

"Use of this document is governed by the terms of use on the first page of this document."

Objectives	Corresponding Endpoints
**Secondary Efficacy Objectives:**	
• To evaluate the efficacy of atezolizumab + nab-pac–AC compared with placebo + nab-pac–AC in the neoadjuvant setting	• EFS defined as the time from randomization until documented disease recurrence, progression, or death from any cause in all patients *(ITT population)* and in the subpopulation with PD-L1–*positive* tumor status<br>• *DFS defined as the time from surgery until documented disease recurrence or death from any cause in all patients (ITT population) who undergo surgery and in the subpopulation of patients with PD-L1–positive tumor status who undergo surgery*<br>• OS defined as the time from randomization to the date of death from any cause in all patient*s (ITT population) and in t*he subpopulation with PD-L1–*positive* tumor status
• To evaluate PROs of function and HRQoL associated with atezolizumab + nab-pac–AC compared with placebo + nab-pac–AC, measured by the functional and HRQoL scales of the EORTC QLQ-C30	• Mean and mean changes from baseline score in function (role, physical) and GHS/HRQoL by cycle and between treatment arms as assessed by the functional and HRQoL scales of the EORTC QLQ-C30
**Exploratory Efficacy Objectives:**	
• To evaluate the efficacy of atezolizumab + nab-pac–AC compared with placebo + nab-pac–AC in the neoadjuvant setting	• Proportion of patients undergoing breast-conserving surgery<br>• RCB index<br>• Correlation of RCB with other clinical endpoints (if deemed appropriate)
• To evaluate PROs of disease/treatment-related symptoms associated with atezolizumab + nab-pac–AC compared with placebo + nab-pac–AC, as measured by the EORTC QLQ-C30	• Mean and mean changes from baseline score in disease/treatment-related symptoms by cycle and between treatment arms as assessed by all symptom items/scales of the EORTC QLQ-C30
• To evaluate any treatment burden patients may experience associated with the addition of atezolizumab to nab-pac–AC compared with placebo + nab-pac–AC, as measured by a single item (GP5: "I am bothered by side effects of treatment") from the physical well-being subscale of the FACT-G Quality of Life instrument	• Proportion of patients reporting each response option at each assessment timepoint by treatment arm for item GP5 from the FACT-G
• To evaluate and compare between treatment arms patient's health utility as measured by the EQ-5D-5L questionnaire to generate utility scores for use in economic models	• Utility scores of the EQ-5D-5L questionnaire
**Safety Objective:**	
• To evaluate the safety and tolerability of atezolizumab + nab-pac–AC compared with placebo + nab-pac–AC	• Occurrence and severity of adverse events as defined by NCI CTCAE v4.0

"Use of this document is governed by the terms of use on the first page of this document."

Objectives	Corresponding Endpoints
**Pharmacokinetic Objectives:**	
• To characterize the pharmacokinetics of atezolizumab when administered in combination with nab-pac–AC chemotherapy	• Serum concentration of atezolizumab at specified timepoints
**Immunogenicity Objective:**	
• To evaluate the immune response to atezolizumab	• Incidence of ADAs during the study and the prevalence of ADAs at baseline
**Exploratory Immunogenicity Objective:**	
• To evaluate potential effects of ADAs	• Relationship between ADA status and efficacy, safety, and PK endpoints
**Exploratory Biomarker Objective:**	
• To assess predictive, prognostic, and pharmacodynamic exploratory biomarkers in archival and/or fresh tumor tissue and blood and their association with efficacy endpoints including but not limited to pCR	• Relationship between PD-L1 IHC and efficacy endpoints *other than pCR*<br>• Relationship between tumor derived RNA-based immune gene signatures and efficacy endpoints, including but not limited to pCR<br>• Relationship between tumor-based tumor infiltrating lymphocytes and/or CD8 IHC and efficacy endpoints, including but not limited to pCR<br>• Pharmacodynamic changes in cancer-related immune, stroma and tumor immune biology parameters on-treatment by, but not limited to, gene expression and IHC in baseline, on-treatment, and residual disease tumor tissues<br>• Relationship of baseline and on-treatment plasma biomarkers and efficacy endpoints, including but not limited to pCR

ADA = anti-drug antibody; atezolizumab + nab-pac–AC = nab-paclitaxel and atezolizumab followed by doxorubicin and cyclophosphamide with atezolizumab; *DFS = disease-free survival;* EFS = event-free survival; EORTC = European Organisation for Research and Treatment of Cancer; EQ-5D-5L = EuroQoL 5-Dimension, 5-Level; FACT–G = Functional Assessment of Cancer Therapy–General; GHS = global health status; HRQoL = health-related quality of life; IC = tumor-infiltrating immune cell; IHC = immunohistochemistry; ITT = intent to treat; NCI CTCAE = National Cancer Institute Common Terminology Criteria for Adverse Events; OS = overall survival; pCR = pathologic complete response; PD–L1 = programmed death–ligand 1; PK = pharmacokinetics; placebo + nab-pac–AC = neoadjuvant nab-paclitaxel and placebo followed by doxorubicin and cyclophosphamide with placebo; PRO = patient-reported outcome; QLQ-C30 = Quality of Life Questionnaire Core 30; RCB = residual cancer burden.

Study Design

Description of Study

This is a global Phase III, double-blind, randomized, placebo-controlled study designed to evaluate the efficacy and safety of neoadjuvant treatment with atezolizumab + nab-pac-AC, or placebo + nab-pac–AC in patients eligible for surgery with initial clinically assessed T2-4d TNBC.

Female and male patients aged ≥ 18 years with an Eastern Cooperative Oncology Group (ECOG) performance status of 0 or 1 who have histologically confirmed invasive TNBC with a primary tumor size of > 2 cm are eligible.

"Use of this document is governed by the terms of use on the first page of this document."

Human epidermal growth factor receptor 2 (HER2) and estrogen/progesterone receptor (ER/PR) status will be used to define TNBC. HER2 negativity will be defined by central laboratory assessment using in situ hybridization (ISH) or immunohistochemistry (IHC) assays per American Society of Clinical Oncology/College of American Pathologists (ASCO/CAP) criteria, and ER/PgR negativity will be defined by using IHC per ASCO/CAP criteria. Central laboratory assessment will occur prior to randomization. Patients whose tumors are not confirmed to be triple negative will not be eligible. Patients whose tumor tissue is not evaluable for PD-L1 will not be eligible.

Patients who do not initially meet all eligibility criteria, other than TNBC status, may be rescreened once.

This study has an adaptive design consisting of two stages. Stage 1 is the randomization and treatment of approximately 204 patients. At the end of Stage 1, an interim analysis for efficacy will be done by an iDMC. Then, a recommendation will be made to either continue the study unchanged or to expand the patient population (Stage 2; approximately 120 additional patients). If the recommendation is to expand the patient population into Stage 2, the Sponsor will remain blinded to the results of the interim analysis performed at the end of Stage 1.

Patients who have consented and are eligible will be randomized in a 1:1 ratio to either of the following treatment groups:

- **Arm A**: atezolizumab (840 mg) administered via intravenous (IV) infusion Q2W in combination with nab-paclitaxel (125 mg/m^2) administered via IV infusion QW for 12 weeks followed by atezolizumab (840 mg) administered Q2W in combination with doxorubicin (60 mg/m^2) + cyclophosphamide (600 mg/m^2) administered Q2W via IV infusions with filgrastim/pegfilgrastim support for 4 cycles. Patients randomized to the atezolizumab arm will continue to receive unblinded atezolizumab post-surgery at a fixed dose of 1200 mg by IV infusion every 3 weeks (Q3W) for 11 cycles, for a total of approximately 12 months of atezolizumab therapy.
- **Arm B**: placebo administered Q2W via IV infusion in combination with nab-paclitaxel (125 mg/m^2) administered QW via IV infusion for 12 weeks followed by placebo administered Q2W in combination with doxorubicin (60 mg/m^2) + cyclophosphamide (600 mg/m^2) administered Q2W via IV infusions with filgrastim/pegfilgrastim support for 4 cycles. Patients randomized to the placebo arm will be unblinded post-surgery and will continue to be followed.

For patients in Stage 1 of the the study, randomization will be stratified by the following factors:

- *American Joint Committee on Cancer (AJCC) stage at diagnosis (II vs. III; see below for evaluation and classification of lymph nodes)*
- *Tumor PD-L1 status (tumor-infiltrating immune cell [IC] IC0 [<1% PD-L1 expressing IC per tumor area] vs. IC1/2/3 [≥1% PD-L1 expressing IC per tumor area])*

Depending on the iDMC recommendation, Stage 2 could follow either an all-comer or PD-L1 enrichment design. If an all-comer design is recommended, the patients enrolled in Stage 2 will be stratified the same as patients enrolled in Stage 1. If an enrichment design is recommended, only PD-L1–positive patients will be enrolled, and the AJCC stage at diagnosis will be used as the sole randomization stratification factor.

Patients who discontinue neoadjuvant therapy early as a result of disease progression must be discontinued from all study treatment, will be managed as per local practice, and will be followed for survival only. Patients who discontinue prematurely from the study will not be replaced.

Any patient who receives non-protocol therapy prior to surgery will be discontinued from study treatment and will be managed as per local practice; these patients will remain on study for survival follow-up.

"Use of this document is governed by the terms of use on the first page of this document."

The primary efficacy endpoint (pathologic complete response [pCR]; ypT0/is ypN0) will be established via local review following completion of neoadjuvant therapy and surgery. Pathologists who review study specimens must utilize the evaluations and assessments outlined in the Pathology Manual. Investigator/individual patient unblinding will occur after pCR assessment. Surgery should be performed at least 14 days after the last dose of neoadjuvant therapy but no later than 6 weeks after the last infusion. Platelet counts should be checked prior to surgery and should be ≥75,000 cells/μL.

Patients with clinically positive axillary nodes by physical examination or by any radiographic imaging at baseline should undergo fine-needle aspiration or core-needle biopsy prior to randomization followed by axillary lymph node dissection (ALND) at time of definitive surgery. The results of the baseline fine-needle aspiration or core-needle biopsy will determine the nodal staging, so that patients with a positive biopsy result should be staged as lymph-node-positive (N1-N3c) whereas patients with a negative or equivocal biopsy result should be staged as lymph-node-negative (N0) regardless of any other clinical measurements.

In patients with clinically or fine-needle biopsy (FNA)/core needle biopsy-proven negative axillary nodes at baseline, axillary surgical management after completion of neoadjuvant therapy should include sentinel lymph node biopsy (SLNB) or ALND. If SLNB is conducted, it is strongly recommended that more than one lymph node (two to three minimum) be removed and all patients with positive macrometastases in sentinel nodes should undergo ALND regardless of the number of positive nodes. All patients with T4 tumors should undergo ALND or current standard of care as described in international or national guidelines.

Postoperative patient management for those in either treatment arm should include radiotherapy as clinically indicated, and management of patients who do not achieve a pCR should follow current standard-of-care guidelines. For those randomized to receive atezolizumab, patients may receive this therapy simultaneously with atezolizumab.

For those randomized to receive atezolizumab, the first dose of postoperative atezolizumab should be administered within 45 days of surgery.

Efficacy, safety, laboratory measurements, patient-reported outcomes (PROs), and pharmacokinetics will be assessed throughout the study. The first 26 patients enrolled (approximately 13 patients in the control arm and approximately 13 patients in the atezolizumab containing arm) will undergo additional cardiac safety monitoring as part of a cardiac safety cohort. Following completion of study treatment and surgery, all patients will continue to be followed for efficacy, safety, and PRO objectives until the end of the study. No interim efficacy analyses for early stopping are planned.

Safety assessments will include the occurrence and severity of adverse events and laboratory abnormalities graded per National Cancer Institute Common Terminology Criteria for Adverse Events (NCI CTCAE) Version 4.0. Laboratory safety assessments will include the regular monitoring of hematology and blood chemistry. Serum samples will be collected to monitor chemotherapy and atezolizumab pharmacokinetics and to detect the presence of antibodies to atezolizumab. Patient samples, including tumor tissues, as well as plasma and blood, will be collected for exploratory biomarker assessments.

An iDMC will evaluate *the primary efficacy endpoint of pCR in the intent-to-treat (ITT) population (defined as all randomized patients) and in the PD-L1–positive subpopulation based on an interim analysis of efficacy data from the Stage 1 patients (approximately 204 enrolled patients). The iDMC will make a recommendation either to continue the study unchanged or to enroll an additional 120 patients (Stage 2). The decision rules to be applied at the interim analysis will be clearly expressed to the iDMC and documented in the iDMC charter so that the study can be conducted with the Sponsor remaining completely blinded to all results at this stage.*

The iDMC will evaluate safety data and study conduct on a regular basis during the study until the primary analysis of pCR, which is performed at Stage 1 (approximately 204 patients) or Stage 2 (approximately 324 patients), if the iDMC recommends extending the target population. Afterwhich, iDMC review of the study data will be discontinued.

Sponsor affiliates will be excluded from iDMC membership. The iDMC will follow a charter that outlines the iDMC roles and responsibilities.

"Use of this document is governed by the terms of use on the first page of this document."

Number of Patients

Approximately 204 patients will be enrolled in this study during Stage 1 and approximately 120 additional patients will be enrolled if the iDMC recommends to expand to Stage 2. Enrollment will take place at approximately 75 global sites.

Target Population

Inclusion Criteria

Patients must meet the following criteria for study entry:

- Signed Informed Consent Form
- Ability to comply with protocol, in the investigator's judgment
- Women or men aged ≥ 18 years
- ECOG performance status of 0 or 1
- Histologically documented TNBC (negative HER2, ER, and PgR status); HER2 negativity will be defined by central laboratory assessment using ISH or IHC assays per ASCO/CAP criteria and ER/PgR negativity will be defined by central laboratory assessment using IHC per ASCO/CAP criteria. Central laboratory assessment will occur prior to randomization.

    Patients with multifocal tumors (more than one tumor confined to the same quadrant as the primary tumor) are eligible provided all discrete lesions are sampled and centrally confirmed as TNBC.

- Confirmed tumor PD-L1 evaluation as documented through central testing of a representative tumor tissue specimen

    In Stage 2, if the iDMC recommendation is to expand to a PD-L1–positive population, only patients with confirmed tumor PD-L1 positive (IC1/2/3) will be considered eligible.

- Primary breast tumor size of > 2 cm by at least one radiographic or clinical measurement
- Stage at presentation: cT2–cT4, cN0–cN3, cM0
- Patient agreement to undergo appropriate surgical management including axillary lymph node surgery and partial or total mastectomy after completion of neoadjuvant treatment
- Baseline LVEF ≥ 53% measured by echocardiogram (ECHO) or multiple-gated acquisition (MUGA) scans
- Adequate hematologic and end-organ function, as defined by the following laboratory results obtained within 14 days prior to the first study treatment:

    ANC ≥ 1500 cells/μL (without granulocyte colony–stimulating factor [G-CSF] support within 2 weeks prior to Cycle 1, Day 1)

    Lymphocyte count ≥ 500 cells/μL

    Platelet count ≥ 100,000 cells/μL (without transfusion within 2 weeks prior to Cycle 1, Day 1)

    Hemoglobin ≥ 9.0 g/dL

    AST, ALT, and alkaline phosphatase ≤ 2.5 × the upper limit of normal (ULN)

    Serum bilirubin ≤ 1.0 × ULN

        Patients with known Gilbert syndrome who have serum bilirubin level ≤ 3 × ULN may be enrolled.

    For patients not receiving therapeutic anticoagulation: INR or aPTT ≤ 1.5 × ULN within 14 days prior to initiation of study treatment

    For patients receiving therapeutic anticoagulation: stable anticoagulant regimen and stable INR during the 14 days immediately preceding initiation of study treatment

    Creatinine clearance ≥ 30 mL/min (calculated using the Cockcroft-Gault formula)

    Serum albumin ≥ 25 g/L (≥ 2.5 g/dL)

"Use of this document is governed by the terms of use on the first page of this document."

- Representative formalin-fixed, paraffin-embedded (FFPE) tumor specimen in paraffin blocks (preferred) or at least 20 unstained slides, with an associated pathology report documenting ER, PgR, and HER2 negativity

  Tumor tissue should be of good quality based on total and viable tumor content and must be evaluated for PD-L1 expression prior to enrollment. Patients whose tumor tissue is not evaluable for PD-L1 expression are not eligible.

  If multiple tumor specimens are submitted, patients may be eligible if at least one specimen is evaluable for PD-L1. For the purpose of stratification, the PD-L1 score of the patient will be the maximum PD-L1 score among the samples.

    In Stage 2, if the recommendation from the iDMC is to expand to a PD-L1–positive population, no further stratification based on PD-L1 status will be conducted.

    Acceptable samples include core-needle biopsies for deep tumor tissue (minimum three cores) or excisional, incisional, punch, or forceps biopsies for cutaneous, subcutaneous, or mucosal lesions.

    Fine-needle aspiration, brushing, and cell pellet from cytology specimens are not acceptable.

- For women of childbearing potential: agreement to remain abstinent (refrain from heterosexual intercourse) or use contraceptive methods, and agreement to refrain from donating eggs, as defined below:

  Women must remain abstinent or use contraceptive methods that result in a failure rate of $<$ 1% per year during the treatment period and for at least 5 months after the last dose of atezolizumab, or 1 month after the last dose of nab-paclitaxel, or 6 months after the last dose of doxorubicin, or 12 months after the last dose of cyclophosphamide, whichever is later. Women must refrain from donating eggs during this same period.

  A woman is considered to be of childbearing potential if she is postmenarcheal, has not reached a postmenopausal state ($\geq$ 12 continuous months of amenorrhea with no identified cause other than menopause), and has not undergone surgical sterilization (removal of ovaries and/or uterus).

  Examples of contraceptive methods with a failure rate of $<$ 1% per year when used consistently and correctly, include combined (estrogen and progestogen containing) hormonal contraception associated with inhibition of ovulation, progestogen-only hormonal contraception associated with inhibition of ovulation, bilateral tubal occlusion; male sterilization; intrauterine devices; intrauterine hormone-releasing system; and sexual abstinence.

  The reliability of sexual abstinence should be evaluated in relation to the duration of the clinical study and the preferred and usual lifestyle of the patient. Periodic abstinence (e.g., calendar, ovulation, symptothermal, or postovulation methods) and withdrawal are not acceptable methods of contraception.

- For men: agreement to remain abstinent (refrain from heterosexual intercourse) or use contraceptive measures and agreement to refrain from donating sperm, as defined below:

  With female partners of childbearing potential, men must remain abstinent or use a condom plus an additional contraceptive method that together result in a failure rate of $<$ 1% per year during the treatment period and for 6 months after the last dose of nab-paclitaxel, cyclophosphamide, or doxorubicin. Men must refrain from donating sperm during this same period.

  With pregnant female partners, men must remain abstinent or use a condom during the treatment period and for 6 months after the last dose of nab-paclitaxel, cyclophosphamide, or doxorubicin to avoid exposing the embryo.

  The reliability of sexual abstinence should be evaluated in relation to the duration of the clinical trial and the preferred and usual lifestyle of the patient. Periodic abstinence (e.g., calendar, ovulation, symptothermal, or postovulation methods) and withdrawal are not acceptable methods of contraception.

"Use of this document is governed by the terms of use on the first page of this document."

- Women who are not postmenopausal (≥ 12 months of non–therapy-induced amenorrhea) or have undergone a sterilization procedure must have a negative serum pregnancy test result within 14 days prior to initiation of study drug
- Willingness and ability to comply with scheduled visits, treatment plans, laboratory tests, and other study procedures, including the completion of PRO questionnaires

Exclusion Criteria

Patients who meet any of the following criteria will be excluded from study entry:

- Prior history of invasive breast cancer
- Stage IV (metastatic) breast cancer
- Prior systemic therapy for treatment and prevention of breast cancer
- Previous therapy with anthracyclines or taxanes for any malignancy
- History of ductal carcinoma in situ (DCIS), except for patients treated exclusively with mastectomy > 5 years prior to diagnosis of current breast cancer
- History of pleomorphic lobular carcinoma in situ (LCIS), except for patients surgically managed > 5 years prior to diagnosis of current breast cancer (note that patients with non-pleomorphic LCIS [either untreated or treated with surgery] are allowed)
- Bilateral breast cancer
- Undergone incisional and/or excisional biopsy of primary tumor and/or axillary lymph nodes. Patients who have undergone SLNB at the baseline may be eligible only if the SLNB was free of invasive carcinoma. Any patient with a positive SLN (involved with invasive carcinoma) is ineligible to participate in this study.
- Axillary lymph node dissection prior to initiation of neoadjuvant therapy
- History of other malignancy within 5 years prior to screening, with the exception of those with a negligible risk of metastasis or death (e.g., 5-year OS of > 90%), such as adequately treated carcinoma in situ of the cervix, non-melanoma skin carcinoma, localized prostate cancer, or Stage I uterine cancer
- History of cerebrovascular accident within 12 months prior to randomization
- Cardiopulmonary dysfunction as defined by any of the following prior to randomization:

  History of NCI CTCAE v4.0 Grade ≥ 3 symptomatic congestive heart failure or New York Heart Association (NYHA) criteria Class ≥ II

  Angina pectoris requiring anti-anginal medication, serious cardiac arrhythmia not controlled by adequate medication, severe conduction abnormality, or clinically significant valvular disease

  High-risk uncontrolled arrhythmias (i.e., atrial tachycardia with a heart rate > 100/min at rest, significant ventricular arrhythmia [ventricular tachycardia], or higher-grade atrioventricular [AV]-block [second-degree AV-block Type 2 [Mobitz 2] or third degree AV-block])

  Significant symptoms (Grade ≥ 2) relating to left ventricular dysfunction, cardiac arrhythmia, or cardiac ischemia

  Myocardial infarction within 12 months prior to randomization

  Uncontrolled hypertension (systolic blood pressure > 180 mmHg and/or diastolic blood pressure > 100 mmHg)

  Evidence of transmural infarction on ECG

  Requirement for oxygen therapy
- History of severe allergic, anaphylactic, or other hypersensitivity reactions to chimeric or humanized antibodies or fusion proteins
- Known hypersensitivity to biopharmaceuticals produced in Chinese hamster ovary cells
- Known allergy or hypersensitivity to the components of the atezolizumab formulation

"Use of this document is governed by the terms of use on the first page of this document."

- Known allergy or hypersensitivity to the components of the nab-paclitaxel, cyclophosphamide, or doxorubicin formulations
- Known allergy or hypersensitivity to filgrastim or pegfilgrastim formulations
- Active or history of autoimmune disease or immune deficiency, including but not limited to myasthenia gravis, myositis, autoimmune hepatitis, systemic lupus erythematosus, rheumatoid arthritis, inflammatory bowel disease, antiphospholipid syndrome, Wegener granulomatosis, Sjögren syndrome, Guillain-Barré syndrome, or multiple sclerosis with the following exceptions:

    Patients with a history of autoimmune-related hypothyroidism on a stable dose of thyroid replacement hormone may be eligible for this study.

    Patients with controlled Type I diabetes mellitus on a stable dose of insulin regimen may be eligible for this study.

    Patients with eczema, psoriasis, lichen simplex chronicus, or vitiligo with dermatologic manifestations only (e.g., patients with psoriatic arthritis are excluded) are permitted provided all of following conditions are met:

        Rash must cover $<$ 10% of body surface area

        Disease is well controlled at baseline and requires only low-potency topical corticosteroids

        No occurrence of acute exacerbations of the underlying condition requiring psoralen plus ultraviolet A radiation, methotrexate, retinoids, biologic agents, oral calcineurin inhibitors, or high-potency or oral corticosteroids within the previous 12 months

- History of idiopathic pulmonary fibrosis, organizing pneumonia (e.g., bronchiolitis obliterans), drug-induced pneumonitis, idiopathic pneumonitis, or evidence of active pneumonitis on screening chest computed tomography (CT) scan

    History of radiation pneumonitis in the radiation field (fibrosis) is permitted.

- Positive HIV test at screening
- Active hepatitis B virus (HBV) infection, defined as having a positive hepatitis B surface antigen (HBsAg) test at screening

    Patients with a past or resolved HBV infection, defined as having a negative HBsAg test and a positive total hepatitis B core antibody (HBcAb) test at screening, are eligible for the study if active HBV infection is ruled out on the basis of HBV DNA viral load per local guidelines.

- Active hepatitis C virus (HCV) infection, defined as having a positive HCV antibody test at screening

    Patients who have a positive HCV antibody test are eligible for the study if a polymerase chain reaction (PCR) assay is negative for HCV RNA.

- Active tuberculosis
- Severe infections within 4 weeks prior to initiation of study treatment, including but not limited to hospitalization for complications of infection, bacteremia, or severe pneumonia
- Treatment with therapeutic oral or IV antibiotics within 2 weeks prior to initiation of study treatment

    Patients receiving prophylactic antibiotics (e.g., for prevention of a urinary tract infection or to prevent chronic obstructive pulmonary disease exacerbation) are eligible for the study.

- Major surgical procedure within 4 weeks prior to initiation of study treatment or anticipation of need for a major surgical procedure (other than anticipated breast surgery) during the course of the study
- Prior allogeneic stem cell or solid organ transplantation

"Use of this document is governed by the terms of use on the first page of this document."

- Administration of a live attenuated vaccine within 4 weeks prior to initiation of study treatment or anticipation of need for such a vaccine during the atezolizumab/placebo treatment or within 5 months after the last dose of atezolizumab/placebo

    Patients must agree not to receive live, attenuated influenza vaccine (e.g., FluMist®) within 4 weeks prior to randomization, during treatment or within 5 months following the last dose of atezolizumab (for patients randomized to atezolizumab).

- Any other disease, metabolic dysfunction, physical examination finding, or clinical laboratory finding giving reasonable suspicion of a disease or condition that contraindicates the use of an investigational drug or that may affect the interpretation of the results or render the patient at high risk from treatment complications
- Prior treatment with CD137 agonists or immune checkpoint–blockade therapies, including anti-CD40, anti–CTLA-4, anti–PD-1, and anti–PD-L1 therapeutic antibodies
- Treatment with systemic immunostimulatory agents (including but not limited to interferons, IL-2) within 4 weeks or 5 half-lives of the drug, whichever is longer, prior to initiation of study treatment
- Treatment with systemic immunosuppressive medications (including but not limited to prednisone, cyclophosphamide, azathioprine, methotrexate, thalidomide, and anti–tumor necrosis [anti-TNF] factor agents) within 2 weeks prior to initiation of study treatment or anticipation of need for systemic immunosuppressive medications during the study

    Patients who have received acute, low-dose, systemic immunosuppressant medications (e.g., a one-time dose of dexamethasone for nausea) may be enrolled in the study after discussion with and approval by the Medical Monitor.

    The use of inhaled corticosteroids and mineralocorticoids (e.g., fludrocortisone for adrenal insufficiency) is allowed.

- Pregnant or lactating, or intending to become pregnant during the study

    Women of childbearing potential must have a negative serum pregnancy test result within 14 days prior to initiation of study treatment.

End of Study

The end of the study is defined as the date when the last patient, last visit (LPLV) occurs for evaluation of secondary endpoints, or the date of Sponsor decision to end the study, whichever is earlier.

Length of Study

The total duration of the study *for Stage 1* is expected to be approximately 51 months. *If the iDMC recommends to expand enrollment (Stage 2), the duration of the study may increase to approximately 74 months.*

<u>Investigational Medicinal Products</u>

The investigational medicinal products (IMPs) for this study are atezolizumab, its placebo, and nab-paclitaxel.

Atezolizumab and Placebo

Patients will receive 840 mg atezolizumab or placebo administered by IV infusion Q2W (14 [±3] days) for 20 weeks (i.e., 10 doses) in combination with nab-paclitaxel, doxorubicin, and cyclophosphamide chemotherapy (see below). Postoperatively, patients randomized to the atezolizumab arm will continue to receive unblinded atezolizumab post-surgery at a fixed dose of 1200 mg by IV infusion every 3 weeks (Q3W) for 11 cycles, for a total of approximately 12 months of atezolizumab therapy; patients randomized to the placebo arm will stop receiving placebo.

Nab-Paclitaxel

Nab-paclitaxel will be administered as background treatment along with the non-IMPs doxorubicin, cyclophosphamide, and filgrastim/pegfilgrastim as specified below.

"Use of this document is governed by the terms of use on the first page of this document."

Non-Investigational Medicinal Products

Patients will receive nab-paclitaxel (125 mg/m^2) administered via IV infusion given over 30 minutes weekly for 12 weeks followed by doxorubicin (60 mg/m^2) + cyclophosphamide (600 mg/m^2) administered via IV infusion Q2W with filgrastim/pegfilgrastim support for 4 cycles (i.e., a total of 4 doses of doxorubicin and cyclophosphamide). The dose of cyclophosphamide should be capped at 1200 mg.

Statistical Methods

Primary Analysis

The primary efficacy objective for this study is to evaluate the efficacy of neoadjuvant atezolizumab + nab-pac–AC compared with placebo + nab-pac–AC in patients with T2-4d TNBC, as measured by pCR defined as eradication of tumor from both breast and lymph nodes (ypT0/is ypN0). The primary efficacy endpoint will be established following completion of neoadjuvant therapy and surgery.

The ITT *as well as PD-L1–positive* population*s* will be used for the primary analysis of pCR. In the primary analysis, patients whose pCR assessment was missing will be counted as not achieving a pCR. An estimate of the pCR rate and its 95% CI (Clopper and Pearson 1934) will be calculated for each treatment arm. The difference in pCR rates will be provided with 95% CI, using the normal approximation to the binomial distribution. *For the ITT population, t*he Cochran-Mantel-Haenszel χ^2 test stratified according to tumor PD-L1 status (IC0 vs. IC1/2/3) and clinical stage at presentation (Stage II vs. III) will be used to test pCR rates between treatment groups at a two-sided significance level of 5%. *For the PD-L1–positive population, similar* test will *be used with stratification only for clinical stage at presentation. An unstratified* χ^2 *versions of these tests will* also be provided as a sensitivity analysis.

Determination of Sample Size

The study will first randomize approximately N_1*=204 patients in Stage 1 (1:1 randomization ratio). Based on information from these patients and a pre-specified adaptive rule, the decision will be made regarding whether or not to randomize approximately* N_2*=120 patients in Stage 2 (1:1 randomization ratio).*

The pre-defined one-sided type I error for the interim analysis at Stage 1 is α_1*=0.0125 (i.e., 50% of the total type I error). The co-primary endpoints in the ITT and PD-L1-positive populations at Stage 1 will be tested using a closed testing procedure using Simes' test for the intersection hypothesis. Test statistics in ITT and PD-L1-positive populations are always positively correlated, hence type I error rate control of the Simes test can be guaranteed in general.*

Importantly, the p-value p_1 *based on Stage 1 data only and the combination p-value* p_{comb} *have the same joint distribution under the null hypothesis as the p-values from a group-sequential test with two stages at information times* $t_1 = w_1$ *and* $t_2 = 1$*. Thus standard statistical software for group sequential designs can be used for the determination of critical values for the adaptive design. As the study design uses a critical value of* α_1*=0.0125 (i.e., 50% of the total type I error) for Stage 1, this implies that a critical value of* α_{comb}*=0.0184 can be applied to the combination p-values.*

"Use of this document is governed by the terms of use on the first page of this document."

Appendix 2 Schedule of Activities (for Both Stage 1 and Stage 2)

	Screening [a]	Treatment			Completion of Study Therapy/Early Term. Visit [c]	
		Neoadjuvant Treatment (28-Day Cycles) (Cycles 1–5; Weeks 1–20)	Pre-Surgery Visit/ Surgery [b]	Arm A: Adjuvant Treatment Arm B: Monitoring (21-Day Cycles) (Cycles 6–16)		
	Days –28 to –1	Day 1 (±3 days)		Day 1 (±3 days)	≤30 Days after Last Dose (Arm A) or Last Monitoring Visit (Arm B)	Survival Follow-Up [d]
Informed consent	x [e]					
Baseline tumor tissue sample submission for HER2 and ER/PgR determination and exploratory biomarkers (mandatory)	x [f]					
Demographic data	x					
Medical history and baseline conditions	x					
Disease status assessments [g]	x	x	x [g]	x [g]	x [g]	x [g]
Tumor Staging [h]	x					
Ultrasound [g]	x		x			
EORTC QLQ-C30, EQ-5D-5L [i]		x		x	x	x [j]
FACT-G, Single Item GP5 [i]		x [k]		x	x	x [j]
Vital signs [l]	x	On each infusion day	x	x [m]	x	
Weight	x	x		x [m]		
Height	x					

"Use of this document is governed by the terms of use on the first page of this document."

Appendix 2 Schedule of Activities (for Both Stage 1 and Stage 2) (cont.)

Complete physical examination [n]	x					
Limited physical examination [o]		x [g]	x	x [m]	x	
ECOG Performance Status [p]	x	x	x	x	x	
ECG (12-lead) [q]	x	As clinically indicated				
ECHO or MUGA scan [r]	x	As described in footnote “r”			x	x
Spirometry (FVC, FEV_1, FEV_1:FVC, $FEF_{25\text{-}75}$)	x	As clinically indicated				
Hematology [s]	x [t]	On each infusion day	x	x [m]	x	
Chemistry [u]	x [t]	On each infusion day	x	x [m]	x	
Pregnancy test [v]	x [t]	x [v]		x [v]	x [v]	x [v]
Coagulation (INR, aPTT)	x [t]		x		x	
TSH, free T3 (or total T3 [w]), free T4	x [t]	x [w]			x	
Viral serology [x]	x [t]					
Urinalysis [y]	x [t]	As clinically indicated				
Serum PK sample for atezolizumab		See Appendix 2 for detailed schedule				
Serum ADA sample for atezolizumab		See Appendix 2 for detailed schedule				
Blood and plasma samples for biomarkers		See Appendix 2 for detailed schedule				
Blood sample for RBR (optional) [z]		x				

"Use of this document is governed by the terms of use on the first page of this document."

Appendix 2 Schedule of Activities (for Both Stage 1 and Stage 2) (cont.)

Tumor tissue (fresh sample preferred) at screening, on-study, and time of disease recurrence [aa]	See Appendix 3 for detailed schedule					
Radiographic assessments (e.g., CT scan, MRI, PET scan)	As clinically indicated					
Bilateral mammogram	x [bb]				x	x [cc]
Concomitant medications	x [dd]	x		x [ee]	x [ee]	x [g, ff]
Adverse events [gg]	x [gg]	x [gg]		x	x	x [gg]
Study treatment administration [hh]		x		x [ii, m]		
Survival follow-up and anti-cancer treatment						x [d, jj]

AC = doxorubicin + cyclophosphamide; ADA = anti-drug antibody; CT = computed tomography; ECHO = echocardiogram; ECOG = Eastern Cooperative Oncology Group; eCRF = electronic Case Report Form; EORTC QLQ-C30 = European Organisation for Research and Treatment of Cancer Quality of Life Questionnaire Core 30; EQ-5D-5L = EuroQoL 5-Dimension, 5-Level; ER = estrogen receptor; FACT-G = Functional Assessment of Cancer Therapy-General; FCV = Forced Vital Capacity; FEV_1 = Forced Expiratory Volume 1; FEF_{25-75} = Forced Expiratory Flow 25%–75%; FFPE = formalin-fixed, paraffin-embedded; HBcAb = hepatitis B core antibody; HBsAb = hepatitis B surface antibody; HBsAg = hepatitis B surface antigen; HBV = hepatitis B virus; HCV = hepatitis C virus; HER2 = human epidermal growth factor receptor 2; MRI = magnetic resonance imaging; MUGA = multiple-gated acquisition; PD-L1 = programmed death–ligand-1; PET = positron emission tomography; PgR = progesterone receptor; PK = pharmacokinetic; PRO = patient-reported outcome; RBR = Research Biosample Repository; T3 = triiodothyronine; T4 = thyroxine; Term. = termination; TSH = thyroid-stimulating hormone.

Notes: On treatment days, all assessments should be performed prior to dosing, unless otherwise specified.

Assessments shaded in gray should be performed as scheduled, but the associated data do not need to be recorded on the eCRF (except in the case of an adverse event).

If treatment is withheld (e.g., by adverse events or delays in initiating post-surgical therapy), the schedule of assessments should be held accordingly (e.g., Day 1 of Week 21 = first administration of atezolizumab post-surgery or Day 1 of Cycle 6).

[b] Results of standard-of-care tests or examinations performed prior to obtaining informed consent and within 30 days prior to Day 1 may be used; such tests do not need to be repeated for screening.

[b] Pre-surgical visit and associated assessments should occur within 14 days of surgery. Surgery should be conducted no earlier than 14 days and no later than 6 weeks after last dose of neoadjuvant therapy. Platelet counts should be checked prior to surgery and should be ≥75,000 cells/µL.

"Use of this document is governed by the terms of use on the first page of this document."

Appendix 2 Schedule of Activities (for Both Stage 1 and Stage 2*)* (cont.)

[c] Patients who discontinue study treatment will return to the clinic for a treatment discontinuation visit not more than 30 days after the last dose of study treatment.

[d] The survival follow-up period begins from the date of treatment completion/early termination visit, *and has a* duration of up to *approximately* 51 months from the date of randomization of the first patient *in Stage 1). This may increase to approximately 74 months if the study includes Stage 2 patients.* Visit windows are ±28 days for quarterly and semiannual assessments.

[e] Informed consent must be documented before any study-specific screening procedure is performed and may be obtained more than 28 days before initiation of study treatment.

[f] After signing of the Informed Consent Form, retrieval and submission of tumor tissue sample can occur outside the 28-day screening period. Tumor tissue should be of good quality based on total and viable tumor content (sites will be informed if the quality of the submitted specimen is inadequate to determine tumor PD-L1 status). An FFPE block or at least 20 unstained slides should be provided. Fine-needle aspiration, brushing, cell pellets from pleural effusion, and lavage samples are not acceptable. For core-needle biopsy specimens, at least three cores should be submitted for evaluation. Retrieval of tumor sample can occur outside the 28-day screening period.

[g] Assessment of primary tumor and regional lymph nodes should be done by physical examination at baseline and prior to administration of each cycle of study treatment during neoadjuvant therapy. Additionally, standard breast imaging modality should include an ultrasound of breast and axilla disease. (Physical examination and ultrasound is mandatory within 28 days prior to randomization and within 14 days pre-surgery.) At baseline, if there is evidence of suspicious axillary lymph nodes, then fine-needle aspiration is required. Ultrasound-detected axillary lymph nodes suspicious of malignancy include those with cortical thickness >2 mm. Disease status based on all available clinical assessments should be documented every 3 months during adjuvant study treatment and follow-up up to 3 years after surgery and every 6 months thereafter. In addition, liver function tests, bone scans, chest X-ray/diagnostic CT scan, liver imaging, and/or other radiographic modalities may be considered when clinically indicated to exclude metastatic disease; these assessments should be performed within a timeline as per current local standard of practice. Whenever possible, disease recurrence should be confirmed pathologically. If disease recurrence is diagnosed at any time during the study, patients will discontinue scheduled study assessments and will be followed for survival, anti-cancer medications, and new relapse events.

[h] See Section 4.5.6 in protocol.

[i] All PRO assessments (EORTC QLQ-C30, followed by the FACT-G single item GP5, and then the EQ-5D-5L questionnaires) must be completed by the patient at the investigational site at the start of the clinic visit before discussion of the patient's health state, lab results or health record, before administration of study treatment, and/or prior to the performance of any other study assessments that could bias the patient's responses. Interview assessment by a member of the clinical staff will be allowed if the patient is not able to complete the measure on her or his own. Study personnel should review all questionnaires for completeness before the patient leaves the investigational site.

The EORTC QLQ-C30 and EQ-5D-5L questionnaires will be completed by patients at baseline (Cycle 1, Day 1) and on Day 1 of every cycle thereafter. The FACT-G, single item GP5 will not be completed by patients at the baseline visit (Cycle 1, Day 1).

"Use of this document is governed by the terms of use on the first page of this document."

Appendix 2 Schedule of Activities (for Both Stage 1 and Stage 2*)* (cont.)

[j] Patients who discontinue study treatment for any reason will continue to complete the EORTC QLQ-C30, FACT-G single item GP5, and EQ-5D-5L questionnaires in-clinic during the survival follow-up period at the following timepoints: every 3 months (±28 days) for Year 1, every 6 months (± 28 days) for years 2-3, and then annually (±28 days) thereafter.

[k] While on study treatment, all patients will complete the FACT-G, single item GP5 beginning on Cycle 2, Day 1 and at Day 1 of every cycle thereafter.

[l] Includes respiratory rate, pulse rate, systolic and diastolic blood pressure while the patient is in a seated position, and temperature. Record abnormalities observed at baseline on the General Medical History and Baseline Conditions eCRF. At subsequent visits, record new or worsened clinically significant abnormalities on the Adverse Event eCRF. For the first infusion, vital signs should be measured within 60 minutes prior to the infusion and, if clinically indicated, every 15 (±5) minutes during and 30 (±10) minutes after the infusion. For subsequent infusions, vital signs should be measured within 60 minutes prior to the infusion and, if clinically indicated or if symptoms occurred during the previous infusion, during and 30 (±10) minutes after the infusion.

[m] For patients in Arm A only.

[n] Includes evaluation of the head, eyes, ears, nose, throat, and the cardiovascular, dermatological, musculoskeletal, respiratory, gastrointestinal, and neurological systems. Record abnormalities observed at baseline on the General Medical History and Baseline Conditions eCRF. At subsequent visits, record new or worsened clinically significant abnormalities on the Adverse Event eCRF.

[o] Perform a limited, symptom-directed examination at specified timepoints and as clinically indicated at other timepoints. Record new or worsened clinically significant abnormalities on the Adverse Event eCRF.

[p] See Appendix 10.

[q] ECG recordings will be obtained during screening and as clinically indicated. Patients should be resting in a supine position for at least 10 minutes prior to ECG recording.

[r] Cardiac monitoring (ECHO or MUGA scan) will be performed on all patients enrolled in the study. ECHO is the preferred method. The same method used for a given patient *at* screening should be used throughout the study. ECHO or MUGA scan should be obtained at **baseline** *and* **after the second dose of AC** (*which would correspond to* Week 16 ±1 week *if no study treatment interruptions or discontinuations have occurred) during neoadjuvant study treatment. During the adjuvant (Arm A) or monitoring (Arm B) phase of the study, ECHO or MUGA scan should be obtainedat* ***Cycle 6, Day 1*** *(which would approximately correspond to* Week *21 ±* 1 week *if no study treatment interruptions or discontinuations have occurred), and* ***every 3 months afterwards*** *while on the Adjuvant/Monitoring Phase (which would correspond to Cycle 10, Day 1 and Cycle 14, Day 1, or approximately* Week *33 ±*1 week, and Week *45 ±*1 week *respectively, if no treatment interruptions or discontinuations have occurred*). ECHO or MUGA scan should be obtained at the **early termination visit** if not performed within the previous 6 weeks. During the survival follow-up period, ECHO or MUGA scan should be obtained **annually until the end of study**. For additional cardiac screening tests for patients in the cardiac safety cohort, please see Appendix 4.

[s] Hematology includes WBC count, RBC count, hemoglobin, hematocrit, platelet count, and differential count (neutrophils, eosinophils, basophils, monocytes, lymphocytes, other cells).

[t] Screening laboratory test results must be obtained within 14 days prior to initiation of study treatment.

"Use of this document is governed by the terms of use on the first page of this document."

[u] Chemistry panel (serum or plasma) includes sodium, potassium, chloride, bicarbonate or total CO2, glucose, BUN or urea, creatinine, total protein, albumin, calcium, total bilirubin, alkaline phosphatase, ALT, AST, and LDH. Magnesium and phosphorus should be included at screening and as clinically indicated during study treatment.

[v] All women of childbearing potential will have a serum pregnancy test at screening. Urine pregnancy tests will be performed at the following specified subsequent visits for women of child-bearing potential (including premenopausal women who have had tubal ligation) and women not meeting the definition of postmenopausal: Day 1 of Cycles 1 – 5; Day 1 of Cycles 6, 8, 10, 12, 14, and 16; at treatment discontinuation (unless administered within 30 days); and at 3 months and 6 months after treatment discontinuation. For all other women, documentation must be present in medical history confirming that the patient is not of childbearing potential. If a urine pregnancy test is positive, it must be confirmed by a serum pregnancy test.

[w] TSH, free T3 (or total T3 for sites where free T3 is not performed), and free T4 will be assessed on Day 1 of Cycle 1 and every fourth cycle thereafter.

[x] At screening, patients will be tested for HIV, HBsAg, HBsAb, total HBcAb, and HCV antibody. If a patient has a negative HBsAg test and a positive total HBcAb test at screening, an HBV DNA test should be performed to rule out active HBV infection prior to initiation of study treatment. If a patient has a positive HCV antibody test at screening, an HCV RNA test should be performed to rule out active HCV infection prior to initiation of study treatment.

[y] Includes pH, specific gravity, glucose, protein, ketones, and blood; dipstick permitted.

[z] Not applicable for a site that has not been granted approval for RBR sampling. Performed only for patients at participating sites who have provided written informed consent to participate. Whole blood for DNA isolation will be collected from patients who have consented to optional RBR sampling at Week 1, Day 1. If, however, the RBR genetic blood sample is not collected during the scheduled visit, it may be collected as soon as possible (after randomization) during the conduct of the clinical study.

[aa] Tumor tissue should be of good quality based on total and viable tumor content (sites will be informed if the quality of the submitted specimen is inadequate to determine tumor PD-L1 status). For tissue sample provided at screening, an FFPE block or at least 20 unstained slides should be provided. Retrieval of tumor screening sample can occur outside the 28-day screening period. Fine-needle aspiration, brushing, cell pellets from pleural effusion, and lavage samples are not acceptable. For core-needle biopsy specimens, at least three cores should be submitted for screening and on-study evaluation. At least two cores should be submitted for disease recurrence specimens. See Section 4.5.8 and Appendix 3 for specific tissue sample requirements for each time point.

[bb] The unaffected breast should have been imaged within 60 days prior to randomization. The affected breast should be imaged within 28 days prior to randomization.

[cc] Mammograms of any remaining breast tissue should be performed at least annually.

[dd] Includes any medication (e.g., prescription drugs, over-the-counter drugs, vaccines, herbal or homeopathic remedies, nutritional supplements) used by a patient in addition to protocol-mandated study treatment from 30 days prior to initiation of study treatment (for the purposes of screening) until the treatment discontinuation visit. Record all prior anti-cancer therapies.

"Use of this document is governed by the terms of use on the first page of this document."

[ee] To be collected for both study arms. For patients in Arm B (Monitoring), only medications given for reportable adverse events as per protocol (see Section 5.3.1) as well as new anti-cancer treatments should be collected.

[ff] Medications related to the treatment of serious adverse events are to be reported during the follow-up period, as well as new anti-cancer treatments.

[gg] After informed consent has been obtained but prior to initiation of study treatment, only serious adverse events caused by a protocol-mandated intervention should be reported. After initiation of study treatment, all adverse events will be reported until 30 days after the last dose of study treatment or until initiation of new anti-cancer therapy, whichever occurs first, and serious adverse events and adverse events of special interest will continue to be reported until 90 days after the last dose of study treatment or until initiation of new anti-cancer therapy, whichever occurs first. After this period, all deaths, regardless of cause, should be reported. In addition, the Sponsor should be notified if the investigator becomes aware of any serious adverse event that is believed to be related to prior exposure to study treatment (see Section 5.6). The investigator should follow each adverse event until the event has resolved to baseline grade or better, the event is assessed as stable by the investigator, the patient is lost to follow-up, or the patient withdraws consent. Every effort should be made to follow all serious adverse events considered to be related to study treatment or trial-related procedures until a final outcome can be reported.

[hh] The initial dose of atezolizumab will be delivered over 60 ($\pm$15) minutes. Subsequent infusions will be delivered over 30 ($\pm$10) minutes if the previous infusion was tolerated without infusion-associated adverse events, or 60 ($\pm$15) minutes if the patient experienced an infusion-associated adverse event with the previous infusion.

[ii] Study drug administration during the maintenance phase for the atezolizumab-containing arm only.

jj After treatment discontinuation, information on survival follow-up and new anti-cancer therapy (including targeted therapy and immunotherapy) will be collected via telephone calls, patient medical records, and/or clinic visits (unless the patient withdraws consent or the Sponsor terminates the study). If a patient requests to be withdrawn from follow-up, this request must be documented in the source documents and signed by the investigator. If the patient withdraws from study, the study staff may use a public information source (e.g., county records) to obtain information about survival status only, where allowable by local regulation.

"Use of this document is governed by the terms of use on the first page of this document."

Appendix 3 Adaptive Multiple Test Design

The performance of the proposed design depends on several factors including the underlying proportion of (pCR) responders in each treatment arms for the subgroup S (PD-L1 IC1/2/3) and the complement group C (PD-L1 IC0). Simulations were carried out to evaluate the operation characteristics of the current design under a variety of conditions as follows.

Three simulation scenarios were considered with numerical details provided in Table 2.

- **Subgroup S with very strong credentials**

  Subgroup S has very strong credentials when convincing evidence indicates that the benefits of the treatment are limited to the subgroup S.

- **Subgroup S with strong credentials**

  Subgroup S has strong credentials when evidence for the predictive ability of the subgroup is convincing enough to assume that the treatment is more likely to be effective (and is probably more effective) in the subgroup S than in the complement subgroup C, but the evidence is not sufficiently compelling to rule out a clinically meaningful effect in complement subgroup C.

- **Subgroup S with weak credentials**

  If the subgroup S has weak credentials when convincing evidence for predictive value of the subgroup S is lacking and the treatment is expected to be broadly effective.

Table 1 Definition of Weak, Strong, and Very Strong Credentials on Subgroup S

Subgroup S Credentials	pCR proportion under treatment in Full population F (π_F)[a]	pCR Proportion under treatment in Subgroup S (π_S)	pCR Proportion under treatment in complement Subgroup C (π_C)	pCR Proportion response under Control ($\pi_{control}$)
Weak	0.68 (Δ_F=0.2)	0.68 (Δ_S=0.20)	0.68 (Δ_C=0.20)	0.48
Strong	0.6376 (Δ_F=0.1576)	0.68 (Δ_S=0.20)	0.60 (Δ_C=0.12)	0.48
Very Strong	0.5952 (Δ_F=0.1152)	0.68 (Δ_S=0.20)	0.52 (Δ_C=0.04)	0.48

[a]Assuming a prevalence of 0.47 of Subgroup S in the full population.

"Use of this document is governed by the terms of use on the first page of this document."

Table 2 Relative Frequencies of Decisions at Stage 1under Weak, Strong, and Very Strong Credentials on Subgroup S

Subgroup S Credentials	Δs	Δc	Relative Frequency[a,b,c]				
			Stop for efficacy	Stop for lack of efficacy	Continue only S	Continue only F	Continue S and F
Weak	0.20	0.20	0.64	0.04	0.08	0.14	0.10
Strong	0.20	0.12	0.46	0.10	0.22	0.11	0.11
Very Strong	0.20	0.04	0.33	0.17	0.38	0.05	0.06

[a]Assuming a prevalence of 0.47 of Subgroup S in the full population.
[b]Assuming probability of drop-out of 0.05 for each treatment arm.
[c]Frequency based on 100000 simulations.

Table 3 Rejection Probabilities under Weak, Strong, and Very Strong Credentials on Subgroup S

Subgroup S Credentials	Δs	Δc	Probability[a,b,c]		
			Reject $H_0^{\{F\}}$	Reject $H_0^{\{S\}}$	Reject $H_0^{\{F\}}$ or $H_0^{\{S\}}$ i.e. Overall Power
Weak	0.20	0.20	0.80	0.49	0.88
Strong	0.20	0.12	0.55	0.57	0.76
Very Strong	0.20	0.04	0.28	0.62	0.67

[a]Assuming a prevalence of 0.47 of Subgroup S in the full population.
[b]Assuming probability of drop-out of 0.05 for each treatment arm.
[c]Probability based on 100000 simulations.

Table 4 Conditional Rejection Probabilities if Stage 2 Activated under Weak, Strong, and Very Strong Credentials on Subgroup S

Subgroup S Credentials	Δs	Δc	Conditional Probability[a,b,c,d] (if stage 2 activated)		
			Reject $H_0^{\{F\}}$	Reject $H_0^{\{S\}}$	Reject $H_0^{\{F\}}$ or $H_0^{\{S\}}$ i.e. Overall "Go" Power
Weak	0.20	0.20	0.54	0.36	0.74
Strong	0.20	0.12	0.29	0.51	0.68
Very Strong	0.20	0.04	0.10	0.63	0.68

[a]Assuming a prevalence of 0.47 of Subgroup S in the full population.
[b]Assuming probability of drop-out of 0.05 for each treatment arm.
[c]Probability based on 100000 simulations.
[d]Conditional probability of event happening once the trial continues to enroll patients in stage 2 ("go").

"Use of this document is governed by the terms of use on the first page of this document."
