# Supplementary material for: Patient-reported outcomes from a randomized trial of neoadjuvant atezolizumab-chemotherapy in early triple-negative breast cancer
Source: NPJ Breast Cancer. 2022 Sep 19;8:108. doi: 10.1038/s41523-022-00457-3 (PMC9485121; doi:10.1038/s41523-022-00457-3)
Supplement: Supplementary file 2 — Supplemental material [file 41523_2022_457_MOESM2_ESM.pdf]

Supplementary figures and tables

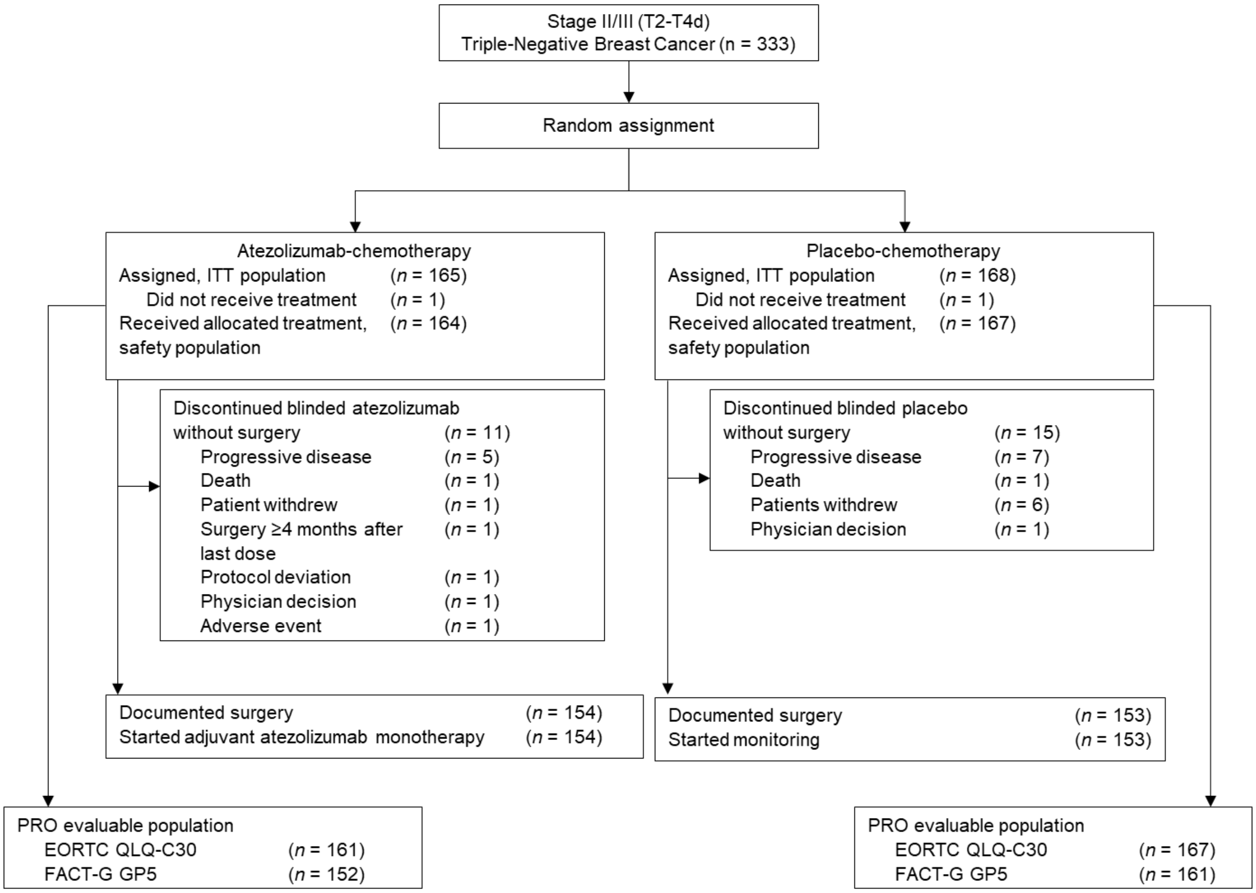

Supplementary Figure 1. CONSORT diagram for the patient-reported outcome analyses from the IMpassion031 study.

EORTC, European Organisation for Research and Treatment of Cancer; FACT-G, Functional Assessment of Cancer Therapy-General; ITT, intention to treat; PRO, patient-reported outcome.

Adapted from Mittendorf EA, et al. *Lancet* **2020**;396:1090-100.

A)

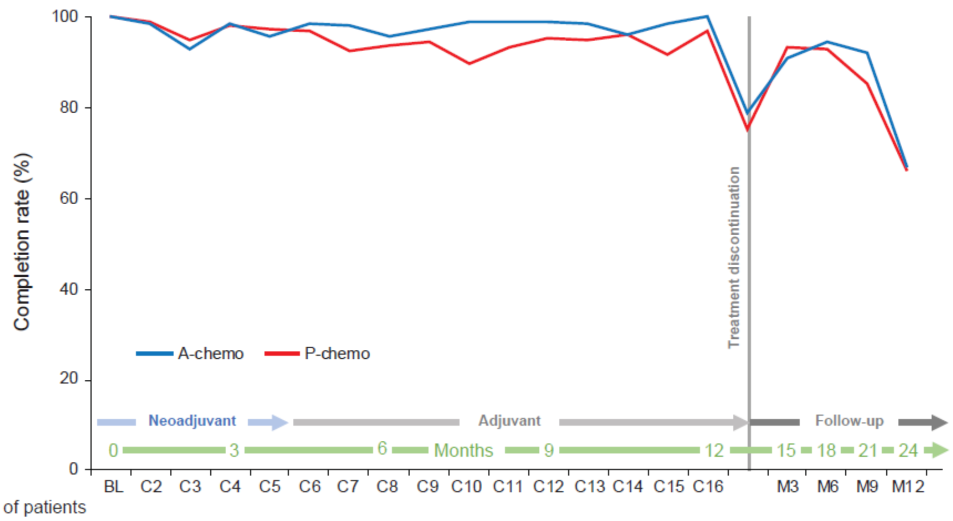

No. of patients																				
A-chemo	164	159	154	151	145	129	118	114	107	98	92	82	79	75	73	71	113	100	91	87	69
P-chemo	167	166	160	156	151	135	121	114	107	97	91	83	78	76	74	70	113	91	86	83	68

B)

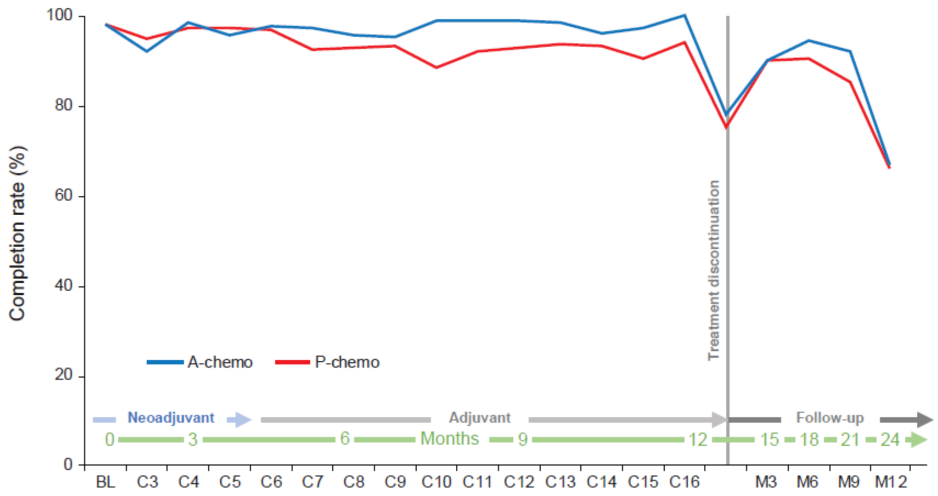

No. of patients																			
A-chemo	159	154	151	145	129	118	114	107	98	92	82	79	75	73	71	113	100	91	87	69
P-chemo	166	160	156	151	135	121	114	107	97	91	83	78	76	74	70	113	91	86	83	68

Supplementary Figure 2. Completion rates among all randomized patients.

Completion rates among all randomized patients for the (A) EORTC QLQ-C30 and (B) FACT-G GP5. A, atezolizumab; BL, baseline; C, cycle; M, month; P, placebo.

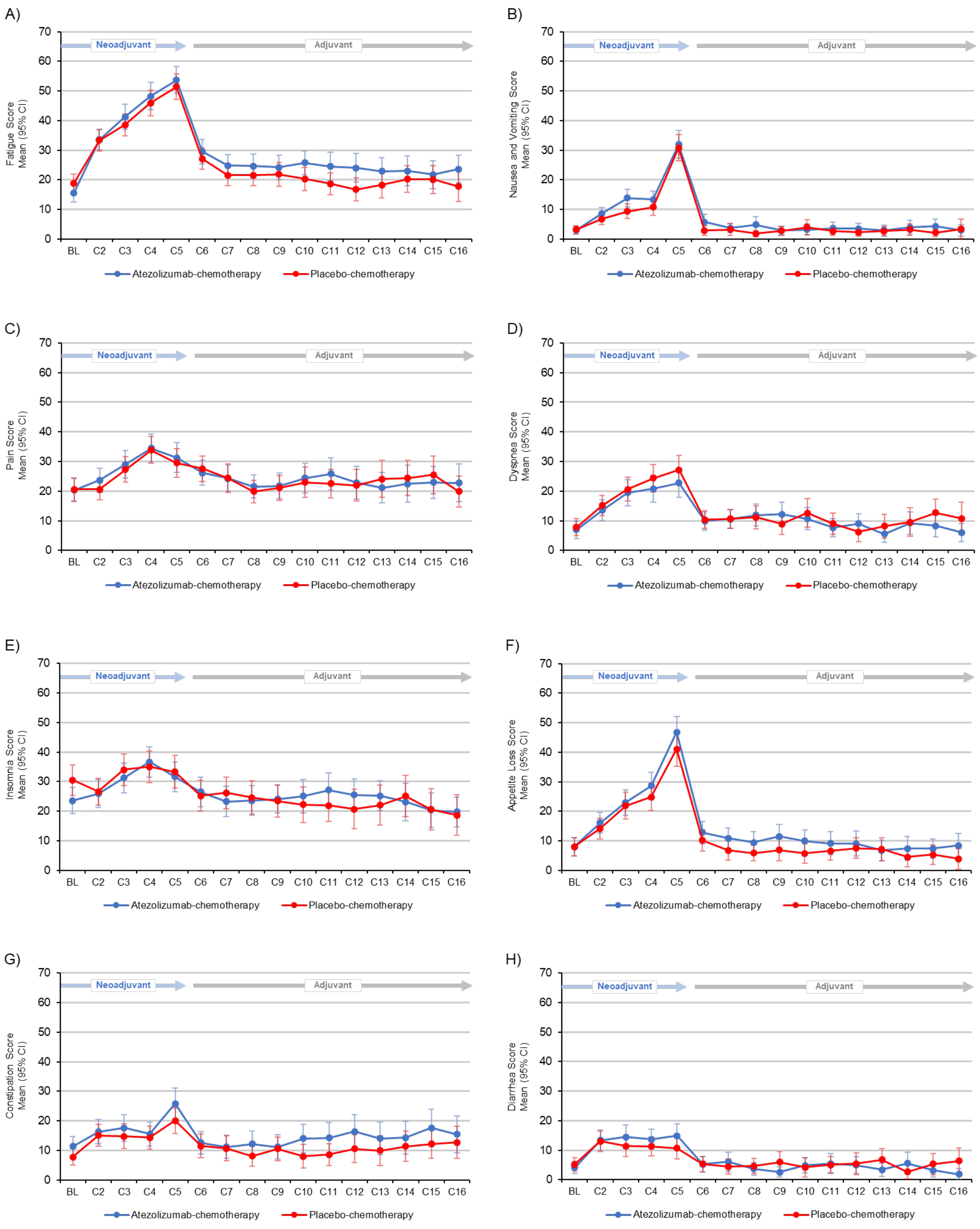

Supplementary Figure 3. Mean values at each time point for (A) fatigue, (B) nausea and vomiting, (C) pain, (D) dyspnea, (E) insomnia, (F) appetite loss, (G) constipation, and (H) diarrhea symptom scales measured using the EORTC QLQ-C30 instrument. Error bars indicate 95% confidence intervals.

BL, baseline; C, cycle.

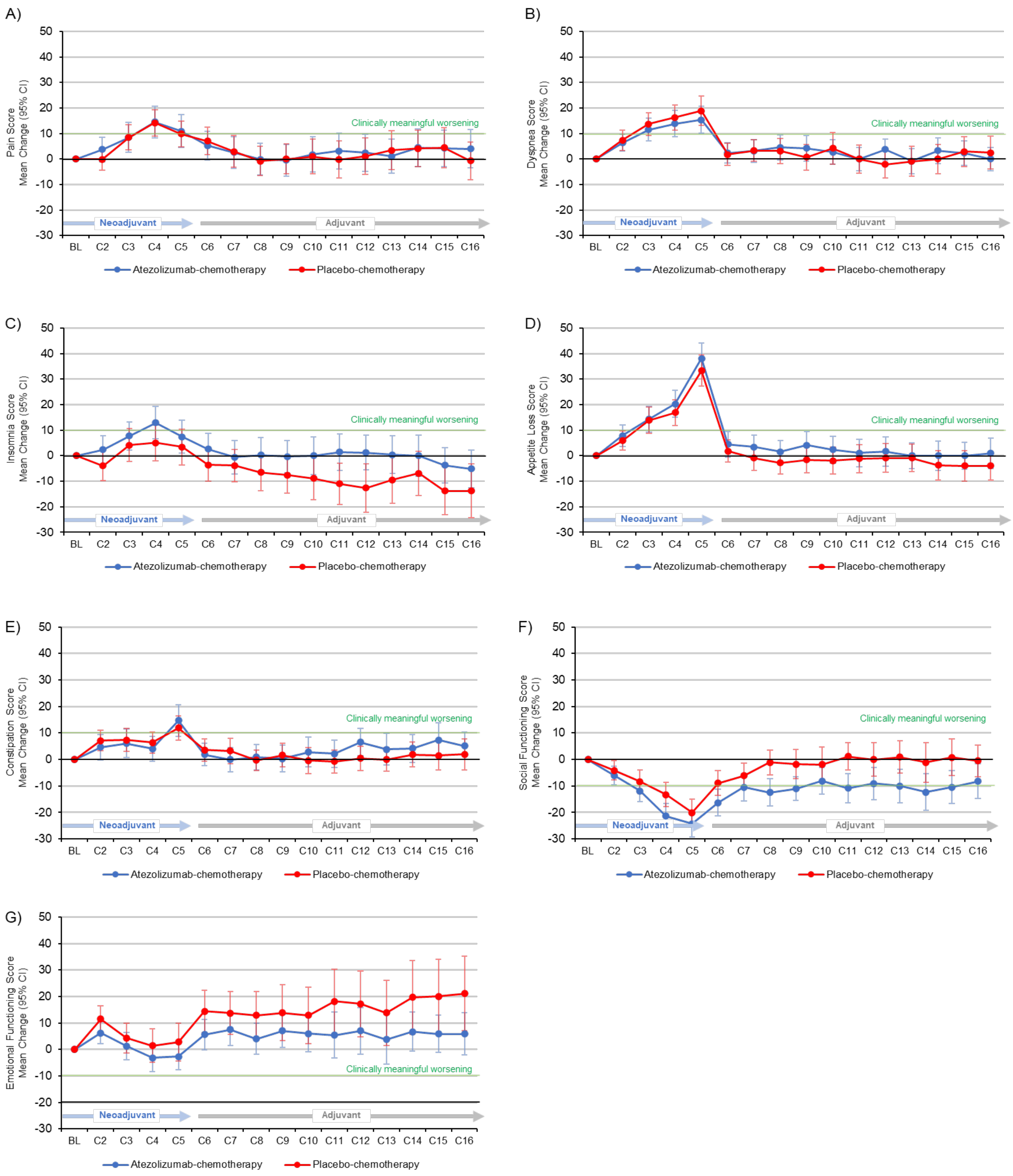

Supplementary Figure 4. Mean change from baseline values at each time point in (A) pain, (B) dyspnea, (C) insomnia, (D) appetite loss, (E) constipation symptom scales, and (F) social and (G) emotional functioning scales measured using the EORTC QLQ-C30. Error bars indicate 95% confidence intervals.

BL, baseline; C, cycle.

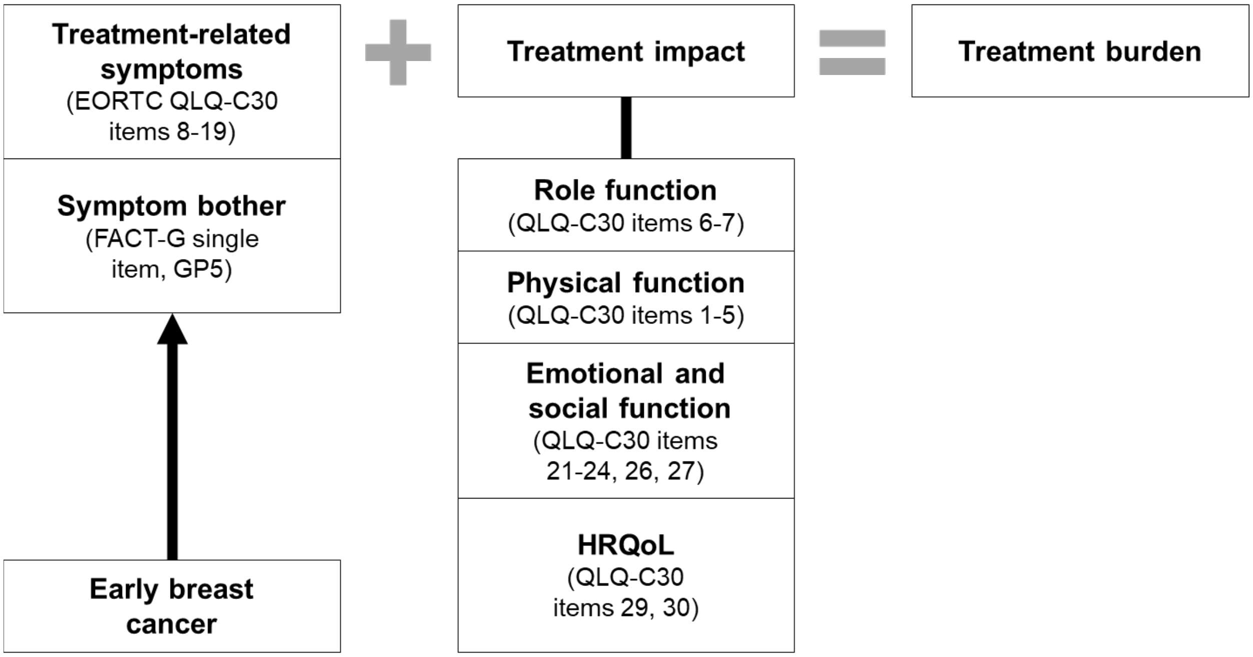

Supplementary Figure 5. The conceptual framework used to document treatment burden in patients with early triple-negative breast cancer. Specific items from the European Organisation for Research and Treatment of Cancer (EORTC) Quality of Life Questionnaire Core 30 (QLQ-C30) were used to assess treatment-related symptoms and the impact of treatment on functioning (role, physical, emotional, and social) and health-related quality of life (HRQoL). Symptom bother was assessed using the Functional Assessment of Cancer Therapy-General (FACT-G) single item GP5 (“I am bothered by side effects of treatment”).

Supplementary Table 1. PRO domains at baseline

PRO measure	Baseline value (95% CI)	
	Atezolizumab-chemotherapy (*n* = 161)	Placebo-chemotherapy (*n* = 167)
Physical functioning	90.9 (88.5-93.2)	90.0 (87.8-92.2)
Role functioning	89.4 (86.1-92.8)	88.9 (85.7-92.0)
GHS/QoL	79.2 (76.3-82.1)	76.5 (73.5-79.4)

GHS, global health status; PRO, patient-reported outcome; QoL, quality of life.

Supplementary Table 2. Summary of longitudinal analysis of EORTC QLQ-C30 domains

PRO measure	Treatment visit *P* value[1]
Physical functioning	0.36
Role functioning	0.77
GHS/QoL	0.50

[1]The mixed model for repeated measures across treatment visits from cycle 2 through month 18 of survival follow-up, adjusted for PD-L1 status, AJCC stage, age group, Eastern Cooperative Oncology Group score, treatment, time point, treatment interaction with time point, and baseline measurement interaction with time point.

AJCC, American Joint Committee on Cancer; GHS, global health status; PD-L1, programmed death-ligand 1; PRO, patient-reported outcome; QoL, quality of life.

Supplementary Table 3. Patients selecting each response option of the FACT-G GP5 Item (“I am bothered by side effects of treatment”) at baseline (cycle 2 day 1).

	Atezolizumab-chemotherapy (*n* = 165)	Placebo-chemotherapy (*n* = 168)
Responses	156	163
	**Patients selecting response option, *n* (%)**	
0 (“Not at all”)	30 (19.2)	39 (23.9)
1 (“A little bit”)	72 (46.2)	71 (43.6)
2 (“Somewhat”)	42 (26.9)	37 (22.7)
3 (“Quite a bit”)	9 (5.8)	13 (8.0)
4 (“Very much”)	3 (1.9)	3 (1.8)
